# *SLAMF8* expression predicts the efficacy of anti‐PD1 immunotherapy in gastrointestinal cancers

**DOI:** 10.1002/cti2.1347

**Published:** 2021-10-26

**Authors:** Qun Zhang, Lei Cheng, Yanmei Qin, Linghui Kong, Xiao Shi, Jing Hu, Li Li, Zhou Ding, Ting Wang, Jie Shen, Yang Yang, Lixia Yu, Baorui Liu, Chenchen Liu, Xiaoping Qian

**Affiliations:** ^1^ The Comprehensive Cancer Center Nanjing University Medical School Affiliated Nanjing Drum Tower Hospital Clinical Cancer Institute of Nanjing University Nanjing China; ^2^ Department of Pulmonary Medicine Shanghai Chest Hospital Shanghai Jiao Tong University Shanghai China; ^3^ Department of Respiratory and Critical Care Medicine Affiliated Hospital of Nantong University Nantong China; ^4^ Department of Pathology Nanjing University Medical School Affiliated Nanjing Drum Tower Hospital Nanjing China; ^5^ Department of Gastric Surgery Fudan University Shanghai Cancer Center Shanghai China

**Keywords:** anti‐PD1 therapy, biomarker, GI cancer, personalised immunotherapy, *SLAMF8*

## Abstract

**Objectives:**

Epstein–Barr virus (EBV) infection is associated with a better response to anti‐PD1 immunotherapy. We hypothesised that genetic alterations induced by EBV infection are responsible for the activation of key immune responses and hence are predictive of anti‐PD1 efficacy.

**Methods:**

With transcriptome data of gastric cancer (GC), we explored differentially expressed genes (DEGs) specific for EBV infection and performed coexpression network analysis using the DEGs to identify the consistent coexpression genes (CCGs) between EBV‐positive and EBV‐negative GC tissues. We selected the tag genes of the CCGs and validated them using RNA sequencing and immunohistochemistry. We established murine models and collected tissues from clinical patients to test the value of *SLAMF8* in predicting anti‐PD1 treatment. The location and expression of *SLAMF8* were characterised by multiplex immunofluorescence and quantitative PCR. Moreover, exogenous overexpression and RNA‐sequencing analysis were used to test the potential function of *SLAMF8*.

**Results:**

We identified 290 CCGs and validated the tag gene *SLAMF8* in transcriptome data of gastrointestinal cancer (GI). We observed that the T‐cell activation pathway was significantly enriched in high‐expression *SLAMF8* GI cancers. Higher SLAMF8 expression was positively associated with CD8 expression and a better response to anti‐PD1 treatment. We further observed dynamically increased expression of *SLAMF8* in murine models relatively sensitive to anti‐PD1 treatment. *SLAMF8* was mainly expressed on the surface of macrophages. Exogenous overexpression of SLAMF8 in macrophages resulted in enrichment of positive regulation of multiple immune‐related pathways.

**Conclusion:**

Higher SLAMF8 expression may predict better anti‐PD1 immunotherapy efficacy in GI cancer.

## Graphical abstract

In this study, we found that *SLAMF8* was involved in activating the antitumour immune response in gastrointestinal (GI) cancer. Thus, higher *SLAMF8* expression may predict better anti‐PD1 immunotherapy efficacy in GI cancer.

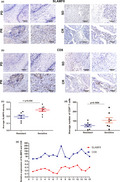

## INTRODUCTION

Gastrointestinal (GI) cancer has a high incidence and mortality rate and remains a major public health problem worldwide,^1^ and the 5‐year survival of advanced GI cancer patients remains as poor as 10%.^2^, ^3^ Recently, several immune checkpoint inhibitors, specifically anti‐PD1 monoclonal antibodies (mAbs), have been approved for the first‐line treatment of several cancers by the US Food and Drug Administration. However, there were notable differences in anti‐PD1 treatment efficacy among patients with GI cancers.^4^, ^5^ Therefore, the identification of novel biomarkers to personalise anti‐PD1 treatment is of utmost importance. Recent studies have suggested that PD‐L1 expression, tumor mutation burden and microsatellite instability status of tumor tissues are potential predictors of the efficacy of anti‐PD1 therapy.^6^, ^7^, ^8^, ^9^ The implementation of biomarker‐guided personalised anti‐PD1 therapy has improved the efficacy in patients with advanced solid tumors, with an increased overall response rate of 34.1% for patients with high‐expression PD‐L1^10^ and 53% (42–64%) for those with microsatellite instability‐high tumors.^11^ Because of these novel findings, US Food and Drug Administration and National Comprehensive Cancer Network guidelines have recommended pembrolizumab, an anti‐PD1 agent, for the treatment of patients with microsatellite instability‐high or DNA mismatch repair deficiency, regardless of the site of primary tumors. This considerable achievement has led to attempts to find more novel biomarkers to guide anti‐PD1 immunotherapy in a personalised way, which is also the focus in a series of recent studies.^12^, ^13^, ^14^, ^15^

The Cancer Genome Atlas database has shown the well‐established genetic landscape of gastric cancer (GC), in which GC is classified into four subtypes by comprehensive molecular characterisation: Epstein–Barr virus (EBV)‐infected tumors, microsatellite unstable tumors, genetically stable tumours and chromosomal unstable tumors. EBV infection was reportedly associated with a better prognosis, likely by the activated immune response in the presence of EBV infection.^16^, ^17^ Recent focus on immunotherapy of GC has also indicated that tumours with EBV infection have a relatively high infiltration of T lymphocytes and PD‐L1 mRNA expression, leading to a good response to anti‐PD1 immunotherapy.^18^, ^19^ Specifically, it was reported that the overall response rate of patients with EBV^+^ GC was as high as 100%.^20^ However, the molecular implication underlying the good response remains unclear, and EBV^+^ GC comprises only 5–10% of all GCs. We hypothesised that there were specific genetic alterations induced by EBV infection, which were responsible for the activation of key antitumor immune responses and hence good responses to anti‐PD1 treatment. Therefore, the identification of biomarkers associated with the antitumor immune response based on the EBV‐related gene signature is crucial. These biomarkers represent the antitumor immune response and may be equally applied to predict anti‐PD1 efficacy in patients with EBV^−^GI cancers. To test our hypothesis, we used a multiple‐stage approach to identify the consistent coexpression genes (CCGs) between EBV^+^ and EBV^−^ GC patients from an EBV‐specific gene signature. Signalling lymphocytic activation molecule family 8 (*SLAMF8*) was further identified as the tag gene of the CCGs.

Signalling lymphocytic activation molecule family members are expressed on the surface of most immune cell types and regulate their functions. Similar to PD1, SLAMF8 is mainly expressed on the surface of immune cells.^21^ Previous reports indicated that SLAMF8 was induced by IFN‐γ stimulation and that its high expression was correlated with the enhanced T‐cell‐mediated immune phenotype.^22^, ^23^ However, no studies have explored the potential relationship between SLAMF8 expression and the efficacy of anti‐PD1 therapy in GI cancer. Here, we evaluated the role of *SLAMF8* might play in predicting the efficacy of anti‐PD1 treatment in GI cancers, which will provide new insights into individualised immunotherapy for patients with GI cancer.

## RESULTS

### The workflow of the present study (Figure␣1)

#### Molecular signature specific for EBV infection in GC patients

We analysed the mRNA microarray dataset GSE51575 from the Gene Expression Omnibus (GEO) database, with comprehensive mRNA expression profiling of 12 paired cancer and adjacent normal tissues from patients with EBV^+^ and 14 other paired tissues from EBV^−^ GC patients. As a result, 1846 and 3190 genes were identified to be differentially expressed in EBV^+^ and EBV^−^ GC tissues compared with corresponding adjacent normal tissues. To identify the signature specific for EBV infection, we excluded the 1,058 DEGs contained in EBV^−^ tumors from the DEGs in EBV^+^ tumors, leaving a 788‐gene signature (Supplementary table 1). Interestingly, we found that this identified signature contained 7 immune checkpoint genes, including *HAVCR2*, *IDO1*, *TNFSF14*, *CTLA4*, *TIGIT*, *IDO2* and *CD80*. The expression of these 7 checkpoint genes was not only upregulated in EBV^+^ GC tissues compared with adjacent normal tissues (Supplementary figure 1a) but also obviously increased in EBV^+^ GC compared with EBV^−^ GC, indicating the potential involvement of the signature in driving the immune checkpoint. Although the hotspot immune checkpoint gene *PD1* (also termed PDCD1) was not included in the identified signature, its expression was also significantly increased in EBV^+^ GC tissues compared with EBV^−^ GC tissues (Supplementary figure 1b). This immune signature potentially induced by EBV infection was then used for further analysis.

**Figure 1:**
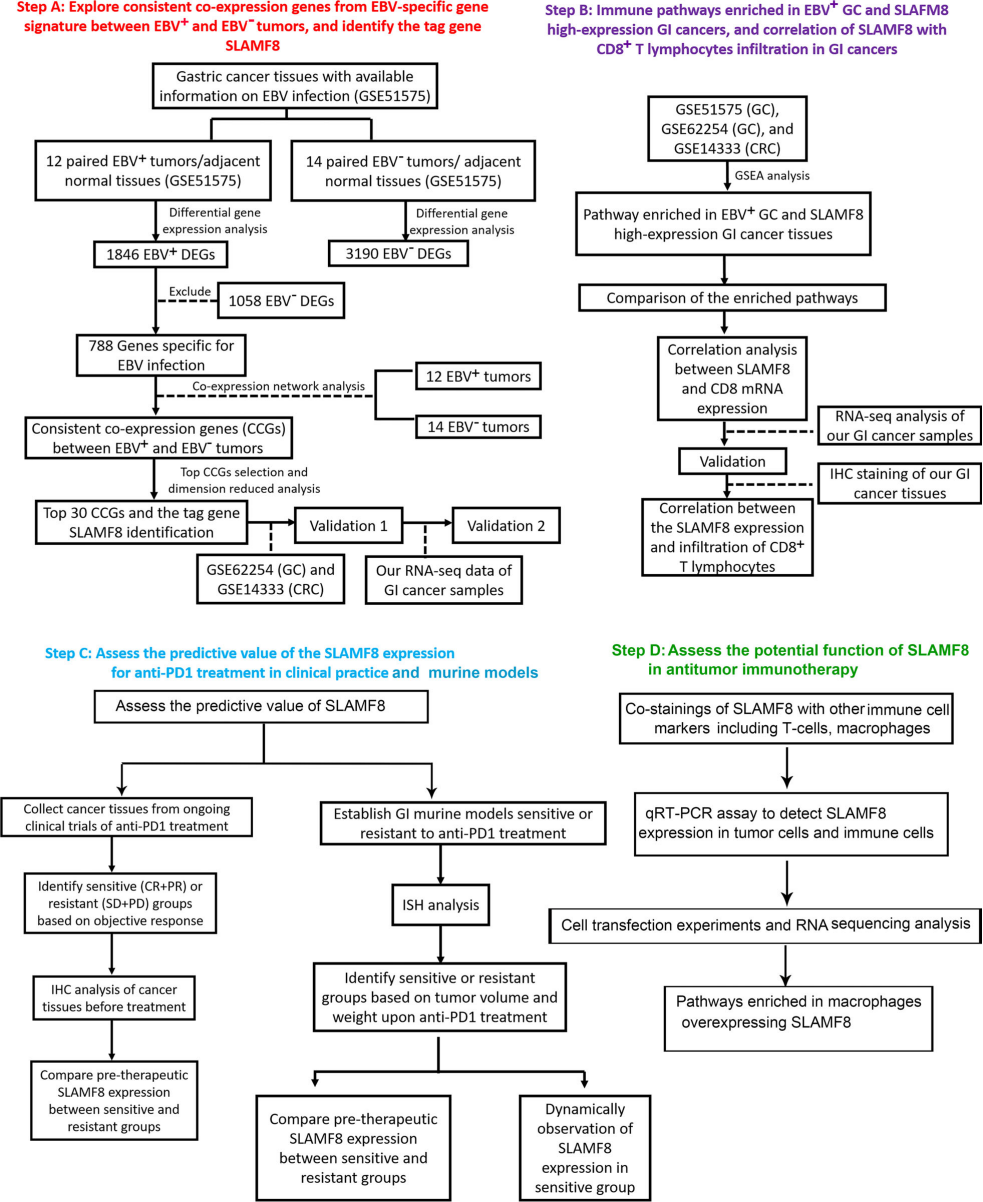
The workflow of the present study.

#### Identification and validation of the consistent coexpressed genes (CCGs) and their tagged gene *SLAMF8* in GI cancer tissues

**Figure 2:**
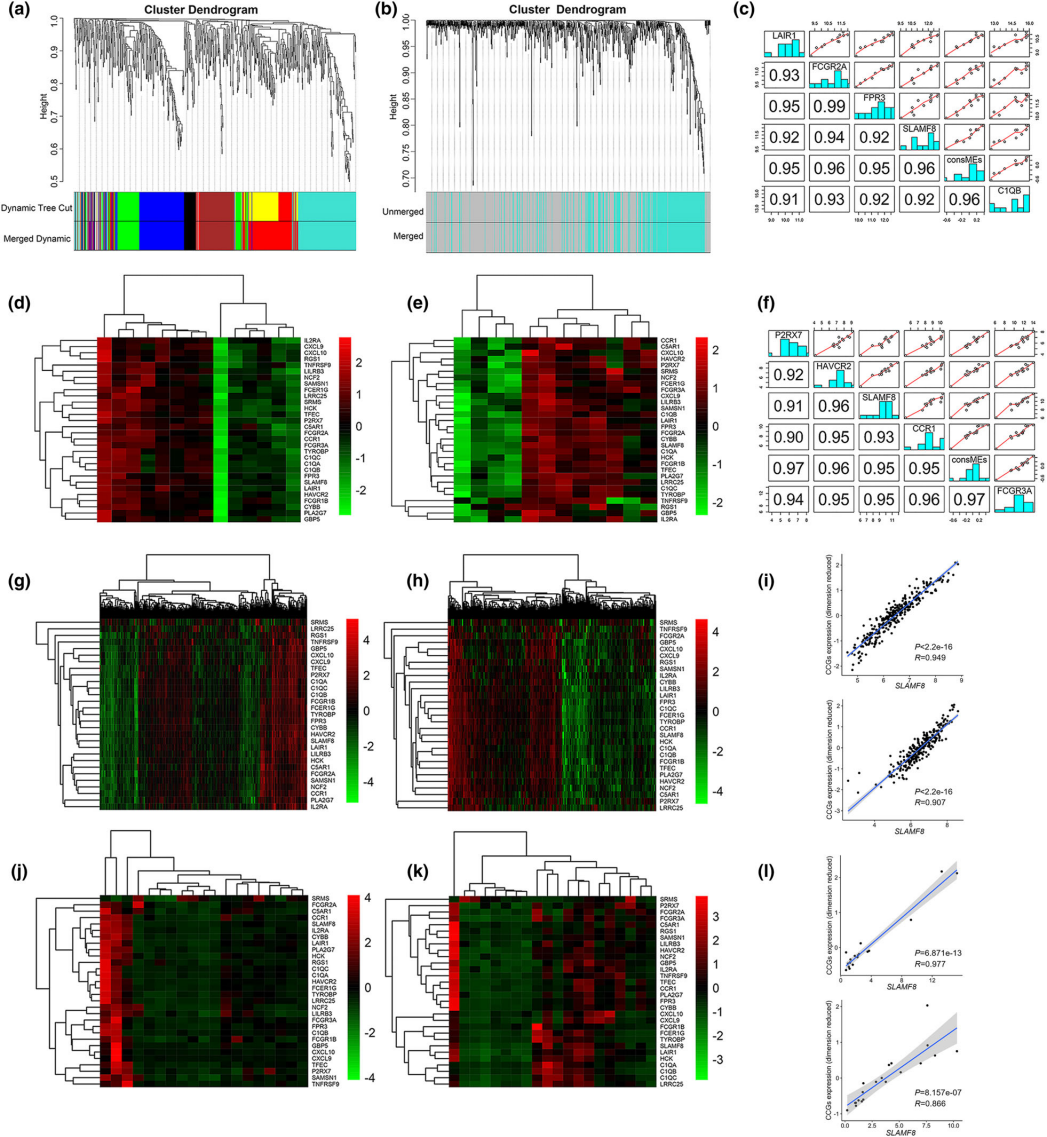
Identification and validation of the consistent coexpression signature and the tag gene *SLAMF8*. Genes coexpressed in EBV^+^ GC **(a)** the squares with different colours on the bottom of the graph represent different coexpressed gene groups; identification of CCGs between EBV^+^ and EBV^−^ GC tissues using the squares of coexpressed genes in EBV^+^ GC **(b)**; good performance of the top 30 CCGs in clustering EBV^+^ **(d)** and EBV^−^ GC **(e)**; top 5 correlations of the CCGs with consistent consME in EBV^+^ **(c)** and EBV^−^ GC **(f)** identified *SLAMF8* as the tag gene of CCGs. Note: ConsME values were derived from primary component analysis. The top 30 CCGs in clustering GC **(g)** and CRC **(h)** tissues using GSE62254 and GSE14333 datasets, respectively, from the GEO database. The significant correlation of mRNA expression between *SLAMF8* and the 30 top CCGs (dimension reduced) in GC (above) and CRC (below) tissues by analysis of GSE62254 and GSE14333 datasets, respectively **(i)**. RNA‐sequencing analysis of GI cancer tissues from our centre also showed that the 30 top CCGs had good performance in clustering GC (*n* = 19, **j**) and CRC tissues (*n* = 20, **k**), and the significant correlation between mRNA expression of *SLAMF8* and the 30 top CCGs (dimension reduced) in GC (above) and CRC (below) tissues **(l)**.

Because the phenotype of EBV infection was attributed to only a small subset of patients, we next explored whether genes from the EBV‐specific signature were able to cluster both EBV^+^ and EBV^−^ GC tissues well so that we could identify the genes that were able to reflect the antitumor immune response irrespective of EBV infection. Using network coexpression analysis, we identified 290 genes from the 788‐gene signature that presented consistent coexpression between EBV^+^ and EBV^−^ GC tissues (Figure 2a and b). We then extracted the 30 top CCGs from the 290 CCGs to cluster the GC tissues. This 30‐gene set contained genes involved in the initiation of inflammation (*CCR1*, *CXCL10* and *CXCL9*), T‐cell activation (*TNFRSF9*, *FCER1G*, *FCGR2A*) and NK cell markers (*FCGR3A*). We found that the 30 CCGs had good performance in clustering GC tissues, regardless of EBV infection (Figure 2d and e). In the WGCNA package, there was a module eigengene value derived from principal component analysis to be used to represent the characterisation of the CCG module. To select the tagged genes from the top 30 CCGs, we correlated the gene expression with the module eigengene value of the CCG signature. Of the 30 CCGs, *SLAMF8* was identified as the tag gene because of its top correlation with the module eigengene value in both EBV^+^ (*r* ^2^ = 0.96, *P* < 0.001, Figure 2c) and EBV^−^ GC tissues (*r* ^2^ = 0.95, *P* < 0.001, Figure 2f).

We next validated the performance of the top 30 CCGs in clustering GC and colorectal cancer (CRC) tissues using public data from the GEO database. We also performed RNA sequencing for 19 GC and 20 CRC tissues from our centre for validation upon quality control. As expected, the promising clustering ability for the top 30 CCGs was successfully replicated in GC and CRC tissues by both public datasets (Figure 2g for GC and h for CRC) and our RNA‐sequencing data (Figure 2j for GC and k for CRC). The tag gene *SLAMF8* was also listed to have the top correlation with dimension reduced expression of the top 30 CCGs, as indicated by the correlation analysis in public datasets (*r* ^2^ = 0.90, *P* < 0.001 for GC and *r* ^2^ = 0.82, and *P* < 0.001 for CRC, Figure 2i) and our RNA‐sequencing data (*r* ^2^ = 0.95, *P* < 0.001 for GC and *r* ^2^ = 0.75, *P* < 0.001 for CRC, Figure 2l). We analysed the protein expression of *SLAMF8* in a cohort of 20 EBV^+^ and 20 EBV^−^ GC tissues and found that *SLAMF8* expression was significantly higher in EBV^+^ GC tissues than in EBV^−^ GC tissues (*P* = 0.021, Supplementary figure 2a, b).

We extracted clinical information from the GSE62254 dataset and found that the expression levels of the top 30 CCGs were markedly increased in EBV^+^ GC tissues compared with EBV^−^ GC tissues (*P* < 0.001, Supplementary table 2). The top 30 CCGs were also found to have a higher expression level in tumors of GC patients with metastatic disease than in those without metastatic disease (*P* = 0.033, Supplementary table 2). With adjustment for age, sex, Lauren classification and pathologic TNM stage, EBV status and adjuvant treatment, the Cox regression analysis showed that compared with low expression, high expression of the CCGs was independently associated with a 44% decreased risk of death (HR = 0.56, 95% CI = 0.35–0.92, *P* = 0.021, Supplementary table 3). In addition, survival analysis also revealed a significant association between expression solely of *SLAMF8* and survival, with a death risk decreased by 50% for high *vs*. low expression (HR = 0.50, 95% CI = 0.31–0.79, *P* = 0.004, Supplementary table 3).

#### High‐expression *SLAMF8* in GI cancer may enrich the pathways involved in the antitumor immune response

On the basis that *SLAMF8* tagged the coexpression signature that was able to cluster the GI cancer tissues irrespective of EBV infection, we tested whether high‐expression *SLAMF8* in GI cancer had an activated phenotype for antitumor immune response. We performed gene set enrichment analysis (GSEA) using three transcriptome datasets from the GEO database. The results revealed that the pathways enriched in GI cancer tissues with high *SLAMF8* expression were associated with an activated antitumor immune response, including T‐cell activation, antigen presentation and positive regulation of IFN‐γ production (Supplementary figure 3a), and most of them intersected with the pathways enriched in EBV^+^ GC tissues (Supplementary table 4). We visualised the antigen presentation (Supplementary figure 3b), T‐cell proliferation (Supplementary figure 3c) and T‐cell activation pathways (Supplementary figure 3d), the three key pathways for activation of the antitumor immune response, as the representation of the intersected enrichment results between *SLAMF8* high‐expression GI cancer tissues and EBV^+^ GC tissues.

#### High expression of *SLAMF8* is associated with increased infiltration of CD8^+^ T lymphocytes in GI cancer tissues

**Figure 3:**
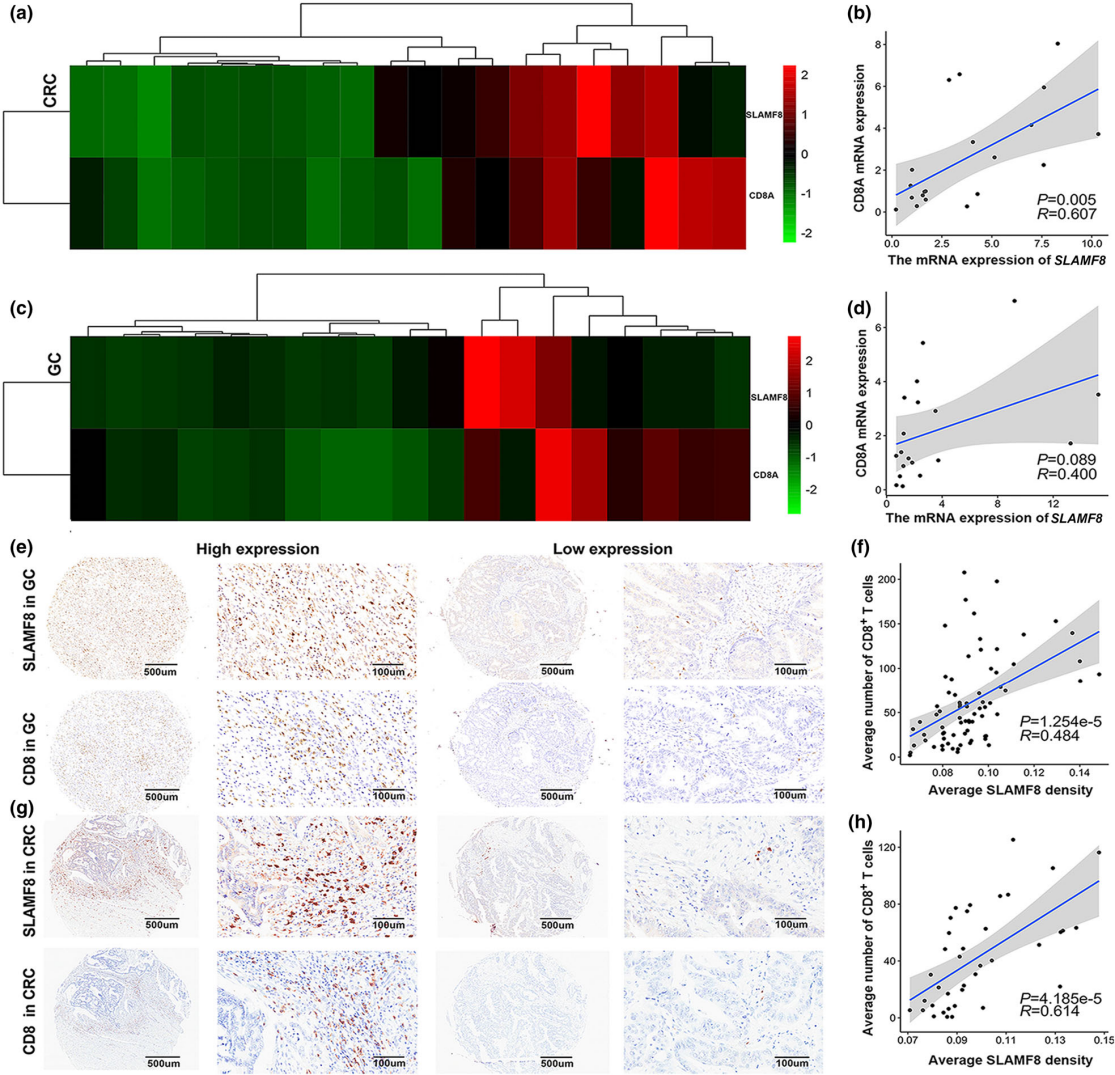
Higher expression of *SLAMF8* was associated with increased infiltration of CD8^+^ T lymphocytes in GI cancer tissues. The heatmap shows the mRNA expression of *SLAMF8* and *CD8* in CRC **(a)** and GC **(c)** tissues and the significant correlation between the two genes using RNA‐sequencing data of CRC (*n* = 20, **b**) and GC (*n* = 19, **d**) tissues from our centre. Representative images of SLAMF8 and CD8 IHC staining in GC (*n* = 74, **e**) and CRC (*n* = 38, **g**) tissues using GC and CRC tissue microarrays, respectively, with scale bars of 500 μm (left) and 100 μm (right). Quantification of IHC analysis in GC **(f)** and CRC **(h)** tissues showed a significant correlation between SLAMF8 and CD8 expression.

CD8^+^ T lymphocytes are known to be central to adaptive antitumor immune responses.^24^, ^25^ Accumulating evidence suggests that infiltration of CD8^+^ T lymphocytes in tumors is a promising indicator for a better response to anti‐PD1 therapy.^26^, ^27^ To further demonstrate the role the tag gene *SLAMF8* may play in the antitumor immune response against GI cancer, we first assessed the mRNA expression levels of *SLAMF8* and CD8A using the three public datasets above and observed a positive and significant correlation between them in GI cancers (Supplementary figure 4a–c, *P* < 0.001 for all). We also validated the significant and positive association between the mRNA expression of *SLAMF8* and CD8A in our RNA‐sequencing data of CRC with 20 tissue samples (Figure 3a and b, *P* = 0.005), but we only observed borderline significance (Figure 3c and d, *P* = 0.089) for this association in the RNA‐sequencing data of 19 GC tissue samples, probably because of the limited sample size. We further performed an immunohistochemistry (IHC) analysis using a tissue microarray and found that high protein expression of SLAMF8 was associated with increased CD8^+^ T lymphocyte infiltration in both GC (*n* = 74) and CRC (*n* = 38) tissue samples and vice versa (Figure 3e for GC and g for CRC). The quantified IHC results also suggested a significant and positive correlation between CD8 and SLAMF8 expression at the protein level in both GC (Figure 3f, *P* < 0.001) and CRC (Figure 3h, *P* < 0.001) tissues.

#### Predictive ability of *SLAMF8* expression for anti‐PD1 efficacy in clinical practice

**Figure 4:**
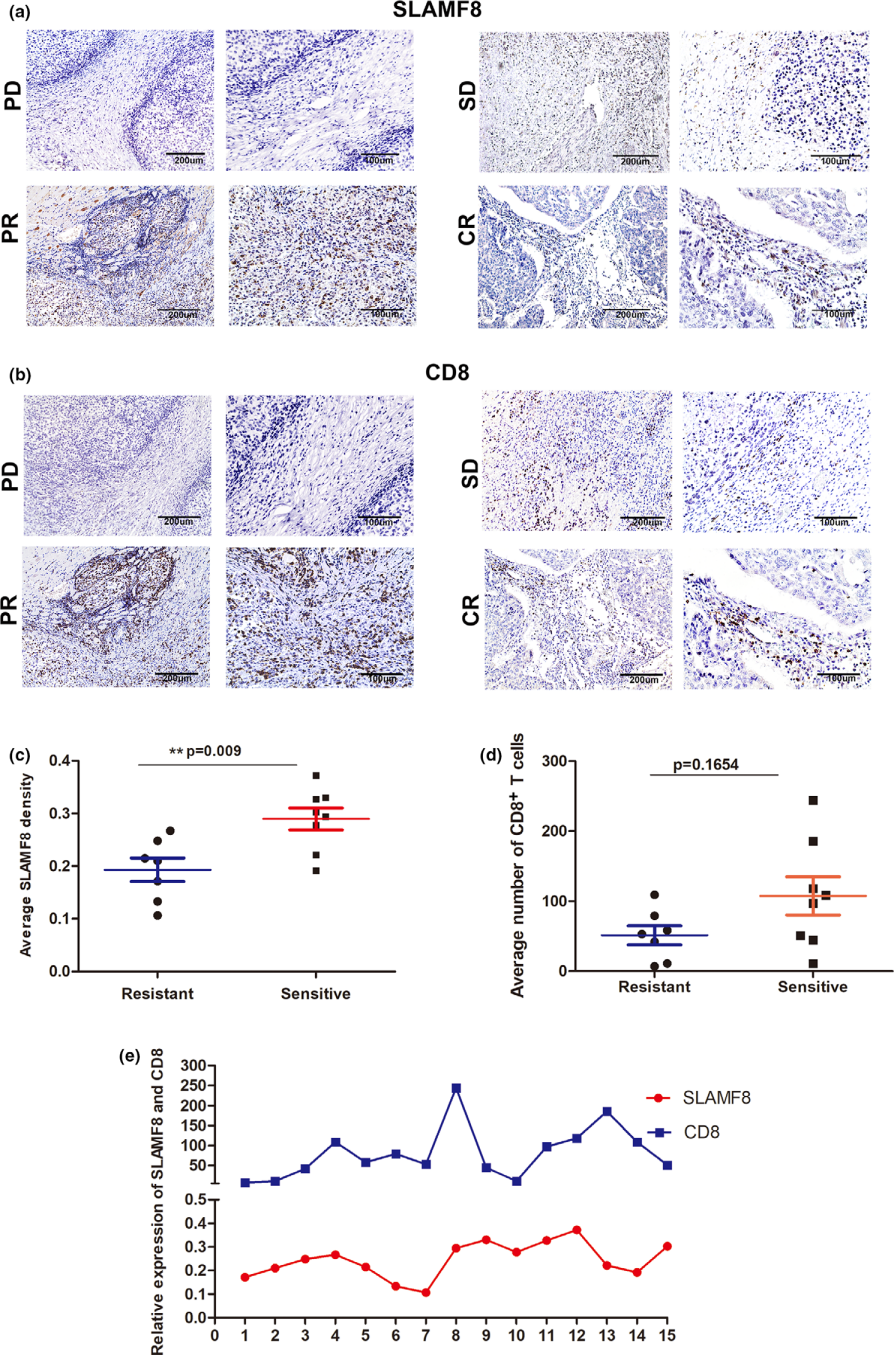
Predictive ability of *SLAMF8* for anti‐PD1 treatment in clinical practice. Representative image of pretherapeutic *SLAMF8* **(a)** and CD8 **(b)** protein expression stained by IHC in cancer tissues, with scale bars of 200 μm (left) and 100 μm (right), from patients who obtained the best response of CR, PR, SD and PD, respectively. Comparison of pretherapeutic SLAMF8 **(c)** and CD8 **(d)** protein expression between patients with tumors sensitive to (CR + PR) and those with tumors relatively resistant to (SD + PD) anti‐PD1 treatment (*n* = 8 (CR + PR) for the sensitive group and *n* = 7 (SD + PD) for the resistant group, **P < 0.01). The relationship between SLAMF8 and CD8 expression **(e)**. Nonstatistical images are from one experiment that is representative of three separate experiments.

To test the predictive impact of SLAMF8 on the efficacy of anti‐PD1 treatment, we systemically included 15 cancer patients for clinical validation, including 12 patients with GC and 1 with CRC. To maintain statistical power, we also included 2 patients with cancers out of GI, including 1 patient with malignant melanoma and 1 with chest wall sarcoma, for analysis. Of these patients, 7 had obtained the best objective response of partial response (PR), 6 had progressive disease (PD), 1 had stable disease (SD), and 1 had a complete response (CR). Patients who had the best response of PR and CR were divided into the relatively sensitive group, while those with the best response of PD and SD were divided into the relatively resistant group. Importantly, using IHC staining, we observed a higher pretherapeutic expression of SLAMF8 proteins in the sensitive group than in the resistant group (Figure 4a and b). Quantitative analysis further demonstrated a higher pretherapeutic expression of SLAMF8 in the sensitive group than in the resistant group (Figure 4c, *P* = 0.009), but the difference in the expression levels of CD8 did not reach statistical significance, likely induced by the limited sample size (Figure 4d, *P* = 0.1654). Moreover, we observed a positive correlation trend between the expression of SLAMF8 and CD8 (Figure 4e). These results provide some preliminary evidence relevant to clinical practice for the potential use of SLAMF8 expression in predicting the response to anti‐PD1 treatment.

#### Predictive ability of *SLAMF8* expression for anti‐PD1 efficacy *in vivo*

**Figure 5:**
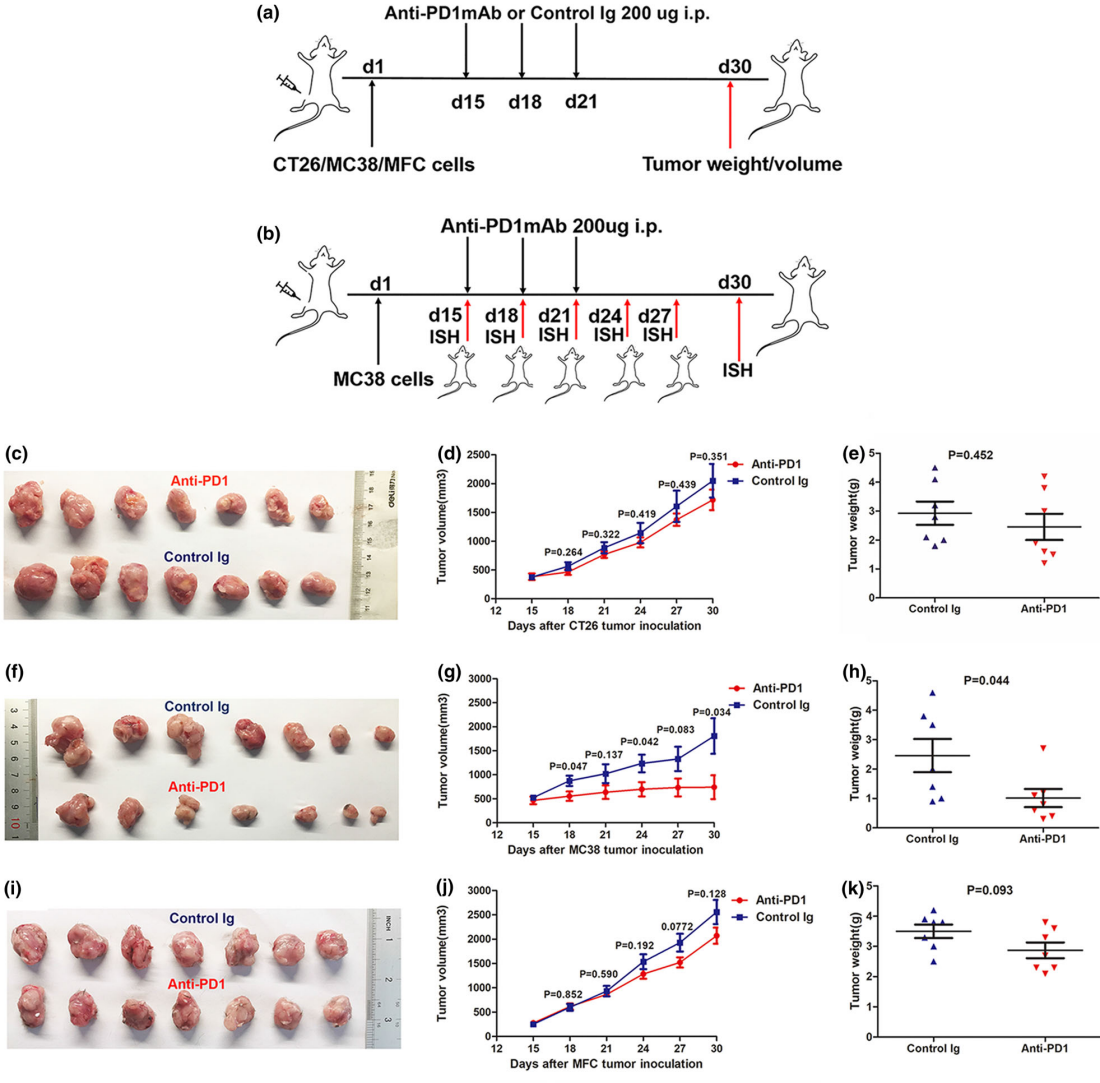
The establishment of tumor models resistant or sensitive to anti‐PD1 treatment. Schematic model of establishment and further treatment process for mice‐bearing murine CT26 (CRC), MC38 (CRC) and MFC (GC) tumors (*n* = 7 per group). Mice were treated with control murine Ig (2A3) or anti‐PD‐1 mAb on Days 15, 18 and 21 and then sacrificed on day 30 for analysis of tumor weight and volume **(a)**. Process diagram of MC38 tumor models treated with anti‐PD1 mAb for dynamic ISH analysis for *SLAMF8* expression. Tumor‐bearing mice were injected with anti‐PD1 mAb on Days 15, 18 and 21 and were sacrificed on Days 15, 18, 21, 24, 27 and 30. Then, the resected tumors were submitted for ISH staining **(b)**. Representative images of CT26 **(c)**, MC38 **(f)** and MFC **(i)** tumor models treated with anti‐PD1 mAb and control Ig (2A3). Tumor growth curves and tumor weight plots of CT26 **(d, e)**, MC38 **(g, h)** and MFC **(j, k)** tumors. Nonstatistical images are from one experiment that is representative of three separate experiments.

To further explore the potential reason for the predictive value of *SLAMF8* expression in anti‐PD1 efficacy, we first conducted *in vivo* analysis to screen murine models relatively sensitive and resistant to anti‐PD1 treatment (Figure 5a). According to genomic characterisation of the CT26 cell line by Castle *et al*., this cell line carried the KRAS mutation but had no mutations in the *TP53, BRAF, POLD1, PIK3CA* and *MMR* genes. However, MC38 was a hypermutated cell line with missense mutations in *TP53, BRAF, POLD1* and MMR gene *MSH3*. Song *et al*. reported that there was a certain amount of T cells inside the MC38 tumor, while those inside the CT26‐FL3 (a subtype of CT26 cells) tumor were minimal.^29^ Consistent with their results, we also observed more CD8^+^ T cells within MC38 tumors than in CT26 tumors (Supplementary figure 5). As expected, we observed resistance to anti‐PD1 treatment in CRC murine models constructed with the CT26 cell line, with no differences in the volume and weight of tumors between the anti‐PD1 mAb‐treated and control groups (Figure 5c–e, *P* > 0.05 for all). However, we observed a significant decrease in tumor volume and weight in response to anti‐PD1 mAb in CRC murine models constructed with the MC38 cell line. (Figure 5f–h), further supporting the results from previous studies.^30^, ^31^ We also established a GC murine model with the murine GC cell line MFC, which has been widely used in cancer immunotherapy research.^32^, ^33^ During the anti‐PD1 treatment, only a trend towards a decreased tumor volume and weight was observed in mice‐bearing MFC cells compared with the control group, but the results did not reach statistical significance (Figure 5i–k). Collectively, these results confirmed that CT26 murine tumors were the most resistant, but MC38 was the one most sensitive to anti‐PD1 treatment. Therefore, these two CRC murine models were optimal selections for further investigations.

**Figure 6:**
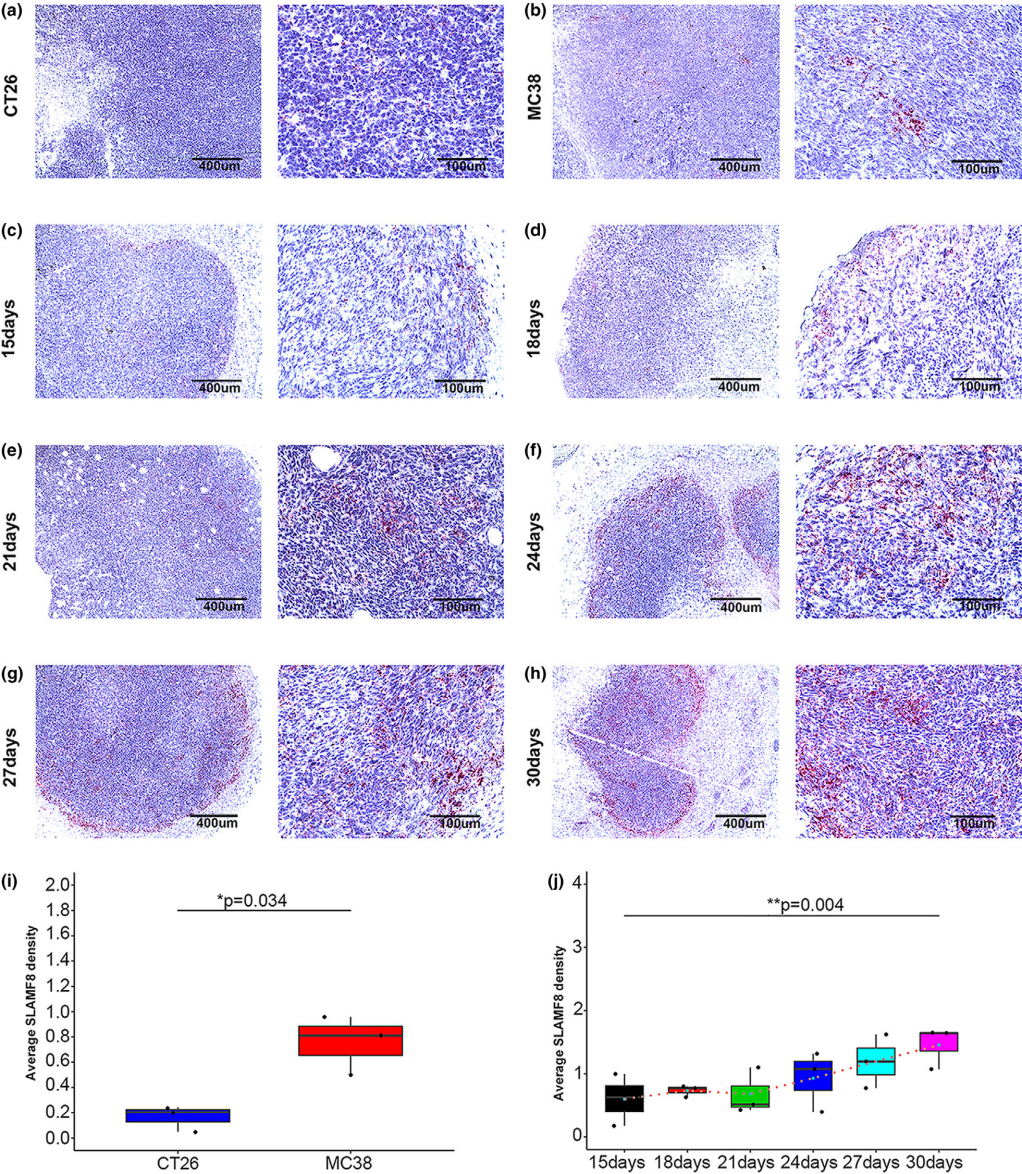
Predictive ability of *SLAMF8* for anti‐PD1 treatment *in vivo*. Representative image of ISH staining of pretherapeutic *SLAMF8* mRNA in murine tumor tissues established by the CT26 CRC cell line **(a)** and MC38 CRC cell line **(b)**, with scale bars of 400 μm (left) and 100 μm (right) (*n* = 3 per group); dynamic change in *SLAMF8* mRNA expression in MC38 tumor tissues from initial anti‐PD1 treatment (15 days) to 30 days as represented by the ISH images, with scale bars of 400 μm (left) and 100 μm (right) **(c–h)**; comparison of pretherapeutic *SLAMF8* mRNA expression between CT26 and MC38 cancer tissues using quantified data from ISH analysis (*n* = 3 per group, *P < 0.05) **(i)**; comparison of *SLAMF8* mRNA expression among different time points during anti‐PD1 treatment in MC38 tumor tissues. (*n* = 3 for murine tumor models collected at each time point, **P < 0.01) **(j)**. Nonstatistical images are from one experiment that is representative of three separate experiments.

We next visualised the expression of *SLAMF8* in CT26 and MC38 murine models in the control group. As expected, we observed a higher pretherapeutic expression of SLAMF8 in MC38 murine models than in CT26 murine models (Figure 6a and b), and the quantitative analysis showed statistical significance (Figure 6i, *P* = 0.034). Given that higher SLAMF8 expression was associated with CD8 expression, we hypothesised that the expression of SLAMF8 might be altered dynamically in tumors that were sensitive to anti‐PD1 treatment. To test this hypothesis, SLAMF8 expression was dynamically tested by in situ hybridisation (ISH) methods during anti‐PD1 treatment in MC38 murine models (Figure 5b). As expected, we found that SLAMF8‐positive cells were altered in a time‐dependent manner in MC38 murine models (Figure 6c–h), with a significant increase in SLAMF8 expression within the tumors from the beginning to the end of the anti‐PD1 treatment (Figure 6j, *P* = 0.004). Collectively, these results further demonstrated that SLAMF8 was a potential biomarker predicting the efficacy of anti‐PD1mAb therapy.

#### The role of *SLAMF8* in antitumor immunotherapy

**Figure 7:**
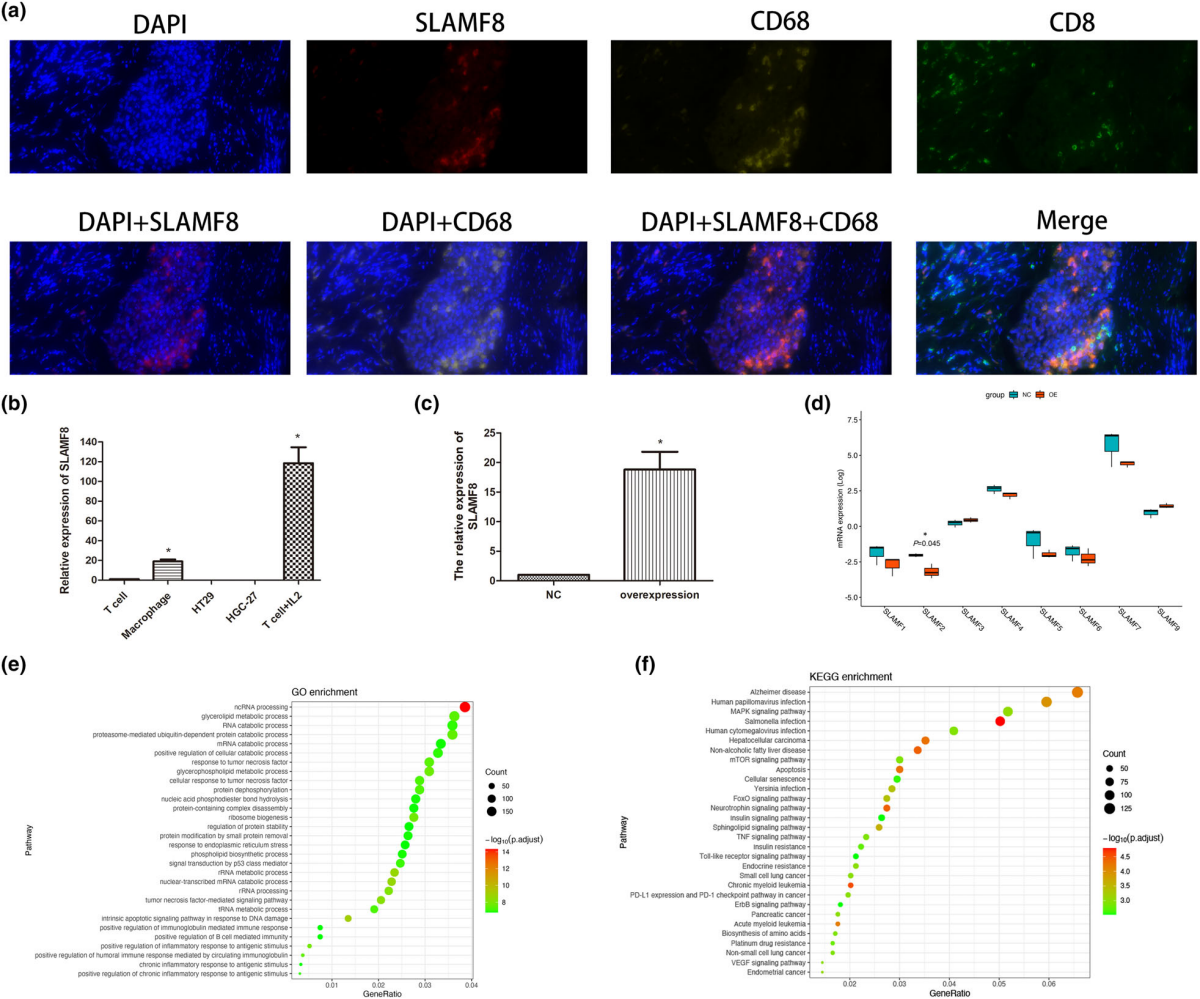
Location, expression and potential function of *SLAMF8* in the antitumor immune response. Representations of multiplex immunofluorescence of *SLAMF8* (red), CD68 (yellow), CD8 (green), and merged images in gastric cancer tissue **(a)**; quantitative RT–PCR was used to analyse the expression levels of *SLAMF8* in common T cells, T cells stimulated by IL‐2, HT29 cells, HGC27 cells and macrophages **(b)**; the mRNA expression of *SLAMF8* in TPH1‐induced macrophages with exogenous overexpression of *SLAMF8* and the negative control **(c)**; the impact of *SLAMF8* overexpression in macrophages on other SLAMF family genes expression by RNA sequencing and related comparison **(d)**; GO **(e)** and KEGG **(f)** pathway analysis to explore the pathways enriched by exogenous overexpression in macrophages. Nonstatistical images are from one experiment that is representative of three separate experiments.

To further understand the detailed mechanism of SLAMF8 in antitumor immunotherapy, we first detected the location of the SLAMF8 protein by multiplex immunofluorescence assay. We identified that the SLAMF8 protein was abundantly present on the surface of CD68^+^ macrophages (Figure 7a). In addition, *in vitro* experiments followed by quantitative real‐time polymerase chain reaction (qRT–PCR) assays showed that *SLAMF8* mRNA was mainly expressed on human macrophages and T cells activated by IL‐2 but not on tumor cells (Figure 7b). Then, we further examined the role of SLAMF8 by assessing the effect of its exogenous overexpression in human macrophages. Compared with the negative control, *SLAMF8* overexpression was validated by qRT–PCR (Figure 7c). *SLAMF8* overexpression in macrophages downregulated *SLAMF2* gene expression (*P* = 0.045) but had no significant impact on other SLAMF family genes (Figure 7d). GO and KEGG pathway analysis using RNA‐sequencing analysis showed the pathways enriched in *SLAMF8* high‐expression macrophages, including positive regulation of B‐cell‐mediated immunity, TNF signing pathway, PDL1 expression and PD1 checkpoint pathway in cancer, and positive regulation of multiple immune response (Figure 7e, f). These results indicated that *SLAMF8* was involved in activating the antitumor immune response.

## DISCUSSION

Although anti‐PD1 immunotherapy has provided a novel option for cancer treatment and resulted in prolonged survival and improved quality of life,^34^ only a very small subset of patients can benefit from the treatment strategy. Previous studies used limited biomarkers to predict the clinical efficacy of anti‐PD1mAb therapy without successful validation across studies, as suggested.^35^, ^36^ EBV infection was previously reported to be associated with the efficacy of anti‐PD1 treatment. However, EBV infection was only observed in a small proportion of patients with specific types of cancer, GC and nasopharyngeal cancer in particular.^37^ Thus, an investigation of the EBV‐related signature may provide clues for identifying biomarkers reflecting the antitumor immune response. In the present study, we identified the CCGs that were coexpressed in both EBV^+^ and EBV^−^ tumors using the EBV‐specific signature. We further selected *SLAMF8* as the tag gene of the CCGs. Thus, the CCGs tagged by *SLAMF8* were the potential target genes indeed responsible for the antitumor immune response, independent of the EBV infection status. We then found that the CCGs tagged by *SLAMF8* were successfully validated in independent datasets of GI cancer samples.

High expression of *SLAMF8* was reportedly correlated with an enhanced T‐cell‐mediated immune phenotype and activated IFN‐γ signalling pathway in glioma.^23^ In accordance with previous studies, our GSEA also suggested that high expression of *SLAMF8* was associated with an activated antitumor immune response, including positive regulation of IFN‐γ production and T‐cell activation in GI cancers. Tumors with a high density of CD8^+^ T cells and a high IFN‐γ signature score were previously reported to have a good response to anti‐PD1 therapy.^38^, ^39^, ^40^ Intriguingly, our results also showed that the increased expression of *SLAMF8* was associated with higher CD8 expression at both the mRNA and protein levels. In addition, our data suggested that *SLAMF8* expression was significantly upregulated when T cells were activated with IL‐2, and its overexpression was associated with activated immune pathways. These results indicated that high *SLAMF8* expression correlated with the ‘hot’ immune microenvironment in GI cancer. Based on these findings, we further observed that higher pretherapeutic *SLAMF8* expression was associated with a better response to anti‐PD1 treatment in clinical patients, thus supporting the potential role of *SLAMF8* in predicting anti‐PD1 treatment efficacy.

In murine models, we observed that pretherapeutic *SLAMF8* expression was higher in tumors relatively sensitive to anti‐PD1 treatment than in those resistant to anti‐PD1 treatment. Moreover, *SLAMF8* expression was increased in a time‐dependent manner from the beginning to the end of anti‐PD1 treatment for the relatively sensitive tumors. It is widely recognised that anti‐PD1 blockade may attenuate the suppression of the immune microenvironment and promote the activation of TILs. Our results indicated that high expression of *SLAMF8* was correlated with the activated T‐cell phenotype. This evidence provides a potential explanation for the dynamic increase in *SLAMF8* expression during anti‐PD1 treatment.

In summary, our results suggest that higher expression of *SLAMF8* is an indicator for better efficacy of anti‐PD1 immunotherapy in GI cancers. However, further investigations are required to characterise the mechanism of *SLAMF8* in more detail and validate the predictive role of *SLAMF8* with a large sample size.

## METHODS

### Identification of the EBV‐related immune signature, the CCGs and its tag genes

The GSE51575 dataset derived from the GEO database was based on a study that focused on mRNA expression profiling in EBV^+^ and EBV^−^ GC tissues as well as adjacent normal tissues.^41^ We used differential gene expression analysis to identify a molecular signature specific for the EBV‐induced antitumor response in three steps. First, we captured the DEGs between GC and normal tissues with EBV infection. Second, DEGs for those without EBV infection were also explored. Third, we identified the gene sets included in DEGs from EBV^+^ GC tissues but not in those from EBV^−^ GC tissues as an EBV‐specific immune signature. The Limma statistical package in R (version 3.3.6) was used to perform differential gene expression analysis.

Using the EBV‐specific immune signature, we further identified the CCGs between EBV^+^ and EBV^−^ GC tissues by network coexpression analysis using the WGCNA statistical package in R software and selected the top 30 CCGs. The tag gene of this signature was finally selected by principal component analysis.

### GSEA

Using GSEA software (v4.0.3, UC San Diego and Broad Institute, USA), we compared the enrichment results of tag gene expression in GI cancers (high expression vs. low expression) and EBV status in GC tissues (positive vs. negative).

### Clinical tumor samples and tissue microarray

We obtained 20 GC and 20 CRC tissues from the tissue bank of Nanjing Drum Tower Hospital for RNA‐sequencing analysis. In addition, we collected 20 EBV^+^ and 20 EBV^−^ GC tissues from Nanjing Drum Tower Hospital. The CRC tissue microarray including 36 paired tumor and normal tissues was obtained from Fudan University Shanghai Cancer Center. Another GC tissue microarray (HStmA150CS02) that contained 74 paired available tumor and normal tissues was obtained from Shanghai Outdo Biotech Company (Shanghai, China).

For clinical validation, we retrospectively collected tumor tissues from 13 patients with GI cancer and an additional 1 patient with melanoma and 1 patient with chest wall sarcoma between September 2018 and March 2021, of whom 6 samples were from patients in a clinical trial (ID: 2016L01455), and the other 9 tissues were from patients who received pembrolizumab in our cancer centre.

### Cell culture and Construction of plasmids and transfection

The human mononuclear cell line THP‐1 was purchased from the Cell Bank of the Chinese Academy of Sciences (Shanghai, China) and maintained in 1640, 10% FBS and 1% penicillin/streptomycin in a humidified atmosphere at 37°C with 5% CO2. Then, THP‐1 cells were induced to differentiate into macrophages with 10 ng ml^−1^ phorbol 12‐myristate 13‐acetate. A pCMV‐SLAMF8 plasmid was obtained from Shanghai GenePharma Company (Shanghai, China) and transfected into macrophages by Lipofectamine 3000 (Thermo Fisher Scientific, Massachusetts, USA) according to the manufacturer's instructions.

### qRT–PCR and RNA‐sequencing analysis

For qRT–PCR, total RNA was extracted and dissolved in reverse‐transcribed RNA into cDNA with a Transcriptor First Strand cDNA Synthesis Kit (Roche, Basel, Switzerland) according to the instructions. qRT–PCR was performed using an ABI 7900 System (Thermo Fisher Scientific, Massachusetts, USA). Primers were synthesised by Tsingke Biological Technology (Beijing, China), and the sequences of primers were as follows: *GAPDH:* forward 5′‐GGAGCGAGATCCCTCCAAAAT‐3′, reverse: 5′‐GGCTGTTGTCATACTTCTCATGG‐3′, *SLAMF8:* forward 5′‐CTGATGGTGGATACAAGGG‐3′, reverse *SLAMF8:* 5′‐GGAAATGGACGTAACGGA‐3′. *GAPDH* was used as the endogenous control.

A total of 19 GC and 20 CRC tissues were successfully used for RNA‐sequencing analysis. RNA isolation and construction of the RNA‐sequencing library and sequencing were performed by the Shanghai Sangon Biological Engineering Technology Company (Shanghai, China) following the manufacturer’s protocols. We validated the performance of the CCGs and the tag gene using the RNA‐sequencing data by dimension reduction analysis and correlation analysis. Macrophages transfected with a pCMV‐SLAMF8 plasmid or negative control were used for total RNA isolation and RNA sequencing by the Shanghai Sangon Biological Engineering Technology Company (Shanghai, China).

### IHC and multiplex immunofluorescence

We performed IHC analysis with rabbit monoclonal anti‐human CD8 antibody (1:200, ab101500, Abcam, Cambridgeshire, England), rabbit polyclonal anti‐human SLAMF8 antibody (1:200, ab221703, Abcam, Cambridgeshire, England) and rabbit monoclonal anti‐mouse CD8 antibody (1:200, ab209775, Abcam, Cambridgeshire, England) according to the manufacturer’s instructions. CD8 and SLAMF8 expression was quantitatively analysed according to the methods by Mahmoud *et al*.^42^ and Zhu *et al*., respectively.^43^

Multiplex immunofluorescence was used to characterise the location of SLAMF8 protein with rabbit polyclonal anti‐human SLAMF8 antibody (1:200, ab221703, Abcam, Cambridgeshire, England), rabbit monoclonal anti‐human CD8 antibody (1:200, ab101500, Abcam, Cambridgeshire, England) and rabbit monoclonal anti‐human CD68 antibody (1:500, M0876, DAKO, Glostrup, Denmark).

### ISH assay

EBV‐encoded RNA (EBER) was detected via chromogenic in situ hybridisation using fluorescein‐labelled oligonucleotide probes (INFORM EBER Probe, Ventana, Cambridgeshire, England). EBER positivity was defined when >20% of the tumor cells were stained for EBER. The RNA scope 2.5 An HD reagent kit was used for the ISH assay (ACD, California, USA) according to the manufacturer’s instructions, with the murine *SLAMF8* probe (catalogue #571981, ACD, California, USA) used as a target probe for hybridisation and the other two probes used as positive (catalogue #313911, ACD, California, USA) and negative control probes (catalogue #310043, ACD, California, USA). The average *SLAMF8* density was calculated by FiJi ImageJ software using the manufacturer’s instructions.

### Murine tumor model establishment

Murine CT26 CRC and MFC GC cell lines were purchased from the cell bank of the Chinese Academy of Sciences (Shanghai, China). The murine MC38 CRC cell line was purchased from the National Infrastructure of Cell Line Resource (Beijing, China). All cell lines were cultured according to the manufacturer’s instructions.

All animal experiments were performed according to the guidelines of the National Institutes of Health. Five‐week‐old BALB/c, C57BL/6 and 615 mice were purchased from the Experimental Animal Center of Nanjing Drum Tower Hospital. A total of 1ⅹ10^6^ CT26 cells and MC38 cells were inoculated subcutaneously into BALB/c and C57BL/6 mice, respectively. A total of 1.5 × 10^6^ MFC cells were inoculated subcutaneously into 615 mice. After 2 weeks, tumor models were established and divided into two groups that received 200 µg anti‐PD1 mAb (clone RMP1–14, catalogue #BE0146, BioXCell, New Hampshire, USA) and control Ig (clone 2A3, catalogue #BE0089, BioXCell, New Hampshire, USA) by intraperitoneal injection (i.p.) on days 15, 18 and 21. Tumor volume was measured every 3 days as 1/2ⅹDⅹd^2^, in which D and d were the longest and shortest diameter of the tumor, respectively. After 30 days, the mice were sacrificed for ISH analysis, and tumor weight was measured. We assessed the expression of the tag gene dynamically in tumor models sensitive to anti‐PD1mAb. The mice were sacrificed at 15 (before initial treatment) and 18, 21, 24, 27 and 30 days for ISH analysis.

### Statistics analysis

Student’s *t*‐tests were used to compare the differences between two groups. One‐way ANOVA was used to determine the differences among multiple groups. Pearson’s correlation coefficient was used to evaluate the correlation matrices. All continuous data are presented as the mean ± standard deviation. A two‐tailed *P* < 0.05 was considered to be statistically significant.

## Conflict of interest

The authors declare that they have no conflicts of interest.

## Author contribution

**Qun Zhang:** Data curation; Investigation; Methodology; Writing‐original draft; Writing‐review & editing. **Lei Cheng:** Investigation; Methodology; Software; Validation; Writing‐review & editing. **Yanmei Qin:** Methodology; Resources; Software; Writing‐review & editing. **Linghui Kong:** Methodology; Software; Writing‐review & editing. **Xiao Shi:** Resources; Supervision; Writing‐review & editing. **Jing Hu:** Resources; Writing‐review & editing. **Li Li:** Supervision; Writing‐review & editing. **Zhou Ding:** Writing‐review & editing. **Ting Wang:** Methodology; Resources. **Jie Shen:** Data curation; Formal analysis; Resources. **Yang Yang:** Data curation; Writing‐review & editing. **Lixia Yu:** Methodology; Resources. **Baorui Liu:** Project administration; Resources. **Chenchen Liu:** Conceptualization; Project administration; Resources; Writing‐review & editing. **Xiaoping Qian:** Conceptualization; Data curation; Funding acquisition; Project administration; Resources; Supervision; Writing‐review & editing.

## Ethical approval

This study was conducted with the approval of the Ethics Committee of Nanjing Drum Tower Hospital (ID: YBK‐2018‐001‐01) and Fudan University Shanghai Cancer Center (ID: 050432‐4‐1911D) and the Ethics Committee of Shanghai Outdo Biotech Company (ID: T18‐1254).

## Supporting information

Supplementary Material
