# Supplementary material for: SLAMF8 expression predicts the efficacy of anti‐PD1 immunotherapy in gastrointestinal cancers
Source: Clin Transl Immunology. 2021 Oct 26;10(10):e1347. doi: 10.1002/cti2.1347 (PMC8546794; doi:10.1002/cti2.1347)
Supplement: Supplementary file 1 — Supplementary Material [file CTI2-10-e1347-s001.pdf]

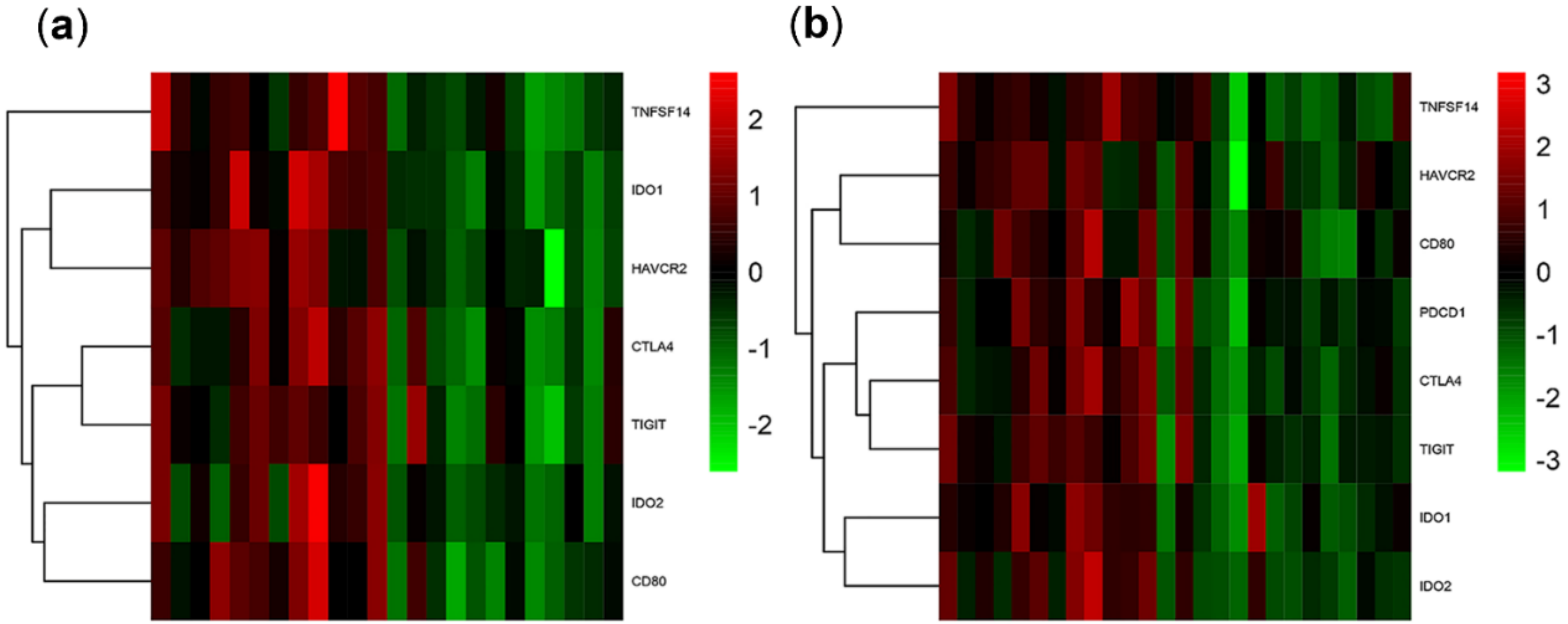


**Supplementary figure 1: Differential expression analysis for 7 immune checkpoint gene, along with *PDCD1*, contained in 788-gene signature specific for EBV infection in GC.**

The differential expression of 7 immune checkpoint genes included in EBV related gene signature. Analysis were performed between EBV+ GC tissues and adjacent normal tissues **(a)**;

The differential expression of *PDCD1*, in addition to the 7 immune checkpoint genes, between $EBV^{+}$ and $EBV^{-}$ GC tissues **(b)**.

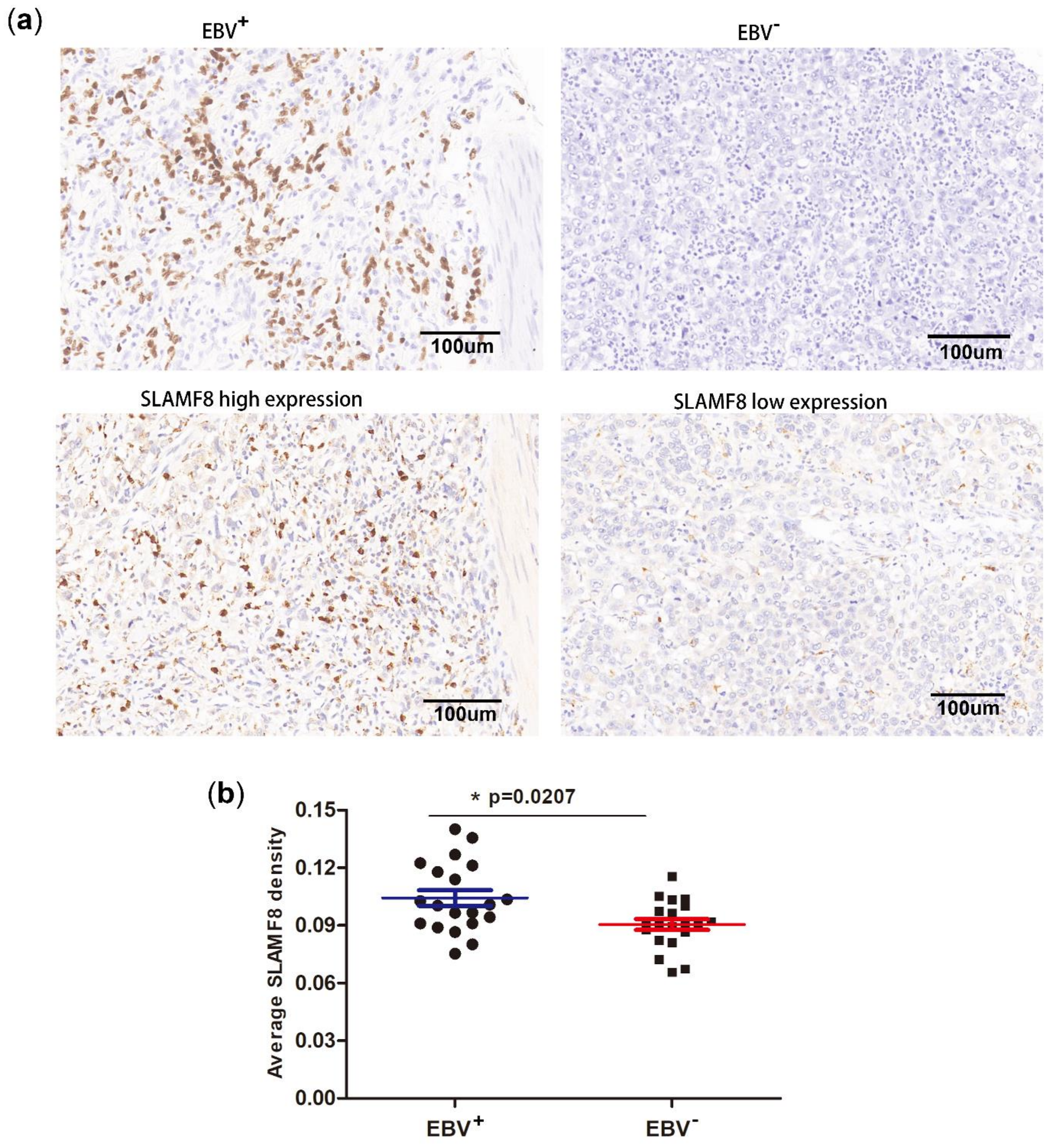


**Supplementary figure2: The expression of SLAMF8 in EBV positive and negative GC tissues.**

Representative image of IHC staining of *SLAMF8* in EBER positive (n=20) and negative GC tissues (n=20) with scale bar of 100μm **(a)**; Comparison of SLAMF8 expression between EBER positive and negative GC tissues by IHC staining **(b)**. Data are from one experiment that is representative of three separate experiments.

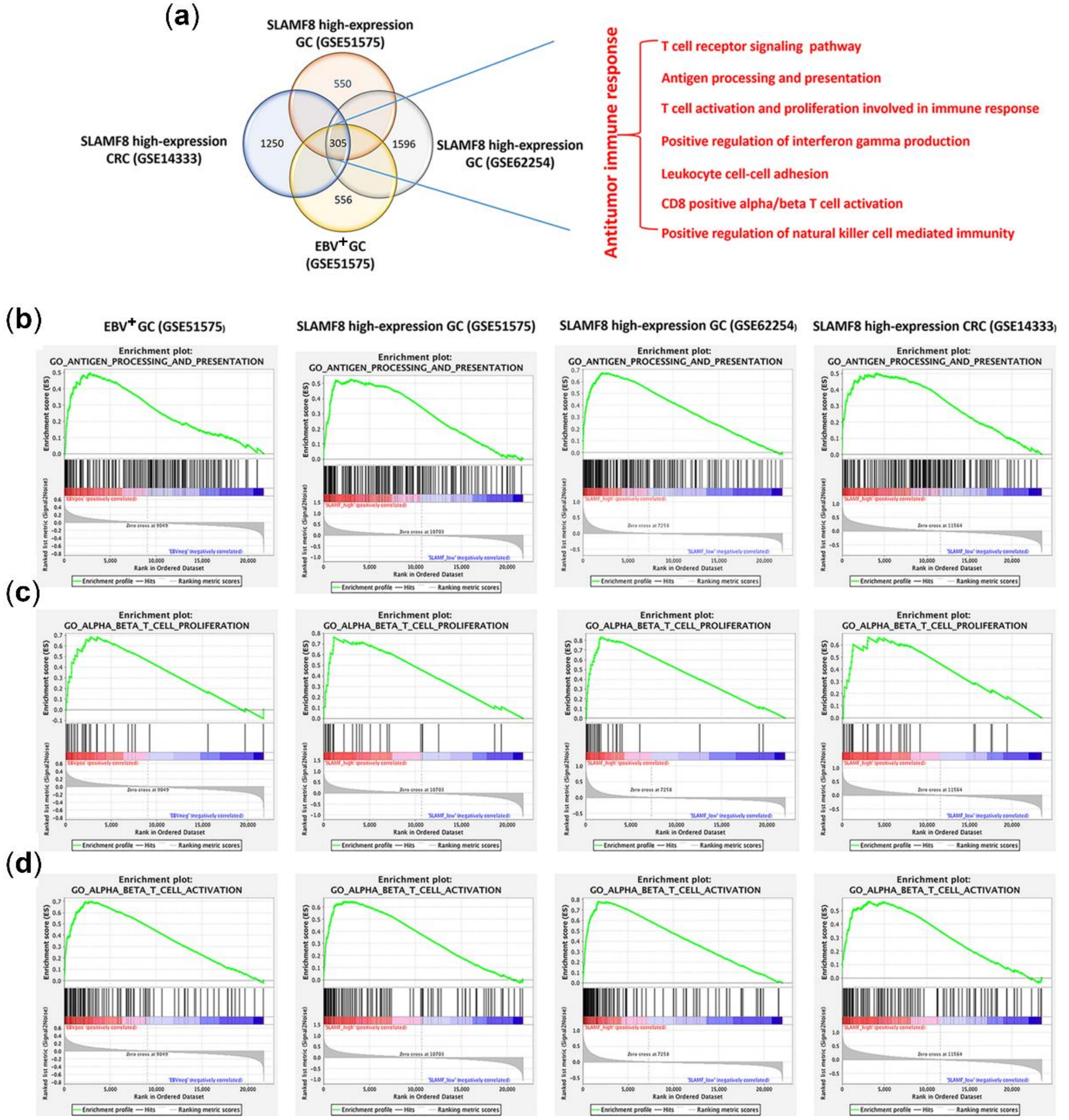


**Supplementary figure 3: High-expression *SLAMF8* GI cancers may enrich the pathways involved in antitumor immune response.**

Using transcriptome data from GEO database for GSEA analysis, we identified number of pathways enriched in *SLAMF8* high-expression GI cancer tissues (GSE62254 and GSE51575 for GC and GSE14333 for CRC) and EBV$^{+}$ GC (GSE51575), and found that the enriched pathways contained pathways including **(a)**, antigen presentation **(b)**, T cells proliferation **(c)** and activation **(d)** pathways.

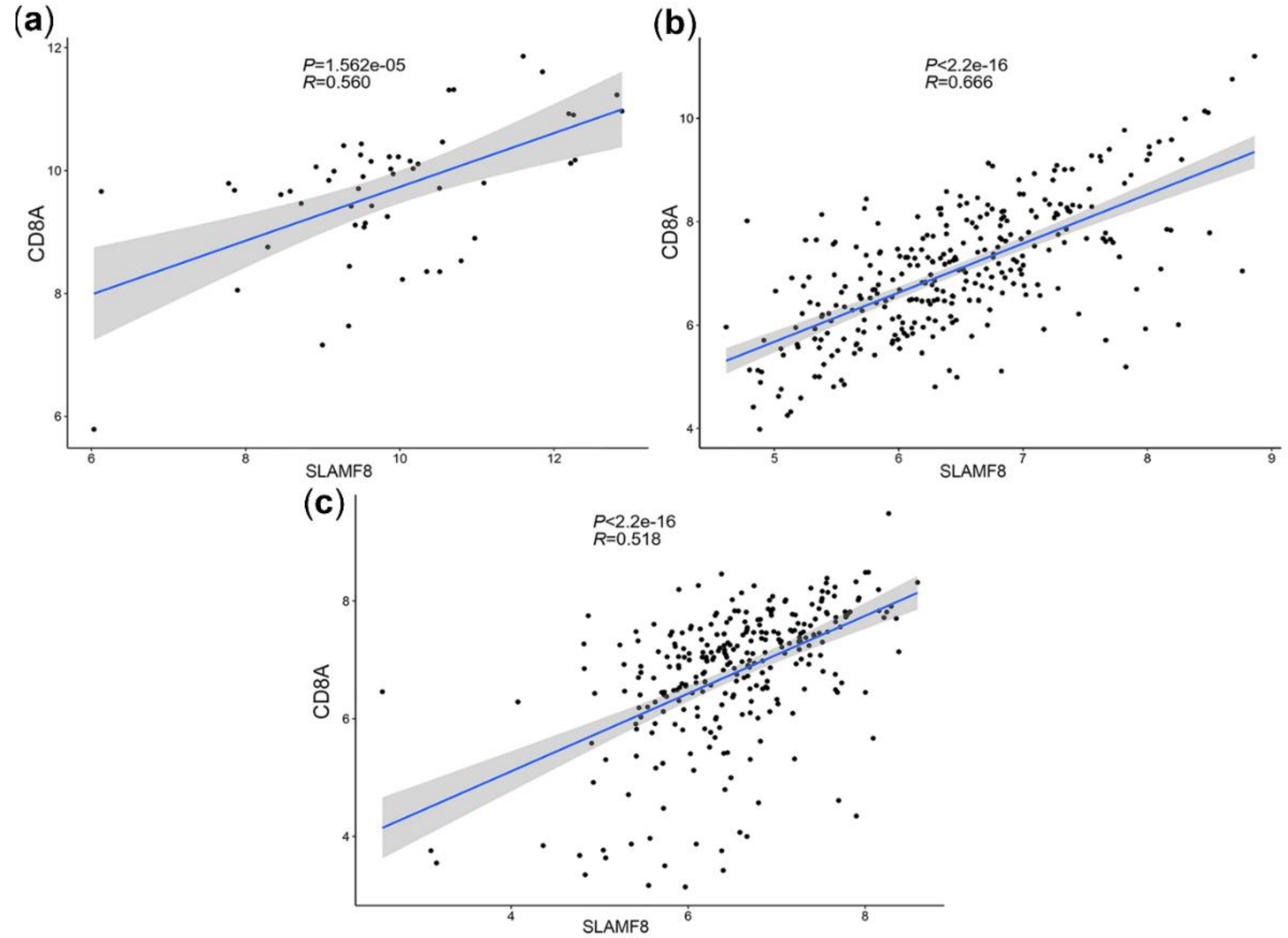


**Supplementary figure 4: Correlation of *SLAMF8* with *CD8* mRNA expression in public GEO database.**

The correlation of *SLAMF8* and *CD8A* mRNA expression in GSE51575 **(a)** and GSE62254 **(b)** datasets of GC tissues, and GSE14333 **(c)** dataset of CRC tissues.

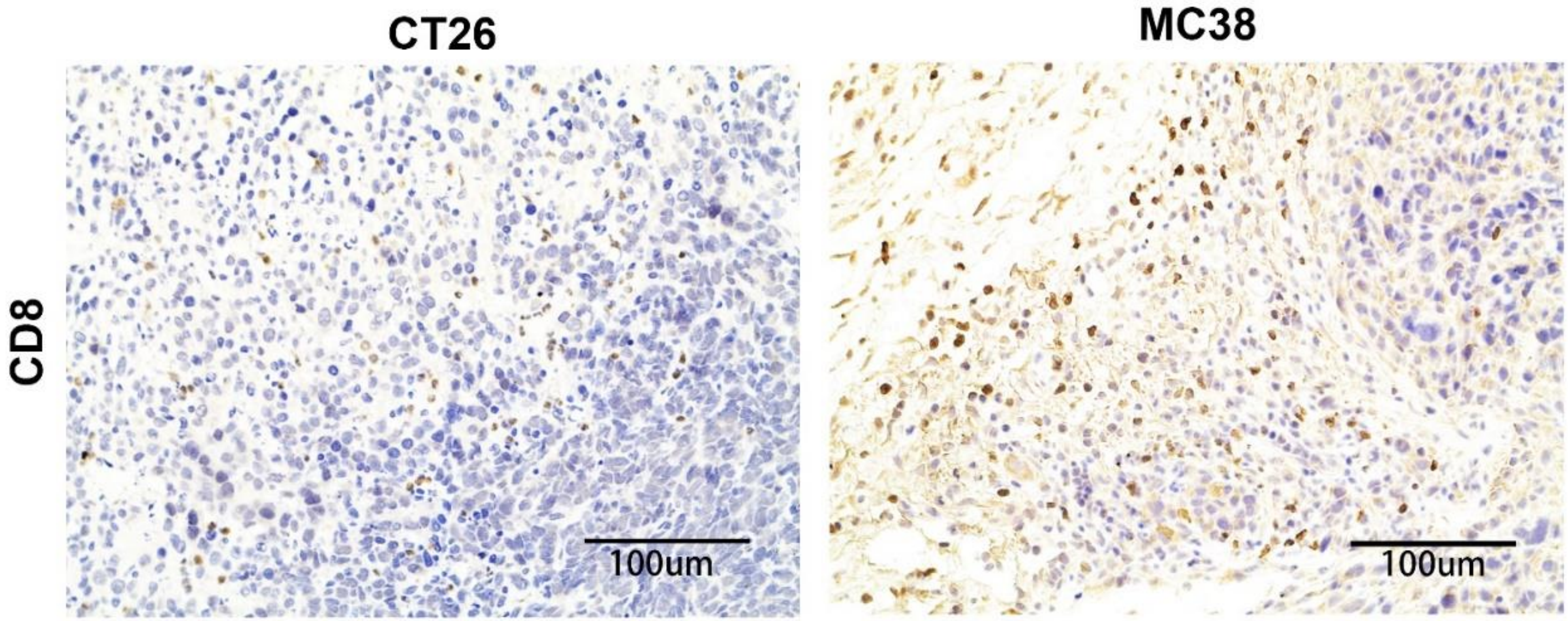


**Supplementary figure 5: The amount of CD8$^+$ T cells in CT26 and MC38 tumors**

Representative image of IHC staining of pre-therapeutic CD8 in murine tumor tissues established by CT26 CRC cell line **(a)** and MC38 CRC cell line **(b)** with scale bar of 100μm. Data are from one experiment that is representative of three separate experiments.

**Supplementary table 1: a 788-gene signature that was potentially involved in immune response against gastric cancer induced by EBV infection.**

| gensymbolpos | logFC | AveExpr | t | P.Value | adj.P.Val | B |
|---|---|---|---|---|---|---|
| RGN | -4.4576197 | 7.563354124 | -13.13731616 | 7.59E-13 | 4.13E-09 | 19.15074968 |
| CDKN1C | -2.8944618 | 10.76131169 | -11.32259017 | 1.96E-11 | 4.26E-08 | 16.11682916 |
| A_33_P3220911 | 2.38518576 | 13.74079917 | 11.04199058 | 3.35E-11 | 6.61E-08 | 15.61153805 |
| TPD52L1 | -3.9732307 | 7.707187139 | -10.86960052 | 4.67E-11 | 6.78E-08 | 15.29603086 |
| BCL2L10 | -1.8818199 | 5.623533936 | -9.786057711 | 4.11E-10 | 2.79E-07 | 13.22157472 |
| BST2 | 2.38379055 | 13.11549176 | 9.637632839 | 5.60E-10 | 3.51E-07 | 12.92481248 |
| NCRNA00087 | -1.5281076 | 8.031775813 | -9.633414449 | 5.65E-10 | 3.51E-07 | 12.91633293 |
| IL8 | 3.40766488 | 7.450233651 | 9.112545918 | 1.71E-09 | 7.64E-07 | 11.84993327 |
| C9orf24 | -2.6045356 | 7.865929234 | -8.950341511 | 2.43E-09 | 9.76E-07 | 11.50994034 |
| LOC100291656 | -2.0532025 | 6.582419881 | -8.943554202 | 2.47E-09 | 9.76E-07 | 11.49563138 |
| VLDLR | -1.5660941 | 6.302322625 | -8.906943683 | 2.68E-09 | 1.04E-06 | 11.41833524 |
| MTSS1 | -1.8258071 | 7.422844849 | -8.791065447 | 3.45E-09 | 1.21E-06 | 11.17241138 |
| FNDC5 | -3.3690757 | 5.828017788 | -8.676917987 | 4.44E-09 | 1.46E-06 | 10.92827367 |
| CTF1 | -2.006424 | 7.79793681 | -8.647199039 | 4.75E-09 | 1.50E-06 | 10.86440364 |
| HSPH1 | 1.27111603 | 12.19599744 | 8.622486837 | 5.02E-09 | 1.53E-06 | 10.81119708 |
| IFI30 | 1.82759405 | 15.17934993 | 8.595813428 | 5.32E-09 | 1.57E-06 | 10.75366942 |
| PCOLCE2 | -4.3542485 | 6.922878879 | -8.586070111 | 5.44E-09 | 1.57E-06 | 10.73263009 |
| HAPLN3 | 2.37367449 | 11.89908022 | 8.576929113 | 5.55E-09 | 1.57E-06 | 10.71287898 |
| OBSL1 | -2.1155925 | 7.693814206 | -8.266455221 | 1.12E-08 | 2.61E-06 | 10.0349089 |
| CXCL10 | 4.17915087 | 9.645611988 | 8.227314056 | 1.22E-08 | 2.80E-06 | 9.948457372 |
| AP3B2 | -1.1148596 | 4.570701783 | -8.208773066 | 1.27E-08 | 2.86E-06 | 9.907429273 |
| SNTA1 | -2.0142653 | 6.814500767 | -8.156355901 | 1.44E-08 | 3.15E-06 | 9.791173456 |
| PDE9A | -2.1057649 | 6.843922283 | -8.104038426 | 1.62E-08 | 3.31E-06 | 9.674748257 |
| LOC388588 | -2.4431597 | 8.420743928 | -7.978701121 | 2.16E-08 | 4.27E-06 | 9.394245631 |
| KCNJ12 | -2.9943633 | 6.349758364 | -7.973328713 | 2.19E-08 | 4.29E-06 | 9.38217252 |
| CXCL9 | 4.01492332 | 12.3927015 | 7.964446937 | 2.23E-08 | 4.34E-06 | 9.362204053 |
| PHGDH | -3.0888271 | 8.761732192 | -7.934920833 | 2.39E-08 | 4.56E-06 | 9.295741832 |
| SLC4A3 | -1.5622434 | 5.699902291 | -7.896099768 | 2.62E-08 | 4.95E-06 | 9.208169799 |
| CDH19 | -1.9054224 | 5.532525701 | -7.823627136 | 3.10E-08 | 5.57E-06 | 9.044119553 |
| DNASE1 | -2.6311296 | 8.674247306 | -7.733878808 | 3.82E-08 | 6.70E-06 | 8.839943816 |
| IL18BP | 2.06620303 | 7.963582989 | 7.629415276 | 4.89E-08 | 7.86E-06 | 8.600879013 |
| C12orf34 | -2.4817055 | 8.555294619 | -7.627109465 | 4.91E-08 | 7.86E-06 | 8.595585117 |
| IL8RBP | 2.91388163 | 7.195426961 | 7.626479486 | 4.92E-08 | 7.86E-06 | 8.594138624 |

| | | | | | | |
|---|---|---|---|---|---|---|
| DIRAS3 | -1.6077998 | 5.558640555 | -7.579074619 | 5.50E-08 | 8.36E-06 | 8.4851355 |
| CRABP1 | -1.9809284 | 5.816155387 | -7.572782499 | 5.59E-08 | 8.43E-06 | 8.470644098 |
| RIMS4 | -2.2096678 | 6.361689834 | -7.476007435 | 7.03E-08 | 9.85E-06 | 8.247077972 |
| CCNJL | -2.5632526 | 6.307042627 | -7.471168119 | 7.11E-08 | 9.90E-06 | 8.235864809 |
| ARHGEF4 | -1.813844 | 6.315995383 | -7.452962932 | 7.42E-08 | 1.01E-05 | 8.19365312 |
| FCGR1B | 3.01478808 | 9.676182174 | 7.448040195 | 7.51E-08 | 1.01E-05 | 8.182231223 |
| CBLN1 | -1.2134403 | 4.109254599 | -7.437687672 | 7.70E-08 | 1.02E-05 | 8.158200238 |
| ATCAY | -1.1138755 | 4.470290925 | -7.434226034 | 7.76E-08 | 1.02E-05 | 8.150161606 |
| ZNF287 | -1.1966095 | 5.109613709 | -7.433388925 | 7.78E-08 | 1.02E-05 | 8.148217424 |
| MGC5566 | 2.55526979 | 6.757831731 | 7.393610007 | 8.55E-08 | 1.09E-05 | 8.055721844 |
| TCP11L2 | -1.0947212 | 7.233926203 | -7.389227052 | 8.64E-08 | 1.09E-05 | 8.045517356 |
| DCBLD2 | -1.4917675 | 7.067905134 | -7.369475966 | 9.06E-08 | 1.13E-05 | 7.999500368 |
| AMH | 1.91326715 | 6.642828299 | 7.368251295 | 9.09E-08 | 1.13E-05 | 7.996645347 |
| GFRA2 | -1.8558171 | 8.21863652 | -7.316158507 | 1.03E-07 | 1.26E-05 | 7.875017512 |
| PLA2G7 | 3.03964204 | 9.814353322 | 7.275345442 | 1.14E-07 | 1.34E-05 | 7.779472752 |
| CRNKL1 | 1.03197928 | 7.880234335 | 7.268665996 | 1.15E-07 | 1.34E-05 | 7.763814859 |
| SOBP | -1.556744 | 6.227449392 | -7.237967682 | 1.24E-07 | 1.43E-05 | 7.691776161 |
| ATF5 | 1.10592341 | 9.08986423 | 7.12283141 | 1.64E-07 | 1.76E-05 | 7.420485922 |
| STAT1 | 1.81689059 | 11.74465476 | 7.106358766 | 1.71E-07 | 1.81E-05 | 7.381530859 |
| UBE2L6 | 1.31209342 | 11.55517015 | 7.106113865 | 1.71E-07 | 1.81E-05 | 7.380951446 |
| C2CD4B | -3.2886148 | 9.644457219 | -7.082046765 | 1.81E-07 | 1.89E-05 | 7.323972927 |
| WNT6 | -2.1125903 | 7.671896472 | -7.063687779 | 1.89E-07 | 1.94E-05 | 7.280458106 |
| GBP5 | 2.97574941 | 10.89151139 | 6.981348226 | 2.31E-07 | 2.31E-05 | 7.084765098 |
| APOC1 | 3.61057954 | 11.88019256 | 6.908049839 | 2.77E-07 | 2.59E-05 | 6.909838275 |
| PARP14 | 1.28388243 | 11.44323574 | 6.901240115 | 2.81E-07 | 2.61E-05 | 6.893552712 |
| UBD | 2.90945003 | 15.26456805 | 6.897534383 | 2.84E-07 | 2.63E-05 | 6.884687973 |
| RASSF10 | -1.6386975 | 6.234710453 | -6.873896855 | 3.01E-07 | 2.77E-05 | 6.828102881 |
| HEJ1 | 1.06999752 | 12.27365854 | 6.87082399 | 3.03E-07 | 2.78E-05 | 6.82074177 |
| PID1 | -2.8779971 | 8.356511667 | -6.859942316 | 3.11E-07 | 2.84E-05 | 6.794665118 |
| MORN5 | -1.1727607 | 4.544840595 | -6.84250085 | 3.25E-07 | 2.91E-05 | 6.752838264 |
| AMBP | -2.3638469 | 6.362039638 | -6.816751837 | 3.46E-07 | 3.01E-05 | 6.691020619 |
| TMEM25 | -2.029771 | 7.484021348 | -6.811030563 | 3.51E-07 | 3.04E-05 | 6.677274111 |
| C10orf81 | 2.07416464 | 8.063152111 | 6.774927277 | 3.84E-07 | 3.25E-05 | 6.590436903 |
| MST1 | -2.2037316 | 11.67485696 | -6.76381628 | 3.94E-07 | 3.30E-05 | 6.563680485 |
| C13orf35 | 1.05553981 | 4.510060816 | 6.76087215 | 3.97E-07 | 3.31E-05 | 6.556588223 |

| | | | | | | |
|---|---|---|---|---|---|---|
| PC | -1.1842014 | 9.896038853 | -6.747578698 | 4.11E-07 | 3.39E-05 | 6.524551978 |
| ULK2 | -1.4373722 | 7.561919004 | -6.731360034 | 4.27E-07 | 3.51E-05 | 6.485437473 |
| CA4 | -1.9635075 | 5.928497268 | -6.711121518 | 4.49E-07 | 3.64E-05 | 6.436584222 |
| EFR3B | -1.1531167 | 6.323253589 | -6.673125141 | 4.94E-07 | 3.93E-05 | 6.344734386 |
| LTB4R | 1.1441526 | 6.669651244 | 6.664541863 | 5.04E-07 | 3.97E-05 | 6.323962171 |
| tcag7.873 | 1.4256654 | 12.229897 | 6.660294565 | 5.10E-07 | 3.99E-05 | 6.313680172 |
| C19orf28 | 1.41264877 | 8.943596765 | 6.656038405 | 5.15E-07 | 4.00E-05 | 6.303374599 |
| RFX6 | -2.6475982 | 5.122838115 | -6.648966353 | 5.24E-07 | 4.04E-05 | 6.286246127 |
| CYS1 | -2.3230461 | 6.718308581 | -6.634933422 | 5.43E-07 | 4.17E-05 | 6.252241147 |
| IDO1 | 4.09481482 | 11.58477047 | 6.619633717 | 5.64E-07 | 4.28E-05 | 6.215140365 |
| GBP4 | 1.89407642 | 12.40051347 | 6.603303244 | 5.87E-07 | 4.40E-05 | 6.175510109 |
| CCDC89 | -1.4078812 | 5.649784571 | -6.594616705 | 6.00E-07 | 4.48E-05 | 6.154417358 |
| BAIAP3 | -1.6595963 | 5.414232933 | -6.54523968 | 6.78E-07 | 4.90E-05 | 6.03435529 |
| THNSL2 | -2.4668583 | 7.730906011 | -6.530179836 | 7.04E-07 | 5.07E-05 | 5.997681552 |
| PART1 | -1.0950456 | 4.878038894 | -6.522209501 | 7.18E-07 | 5.14E-05 | 5.978261834 |
| SERPINE1 | 1.83714039 | 6.230151994 | 6.521896544 | 7.19E-07 | 5.14E-05 | 5.977499168 |
| TMEM61 | -3.1582993 | 7.926468974 | -6.509018252 | 7.42E-07 | 5.26E-05 | 5.946105672 |
| NTF3 | -1.6155514 | 6.654577876 | -6.508848923 | 7.42E-07 | 5.26E-05 | 5.945692774 |
| OSBPL3 | 1.29040989 | 9.993395384 | 6.496177919 | 7.66E-07 | 5.37E-05 | 5.914786222 |
| SLC7A2 | -2.9367945 | 8.15800252 | -6.438812077 | 8.84E-07 | 5.98E-05 | 5.774639179 |
| PPP3CA | -1.0631504 | 9.953191228 | -6.435109802 | 8.93E-07 | 6.00E-05 | 5.765581942 |
| HMGCS2 | -3.8969093 | 8.07225075 | -6.430901113 | 9.02E-07 | 6.01E-05 | 5.755283998 |
| TNFSF13B | 1.51536579 | 10.51460734 | 6.424857675 | 9.16E-07 | 6.09E-05 | 5.74049336 |
| CYP39A1 | -1.2679418 | 5.280499438 | -6.420747486 | 9.25E-07 | 6.13E-05 | 5.730431864 |
| ZNF480 | -1.7429813 | 7.596905305 | -6.394906585 | 9.87E-07 | 6.37E-05 | 5.667132988 |
| ENST00000287912 | 1.19164749 | 8.013786247 | 6.387265662 | 1.01E-06 | 6.41E-05 | 5.648402293 |
| EPB41L4B | -1.3014447 | 7.401388964 | -6.377731987 | 1.03E-06 | 6.53E-05 | 5.625022994 |
| CD300LF | 2.11643235 | 7.457585065 | 6.363636774 | 1.07E-06 | 6.69E-05 | 5.590439726 |
| LOC154092 | 1.53252882 | 4.526520664 | 6.350598139 | 1.10E-06 | 6.85E-05 | 5.558430026 |
| VAV3 | -1.7482693 | 9.892396988 | -6.350074198 | 1.10E-06 | 6.85E-05 | 5.55714338 |
| CREB3L4 | -1.5968751 | 10.31060108 | -6.349608773 | 1.11E-06 | 6.85E-05 | 5.556000409 |
| SCG2 | -2.138421 | 5.986791989 | -6.345196893 | 1.12E-06 | 6.91E-05 | 5.545164759 |
| GM2A | 1.43810396 | 11.23956646 | 6.284820178 | 1.30E-06 | 7.82E-05 | 5.39667389 |
| CLGN | -1.5433447 | 5.346180461 | -6.25781648 | 1.39E-06 | 8.28E-05 | 5.330138933 |
| KCNJ2 | 2.06165065 | 8.837316428 | 6.248984312 | 1.43E-06 | 8.36E-05 | 5.30836105 |

| | | | | | | |
|---|---|---|---|---|---|---|
| CAMK2N2 | 2.89860314 | 9.107650413 | 6.235312813 | 1.48E-06 | 8.55E-05 | 5.27463505 |
| CAPS | -1.9497291 | 8.306114162 | -6.233290738 | 1.48E-06 | 8.55E-05 | 5.269645226 |
| PLK1S1 | -1.2105154 | 9.680612417 | -6.231745269 | 1.49E-06 | 8.55E-05 | 5.265831233 |
| MANEAL | 1.23375256 | 8.776551506 | 6.231199388 | 1.49E-06 | 8.55E-05 | 5.264484023 |
| ZC4H2 | -1.9344373 | 9.19581362 | -6.22629913 | 1.51E-06 | 8.60E-05 | 5.252389046 |
| MAGI2 | -1.0387935 | 5.858292224 | -6.225545162 | 1.51E-06 | 8.60E-05 | 5.250527865 |
| DCDC2 | -2.18822 | 6.09309942 | -6.22120525 | 1.53E-06 | 8.61E-05 | 5.239813619 |
| CBS | -2.644146 | 10.46414939 | -6.207801622 | 1.58E-06 | 8.86E-05 | 5.20671127 |
| SMARCA1 | -2.1072416 | 8.559177121 | -6.191789853 | 1.65E-06 | 9.13E-05 | 5.167144337 |
| MSX2 | 1.76231521 | 5.728119302 | 6.18505342 | 1.67E-06 | 9.22E-05 | 5.150490268 |
| ZNF577 | -1.213652 | 7.052524276 | -6.178976651 | 1.70E-06 | 9.33E-05 | 5.135463222 |
| CTSB | 1.14947384 | 12.76695735 | 6.171963029 | 1.73E-06 | 9.41E-05 | 5.118114968 |
| CHIC1 | -1.0498058 | 5.842258594 | -6.166805588 | 1.75E-06 | 9.50E-05 | 5.105354928 |
| TBX1 | -1.4654553 | 6.805620639 | -6.165693639 | 1.76E-06 | 9.50E-05 | 5.102603513 |
| SALL2 | -1.9937904 | 7.525371663 | -6.164216793 | 1.77E-06 | 9.50E-05 | 5.098949009 |
| SYT12 | -1.5627566 | 5.973205675 | -6.142126288 | 1.87E-06 | 9.90E-05 | 5.044260083 |
| FAM172A | -1.2551647 | 10.07714306 | -6.12925105 | 1.93E-06 | 0.000101437 | 5.012363496 |
| CCL3 | 2.13280247 | 11.75467095 | 6.127642976 | 1.94E-06 | 0.000101437 | 5.008378603 |
| RAB6B | -1.8977594 | 5.279652011 | -6.114695931 | 2.00E-06 | 0.000103574 | 4.976286182 |
| CITED4 | -2.0020738 | 12.17944912 | -6.102156342 | 2.07E-06 | 0.000105415 | 4.945188594 |
| MOGAT1 | -2.027049 | 5.592707064 | -6.101233874 | 2.07E-06 | 0.000105415 | 4.942900333 |
| ENST00000369310 | 2.64047313 | 7.794359351 | 6.08968768 | 2.13E-06 | 0.000107793 | 4.914252229 |
| FAM189A1 | -1.8423789 | 5.731547204 | -6.084408814 | 2.16E-06 | 0.000108994 | 4.901150293 |
| IRX5 | -2.8575541 | 8.195524925 | -6.069035459 | 2.25E-06 | 0.000112034 | 4.862979466 |
| GK | 1.39571938 | 7.37693986 | 6.060618399 | 2.30E-06 | 0.000113762 | 4.842071304 |
| FAM50B | -1.8262951 | 8.551483736 | -6.056002784 | 2.32E-06 | 0.000114452 | 4.830603246 |
| APOC2 | 2.71490484 | 7.122226961 | 6.01776956 | 2.56E-06 | 0.000123273 | 4.735533496 |
| GBP1 | 2.11479899 | 10.06842536 | 6.013714933 | 2.59E-06 | 0.000123273 | 4.725443623 |
| LAMP3 | 2.31823819 | 10.03197185 | 6.007921552 | 2.63E-06 | 0.000124316 | 4.711024337 |
| SVOP | -1.9887917 | 5.066939586 | -6.005733182 | 2.64E-06 | 0.000124739 | 4.70557687 |
| MID2 | -1.6743116 | 7.138293344 | -6.001980982 | 2.67E-06 | 0.000125664 | 4.696235602 |
| OR8B3 | 1.45281653 | 5.347520378 | 6.000802858 | 2.67E-06 | 0.000125768 | 4.693302353 |
| ACADM | -1.1905949 | 9.567806292 | -5.962546754 | 2.95E-06 | 0.000136248 | 4.597987256 |
| C2 | 1.43590072 | 9.867141349 | 5.961829874 | 2.95E-06 | 0.000136248 | 4.596199929 |
| KYNU | 1.57580322 | 8.74158129 | 5.956415081 | 2.99E-06 | 0.00013785 | 4.582698319 |

| | | | | | | |
|---|---|---|---|---|---|---|
| HAVCR2 | 1.92688164 | 7.562414414 | 5.937266556 | 3.14E-06 | 0.000143729 | 4.534931862 |
| PCMTD1 | 1.50150424 | 5.008655271 | 5.936742172 | 3.15E-06 | 0.000143729 | 4.533623335 |
| KIAA1324L | -1.7451036 | 6.627989079 | -5.929750257 | 3.20E-06 | 0.000145383 | 4.516173715 |
| SLCO4A1 | 1.96758425 | 10.06669929 | 5.929230639 | 3.21E-06 | 0.000145383 | 4.514876745 |
| LOC100289258 | 1.00169655 | 15.77809177 | 5.928984622 | 3.21E-06 | 0.000145383 | 4.514262678 |
| GGT7 | -1.4165834 | 7.10323884 | -5.926833299 | 3.23E-06 | 0.00014588 | 4.50889268 |
| TMEM120A | -1.08667 | 10.90753837 | -5.922891226 | 3.26E-06 | 0.000146168 | 4.499051706 |
| BAIAP2 | -1.5518222 | 7.988334558 | -5.922727886 | 3.26E-06 | 0.000146168 | 4.498643916 |
| ENST00000382464 | 1.00870648 | 8.286133075 | 5.921205144 | 3.28E-06 | 0.000146168 | 4.494842178 |
| CD300A | 1.09201573 | 7.733943487 | 5.916912605 | 3.31E-06 | 0.000147028 | 4.484124201 |
| PDE4A | -1.0440669 | 8.066662262 | -5.916504561 | 3.31E-06 | 0.000147028 | 4.483105283 |
| SYT7 | -1.8309845 | 6.079480528 | -5.907230525 | 3.39E-06 | 0.000149687 | 4.459943485 |
| CXCL11 | 3.39509619 | 9.20911225 | 5.907101028 | 3.40E-06 | 0.000149687 | 4.459620018 |
| TAP1 | 1.72258392 | 14.84613873 | 5.897816544 | 3.48E-06 | 0.000151593 | 4.436424839 |
| NCKAP5 | -1.6933655 | 6.124207272 | -5.897417905 | 3.48E-06 | 0.000151593 | 4.435428768 |
| LRRN1 | -1.2931651 | 5.049171586 | -5.876611214 | 3.67E-06 | 0.00015829 | 4.383421401 |
| GALNTL1 | -2.7792565 | 7.643108542 | -5.872693962 | 3.71E-06 | 0.000159568 | 4.373626063 |
| RNF180 | -1.4323811 | 5.578383602 | -5.863179338 | 3.80E-06 | 0.000163177 | 4.349828949 |
| OR4X2 | 1.09884623 | 5.624172334 | 5.851498756 | 3.91E-06 | 0.000167139 | 4.320604534 |
| PRPH2 | -1.3288211 | 5.91029998 | -5.846246679 | 3.97E-06 | 0.000168739 | 4.30746045 |
| SGK2 | -2.1706629 | 9.213830663 | -5.839491982 | 4.04E-06 | 0.000170099 | 4.290552608 |
| C22orf36 | -2.5006612 | 8.441107786 | -5.83563821 | 4.08E-06 | 0.000171374 | 4.280904516 |
| F2RL2 | 2.59849719 | 8.00875218 | 5.824616178 | 4.19E-06 | 0.000174929 | 4.253303882 |
| SEMA6A | -1.6404922 | 5.509429366 | -5.810666192 | 4.35E-06 | 0.000179232 | 4.21835761 |
| LOC284242 | 1.26904101 | 6.284225411 | 5.801602155 | 4.45E-06 | 0.000182753 | 4.195643081 |
| GLA | 1.16797435 | 11.99502263 | 5.795370418 | 4.52E-06 | 0.000183959 | 4.180022639 |
| SLC25A21 | -1.4693207 | 5.37640358 | -5.792188255 | 4.56E-06 | 0.000184087 | 4.172045096 |
| CABC1 | -1.3679688 | 9.717253817 | -5.782037044 | 4.68E-06 | 0.000188594 | 4.146591293 |
| ADM | -1.7851936 | 11.08631042 | -5.758216805 | 4.97E-06 | 0.000196476 | 4.086832344 |
| NACAD | -1.1029958 | 5.843325448 | -5.75156786 | 5.06E-06 | 0.000199281 | 4.070144266 |
| FPR3 | 1.75437814 | 10.64496249 | 5.751283895 | 5.06E-06 | 0.000199281 | 4.069431475 |
| BARX1 | -1.6144144 | 6.78100022 | -5.737278908 | 5.25E-06 | 0.000205465 | 4.034269647 |
| ICAM1 | 2.27930382 | 10.4631707 | 5.732160041 | 5.32E-06 | 0.00020707 | 4.021414313 |
| LOC100128593 | 1.14591865 | 6.439782721 | 5.73084007 | 5.33E-06 | 0.000207398 | 4.018099077 |
| ZNF232 | 1.13682106 | 8.887417676 | 5.723162644 | 5.44E-06 | 0.000210036 | 3.99881398 |

| | | | | | | |
|---|---|---|---|---|---|---|
| NLRC5 | 1.07729571 | 7.973632183 | 5.721769652 | 5.46E-06 | 0.000210036 | 3.995314435 |
| A_24_P68222 | 1.09789021 | 7.816328288 | 5.717610958 | 5.52E-06 | 0.000211919 | 3.984865931 |
| PRICKLE1 | -1.6526257 | 8.062148196 | -5.714947985 | 5.56E-06 | 0.000212723 | 3.9781747 |
| CRAT | -1.7356721 | 12.52434526 | -5.71476576 | 5.56E-06 | 0.000212723 | 3.977716806 |
| MLYCD | -1.4541809 | 7.484437341 | -5.712882439 | 5.59E-06 | 0.00021338 | 3.972984269 |
| ENTPD3 | -3.3711814 | 6.417541133 | -5.698312512 | 5.80E-06 | 0.000219599 | 3.936363461 |
| NINL | -1.4808288 | 8.245795846 | -5.690001077 | 5.93E-06 | 0.000222793 | 3.915466376 |
| CENPK | 1.78358313 | 7.533333408 | 5.681185029 | 6.06E-06 | 0.000225565 | 3.893295304 |
| DUXA | 1.11011221 | 5.049229091 | 5.672165001 | 6.20E-06 | 0.000229684 | 3.870605696 |
| SGEF | -1.7464461 | 6.22159367 | -5.66853463 | 6.26E-06 | 0.000231054 | 3.861472032 |
| PPIAL4A | 1.01474313 | 14.87498409 | 5.66553506 | 6.31E-06 | 0.000232122 | 3.853924723 |
| CCL3L3 | 1.7940387 | 8.108619282 | 5.665427422 | 6.31E-06 | 0.000232122 | 3.853653881 |
| SLAMF8 | 1.994987 | 10.55464263 | 5.664631559 | 6.32E-06 | 0.000232205 | 3.851651278 |
| BMP7 | -1.7174642 | 7.198551689 | -5.657140343 | 6.45E-06 | 0.000234657 | 3.832799278 |
| KRTAP11.1 | 1.113061 | 5.901020189 | 5.656637038 | 6.46E-06 | 0.000234657 | 3.831532551 |
| TIAM2 | -1.4304545 | 9.654140513 | -5.653859106 | 6.50E-06 | 0.000235394 | 3.824540698 |
| DTX2 | 1.18388289 | 9.810234594 | 5.653476956 | 6.51E-06 | 0.000235394 | 3.823578813 |
| SLC16A2 | -2.0355678 | 7.935035954 | -5.651940668 | 6.54E-06 | 0.000235935 | 3.819711825 |
| LOC100133478 | 1.98051028 | 7.392507597 | 5.649965964 | 6.57E-06 | 0.000236499 | 3.814741066 |
| C6orf168 | -1.3246898 | 4.718288404 | -5.641551955 | 6.71E-06 | 0.00024034 | 3.793558268 |
| KLK10 | 1.97969528 | 5.60590722 | 5.634548173 | 6.83E-06 | 0.000243703 | 3.775922221 |
| TNNC2 | -1.6462542 | 6.747393839 | -5.627295158 | 6.96E-06 | 0.000246493 | 3.757655182 |
| SYT17 | -1.384092 | 7.325596189 | -5.62344244 | 7.03E-06 | 0.00024742 | 3.747950542 |
| CTSD | 1.07619729 | 13.02050389 | 5.623321969 | 7.04E-06 | 0.00024742 | 3.747647073 |
| TMEM52 | -1.6908467 | 8.471645864 | -5.621527413 | 7.07E-06 | 0.000248165 | 3.743126407 |
| NMI | 1.0306586 | 11.29984726 | 5.619294 | 7.11E-06 | 0.000249196 | 3.737499928 |
| ENST00000455537 | -1.3095457 | 4.624654022 | -5.616691476 | 7.16E-06 | 0.000249769 | 3.730943165 |
| MED29 | 1.08530103 | 6.751236793 | 5.616532527 | 7.16E-06 | 0.000249769 | 3.730542697 |
| IGSF6 | 2.26481142 | 8.901511331 | 5.611334962 | 7.26E-06 | 0.000251926 | 3.717446661 |
| OPN1SW | 1.22223269 | 5.37227652 | 5.610594148 | 7.27E-06 | 0.000252005 | 3.715579931 |
| TTLL7 | -1.3798163 | 5.662486904 | -5.597479159 | 7.52E-06 | 0.000258615 | 3.682526471 |
| ISPD | -1.2818987 | 5.082191025 | -5.59481277 | 7.57E-06 | 0.00025919 | 3.675805086 |
| LOC732419 | 1.04531797 | 7.016140286 | 5.592051267 | 7.63E-06 | 0.000260613 | 3.668843468 |
| A_33_P3332581 | -1.0384393 | 4.100837814 | -5.584191107 | 7.78E-06 | 0.00026388 | 3.649025762 |
| CHI3L1 | 3.64220236 | 9.526205651 | 5.579912965 | 7.87E-06 | 0.000265873 | 3.638237728 |

| | | | | | | |
|---|---|---|---|---|---|---|
| TDGF1 | -2.4658358 | 6.989638616 | -5.579466464 | 7.88E-06 | 0.000265873 | 3.637111736 |
| CAMK2B | -1.4213166 | 5.128506146 | -5.578281585 | 7.90E-06 | 0.000266274 | 3.634123636 |
| SLC38A6 | 1.40327363 | 5.840788719 | 5.570457041 | 8.06E-06 | 0.000269618 | 3.614389059 |
| INSM1 | -2.4521679 | 5.378825702 | -5.566979901 | 8.14E-06 | 0.000270581 | 3.605618025 |
| ENST00000390027 | -1.4904152 | 5.105256999 | -5.565781876 | 8.16E-06 | 0.000270581 | 3.602595854 |
| SLC15A1 | -4.8372556 | 6.637348349 | -5.54983175 | 8.50E-06 | 0.000279638 | 3.562351363 |
| OR8B8 | 1.14698615 | 7.305673218 | 5.546178819 | 8.59E-06 | 0.000281439 | 3.553132339 |
| AMIGO1 | -1.7732357 | 6.932343205 | -5.544031929 | 8.63E-06 | 0.000282578 | 3.54771379 |
| KBTBD11 | -2.6781365 | 9.780712231 | -5.538839346 | 8.75E-06 | 0.000284255 | 3.534607072 |
| TMEM136 | -1.2998638 | 8.391078033 | -5.537864039 | 8.77E-06 | 0.000284547 | 3.532145099 |
| WFDC2 | -4.5039631 | 9.138740827 | -5.535763395 | 8.82E-06 | 0.000285639 | 3.526842243 |
| FERMT1 | 1.87095037 | 11.02482954 | 5.535228001 | 8.83E-06 | 0.000285639 | 3.525490658 |
| TNRC4 | -1.4734922 | 5.513954912 | -5.534344346 | 8.85E-06 | 0.000285867 | 3.523259858 |
| NDRG4 | -1.654618 | 8.243832159 | -5.533335227 | 8.88E-06 | 0.000286188 | 3.520712266 |
| DACT2 | -1.8976573 | 5.572963219 | -5.530180451 | 8.95E-06 | 0.000287678 | 3.512747431 |
| LRRC49 | -1.2977213 | 7.604081379 | -5.521229398 | 9.16E-06 | 0.000292957 | 3.490145645 |
| SUMO1P3 | 1.07890388 | 8.600449453 | 5.520602725 | 9.17E-06 | 0.000292957 | 3.488563096 |
| IL2RA | 2.03519085 | 7.068821981 | 5.510497982 | 9.41E-06 | 0.000298639 | 3.463042298 |
| SMO | -1.2182678 | 8.664417371 | -5.499683566 | 9.68E-06 | 0.000304968 | 3.435722722 |
| IFITM1 | 1.93782046 | 13.8927283 | 5.49601201 | 9.77E-06 | 0.000306989 | 3.426446079 |
| GJB2 | 1.6858955 | 10.01903385 | 5.494898237 | 9.80E-06 | 0.00030743 | 3.423631844 |
| SELE | 1.69346873 | 5.827789859 | 5.482303357 | 1.01E-05 | 0.000314889 | 3.391802874 |
| GGT1 | -1.2919514 | 8.634034743 | -5.470692607 | 1.04E-05 | 0.00032357 | 3.362453272 |
| C16orf45 | -1.6051198 | 9.167104175 | -5.460489205 | 1.07E-05 | 0.000328462 | 3.33665519 |
| FILIP1L | -1.0667355 | 7.83418219 | -5.445741634 | 1.11E-05 | 0.000335742 | 3.29935803 |
| WARS | 2.03479728 | 12.73521145 | 5.430349841 | 1.16E-05 | 0.000346841 | 3.260419646 |
| ABCC6P1 | -2.6511988 | 6.496712873 | -5.428086644 | 1.17E-05 | 0.0003478 | 3.254693164 |
| CXCL16 | 1.2055683 | 10.78629654 | 5.411739904 | 1.22E-05 | 0.00035944 | 3.213324043 |
| TLE6 | 1.32084537 | 6.92412359 | 5.391168494 | 1.28E-05 | 0.000374133 | 3.161244938 |
| NFAM1 | 1.62199589 | 8.947302222 | 5.384860923 | 1.30E-05 | 0.000377748 | 3.145272502 |
| MLF1 | -1.2606378 | 6.201595882 | -5.38402089 | 1.31E-05 | 0.000377748 | 3.143145174 |
| ZNF683 | 2.07769492 | 9.842594824 | 5.383889401 | 1.31E-05 | 0.000377748 | 3.142812185 |
| GCOM1 | -1.2496702 | 8.506888968 | -5.380582997 | 1.32E-05 | 0.000378953 | 3.134438604 |
| C19orf46 | -1.8704957 | 9.205956169 | -5.374432717 | 1.34E-05 | 0.000383933 | 3.118861467 |
| NPW | -1.5181888 | 5.554222232 | -5.372726023 | 1.35E-05 | 0.000384216 | 3.114538526 |

| | | | | | | |
|---|---|---|---|---|---|---|
| CAND2 | -1.2554908 | 6.769144321 | -5.368673348 | 1.36E-05 | 0.000386272 | 3.10427284 |
| CHRNB2 | -1.0677277 | 5.131363563 | -5.368649604 | 1.36E-05 | 0.000386272 | 3.104212693 |
| CTSZ | 1.15599266 | 10.4955358 | 5.364074803 | 1.38E-05 | 0.000388347 | 3.092623531 |
| CNTFR | -1.5452058 | 4.834030038 | -5.358987966 | 1.39E-05 | 0.000392489 | 3.079736146 |
| ARL4D | -1.2262145 | 6.003806075 | -5.357444558 | 1.40E-05 | 0.000392808 | 3.07582573 |
| IRF1 | 1.43820957 | 10.85463608 | 5.357180465 | 1.40E-05 | 0.000392808 | 3.075156607 |
| PBX1 | -1.3834042 | 8.370713109 | -5.350262483 | 1.43E-05 | 0.00039701 | 3.057627664 |
| OR9A1P | 1.65096092 | 4.973181275 | 5.331464965 | 1.50E-05 | 0.000410894 | 3.009987592 |
| TEKT2 | -1.5461995 | 5.915683984 | -5.305686693 | 1.60E-05 | 0.000433338 | 2.944631556 |
| ZNF605 | -1.9020663 | 8.575133933 | -5.305224925 | 1.60E-05 | 0.000433338 | 2.943460582 |
| CTSC | 1.33821698 | 9.632170913 | 5.301712462 | 1.62E-05 | 0.000435772 | 2.934553234 |
| TMEM44 | 1.18039409 | 8.046597053 | 5.291329909 | 1.66E-05 | 0.000444299 | 2.908221006 |
| HLA.DRB1 | 1.51593165 | 15.47712422 | 5.285594953 | 1.69E-05 | 0.000449865 | 2.893674224 |
| ANGPTL7 | -1.6615769 | 4.385494084 | -5.283135953 | 1.70E-05 | 0.000451642 | 2.887436558 |
| SLC6A8 | -2.6946805 | 7.543678578 | -5.281418456 | 1.71E-05 | 0.000452774 | 2.8830797 |
| CD47 | 1.14909605 | 12.49678276 | 5.2788781 | 1.72E-05 | 0.000453842 | 2.876635255 |
| APCDD1 | -2.407496 | 8.70575728 | -5.278629521 | 1.72E-05 | 0.000453842 | 2.876004642 |
| TRIM63 | -1.6916834 | 4.960095508 | -5.278452163 | 1.72E-05 | 0.000453842 | 2.875554702 |
| ENST00000367555 | 1.38290999 | 7.786421561 | 5.275874721 | 1.73E-05 | 0.000455354 | 2.86901589 |
| PNMT | -1.1515942 | 4.846678812 | -5.270798072 | 1.75E-05 | 0.000459614 | 2.856136016 |
| GGTLC1 | -1.8995577 | 9.294680028 | -5.267470779 | 1.77E-05 | 0.000461381 | 2.847693888 |
| MXI1 | -1.1276335 | 8.291843536 | -5.256679828 | 1.82E-05 | 0.000469441 | 2.820311931 |
| DPY19L1P1 | 1.18666248 | 7.269233753 | 5.255398795 | 1.82E-05 | 0.000469892 | 2.817061044 |
| SLC47A1 | 1.25667934 | 5.655319771 | 5.250006679 | 1.85E-05 | 0.000475402 | 2.803376793 |
| LOC100131432 | 1.13219962 | 6.630351239 | 5.244789433 | 1.88E-05 | 0.000480475 | 2.790135364 |
| NR4A1 | 1.87393494 | 5.540898111 | 5.244315599 | 1.88E-05 | 0.000480475 | 2.78893272 |
| ADIPOR2 | -1.0469033 | 11.45615633 | -5.239405112 | 1.90E-05 | 0.000484702 | 2.776468915 |
| CASP1 | 1.51896139 | 12.20266482 | 5.238942154 | 1.90E-05 | 0.000484702 | 2.775293792 |
| PGM2 | 1.04951672 | 9.720706793 | 5.234277466 | 1.93E-05 | 0.000489475 | 2.763453041 |
| CHGB | -2.9071839 | 6.371192648 | -5.223753098 | 1.98E-05 | 0.000500726 | 2.736735538 |
| CLEC7A | 1.38066484 | 5.454242174 | 5.221773517 | 1.99E-05 | 0.000502728 | 2.731709704 |
| TNFSF14 | 1.50861649 | 8.333746931 | 5.21833026 | 2.01E-05 | 0.000505492 | 2.722967534 |
| B3GALNT1 | -1.0927469 | 8.552673558 | -5.212497914 | 2.04E-05 | 0.000507934 | 2.70815878 |
| ENPP3 | -1.5751307 | 5.602916952 | -5.211009478 | 2.05E-05 | 0.000509322 | 2.704379361 |
| ABHD8 | -1.3476075 | 10.540149 | -5.208589279 | 2.06E-05 | 0.000510788 | 2.698233872 |

| | | | | | | |
|---|---|---|---|---|---|---|
| SCIN | -3.2376625 | 7.761169153 | -5.202440458 | 2.09E-05 | 0.000515869 | 2.682619664 |
| SLC37A2 | 1.15804042 | 7.187628453 | 5.202428596 | 2.09E-05 | 0.000515869 | 2.68258954 |
| LOC145216 | 1.14982454 | 7.166872356 | 5.198274331 | 2.12E-05 | 0.000519361 | 2.672039616 |
| WNK4 | -1.3969127 | 7.624509039 | -5.193983642 | 2.14E-05 | 0.000522249 | 2.661142693 |
| AASS | -1.535098 | 7.057486641 | -5.189363696 | 2.17E-05 | 0.000526796 | 2.649408954 |
| NPL | 1.41926497 | 7.475262733 | 5.184518851 | 2.19E-05 | 0.00053229 | 2.637103344 |
| GDF1 | -1.0627064 | 5.140111605 | -5.180109402 | 2.22E-05 | 0.000535271 | 2.625903027 |
| PLTP | -1.6287708 | 10.35194908 | -5.168508188 | 2.29E-05 | 0.000546423 | 2.596432488 |
| SLC25A27 | -1.577414 | 5.68442029 | -5.161183453 | 2.33E-05 | 0.000554735 | 2.577823578 |
| GZMB | 2.06594925 | 11.65072231 | 5.161029512 | 2.33E-05 | 0.000554735 | 2.577432467 |
| MYOT | -1.0320957 | 4.828691867 | -5.156816177 | 2.36E-05 | 0.000559438 | 2.566727579 |
| PRKY | 1.00725509 | 8.766409661 | 5.146696894 | 2.42E-05 | 0.000570847 | 2.541015472 |
| F10 | -2.1504875 | 7.40640755 | -5.145923031 | 2.43E-05 | 0.00057138 | 2.539049054 |
| LOC148189 | -1.1057495 | 7.157203269 | -5.143395882 | 2.44E-05 | 0.00057369 | 2.53262736 |
| IL27RA | 1.42160823 | 9.803088116 | 5.142222323 | 2.45E-05 | 0.00057369 | 2.529645195 |
| FOXD3 | -1.139747 | 4.630370353 | -5.142114498 | 2.45E-05 | 0.00057369 | 2.529371197 |
| SERHL2 | -1.3233191 | 6.806001924 | -5.141484967 | 2.45E-05 | 0.00057369 | 2.52777146 |
| MAPT | -2.0052359 | 5.600647027 | -5.138844822 | 2.47E-05 | 0.000576397 | 2.521062341 |
| DHDPSL | 1.16891837 | 5.59315999 | 5.132800242 | 2.51E-05 | 0.000582862 | 2.50570125 |
| TXNDC3 | 1.49485705 | 6.158738865 | 5.125621115 | 2.56E-05 | 0.000589691 | 2.487455805 |
| COL22A1 | 2.10301065 | 6.311333743 | 5.125595182 | 2.56E-05 | 0.000589691 | 2.487389895 |
| FAM13A | -1.6307366 | 9.773120222 | -5.121868847 | 2.58E-05 | 0.000592932 | 2.477919097 |
| LOC100288921 | -1.3514399 | 5.324070445 | -5.113300125 | 2.64E-05 | 0.000602368 | 2.456139768 |
| TMEM62 | 1.08116331 | 9.218314418 | 5.106478887 | 2.69E-05 | 0.000609951 | 2.438800907 |
| CAND1 | 1.22229391 | 9.615036708 | 5.09782669 | 2.75E-05 | 0.000620803 | 2.416806521 |
| ADAMTS18 | 1.46830785 | 5.042669803 | 5.096360815 | 2.76E-05 | 0.000622534 | 2.41308003 |
| PPP1R9A | -2.6157704 | 6.929795493 | -5.090858622 | 2.80E-05 | 0.000628922 | 2.399092175 |
| GZMH | 1.65357547 | 10.95225707 | 5.086305918 | 2.83E-05 | 0.000634468 | 2.387517689 |
| RAB42 | 1.75694803 | 8.022562939 | 5.078270887 | 2.89E-05 | 0.000643268 | 2.367089005 |
| ENST00000360201 | -1.3549034 | 6.86007547 | -5.075270227 | 2.92E-05 | 0.000645682 | 2.359459661 |
| TIMM8A | 1.03693969 | 9.902064258 | 5.074233389 | 2.93E-05 | 0.000646773 | 2.356823403 |
| CDRT8 | 1.19910294 | 5.096018508 | 5.071860648 | 2.94E-05 | 0.000649015 | 2.350790419 |
| UGT3A1 | 1.01405044 | 5.639699918 | 5.068543336 | 2.97E-05 | 0.000653785 | 2.342355578 |
| FAM169A | 1.25576972 | 8.14637453 | 5.067424447 | 2.98E-05 | 0.000654535 | 2.339510563 |
| CERK | -1.2789019 | 9.008494657 | -5.067327494 | 2.98E-05 | 0.000654535 | 2.33926404 |

| | | | | | | |
|---|---|---|---|---|---|---|
| CRHR2 | 1.01212562 | 5.238219235 | 5.055089266 | 3.08E-05 | 0.000671023 | 2.308144334 |
| C15orf52 | -1.5754587 | 10.54319835 | -5.054413249 | 3.08E-05 | 0.000671534 | 2.306425268 |
| PKDREJ | -1.1887319 | 5.222903208 | -5.051440866 | 3.10E-05 | 0.000674732 | 2.298866606 |
| LOC729897 | 1.15998126 | 5.217398571 | 5.047103498 | 3.14E-05 | 0.000681731 | 2.287836588 |
| WIPF3 | -1.4276288 | 5.891954898 | -5.046380453 | 3.15E-05 | 0.000682337 | 2.285997843 |
| PCDH20 | -2.2611789 | 5.060980919 | -5.03656809 | 3.23E-05 | 0.000692214 | 2.261043645 |
| CD80 | 1.15236152 | 5.568775945 | 5.028089742 | 3.30E-05 | 0.000700886 | 2.239480926 |
| ENST00000395354 | 1.30360317 | 6.463289561 | 5.027781566 | 3.30E-05 | 0.000700886 | 2.238697134 |
| NPY1R | -1.2726423 | 4.879006421 | -5.020046469 | 3.37E-05 | 0.000711707 | 2.219023834 |
| ITGAX | 1.77928108 | 9.040445992 | 5.019452434 | 3.37E-05 | 0.000712119 | 2.217512944 |
| ETNK2 | -1.1108706 | 5.551056543 | -5.013567763 | 3.43E-05 | 0.000720346 | 2.202545437 |
| PCDHA11 | -1.4487681 | 5.297966723 | -5.009461665 | 3.46E-05 | 0.000727406 | 2.192101441 |
| FCGR3A | 2.17804124 | 11.88735887 | 5.008149756 | 3.48E-05 | 0.000729197 | 2.188764516 |
| VSTM2L | -1.5540711 | 5.690283897 | -5.00718672 | 3.48E-05 | 0.000730328 | 2.186314958 |
| PSMB9 | 1.1514573 | 13.68116901 | 5.001675209 | 3.54E-05 | 0.000738776 | 2.17229581 |
| LOC100129509 | 1.27118521 | 4.950714945 | 4.996246507 | 3.59E-05 | 0.000745024 | 2.158486979 |
| PRLR | -1.3113346 | 5.560258304 | -4.991063485 | 3.63E-05 | 0.0007507 | 2.145302801 |
| PDZRN3 | -2.1925137 | 7.183910968 | -4.990642557 | 3.64E-05 | 0.0007507 | 2.144232065 |
| SLC25A18 | -1.6977163 | 4.539307367 | -4.989986666 | 3.64E-05 | 0.000750842 | 2.142563639 |
| FBXO22OS | 1.87314168 | 6.785844559 | 4.986366843 | 3.68E-05 | 0.000754398 | 2.133355622 |
| LOC100128916 | 1.00033158 | 6.123366233 | 4.985211421 | 3.69E-05 | 0.000755964 | 2.130416463 |
| TNFAIP3 | 1.42509337 | 10.1185153 | 4.980782134 | 3.73E-05 | 0.00076136 | 2.119149147 |
| ENST00000382123 | 1.17392093 | 5.573408888 | 4.98068787 | 3.73E-05 | 0.00076136 | 2.118909354 |
| ENST00000372315 | 1.04093098 | 5.545219783 | 4.974106147 | 3.80E-05 | 0.00077311 | 2.102166321 |
| DEPDC6 | -1.5532874 | 10.53761365 | -4.96426655 | 3.90E-05 | 0.000787749 | 2.077135078 |
| MNX1 | 1.06106148 | 10.28350825 | 4.957656092 | 3.97E-05 | 0.000797346 | 2.060318174 |
| LILRA4 | 1.26851133 | 7.83213369 | 4.952678462 | 4.02E-05 | 0.000802584 | 2.047654988 |
| IFI44 | 1.02078028 | 11.78744956 | 4.948165472 | 4.07E-05 | 0.000810612 | 2.036173739 |
| ZNF134 | -1.1169781 | 6.652325784 | -4.94722326 | 4.08E-05 | 0.000811865 | 2.033776697 |
| PON3 | -2.2728975 | 8.028893862 | -4.945434921 | 4.09E-05 | 0.00081492 | 2.029227048 |
| GGT3P | -1.2267857 | 7.538851565 | -4.921276506 | 4.36E-05 | 0.00085646 | 1.967765312 |
| AGRN | 1.10648983 | 10.43467275 | 4.921184531 | 4.36E-05 | 0.00085646 | 1.967531314 |
| PPP1R2P3 | 1.11154459 | 5.953374494 | 4.897976479 | 4.64E-05 | 0.000892277 | 1.90848635 |
| JAKMIP1 | 1.25743365 | 6.91607468 | 4.895984304 | 4.66E-05 | 0.000894558 | 1.903417938 |
| CCL4 | 1.61145903 | 11.40093905 | 4.892110619 | 4.71E-05 | 0.000902143 | 1.893562676 |

| | | | | | | |
|---|---|---|---|---|---|---|
| C12orf53 | -1.5651052 | 4.842886256 | -4.892078387 | 4.71E-05 | 0.000902143 | 1.893480674 |
| CXCL1 | 2.17677979 | 11.08965792 | 4.885479531 | 4.79E-05 | 0.000913808 | 1.876692199 |
| HRASLS | -1.2967141 | 5.582943367 | -4.883798498 | 4.81E-05 | 0.000917027 | 1.872415415 |
| ENTPD8 | -3.9960155 | 9.617827517 | -4.878310287 | 4.88E-05 | 0.000927348 | 1.858452678 |
| MGAT5B | -1.2434196 | 5.531125702 | -4.878181949 | 4.88E-05 | 0.000927348 | 1.858126172 |
| C15orf53 | 1.00474127 | 4.830439787 | 4.861048961 | 5.10E-05 | 0.000958913 | 1.81453824 |
| MAGI3 | -1.0511364 | 9.901793985 | -4.859471604 | 5.13E-05 | 0.00096155 | 1.810525359 |
| IRS2 | -1.825997 | 9.072982691 | -4.857601449 | 5.15E-05 | 0.000962603 | 1.805767601 |
| SCN3B | -1.5451932 | 6.343949138 | -4.849599074 | 5.26E-05 | 0.000975404 | 1.785409416 |
| TMEFF2 | -1.0119718 | 5.002516144 | -4.833176274 | 5.49E-05 | 0.001008636 | 1.743630827 |
| SCN7A | -1.0992134 | 4.713460566 | -4.826276309 | 5.59E-05 | 0.001020193 | 1.726078364 |
| TTC25 | -1.1610772 | 5.61585264 | -4.82547553 | 5.60E-05 | 0.001021474 | 1.724041331 |
| ADAMTS17 | -1.3747177 | 5.959610095 | -4.820287837 | 5.68E-05 | 0.001031626 | 1.710844943 |
| SLC4A11 | 1.40058122 | 9.572867328 | 4.820092081 | 5.68E-05 | 0.001031626 | 1.710346986 |
| RP11.431O22.2 | 1.07117113 | 13.34129821 | 4.816200893 | 5.74E-05 | 0.001038705 | 1.700448806 |
| UPK1B | -3.4396524 | 5.766043932 | -4.791638792 | 6.12E-05 | 0.001086795 | 1.637972965 |
| C4orf40 | 1.82208961 | 5.05849955 | 4.788639895 | 6.17E-05 | 0.001093689 | 1.630345512 |
| GNGT2 | 1.27300427 | 6.966477226 | 4.788284385 | 6.17E-05 | 0.001093689 | 1.629441308 |
| RUNX1 | 1.06166785 | 8.644693577 | 4.787466271 | 6.19E-05 | 0.001094248 | 1.627360525 |
| RPL23AP64 | 1.16727362 | 5.868275938 | 4.783875935 | 6.25E-05 | 0.001102776 | 1.618229009 |
| LOC644662 | -1.4393503 | 5.077263341 | -4.778855765 | 6.33E-05 | 0.001112829 | 1.605461212 |
| PDE8B | -1.5947123 | 7.646867929 | -4.776951344 | 6.36E-05 | 0.001115484 | 1.600617794 |
| C1QC | 1.58471261 | 10.17617166 | 4.776577375 | 6.37E-05 | 0.001115484 | 1.599666703 |
| RGS1 | 1.89488237 | 12.05028419 | 4.773863227 | 6.41E-05 | 0.001120387 | 1.592764053 |
| GOLSYN | -1.8521167 | 8.540001407 | -4.772923365 | 6.43E-05 | 0.001121157 | 1.590373812 |
| HYLS1 | -1.6740047 | 7.908867745 | -4.767189305 | 6.52E-05 | 0.001133531 | 1.575791346 |
| NUDT16 | -1.2260508 | 9.449273708 | -4.765089041 | 6.56E-05 | 0.001137064 | 1.57045023 |
| LASS1 | -1.2321578 | 5.849002822 | -4.759462318 | 6.66E-05 | 0.001151166 | 1.556141436 |
| LAP3 | 1.35762316 | 12.42701719 | 4.754296924 | 6.75E-05 | 0.001163121 | 1.543006274 |
| HSPB3 | -1.1700039 | 4.444049306 | -4.741702094 | 6.97E-05 | 0.00119429 | 1.510980674 |
| LOC100128219 | 1.11767093 | 5.826208201 | 4.738905351 | 7.03E-05 | 0.001199482 | 1.503869634 |
| SRMS | 1.22255157 | 6.994433451 | 4.7367972 | 7.06E-05 | 0.001203278 | 1.498509519 |
| DYNC2H1 | -1.1387179 | 6.800457405 | -4.725925598 | 7.27E-05 | 0.001225497 | 1.470869152 |
| GUCY2G | 1.301634 | 5.860690402 | 4.72379635 | 7.31E-05 | 0.001231386 | 1.465455957 |
| FCGR2A | 1.35464293 | 10.20125304 | 4.720982311 | 7.36E-05 | 0.001236647 | 1.458301963 |

| | | | | | | |
|---|---|---|---|---|---|---|
| APOE | 2.17832004 | 15.84921253 | 4.715287912 | 7.47E-05 | 0.001247491 | 1.443825896 |
| NHS | 1.00645276 | 8.163945465 | 4.714619815 | 7.49E-05 | 0.001247957 | 1.442127536 |
| LOC340357 | 1.44740136 | 5.859160454 | 4.712469908 | 7.53E-05 | 0.001250954 | 1.43666235 |
| CD226 | 1.03639961 | 5.812400314 | 4.710342761 | 7.57E-05 | 0.001256013 | 1.431255124 |
| SLC22A2 | 1.08839739 | 4.925997847 | 4.709925503 | 7.58E-05 | 0.001256425 | 1.430194463 |
| ANXA2 | 1.60133906 | 12.05414554 | 4.702630169 | 7.72E-05 | 0.001278678 | 1.411650527 |
| ATP2C1 | 1.06508059 | 9.932153117 | 4.701313591 | 7.75E-05 | 0.001281137 | 1.408304062 |
| PF4 | -1.8215546 | 7.379139232 | -4.698008994 | 7.82E-05 | 0.001289316 | 1.39990466 |
| SCO2 | 1.27656226 | 11.41993747 | 4.696326667 | 7.85E-05 | 0.00129304 | 1.395628738 |
| LTK | -1.4333172 | 11.01675728 | -4.69508358 | 7.88E-05 | 0.001294004 | 1.392469264 |
| BATF2 | 1.66199963 | 10.45885099 | 4.694884867 | 7.88E-05 | 0.001294004 | 1.391964212 |
| HOXA4 | -1.5081971 | 6.987541893 | -4.691587462 | 7.95E-05 | 0.001302612 | 1.383583612 |
| C19orf59 | 1.49115298 | 5.879036154 | 4.690074681 | 7.98E-05 | 0.001302612 | 1.379738856 |
| TBX21 | 1.05754881 | 7.783957835 | 4.690046613 | 7.98E-05 | 0.001302612 | 1.379667522 |
| TFEC | 1.70210363 | 7.340914753 | 4.687882355 | 8.03E-05 | 0.001307062 | 1.374167134 |
| SLC27A6 | -1.479156 | 4.763533597 | -4.687057889 | 8.05E-05 | 0.001308903 | 1.372071811 |
| PRKAB2 | -1.3691218 | 9.176797417 | -4.684329171 | 8.10E-05 | 0.001314343 | 1.365137098 |
| APOBEC3H | 1.17196842 | 6.497927367 | 4.678417352 | 8.23E-05 | 0.001327886 | 1.350113579 |
| LOC729970 | -1.1765745 | 7.730007978 | -4.676246908 | 8.28E-05 | 0.001333466 | 1.344598133 |
| DKFZP564C152 | -1.9692 | 10.53780991 | -4.675026085 | 8.30E-05 | 0.001334737 | 1.341495882 |
| C21orf88 | -1.114129 | 8.069820551 | -4.672432267 | 8.36E-05 | 0.001341399 | 1.334904829 |
| MOCS1 | -1.5188569 | 6.913453791 | -4.668855755 | 8.44E-05 | 0.001348498 | 1.325816996 |
| ADAMTS14 | 1.67207913 | 7.394007407 | 4.666186135 | 8.50E-05 | 0.001354957 | 1.319033787 |
| HTRA4 | 2.3130232 | 6.81699109 | 4.664093131 | 8.54E-05 | 0.001358245 | 1.313715839 |
| CCDC122 | -1.5543835 | 6.827910479 | -4.661912376 | 8.59E-05 | 0.001362542 | 1.308175065 |
| WTIP | -1.3028594 | 9.979209004 | -4.659372611 | 8.65E-05 | 0.001370271 | 1.301722304 |
| NAP1L5 | -1.4234746 | 7.472773663 | -4.657289065 | 8.70E-05 | 0.001374752 | 1.296428799 |
| C9orf122 | -2.4247639 | 6.528704739 | -4.652799644 | 8.80E-05 | 0.001385949 | 1.285023309 |
| MMP25 | 1.05006408 | 7.591465964 | 4.646577428 | 8.94E-05 | 0.001403602 | 1.269216615 |
| ENST00000378898 | -1.0188509 | 5.366522745 | -4.642487624 | 9.04E-05 | 0.001413746 | 1.258827677 |
| LRRC3B | -1.0329585 | 4.865004039 | -4.642359214 | 9.04E-05 | 0.001413746 | 1.258501497 |
| LOC285972 | 1.14067935 | 6.033762911 | 4.639684253 | 9.11E-05 | 0.001421523 | 1.251706844 |
| ENST00000400419 | 1.09028678 | 4.563956443 | 4.639071992 | 9.12E-05 | 0.001422184 | 1.250151675 |
| ELOVL7 | 1.13267357 | 8.418498579 | 4.63046987 | 9.33E-05 | 0.00144418 | 1.228303206 |
| LILRB4 | 1.25660768 | 8.068831158 | 4.626663612 | 9.42E-05 | 0.001453876 | 1.218636493 |

| | | | | | | |
|---|---|---|---|---|---|---|
| TM4SF4 | -3.8941436 | 7.412091187 | -4.618445506 | 9.63E-05 | 0.001473523 | 1.197766726 |
| LOC650157 | 1.14018529 | 6.142594778 | 4.611044565 | 9.82E-05 | 0.001493031 | 1.178974135 |
| HIST1H1A | -1.2095593 | 7.868430967 | -4.609982874 | 9.84E-05 | 0.001495961 | 1.176278432 |
| ZP1 | -1.0210756 | 5.864097227 | -4.608977591 | 9.87E-05 | 0.001496759 | 1.17372599 |
| IFI44L | 1.69446431 | 10.10049155 | 4.603142501 | 0.000100204 | 0.001510287 | 1.158911243 |
| HCN4 | -1.4139556 | 4.941562905 | -4.579687888 | 0.000106542 | 0.001586015 | 1.099375037 |
| C6orf174 | -1.3645946 | 6.561207701 | -4.574664557 | 0.00010795 | 0.001602593 | 1.086626811 |
| ENST00000318291 | -1.0528182 | 8.241878418 | -4.574292781 | 0.000108055 | 0.001603056 | 1.085683358 |
| STX19 | -2.3524422 | 8.851666916 | -4.565447002 | 0.000110584 | 0.001636012 | 1.063237135 |
| APOL3 | 1.03403134 | 8.864397111 | 4.560710267 | 0.000111962 | 0.001648628 | 1.051218975 |
| VPS37D | -1.1792046 | 8.816484219 | -4.55423975 | 0.000113872 | 0.001669966 | 1.03480336 |
| SOCS3 | 1.51903337 | 10.77670741 | 4.553824953 | 0.000113995 | 0.00167065 | 1.033751085 |
| LOC645722 | -1.0111449 | 8.172179053 | -4.550302566 | 0.00011505 | 0.00168044 | 1.024815656 |
| RENBP | 1.5034595 | 6.635963961 | 4.538638432 | 0.000118612 | 0.001718609 | 0.995230529 |
| C4BPA | 2.55890109 | 6.887033726 | 4.533925012 | 0.000120083 | 0.001730685 | 0.983277061 |
| ENST00000373544 | 1.51811724 | 9.755695925 | 4.53285163 | 0.00012042 | 0.001734398 | 0.98055505 |
| PRAM1 | 1.29755602 | 8.683576358 | 4.531023149 | 0.000120997 | 0.001741553 | 0.975918296 |
| LDHB | -1.4060025 | 13.57426297 | -4.530255102 | 0.00012124 | 0.001742745 | 0.973970691 |
| FAM110A | 1.04021635 | 12.46653762 | 4.527294899 | 0.000122182 | 0.001750443 | 0.966464493 |
| LOC400743 | 1.03271968 | 5.335440866 | 4.526432622 | 0.000122457 | 0.001752133 | 0.964278092 |
| SOX9 | 1.09729281 | 8.582990986 | 4.524650483 | 0.000123029 | 0.001758753 | 0.959759385 |
| NCF2 | 1.56545512 | 8.772211622 | 4.523435306 | 0.000123421 | 0.00176127 | 0.956678327 |
| PRUNE2 | -1.2011337 | 6.456685689 | -4.523224486 | 0.000123489 | 0.00176127 | 0.956143803 |
| ZNF75A | -1.1348743 | 9.567795647 | -4.522848926 | 0.00012361 | 0.001761664 | 0.955191597 |
| GOLT1A | -2.4764525 | 7.108791199 | -4.521909525 | 0.000123914 | 0.001762549 | 0.952809838 |
| FLJ35024 | -1.1403691 | 4.618039906 | -4.514927223 | 0.000126196 | 0.001787968 | 0.935108217 |
| GNA15 | 1.26263922 | 7.758037267 | 4.511784023 | 0.000127237 | 0.001798665 | 0.927140309 |
| EMR2 | 1.63203351 | 8.17824511 | 4.506032662 | 0.000129164 | 0.001819344 | 0.912562052 |
| HEBP1 | -1.2529432 | 11.85407119 | -4.504783996 | 0.000129586 | 0.001824109 | 0.909397216 |
| VRK2 | 1.14386308 | 9.011899578 | 4.503116397 | 0.000130152 | 0.001829702 | 0.905170683 |
| SLC6A10P | -1.2740595 | 8.577745483 | -4.490741508 | 0.00013443 | 0.001874084 | 0.873810907 |
| TUB | -1.2532658 | 4.996817277 | -4.487724701 | 0.000135494 | 0.001887706 | 0.866167074 |
| CCR1 | 1.35323944 | 9.134493001 | 4.486729237 | 0.000135847 | 0.001890103 | 0.863644922 |
| LEPREL2 | -1.306547 | 11.8123058 | -4.480762188 | 0.000137981 | 0.001910118 | 0.84852764 |
| DDRGK1 | 1.0191924 | 10.1945318 | 4.479707338 | 0.000138362 | 0.001912214 | 0.845855416 |

| | | | | | | |
|---|---|---|---|---|---|---|
| PPAP2B | -1.3902836 | 11.19381778 | -4.476831538 | 0.000139406 | 0.00192127 | 0.838570529 |
| GCNT2 | -1.0182732 | 5.801560041 | -4.467010304 | 0.000143029 | 0.001959089 | 0.813695067 |
| DKK1 | 3.51954415 | 7.841748063 | 4.466357513 | 0.000143273 | 0.001960894 | 0.81204185 |
| LOC646993 | 1.31017075 | 6.781828628 | 4.465928041 | 0.000143434 | 0.00196186 | 0.810954211 |
| ENST00000382794 | -1.1361979 | 4.612315161 | -4.462988989 | 0.00014454 | 0.00196909 | 0.803511316 |
| KCNB1 | -1.3904713 | 4.957233899 | -4.461069416 | 0.000145266 | 0.001973415 | 0.798650425 |
| TMEM63C | -1.2459848 | 7.379563392 | -4.459890916 | 0.000145714 | 0.001976874 | 0.795666239 |
| ISG15 | 1.36680265 | 13.43695642 | 4.45753416 | 0.000146614 | 0.00198338 | 0.789698718 |
| CD84 | 1.37049488 | 7.389630621 | 4.457361715 | 0.00014668 | 0.00198338 | 0.789262084 |
| F11 | -1.2050819 | 5.645409346 | -4.457202116 | 0.000146741 | 0.00198338 | 0.788857977 |
| TTC14 | -1.3348411 | 8.218729937 | -4.456235456 | 0.000147112 | 0.001985983 | 0.786410415 |
| BMPR1B | -1.3147873 | 4.431901303 | -4.451868763 | 0.0001488 | 0.002000304 | 0.77535471 |
| APOA1 | -3.7450608 | 6.812624226 | -4.450955419 | 0.000149155 | 0.002000481 | 0.77304242 |
| TPPP2 | -1.0551811 | 5.066916993 | -4.449386385 | 0.000149768 | 0.00200558 | 0.769070248 |
| LNX1 | -1.452355 | 8.220495537 | -4.44723932 | 0.00015061 | 0.002014377 | 0.763634964 |
| ENST00000400702 | 1.49086833 | 6.719521584 | 4.446626975 | 0.000150851 | 0.002016361 | 0.762084863 |
| RBBP8 | 1.05638455 | 9.890627096 | 4.443150977 | 0.000152227 | 0.002029208 | 0.753286083 |
| KRTAP23.1 | 1.16777553 | 6.020346151 | 4.437411305 | 0.000154526 | 0.002052858 | 0.738758831 |
| STX11 | 1.0783567 | 7.124217139 | 4.434352873 | 0.000155766 | 0.002065534 | 0.731018663 |
| S1PR3 | -1.4899359 | 9.59352918 | -4.431588651 | 0.000156894 | 0.00207923 | 0.724023554 |
| LILRB3 | 1.25620713 | 9.581654797 | 4.430964104 | 0.00015715 | 0.002081355 | 0.722443145 |
| GRIN3A | 1.5602421 | 7.447692515 | 4.429412292 | 0.000157788 | 0.002085039 | 0.718516408 |
| PRKCQ | 1.28655726 | 7.230139213 | 4.427150613 | 0.000158723 | 0.002094528 | 0.712793672 |
| MAGED1 | -1.505719 | 11.27871252 | -4.421266672 | 0.000161181 | 0.002116678 | 0.697906974 |
| ACOX2 | -1.6328892 | 8.120562338 | -4.418490143 | 0.000162354 | 0.002125658 | 0.690882944 |
| C6orf186 | -1.1392465 | 4.44751786 | -4.40552632 | 0.000167943 | 0.002186981 | 0.658093626 |
| ADRA1B | -1.1013982 | 6.157860175 | -4.399963928 | 0.000170399 | 0.002207582 | 0.644027993 |
| LCP2 | 1.14056173 | 10.57086547 | 4.398844744 | 0.000170898 | 0.00220984 | 0.641198152 |
| FCER1G | 1.00579423 | 11.86674388 | 4.398321048 | 0.000171132 | 0.002211284 | 0.639874023 |
| TTTY13 | 1.02917957 | 5.583283903 | 4.393921734 | 0.000173108 | 0.002225677 | 0.628751363 |
| ENHO | -1.1007038 | 7.244905034 | -4.3923783 | 0.000173807 | 0.00222863 | 0.624849445 |
| ENST00000421344 | 1.04634793 | 7.430049152 | 4.388751283 | 0.000175461 | 0.002244533 | 0.615680692 |
| CXCL3 | 2.56578947 | 10.07953866 | 4.388318311 | 0.000175659 | 0.002245749 | 0.61458624 |
| KRBA1 | -1.3080444 | 10.37728708 | -4.384563197 | 0.000177389 | 0.002261218 | 0.605094703 |
| LOC100129555 | -1.4678081 | 6.434357034 | -4.382346435 | 0.000178418 | 0.002271674 | 0.599491994 |

| | | | | | | |
|---|---|---|---|---|---|---|
| GHSR | 1.19355574 | 7.08918392 | 4.381195097 | 0.000178955 | 0.002275844 | 0.596582202 |
| MXRA7 | -1.1528763 | 8.705655381 | -4.377579097 | 0.000180652 | 0.002292058 | 0.587444017 |
| KRTAP13.2 | 1.12682897 | 4.93443682 | 4.376176157 | 0.000181315 | 0.002296444 | 0.583898813 |
| HSPB2 | -1.6767632 | 9.105242416 | -4.371796952 | 0.000183399 | 0.00231609 | 0.572833511 |
| CSRP2 | -1.5240482 | 11.5995105 | -4.369069153 | 0.000184709 | 0.002328576 | 0.565941625 |
| RNF213 | 1.20071199 | 8.331836952 | 4.359131655 | 0.000189561 | 0.002375204 | 0.540838568 |
| NELL1 | -1.165221 | 5.500134366 | -4.356809294 | 0.000190713 | 0.002384901 | 0.53497307 |
| C3orf55 | -2.4645906 | 6.216140013 | -4.348470324 | 0.000194908 | 0.002427576 | 0.513914849 |
| PSAT1 | 1.61118861 | 11.13678835 | 4.346853283 | 0.000195731 | 0.002430677 | 0.509831955 |
| MYO1F | 1.10350332 | 11.06859445 | 4.340147279 | 0.000199185 | 0.002465282 | 0.492901886 |
| PRSS8 | -1.0914089 | 15.06131886 | -4.338317623 | 0.000200138 | 0.002471479 | 0.488283288 |
| LAIR1 | 1.12810199 | 9.701580345 | 4.330916139 | 0.000204039 | 0.00250825 | 0.469602277 |
| CTLA4 | 1.72714598 | 7.656729344 | 4.329444784 | 0.000204824 | 0.002515048 | 0.465889134 |
| PSD3 | -1.2204681 | 7.891031364 | -4.327108954 | 0.000206075 | 0.002521372 | 0.459994718 |
| ZNF347 | -1.1025569 | 6.994705649 | -4.327047261 | 0.000206108 | 0.002521372 | 0.459839043 |
| TNFRSF4 | 1.13966534 | 8.543205581 | 4.326968216 | 0.000206151 | 0.002521372 | 0.459639582 |
| ENST00000344149 | -1.0757546 | 5.298524412 | -4.321834505 | 0.000208929 | 0.002544139 | 0.446686289 |
| ENTPD5 | -1.1981067 | 7.48433612 | -4.321190059 | 0.00020928 | 0.002545327 | 0.445060378 |
| FAM47E | -1.3024602 | 7.757275839 | -4.314718041 | 0.000212842 | 0.002579981 | 0.428733502 |
| HECW2 | 1.37816417 | 7.809718147 | 4.312578932 | 0.000214032 | 0.002590076 | 0.423337913 |
| MBIP | -1.0390325 | 9.62140168 | -4.303675523 | 0.000219058 | 0.00263476 | 0.400884234 |
| FAH | -1.4076098 | 8.619717737 | -4.299613832 | 0.00022139 | 0.002654015 | 0.390643065 |
| SYCE2 | 1.17986212 | 5.810480278 | 4.299398788 | 0.000221514 | 0.002654015 | 0.390100889 |
| APOL4 | 1.00634834 | 7.849214495 | 4.292558214 | 0.000225499 | 0.002695814 | 0.372856145 |
| IGSF9 | -1.1039711 | 12.06557506 | -4.289001815 | 0.000227598 | 0.002708995 | 0.36389214 |
| SAMSN1 | 1.21523761 | 9.219920989 | 4.284939949 | 0.00023002 | 0.002727593 | 0.353655357 |
| MUC16 | 1.02719082 | 4.882674717 | 4.28469893 | 0.000230165 | 0.002727593 | 0.353047981 |
| FOXD4 | 1.22071104 | 6.605080488 | 4.283772569 | 0.000230721 | 0.00272972 | 0.350713559 |
| A2LD1 | -1.5068034 | 8.40338649 | -4.28234907 | 0.000231579 | 0.002737606 | 0.347126492 |
| HSD17B12 | -1.6071341 | 11.51924029 | -4.282247899 | 0.00023164 | 0.002737606 | 0.346871558 |
| C6orf225 | -1.3984908 | 6.490335461 | -4.278389702 | 0.00023398 | 0.002761897 | 0.337150189 |
| CHST4 | 2.02594279 | 7.375910937 | 4.275875168 | 0.000235518 | 0.00277439 | 0.330815071 |
| IL9R | 1.18260482 | 6.875698139 | 4.275195589 | 0.000235935 | 0.002776989 | 0.329103028 |
| CCL20 | 2.64771099 | 10.57528375 | 4.26729054 | 0.000240845 | 0.002818804 | 0.309190963 |
| PRO0628 | 1.13552455 | 8.230527551 | 4.26550673 | 0.000241966 | 0.002828886 | 0.304698448 |

| | | | | | | |
|---|---|---|---|---|---|---|
| OLFML2B | 1.02465915 | 7.714790338 | 4.263206689 | 0.000243421 | 0.0028413 | 0.298906214 |
| PLAG1 | -1.572929 | 8.104522073 | -4.262057974 | 0.00024415 | 0.002845231 | 0.296013553 |
| SCAMP5 | -1.0891273 | 9.394913488 | -4.255789654 | 0.000248169 | 0.002878175 | 0.280230858 |
| TUBB4 | 1.15030825 | 5.835311154 | 4.253990457 | 0.000249334 | 0.00289015 | 0.275701379 |
| CHRNA1 | 1.2577965 | 4.910660489 | 4.252085213 | 0.000250575 | 0.002900139 | 0.270905235 |
| TRIM2 | -1.2980687 | 10.28091188 | -4.250366498 | 0.000251699 | 0.002910316 | 0.266578922 |
| ZNF512B | -1.3002159 | 9.061241628 | -4.250297936 | 0.000251744 | 0.002910316 | 0.266406343 |
| KCNMB4 | -1.3568532 | 7.985948623 | -4.248628652 | 0.00025284 | 0.002918337 | 0.262204714 |
| SCG5 | -2.3276932 | 8.059215718 | -4.245512381 | 0.0002549 | 0.002934323 | 0.25436164 |
| SIGLECP3 | -1.0383083 | 6.391938581 | -4.242306313 | 0.000257037 | 0.002955568 | 0.246293465 |
| C1QTNF4 | 1.71501412 | 7.037743637 | 4.234285465 | 0.000262461 | 0.003007025 | 0.226112761 |
| FDXR | 1.1241727 | 12.86244703 | 4.23389208 | 0.00026273 | 0.00300852 | 0.225123142 |
| DUSP5P | 1.6938745 | 5.516680696 | 4.232305298 | 0.000263817 | 0.00301462 | 0.221131493 |
| KLHDC7B | 1.36544737 | 8.015641143 | 4.23154558 | 0.00026434 | 0.003017671 | 0.219220454 |
| DSEL | -1.1221159 | 5.777955086 | -4.231512974 | 0.000264362 | 0.003017671 | 0.219138437 |
| CTSS | 1.37529272 | 9.159636448 | 4.227062813 | 0.000267442 | 0.003041091 | 0.207945321 |
| RAMP1 | -2.1354577 | 11.56063566 | -4.226671371 | 0.000267715 | 0.003041565 | 0.206960848 |
| KRTAP12.2 | 1.02697966 | 6.901597298 | 4.22317109 | 0.000270165 | 0.003059804 | 0.198158286 |
| C5AR1 | 1.27802157 | 7.763748592 | 4.222181321 | 0.000270862 | 0.003066099 | 0.195669404 |
| GGTLC2 | -1.2370949 | 8.745605037 | -4.2171777 | 0.000274412 | 0.003096609 | 0.183088643 |
| PYROXD2 | -1.287878 | 7.764440381 | -4.216702065 | 0.000274752 | 0.003098122 | 0.181892861 |
| CCDC21 | 1.94896055 | 7.862430269 | 4.214977125 | 0.000275988 | 0.003107898 | 0.177556408 |
| GBP2 | 1.04206282 | 11.20844835 | 4.214664826 | 0.000276212 | 0.003107898 | 0.176771325 |
| RBM24 | -1.2336342 | 4.016175242 | -4.211961978 | 0.000278162 | 0.003117878 | 0.169977076 |
| HOXA5 | -1.0183692 | 7.972522823 | -4.203714856 | 0.000284195 | 0.003166156 | 0.14925024 |
| TYROBP | 1.25445578 | 13.68251582 | 4.202547744 | 0.000285059 | 0.003172284 | 0.146317553 |
| PEG10 | -1.0269622 | 5.087216928 | -4.200454085 | 0.000286616 | 0.003181463 | 0.141056989 |
| VANGL2 | -1.0943173 | 5.608424728 | -4.19765989 | 0.000288707 | 0.003199769 | 0.134036902 |
| C15orf26 | 1.35456809 | 4.931388155 | 4.19578947 | 0.000290115 | 0.003210463 | 0.129338112 |
| PTK7 | -1.2193198 | 7.25354591 | -4.194960411 | 0.000290741 | 0.003213065 | 0.127255492 |
| OAS2 | 1.16136664 | 9.440748243 | 4.186207474 | 0.000297435 | 0.003263229 | 0.105271954 |
| TLR8 | 1.59826187 | 6.431881587 | 4.18295383 | 0.000299962 | 0.003285979 | 0.097102141 |
| CRTAC1 | -1.246435 | 5.432697052 | -4.18074126 | 0.000301693 | 0.00329663 | 0.091547033 |
| C2orf84 | -1.1655026 | 5.377226317 | -4.17977752 | 0.00030245 | 0.003303241 | 0.08912752 |
| LOC100131544 | 1.44350342 | 6.868173052 | 4.178644048 | 0.000303343 | 0.003311326 | 0.086282005 |

| | | | | | | |
|---|---|---|---|---|---|---|
| C1QA | 1.51530224 | 11.47784328 | 4.177746389 | 0.000304052 | 0.003315733 | 0.084028575 |
| CEP78 | 1.03750096 | 9.021589313 | 4.176768897 | 0.000304825 | 0.003322503 | 0.081574828 |
| hCG_1776018 | -1.7066965 | 5.569605305 | -4.176149146 | 0.000305317 | 0.003324526 | 0.080019149 |
| NOL3 | -1.0067389 | 10.94401615 | -4.175637305 | 0.000305723 | 0.003327286 | 0.07873437 |
| PRKAG2 | -1.1377043 | 9.952641138 | -4.170815425 | 0.00030958 | 0.003357489 | 0.066632205 |
| NFATC4 | -1.0741235 | 6.498914677 | -4.169531503 | 0.000310615 | 0.003367033 | 0.063410156 |
| EPOR | -1.1271944 | 6.953693564 | -4.167268931 | 0.000312447 | 0.00338015 | 0.057732554 |
| VPS13D | -1.1903701 | 7.153058886 | -4.16660258 | 0.000312989 | 0.003380961 | 0.056060539 |
| MPHOSPH6 | 1.03082533 | 8.293414182 | 4.165367499 | 0.000313995 | 0.003390147 | 0.052961582 |
| PLOD2 | -1.2603699 | 9.767785291 | -4.16491519 | 0.000314364 | 0.0033909 | 0.051826726 |
| GBE1 | -1.1437622 | 9.640768729 | -4.155032709 | 0.000322543 | 0.003458383 | 0.02703654 |
| MCOLN3 | -1.0065838 | 4.883349302 | -4.150620728 | 0.000326262 | 0.003486618 | 0.015972345 |
| CLDN16 | 1.00993759 | 5.323187893 | 4.148402885 | 0.000328147 | 0.003501201 | 0.010411289 |
| TMEM151B | -1.0959728 | 7.852321291 | -4.145038787 | 0.000331028 | 0.003518199 | 0.001977076 |
| NKX2.2 | -1.0531592 | 4.411676961 | -4.141536061 | 0.000334053 | 0.003543346 | -0.006803428 |
| PXMP4 | -1.0835555 | 8.781413717 | -4.138891277 | 0.000336356 | 0.003562556 | -0.013432418 |
| RHBDF2 | 1.07779371 | 11.72950772 | 4.138483484 | 0.000336713 | 0.00356298 | -0.014454462 |
| SPRR2C | 1.66889091 | 6.771988459 | 4.136632078 | 0.000338336 | 0.003571339 | -0.019094376 |
| CDH16 | 2.94628917 | 6.95209617 | 4.13431281 | 0.00034038 | 0.003583005 | -0.024906312 |
| MAPK10 | -1.1159228 | 5.638407981 | -4.133681412 | 0.000340938 | 0.00358662 | -0.026488453 |
| KRTAP4.2 | 1.8099639 | 6.456383428 | 4.132325291 | 0.000342141 | 0.003597532 | -0.029886447 |
| P2RX7 | 1.38238627 | 6.760309993 | 4.131369386 | 0.000342992 | 0.003602988 | -0.032281513 |
| C1QB | 1.58869764 | 13.87728887 | 4.125034089 | 0.000348681 | 0.0036504 | -0.04815241 |
| TNFRSF9 | 1.67659382 | 6.868158597 | 4.121815335 | 0.000351607 | 0.003670427 | -0.056214224 |
| C1orf194 | -1.3195876 | 5.659995858 | -4.120972358 | 0.000352377 | 0.003674938 | -0.058325388 |
| MYEF2 | -1.3598657 | 5.849374224 | -4.117454469 | 0.00035561 | 0.00370155 | -0.067134808 |
| C4BPB | -2.2813273 | 7.914648894 | -4.114122576 | 0.000358699 | 0.003717679 | -0.075477209 |
| ENST00000424686 | 1.33480326 | 13.82658109 | 4.111841339 | 0.00036083 | 0.003732638 | -0.08118827 |
| KLK12 | -2.187081 | 6.107877312 | -4.108052501 | 0.000364395 | 0.00375879 | -0.090672325 |
| GALNT14 | -1.9466049 | 7.660154998 | -4.105593234 | 0.000366729 | 0.003781062 | -0.096827402 |
| ENST00000329043 | 1.46649777 | 5.870438025 | 4.104595123 | 0.00036768 | 0.003787275 | -0.099325291 |
| DEPDC7 | -1.4771583 | 6.252303811 | -4.098340462 | 0.000373696 | 0.003832223 | -0.114975761 |
| LAMC2 | 1.74194369 | 10.36671388 | 4.092762449 | 0.000379143 | 0.003877785 | -0.12892939 |
| CDH10 | -1.259683 | 4.962738143 | -4.090041326 | 0.000381829 | 0.003890607 | -0.135735106 |
| RTN4RL1 | -1.2746465 | 6.607071115 | -4.089679302 | 0.000382187 | 0.003890614 | -0.136640487 |

| | | | | | | |
|---|---|---|---|---|---|---|
| C2CD4A | 1.75869473 | 8.098328159 | 4.088223385 | 0.000383633 | 0.003903506 | -0.140281429 |
| KLHDC9 | -1.1172523 | 9.065410469 | -4.085717622 | 0.000386135 | 0.003923668 | -0.146547244 |
| MIPOL1 | -1.5300677 | 6.200787523 | -4.083960976 | 0.000387898 | 0.003937683 | -0.150939411 |
| PNPLA1 | -1.4488448 | 4.816730019 | -4.079795612 | 0.000392111 | 0.003973027 | -0.161352714 |
| OPALIN | 1.25635322 | 5.068551813 | 4.065220009 | 0.000407212 | 0.004091702 | -0.197775388 |
| PTCRA | 1.41187469 | 6.885028156 | 4.063541159 | 0.000408988 | 0.004107647 | -0.201969022 |
| C10orf116 | -3.1390927 | 12.61080206 | -4.062681905 | 0.0004099 | 0.004114904 | -0.20411524 |
| IFIT3 | 1.15291454 | 10.67509488 | 4.058425965 | 0.000414446 | 0.004147135 | -0.214744302 |
| JAG1 | 1.38554585 | 10.34243303 | 4.052719648 | 0.00042062 | 0.004191545 | -0.228992216 |
| GNLY | 1.6146318 | 9.403897006 | 4.049109557 | 0.000424573 | 0.00422319 | -0.238004105 |
| ENST00000380640 | 1.12813273 | 5.349789184 | 4.048235748 | 0.000425535 | 0.00422889 | -0.240185159 |
| MAPRE3 | -1.0227484 | 5.536131052 | -4.035356194 | 0.000439971 | 0.004346513 | -0.272322109 |
| SLC7A4 | -1.7820584 | 6.29132867 | -4.034053284 | 0.000441458 | 0.004357242 | -0.275571981 |
| LOC388948 | 1.03840279 | 6.378587232 | 4.029940483 | 0.000446184 | 0.004391926 | -0.285829227 |
| SLC15A3 | 1.08038757 | 11.49974311 | 4.027883441 | 0.000448567 | 0.004411384 | -0.290958658 |
| VASH1 | 1.04308939 | 11.04749936 | 4.027453717 | 0.000449067 | 0.004414297 | -0.292030149 |
| NAP1L2 | -1.2714183 | 5.175342992 | -4.026512602 | 0.000450162 | 0.004421067 | -0.294376681 |
| VEPH1 | -1.1539596 | 6.295048217 | -4.025123837 | 0.000451783 | 0.004432985 | -0.297839163 |
| C10orf58 | -1.4222381 | 9.576939813 | -4.024559629 | 0.000452444 | 0.004437461 | -0.299245781 |
| OR10P1 | 1.03533738 | 5.912141391 | 4.021006811 | 0.000456624 | 0.004469759 | -0.308102339 |
| HRH3 | 1.13283127 | 6.790423886 | 4.014515731 | 0.00046436 | 0.004535914 | -0.324279343 |
| BMP4 | -1.6430131 | 7.654617612 | -4.012153786 | 0.000467207 | 0.004555534 | -0.330164426 |
| A_33_P3327300 | 1.16009681 | 5.478584954 | 4.011524449 | 0.000467969 | 0.004560913 | -0.331732379 |
| MACC1 | 1.00294538 | 8.634373168 | 4.008267847 | 0.000471929 | 0.004591273 | -0.339845179 |
| CNR1 | -2.0128191 | 6.178409966 | -4.006638281 | 0.000473923 | 0.004606547 | -0.343904218 |
| CD68 | 1.11707256 | 11.56706587 | 4.004360814 | 0.000476724 | 0.004631699 | -0.349576521 |
| CD74 | 1.25778183 | 11.24081861 | 3.997924789 | 0.000484727 | 0.004682245 | -0.365602573 |
| CLCF1 | 1.16453793 | 7.495321505 | 3.995178935 | 0.000488182 | 0.004707121 | -0.372438256 |
| LOC339751 | 2.3469019 | 6.47235571 | 3.995037815 | 0.00048836 | 0.004707121 | -0.372789541 |
| SGCG | -1.1746692 | 4.73769451 | -3.993612058 | 0.000490164 | 0.00472094 | -0.376338484 |
| LOC100130905 | 1.54163769 | 7.458460623 | 3.986895176 | 0.000498752 | 0.004787478 | -0.393054305 |
| PACSIN1 | -1.7833718 | 7.977142449 | -3.98397613 | 0.00050253 | 0.004806433 | -0.400316864 |
| LRTM2 | -1.0193914 | 4.223000216 | -3.982653127 | 0.000504252 | 0.004814541 | -0.403608109 |
| GRK4 | -1.0062463 | 5.65718344 | -3.98213784 | 0.000504924 | 0.00481739 | -0.40488993 |
| SIGLEC7 | 1.02759834 | 6.429800192 | 3.978441769 | 0.000509771 | 0.004855111 | -0.414083185 |

| | | | | | | |
|---|---|---|---|---|---|---|
| LOC646548 | -1.0411597 | 4.936472348 | -3.974964823 | 0.000514373 | 0.004892572 | -0.422729739 |
| NOV | -1.5800706 | 7.810647835 | -3.974855053 | 0.000514519 | 0.004892572 | -0.423002691 |
| CCDC148 | 1.06463984 | 5.277359057 | 3.974264546 | 0.000515304 | 0.00489562 | -0.424471007 |
| FAM26F | 1.6532043 | 10.65246034 | 3.974210982 | 0.000515376 | 0.00489562 | -0.424604193 |
| A_33_P3226439 | -1.1078335 | 8.292705655 | -3.968992705 | 0.000522373 | 0.004940965 | -0.437577539 |
| WNK2 | -1.4491184 | 7.949028633 | -3.968955508 | 0.000522423 | 0.004940965 | -0.437670002 |
| TSPAN7 | -1.8586788 | 7.44491944 | -3.963048491 | 0.000530457 | 0.005001715 | -0.452351155 |
| SMTNL2 | -1.0244375 | 4.353753592 | -3.954976696 | 0.000541633 | 0.005085031 | -0.472404862 |
| SLMO1 | 1.20742212 | 7.914834501 | 3.95425423 | 0.000542644 | 0.005090602 | -0.474199333 |
| A_33_P3364869 | 1.09523108 | 10.10932566 | 3.954051384 | 0.000542929 | 0.005090602 | -0.474703151 |
| PTPN13 | -1.3922154 | 7.540168795 | -3.953602004 | 0.000543559 | 0.005092118 | -0.475819276 |
| KCNH6 | -1.0813876 | 5.360236361 | -3.950857436 | 0.000547425 | 0.005117307 | -0.482635356 |
| NCRNA00164 | 1.23436328 | 6.907141339 | 3.950204125 | 0.000548349 | 0.005119549 | -0.484257688 |
| HCK | 1.15399681 | 8.385398837 | 3.950188411 | 0.000548372 | 0.005119549 | -0.484296707 |
| ENST00000342354 | 1.12122367 | 5.249606401 | 3.948432354 | 0.000550864 | 0.005136197 | -0.488657128 |
| FOXF2 | -1.3341974 | 7.3792076 | -3.94446723 | 0.000556532 | 0.005169816 | -0.49850124 |
| ZNF256 | -1.0413882 | 7.5628118 | -3.944412722 | 0.00055661 | 0.005169816 | -0.498636551 |
| PCDH7 | 1.73101347 | 8.78417074 | 3.942845779 | 0.000558866 | 0.005176842 | -0.502526141 |
| GPR4 | 1.05212684 | 7.749435919 | 3.94282384 | 0.000558898 | 0.005176842 | -0.502580597 |
| GPM6B | -1.2734482 | 6.1187314 | -3.940234131 | 0.000562647 | 0.005201432 | -0.509008214 |
| BDH1 | -1.9868415 | 10.39234775 | -3.939396289 | 0.000563865 | 0.005203839 | -0.51108752 |
| ADAMDEC1 | 2.09535466 | 7.679347866 | 3.938271453 | 0.000565504 | 0.005216754 | -0.513878919 |
| PLA2G2A | 3.8809909 | 9.155415137 | 3.935133505 | 0.000570103 | 0.005245537 | -0.521665115 |
| ME1 | -1.8196574 | 7.944717949 | -3.933513294 | 0.000572491 | 0.005261111 | -0.525684803 |
| NEFM | -1.050037 | 4.830362869 | -3.932804144 | 0.00057354 | 0.00526852 | -0.527444061 |
| HAND1 | 1.03652942 | 4.69093792 | 3.931487578 | 0.000575492 | 0.005282972 | -0.530710006 |
| SLC46A1 | -1.7501532 | 8.100640694 | -3.924989377 | 0.000585222 | 0.005355455 | -0.546826168 |
| FAM115C | 1.19028897 | 6.76908884 | 3.923864887 | 0.000586922 | 0.005366494 | -0.549614394 |
| MAPK4 | -1.0146022 | 4.680765407 | -3.916881995 | 0.000597588 | 0.005434303 | -0.566924727 |
| DNAH14 | -1.2546031 | 6.236324091 | -3.914789401 | 0.000600822 | 0.005450026 | -0.572110812 |
| AGTR1 | -1.4347169 | 4.662890187 | -3.914529452 | 0.000601225 | 0.005451405 | -0.572755001 |
| TRIMP1 | 1.19544443 | 6.546530358 | 3.907382005 | 0.000612407 | 0.005529727 | -0.590463458 |
| ATP6V0D2 | 1.33005346 | 5.494232025 | 3.905506583 | 0.000615375 | 0.00554898 | -0.595108747 |
| FLJ31183 | -1.251826 | 5.353514968 | -3.905389592 | 0.00061556 | 0.00554898 | -0.595398508 |
| C2orf61 | 1.11618253 | 5.66203377 | 3.90512312 | 0.000615983 | 0.005550491 | -0.596058493 |

| | | | | | | |
|---|---|---|---|---|---|---|
| ProSAPiP1 | -1.515859 | 8.388236043 | -3.900980865 | 0.000622595 | 0.005584601 | -0.606316492 |
| ZBED2 | 1.56352553 | 6.795106758 | 3.899782026 | 0.000624522 | 0.005594955 | -0.609284858 |
| HMGA2 | 1.0152836 | 7.548397179 | 3.897313534 | 0.000628507 | 0.005621392 | -0.615396253 |
| HLA.DMB | 1.21353417 | 13.88089179 | 3.892155476 | 0.000636916 | 0.005675581 | -0.628163433 |
| BHLHE40 | 1.18178638 | 9.722447411 | 3.890409174 | 0.000639788 | 0.005696502 | -0.632484961 |
| C16orf90 | 1.06678077 | 6.063664368 | 3.888629422 | 0.000642728 | 0.005715655 | -0.636888797 |
| TSPAN5 | -1.4348023 | 9.025819608 | -3.881921005 | 0.00065393 | 0.005789216 | -0.653483869 |
| SLC44A4 | -2.8237325 | 10.607906 | -3.881744311 | 0.000654228 | 0.005789492 | -0.653920875 |
| SOD3 | -1.5013837 | 13.24033568 | -3.880395261 | 0.000656505 | 0.005804914 | -0.657257255 |
| DAG1 | 1.03385602 | 10.4613658 | 3.879760238 | 0.000657579 | 0.005809687 | -0.658827653 |
| LRRC25 | 1.07206716 | 8.226920781 | 3.876921518 | 0.000662404 | 0.005835703 | -0.665846998 |
| IL27 | 1.01477559 | 6.84586925 | 3.869744866 | 0.000674756 | 0.005925309 | -0.68358731 |
| C17orf66 | 1.29582524 | 5.337983035 | 3.864687803 | 0.000683595 | 0.005983593 | -0.696083342 |
| AOX1 | -1.7131387 | 6.803556989 | -3.861533043 | 0.000689168 | 0.006022665 | -0.703876761 |
| PLCXD1 | -1.0281639 | 9.075800869 | -3.860178141 | 0.000691574 | 0.006034462 | -0.707223394 |
| RAB31 | 1.06439307 | 10.58128851 | 3.857507534 | 0.000696343 | 0.006063425 | -0.713819001 |
| LPA | 1.14619792 | 5.782960142 | 3.855601961 | 0.000699765 | 0.006083474 | -0.718524519 |
| OR4N4 | -1.0576067 | 5.296983842 | -3.853976353 | 0.000702697 | 0.006099109 | -0.722538256 |
| FGF14 | -1.2448954 | 5.070952576 | -3.852034961 | 0.000706215 | 0.006117526 | -0.727331141 |
| TCEAL7 | -1.3145267 | 6.163104829 | -3.847521724 | 0.00071446 | 0.006161023 | -0.738471067 |
| CKLF | 1.12618202 | 10.28558 | 3.847215465 | 0.000715023 | 0.006161897 | -0.739226882 |
| PCDHA5 | 1.55602378 | 6.418658621 | 3.842671073 | 0.000723427 | 0.006213918 | -0.750440226 |
| GPR113 | 1.05947642 | 5.323320488 | 3.842560561 | 0.000723632 | 0.006213918 | -0.750712873 |
| ENST00000304890 | 1.1696689 | 6.033252322 | 3.842164202 | 0.00072437 | 0.006217035 | -0.751690732 |
| SSBP2 | -1.0504963 | 8.791165628 | -3.840866558 | 0.000726791 | 0.006225169 | -0.754891975 |
| XAGE1D | -1.1826521 | 4.744540303 | -3.840629476 | 0.000727234 | 0.006225169 | -0.755476819 |
| LOC731223 | 1.02924875 | 7.160892249 | 3.840278168 | 0.000727891 | 0.006225889 | -0.756343424 |
| NAT8B | -1.6199104 | 7.067054563 | -3.834610428 | 0.000738573 | 0.006294086 | -0.770321854 |
| VIPR1 | -1.9275946 | 7.174412767 | -3.834407521 | 0.000738958 | 0.006294086 | -0.77082219 |
| SLC1A7 | -1.7689708 | 7.668422967 | -3.833971495 | 0.000739787 | 0.006294424 | -0.771897339 |
| PTPN21 | -1.0337221 | 8.518856211 | -3.832464728 | 0.000742657 | 0.006301398 | -0.775612477 |
| SGCE | -1.6085143 | 9.503169772 | -3.827690385 | 0.000751825 | 0.006355565 | -0.787381842 |
| FAM174A | -1.4511147 | 8.765730219 | -3.825500506 | 0.000756067 | 0.006380334 | -0.792778937 |
| TMEM150C | -1.2351031 | 6.298618354 | -3.822681623 | 0.000761562 | 0.006420396 | -0.799725101 |
| C9orf98 | 1.42108695 | 7.962427401 | 3.812245946 | 0.000782248 | 0.006554118 | -0.825428905 |

| | | | | | | |
|---|---|---|---|---|---|---|
| KHNYN | -1.0809999 | 11.46793492 | -3.811616336 | 0.000783514 | 0.006558938 | -0.826979106 |
| LONRF3 | -1.1829307 | 7.813013722 | -3.811539222 | 0.000783669 | 0.006558938 | -0.827168966 |
| LOC100293193 | 1.41660058 | 9.152202656 | 3.80909018 | 0.000788612 | 0.006592188 | -0.833198243 |
| HPDL | 1.941191 | 10.67361866 | 3.808165876 | 0.000790485 | 0.00660277 | -0.835473519 |
| CYBB | 1.12258977 | 11.13862669 | 3.805948828 | 0.000794997 | 0.006629146 | -0.840930452 |
| ALDOC | -1.582635 | 7.873323478 | -3.803778479 | 0.000799438 | 0.006639826 | -0.846271648 |
| CACNA1A | -1.1045376 | 7.645434927 | -3.802170352 | 0.000802744 | 0.006648292 | -0.850228718 |
| IGSF10 | -1.3069482 | 4.917585602 | -3.79945915 | 0.000808349 | 0.006687726 | -0.856899113 |
| SLC16A10 | -1.4733973 | 5.858949183 | -3.798256039 | 0.000810848 | 0.006703301 | -0.859858742 |
| FBXO27 | -1.1439829 | 5.885054283 | -3.789144846 | 0.000830024 | 0.006815168 | -0.882264178 |
| THY1 | 1.52818666 | 12.00082196 | 3.781471548 | 0.000846519 | 0.006896297 | -0.901122738 |
| HRCT1 | -1.5502048 | 6.958257878 | -3.781448682 | 0.000846569 | 0.006896297 | -0.901178922 |
| PCNX | 1.09943792 | 10.18251287 | 3.779345565 | 0.000851146 | 0.006926305 | -0.906345957 |
| SIGLEC1 | 1.26447105 | 6.86823207 | 3.778220501 | 0.000853604 | 0.006930231 | -0.909109755 |
| HLA.DRA | 1.23012718 | 14.34206922 | 3.776038824 | 0.000858391 | 0.006953513 | -0.914468577 |
| SPRR1B | 1.71914855 | 6.20193236 | 3.77545264 | 0.000859682 | 0.006961374 | -0.915908272 |
| CIDEC | -1.9639119 | 9.114733131 | -3.77520245 | 0.000860233 | 0.006961928 | -0.916522731 |
| MRAP | -1.3357467 | 4.930153101 | -3.772119221 | 0.000867058 | 0.007002179 | -0.924094177 |
| HS3ST2 | 1.96029977 | 6.189815772 | 3.766978546 | 0.000878555 | 0.007073504 | -0.936714399 |
| CRYBG3 | -1.0251863 | 9.235303374 | -3.766898069 | 0.000878737 | 0.007073504 | -0.936911932 |
| F2R | 1.21528758 | 8.148148057 | 3.765288296 | 0.000882368 | 0.007094854 | -0.940862915 |
| TMEM217 | 1.02178024 | 6.105870077 | 3.761291821 | 0.000891448 | 0.007154629 | -0.950669798 |
| LOC100131733 | 1.02590201 | 11.18813022 | 3.760659912 | 0.000892892 | 0.00716093 | -0.952220172 |
| DHCR24 | -1.2007748 | 11.60723675 | -3.759626479 | 0.000895259 | 0.007172801 | -0.954755526 |
| TAP2 | 1.12708141 | 9.217988787 | 3.759581227 | 0.000895363 | 0.007172801 | -0.954866542 |
| NGFRAP1 | -1.5932743 | 12.51944088 | -3.758749546 | 0.000897272 | 0.007185448 | -0.956906787 |
| PRKAB1 | -1.0302907 | 8.880493683 | -3.755524984 | 0.000904712 | 0.007229049 | -0.964816003 |
| ROR2 | -1.0830944 | 6.499230279 | -3.755224161 | 0.000905409 | 0.007229304 | -0.965553769 |
| ZMYND12 | -1.0676693 | 7.386163283 | -3.752231287 | 0.000912374 | 0.007260707 | -0.972892898 |
| JAK2 | 1.20411902 | 9.93634792 | 3.751429934 | 0.000914248 | 0.007265181 | -0.974857708 |
| FAM110C | -1.6157056 | 7.53566753 | -3.743674896 | 0.000932578 | 0.007370419 | -0.993866117 |
| CCDC85A | -1.5963058 | 5.535536512 | -3.741431249 | 0.000937948 | 0.007402093 | -0.99936353 |
| HMOX1 | 1.18405299 | 12.10307602 | 3.7408561 | 0.00093933 | 0.007410304 | -1.000772621 |
| PVALB | -1.0793151 | 6.22597753 | -3.740252702 | 0.000940781 | 0.007419062 | -1.00225086 |
| PAQR5 | -2.2212742 | 7.816706411 | -3.738213953 | 0.000945701 | 0.007449755 | -1.007245012 |

| | | | | | | |
|---|---|---|---|---|---|---|
| SLC16A6 | 1.05713831 | 6.511233969 | 3.731893962 | 0.000961115 | 0.007543834 | -1.022721807 |
| C7orf29 | 1.05347735 | 10.36081035 | 3.722000939 | 0.000985739 | 0.007700953 | -1.046933916 |
| C3orf43 | 1.19597554 | 5.669343834 | 3.71928437 | 0.000992608 | 0.007743483 | -1.053579296 |
| NPDC1 | -1.6092411 | 13.21153032 | -3.717083625 | 0.000998207 | 0.007775998 | -1.058961853 |
| RASSF4 | 1.05423267 | 10.36240451 | 3.716373086 | 0.001000021 | 0.007784551 | -1.060699489 |
| ABAT | -1.1018624 | 9.933431437 | -3.708420088 | 0.001020551 | 0.007913185 | -1.080142341 |
| SRPK3 | -1.0792009 | 6.524410381 | -3.706013662 | 0.001026844 | 0.007952726 | -1.086023063 |
| LOC100133920 | 1.0385383 | 5.163139808 | 3.705925003 | 0.001027077 | 0.007952726 | -1.086239703 |
| PLXDC2 | 1.21137198 | 8.734242993 | 3.705216927 | 0.001028936 | 0.007960639 | -1.087969853 |
| LOC100130274 | 1.08264693 | 6.969638039 | 3.705103038 | 0.001029235 | 0.007960639 | -1.088248126 |
| DYSF | 1.16030581 | 7.708262912 | 3.704857155 | 0.001029882 | 0.007962807 | -1.088848903 |
| CLEC4A | 1.11946724 | 8.71715021 | 3.703532943 | 0.001033371 | 0.007981266 | -1.09208421 |
| SYPL2 | -1.111628 | 5.786965781 | -3.70256961 | 0.001035916 | 0.007995244 | -1.094437614 |
| FAM117A | -1.2107346 | 10.56131877 | -3.701749964 | 0.001038087 | 0.008006311 | -1.096439855 |
| FYB | 1.21379893 | 9.992919699 | 3.69783915 | 0.001048506 | 0.008069483 | -1.105991512 |
| IDO2 | 1.39131457 | 5.775853616 | 3.696636119 | 0.001051731 | 0.008084027 | -1.10892918 |
| TBCEL | -1.0454327 | 7.934379768 | -3.688562585 | 0.00107363 | 0.008196008 | -1.128636785 |
| TMEM132C | -1.3054683 | 4.880498384 | -3.685985412 | 0.001080714 | 0.008229933 | -1.134925098 |
| CDCA7L | -1.042332 | 8.511408637 | -3.68387444 | 0.00108655 | 0.008257021 | -1.140074933 |
| KRT6A | 1.45961219 | 5.379500544 | 3.670526828 | 0.001124175 | 0.008504288 | -1.172617402 |
| ZIM2 | 1.0020294 | 5.516586171 | 3.66916362 | 0.001128089 | 0.008527958 | -1.175939071 |
| NR2E1 | -1.0378788 | 4.835760344 | -3.662683534 | 0.001146876 | 0.008645922 | -1.191723867 |
| PRKD1 | -1.1366348 | 6.051601851 | -3.662520627 | 0.001147352 | 0.008646513 | -1.192120584 |
| MYOM2 | -1.2360865 | 6.983232526 | -3.661070458 | 0.0011516 | 0.008666504 | -1.195651874 |
| SLC16A9 | -1.7971885 | 6.699465901 | -3.658688278 | 0.001158611 | 0.008713233 | -1.201451797 |
| HLA.DPA1 | 1.28814459 | 14.91883863 | 3.656401362 | 0.001165381 | 0.008752034 | -1.207018733 |
| LY6K | -1.7060908 | 6.494856414 | -3.649287476 | 0.001186689 | 0.008875255 | -1.224329151 |
| MEP1B | -2.5627738 | 5.575691243 | -3.647275244 | 0.001192785 | 0.008917778 | -1.229223756 |
| CHIT1 | 1.11131097 | 8.921037761 | 3.645875234 | 0.001197044 | 0.008936552 | -1.232628701 |
| TM6SF2 | -1.4946508 | 6.953264176 | -3.641780283 | 0.001209588 | 0.009000061 | -1.242585738 |
| PHGR1 | -4.2132998 | 13.75845205 | -3.636704417 | 0.001225315 | 0.009085974 | -1.254923256 |
| ZDBF2 | -1.5518401 | 6.199460901 | -3.631862671 | 0.001240502 | 0.009182929 | -1.266686897 |
| NTS | -1.648361 | 5.250508566 | -3.628919187 | 0.001249826 | 0.009236215 | -1.273836156 |
| FMO1 | 1.43384619 | 6.93233298 | 3.627652624 | 0.001253858 | 0.00925029 | -1.276911899 |
| OR6K2 | 1.38491766 | 6.418587607 | 3.622115584 | 0.001271638 | 0.009333932 | -1.290354315 |

| | | | | | | |
|---|---|---|---|---|---|---|
| LOC642666 | 1.89043732 | 5.60632323 | 3.614896212 | 0.00129519 | 0.009471137 | -1.307871582 |
| PLP1 | -1.4012738 | 5.743695019 | -3.612961644 | 0.001301573 | 0.009502312 | -1.312563849 |
| TOM1L1 | -1.098412 | 10.28967019 | -3.611885913 | 0.001305135 | 0.009520477 | -1.315172687 |
| SCGB1D2 | -1.1734048 | 4.562290111 | -3.611813682 | 0.001305375 | 0.009520477 | -1.315347852 |
| DAPK2 | -1.2506035 | 8.930777141 | -3.61156196 | 0.00130621 | 0.009523372 | -1.315958284 |
| F8 | 1.5181314 | 7.087376941 | 3.611010981 | 0.00130804 | 0.009533516 | -1.317294376 |
| MMAB | -1.2037863 | 7.139212012 | -3.610719268 | 0.00130901 | 0.0095342 | -1.318001738 |
| HEYL | 1.43706451 | 8.539297526 | 3.607123781 | 0.001321022 | 0.009597199 | -1.326718831 |
| LOC100134937 | -1.1450754 | 7.279146956 | -3.60516643 | 0.001327606 | 0.00963734 | -1.331463214 |
| C11orf53 | -2.0448545 | 7.771513112 | -3.60073963 | 0.001342616 | 0.009725766 | -1.34219031 |
| CBX6 | -1.1920917 | 11.63439669 | -3.600388124 | 0.001343815 | 0.009725766 | -1.343041912 |
| SULT1A2 | -1.233947 | 11.70119509 | -3.599821398 | 0.00134575 | 0.009730052 | -1.344414876 |
| LOC389332 | -2.8008394 | 6.884432191 | -3.59839257 | 0.001350641 | 0.009758829 | -1.347876091 |
| TIGIT | 1.27685937 | 7.428615508 | 3.596872606 | 0.001355863 | 0.009781146 | -1.351557607 |
| G0S2 | -1.889152 | 11.4478232 | -3.596249639 | 0.001358009 | 0.009789379 | -1.353066362 |
| KRTAP10.9 | 1.01318652 | 6.944900103 | 3.594127931 | 0.001365343 | 0.009826874 | -1.358204282 |
| CCL18 | 2.38013803 | 12.43819871 | 3.593275938 | 0.001368299 | 0.00983745 | -1.360267197 |
| MGC13005 | 1.56526181 | 7.381980102 | 3.589161059 | 0.001382663 | 0.009911213 | -1.370228341 |
| CGREF1 | -2.3819307 | 7.231196691 | -3.586555767 | 0.001391833 | 0.009963803 | -1.376533288 |
| TMPRSS4 | 1.82434519 | 10.44591486 | 3.585476241 | 0.001395651 | 0.00998784 | -1.379145378 |

**Supplementary table 2. The relationship between module expression and clinical characteristics of GC patients**

| Variable | CCGs module expression | | | | | | $P$ [a] |
|---|---|---|---|---|---|---|---|
| | Low (N=100) | | Medium (N=100) | | High (N=100) | | |
| | No. | % | No. | % | No. | % | |
| Age | | | | | | | 0.157 |
| <60 | 29 | 27.36 | 42 | 39.62 | 35 | 33.02 | |
| ≥60 | 71 | 36.60 | 58 | 29.90 | 65 | 33.51 | |
| Sex | | | | | | | 0.788 |
| Female | 31 | 30.69 | 35 | 34.65 | 35 | 34.65 | |
| Male | 69 | 34.67 | 65 | 32.66 | 65 | 32.66 | |
| **EBV status** | | | | | | | **1.105e-06** |
| Positive | 0 | 0.00 | 2 | 11.11 | 16 | 88.88 | |
| Negative | 91 | 35.41 | 91 | 35.41 | 75 | 29.18 | |
| T stage | | | | | | | 0.163 |
| 2 | 68 | 36.56 | 61 | 32.80 | 57 | 30.65 | |
| 3 | 21 | 23.08 | 34 | 37.36 | 36 | 39.56 | |
| 4 | 9 | 42.86 | 5 | 23.81 | 7 | 33.33 | |
| N stage | | | | | | | 0.379 |
| 0 | 12 | 31.58 | 9 | 23.68 | 17 | 44.74 | |
| 1 | 48 | 36.64 | 48 | 36.64 | 35 | 26.72 | |
| 2 | 23 | 28.75 | 28 | 35.00 | 29 | 36.25 | |
| 3 | 17 | 33.33 | 15 | 29.41 | 19 | 37.25 | |
| **M stage** | | | | | | | **0.033** [b] |
| 0 | 97 | 35.53 | 89 | 32.60 | 87 | 31.87 | |
| 1 | 3 | 11.11 | 11 | 40.74 | 13 | 48.15 | |
| **Lauren** | | | | | | | **0.0002** [b] |
| Intestinal | 66 | 45.21 | 41 | 28.08 | 39 | 26.71 | |
| Diffuse | 27 | 20.15 | 54 | 40.30 | 53 | 39.55 | |
| Mixed | 7 | 41.18 | 3 | 17.65 | 7 | 41.18 | |
| Indeterminate | 0 | 0.00 | 1 | 50.00 | 1 | 50.00 | |

**Abbreviations**: CCG, consistent co-expression genes; Lauren, Lauren classification; GC, gastric cancer.

**Note**: [a] $P<0.05$ was considered as significant; and $0.1>P>0.05$ was considered as boundary significant.

[b] Fisher's exact test.

The statistically significant results were marked in **bold**.

**Supplementary table 3. The impact of mRNA expression levels of module or *SLAMF8* on GC patients' survival**

| **SLAMF8 expression** | **Univariate analysis** | | **Multivariate analysis** | | **CCGs module expression** | **Univariate analysis** | | **Multivariate analysis** [b] | |
|---|---|---|---|---|---|---|---|---|---|
| | **HR (95%CI)** | ***P*** | **HR (95%CI)** | ***P*** | | **HR (95%CI)** | ***P*** [a] | **HR (95%CI)** | ***P*** |
| Low | 1.00 (Reference) | | 1.00 (Reference) | Low | Low | 1.00 (Reference) | | 1.00 (Reference) | |
| Medium | 0.80 (0.66-0.98) | 0.028 | 0.68 (0.45-1.05) | 0.080 | Medium | 0.96 (0.66-1.40) | 0.825 | 0.80 (0.52-1.25) | 0.330 |
| **High** | **0.64 (0.43-0.95)** | **0.027** | **0.50 (0.31-0.79)** | **0.004** | **High** | **0.74 (0.49-1.10)** | **0.131** | **0.56 (0.35-0.92)** | **0.021** |
| **Trend** | | | | **0.003** | | | | | **0.018** |

**Abbreviation:** GC, gastric cancer; CCG, consistent co-expression genes

**Note**: [a] All *P* value were calculated from Cox proportional hazards regression analysis. $P<0.05$ was considered as significant; and $0.1>P>0.05$ was considered as boundary significant.

[b] Adjust for age, sex, Lauren classification, pathologic TNM stage, EBV status and adjuvant treatment.

The statistically significant results were marked in **bold**.

**Supplementary table 4: High-expression SLAMF8 GI cancers may enrich the pathways involved in antitumor immune response.**

**High-expression *SLAMF8* vs. low-expression *SLAMF8* gastric cancer tissues (GSE51575)**

| NAME | SIZE | ES | NES | NOM.p.val | FDR.q.val | FWER.p.val | RANK.AT.MAX | LEADING.EDGE |
|---|---|---|---|---|---|---|---|---|
| GO_DOUBLE_STRANDED_RNA_BINDING | 67 | 0.467236 | 1.672284 | 0.028736 | 0.168964 | 0.927 | 7431 | tags=60%, list=34%, signal=90% |
| GO_T_CELL_RECEPTOR_SIGNALING_PATHWAY | 171 | 0.535854 | 1.657666 | 0.041016 | 0.14619 | 0.942 | 4947 | tags=40%, list=23%, signal=52% |
| GO_ANTIGEN_RECEPTOR_MEDIATED_SIGNALING_PATHWAY | 212 | 0.583773 | 1.662842 | 0.038462 | 0.152928 | 0.939 | 2988 | tags=34%, list=14%, signal=40% |
| GO_NEGATIVE_REGULATION_OF_TYPE_I_INTERFERON_PRODUCTION | 41 | 0.643962 | 1.736948 | 0.003914 | 0.228686 | 0.819 | 3914 | tags=44%, list=18%, signal=53% |
| GO_IMMUNE_RESPONSE_REGULATING_CELL_SURFACE_RECEPTOR_SIGNALING_PATHWAY | 349 | 0.552287 | 1.644021 | 0.037624 | 0.137786 | 0.951 | 2988 | tags=33%, list=14%, signal=38% |
| GO_FC_RECEPTOR_SIGNALING_PATHWAY | 172 | 0.447083 | 1.619786 | 0.045726 | 0.13758 | 0.967 | 4956 | tags=35%, list=23%, signal=45% |
| GO_NEGATIVE_REGULATION_OF_ANTIGEN_RECEPTOR_MEDIATED_SIGNALING_PATHWAY | 27 | 0.762306 | 1.675931 | 0.005639 | 0.178565 | 0.925 | 1275 | tags=41%, list=6%, signal=43% |
| GO_REGULATION_OF_DEFENSE_RESPONSE_TO_VIRUS | 65 | 0.650576 | 1.791706 | 0 | 0.387076 | 0.679 | 5323 | tags=52%, list=24%, signal=69% |
| GO_ALPHA_BETA_T_CELL_DIFFERENTIATION | 97 | 0.636863 | 1.666487 | 0.020677 | 0.153047 | 0.937 | 2951 | tags=49%, list=14%, signal=57% |
| GO_REGULATION_OF_T_CELL_RECEPTOR_SIGNALING_PATHWAY | 36 | 0.727104 | 1.632334 | 0.015414 | 0.134016 | 0.956 | 1275 | tags=36%, list=6%, signal=38% |
| GO_T_HELPER_1_TYPE_IMMUNE_RESPONSE | 41 | 0.703003 | 1.634092 | 0.011494 | 0.135976 | 0.956 | 2917 | tags=59%, list=13%, signal=67% |
| GO_NEGATIVE_REGULATION_OF_T_CELL_RECEPTOR_SIGNALING_PATHWAY | 20 | 0.762535 | 1.598214 | 0.017893 | 0.141404 | 0.98 | 1275 | tags=40%, list=6%, signal=42% |
| GO_T_CELL_ACTIVATION_INVOLVED_IN_IMMUNE_RESPONSE | 93 | 0.665913 | 1.705225 | 0.005769 | 0.202523 | 0.89 | 2951 | tags=48%, list=14%, signal=56% |
| GO_RESPONSE_TO_TYPE_I_INTERFERON | 83 | 0.64012 | 1.745469 | 0.015267 | 0.281605 | 0.799 | 3725 | tags=48%, list=17%, signal=58% |
| GO_POSITIVE_REGULATION_OF_ALPHA_BETA_T_CELL_ACTIVATION | 58 | 0.663015 | 1.608101 | 0.019194 | 0.142386 | 0.972 | 2951 | tags=53%, list=14%, signal=62% |

| | | | | | | | | |
|---|---|---|---|---|---|---|---|---|
| GO_ALPHA_BETA_T_CELL_ACTIVATION | 129 | 0.644528 | 1.655476 | 0.01711 | 0.147859 | 0.943 | 2951 | tags=48%, list=14%, signal=55% |
| GO_POSITIVE_REGULATION_OF_LEUKOCYTE_DIFFERENTIATION | 139 | 0.628029 | 1.671682 | 0.009862 | 0.168808 | 0.929 | 2246 | tags=40%, list=10%, signal=45% |
| GO_NATURAL_KILLER_CELL_ACTIVATION | 70 | 0.628578 | 1.742537 | 0.001931 | 0.262159 | 0.804 | 1792 | tags=33%, list=8%, signal=36% |
| GO_POSITIVE_REGULATION_OF_ALPHA_BETA_T_CELL_DIFFERENTIATION | 45 | 0.640741 | 1.605087 | 0.009728 | 0.142809 | 0.976 | 2951 | tags=51%, list=14%, signal=59% |
| GO_NON_MEMBRANE_SPANNING_PROTEIN_TYROSINE_KINASE_ACTIVITY | 44 | 0.634397 | 1.648452 | 0.015414 | 0.141561 | 0.947 | 2963 | tags=43%, list=14%, signal=50% |
| GO_CD4_POSITIVE_ALPHA_BETA_T_CELL_DIFFERENTIATION | 71 | 0.665356 | 1.660691 | 0.011561 | 0.15035 | 0.941 | 3139 | tags=56%, list=14%, signal=66% |
| GO_LYMPHOCYTE_ACTIVATION_INVOLVED_IN_IMMUNE_RESPONSE | 161 | 0.643853 | 1.745157 | 0 | 0.263288 | 0.799 | 2951 | tags=43%, list=14%, signal=49% |
| GO_POSITIVE_REGULATION_OF_CELL_ACTIVATION | 295 | 0.641077 | 1.678417 | 0.005837 | 0.186258 | 0.921 | 2659 | tags=46%, list=12%, signal=51% |
| GO_T_CELL_ACTIVATION | 428 | 0.609767 | 1.697589 | 0.005929 | 0.192147 | 0.9 | 2262 | tags=38%, list=10%, signal=42% |
| GO_REGULATION_OF_ANTIGEN_RECEPTOR_MEDIATED_SIGNALING_PATHWAY | 57 | 0.730172 | 1.652262 | 0.011321 | 0.145856 | 0.944 | 2077 | tags=46%, list=10%, signal=50% |
| GO_POSITIVE_REGULATION_OF_LYMPHOCYTE_DIFFERENTIATION | 90 | 0.65334 | 1.614403 | 0.017476 | 0.139523 | 0.969 | 2226 | tags=47%, list=10%, signal=52% |
| GO_LYMPHOCYTE_DIFFERENTIATION | 330 | 0.595905 | 1.664713 | 0.009921 | 0.15288 | 0.939 | 2712 | tags=39%, list=12%, signal=44% |
| GO_DEFENSE_RESPONSE_TO_VIRUS | 216 | 0.619116 | 1.794858 | 0.003922 | 0.408072 | 0.663 | 3452 | tags=40%, list=16%, signal=47% |
| GO_REGULATION_OF_LEUKOCYTE_DEGRANULATION | 41 | 0.706329 | 1.753255 | 0.002 | 0.312143 | 0.778 | 2620 | tags=44%, list=12%, signal=50% |
| GO_POSITIVE_REGULATION_OF_INTERFERON_ALPHA_PRODUCTION | 21 | 0.677428 | 1.570643 | 0.038835 | 0.149827 | 0.989 | 5572 | tags=62%, list=26%, signal=83% |
| GO_RESPONSE_TO_VIRUS | 293 | 0.57097 | 1.736362 | 0.003937 | 0.224221 | 0.819 | 3493 | tags=39%, list=16%, signal=45% |
| GO_REGULATION_OF_ALPHA_BETA_T_CELL_DIFFERENTIATION | 60 | 0.676287 | 1.658423 | 0.003891 | 0.146004 | 0.941 | 3139 | tags=53%, list=14%, signal=62% |
| GO_REGULATION_OF_DEFENSE_RESPONSE_TO_VIRUS_BY_VIRUS | 30 | 0.708637 | 1.854336 | 0 | 1 | 0.467 | 1060 | tags=27%, list=5%, signal=28% |

| | | | | | | | | |
|---|---|---|---|---|---|---|---|---|
| GO_CD4_POSITIVE_ALPHA_BETA_T_CELL_ACTIVATION | 86 | 0.649942 | 1.661529 | 0.009634 | 0.151949 | 0.941 | 2951 | tags=50%, list=14%, signal=58% |
| GO_REGULATION_OF_NATURAL_KILLER_CELL_ACTIVATION | 32 | 0.629212 | 1.552107 | 0.043053 | 0.158403 | 0.992 | 1111 | tags=34%, list=5%, signal=36% |
| GO_POSITIVE_REGULATION_OF_LEUKOCYTE_CELL_CELL_ADHESION | 202 | 0.676791 | 1.65264 | 0.001996 | 0.147196 | 0.944 | 2259 | tags=47%, list=10%, signal=52% |
| GO_INTERFERON_GAMMA_PRODUCTION | 102 | 0.679368 | 1.620991 | 0.015534 | 0.136934 | 0.964 | 2226 | tags=49%, list=10%, signal=54% |
| GO_ACTIVATED_T_CELL_PROLIFERATION | 39 | 0.576155 | 1.607167 | 0.011881 | 0.142201 | 0.973 | 2036 | tags=31%, list=9%, signal=34% |
| GO_CELLULAR_RESPONSE_TO_INTERFERON_BETA | 19 | 0.784348 | 1.841141 | 0 | 0.557828 | 0.53 | 599 | tags=32%, list=3%, signal=32% |
| GO_ACTIVATION_OF_INNATE_IMMUNE_RESPONSE | 288 | 0.456736 | 1.545998 | 0.046653 | 0.159748 | 0.993 | 5110 | tags=42%, list=24%, signal=54% |
| GO_MYD88_INDEPENDENT_TOLL_LIKE_RECEPTOR_SIGNALING_PATHWAY | 32 | 0.597825 | 1.635912 | 0.02947 | 0.136223 | 0.956 | 3762 | tags=34%, list=17%, signal=42% |
| GO_INTERFERON_ALPHA_PRODUCTION | 29 | 0.689032 | 1.62663 | 0.019084 | 0.136234 | 0.962 | 2801 | tags=41%, list=13%, signal=47% |
| GO_T_CELL_DIFFERENTIATION | 226 | 0.617476 | 1.685026 | 0.009728 | 0.184682 | 0.914 | 2712 | tags=42%, list=12%, signal=48% |
| GO_POSITIVE_REGULATION_OF_INTERFERON_BETA_PRODUCTION | 30 | 0.603305 | 1.701191 | 0.011538 | 0.194224 | 0.897 | 5572 | tags=53%, list=26%, signal=72% |
| GO_T_CELL_PROLIFERATION | 169 | 0.622033 | 1.706032 | 0 | 0.20723 | 0.89 | 2259 | tags=38%, list=10%, signal=43% |
| GO_TUMOR_NECROSIS_FACTOR_BIOSYNTHETIC_PROCESS | 30 | 0.674022 | 1.57796 | 0.032505 | 0.145866 | 0.988 | 2035 | tags=43%, list=9%, signal=48% |
| GO_RESPONSE_TO_INTERFERON_ALPHA | 20 | 0.744673 | 1.551279 | 0.014315 | 0.15806 | 0.992 | 3018 | tags=60%, list=14%, signal=70% |
| GO_REGULATION_OF_ALPHA_BETA_T_CELL_ACTIVATION | 85 | 0.682202 | 1.660675 | 0.005725 | 0.149342 | 0.941 | 2951 | tags=52%, list=14%, signal=60% |
| GO_POSITIVE_REGULATION_OF_RESPONSE_TO_BIOTIC_STIMULUS | 357 | 0.506282 | 1.620014 | 0.023576 | 0.137821 | 0.965 | 3432 | tags=33%, list=16%, signal=39% |
| GO_T_CELL_DIFFERENTIATION_INVOLVED_IN_IMMUNE_RESPONSE | 65 | 0.674108 | 1.643087 | 0.015385 | 0.137681 | 0.952 | 3139 | tags=55%, list=14%, signal=65% |
| GO_LEUKOCYTE_DIFFERENTIATION | 483 | 0.557242 | 1.651578 | 0.007968 | 0.142553 | 0.944 | 2980 | tags=37%, list=14%, signal=42% |

| | | | | | | | | |
|---|---|---|---|---|---|---|---|---|
| GO_REGULATION_OF_T_HELPER_1_TYPE_IMMUNE_RESPONSE | 26 | 0.695444 | 1.53512 | 0.039548 | 0.166197 | 0.993 | 2917 | tags=65%, list=13%, signal=75% |
| GO_REGULATION_OF_OSTEOCLAST_DIFFERENTIATION | 60 | 0.568754 | 1.574513 | 0.027613 | 0.1466 | 0.988 | 2181 | tags=33%, list=10%, signal=37% |
| GO_POSITIVE_REGULATION_OF_HEMOPOIESIS | 174 | 0.584335 | 1.657524 | 0.006061 | 0.145468 | 0.942 | 3762 | tags=47%, list=17%, signal=57% |
| GO_POSITIVE_REGULATION_OF_T_HELPER_1_TYPE_IMMUNE_RESPONSE | 19 | 0.747776 | 1.58361 | 0.01341 | 0.145859 | 0.986 | 2659 | tags=68%, list=12%, signal=78% |
| GO_REGULATION_OF_MYELOID_LEUKOCYTE_DIFFERENTIATION | 110 | 0.535268 | 1.648135 | 0.009823 | 0.140417 | 0.947 | 2380 | tags=32%, list=11%, signal=36% |
| GO_ADAPTIVE_IMMUNE_RESPONSE | 360 | 0.670422 | 1.712724 | 0.003781 | 0.215094 | 0.874 | 3092 | tags=50%, list=14%, signal=57% |
| GO_REGULATION_OF_DEFENSE_RESPONSE_TO_VIRUS_BY_HOST | 33 | 0.709069 | 1.686116 | 0.007767 | 0.186476 | 0.914 | 1387 | tags=36%, list=6%, signal=39% |
| GO_PHAGOCYTOSIS | 244 | 0.518121 | 1.630926 | 0.017928 | 0.134238 | 0.958 | 2232 | tags=32%, list=10%, signal=35% |
| GO_REGULATION_OF_LEUKOCYTE_DIFFERENTIATION | 256 | 0.587823 | 1.669792 | 0.007921 | 0.166623 | 0.932 | 2246 | tags=36%, list=10%, signal=39% |
| GO_TUMOR_NECROSIS_FACTOR_SUPERFAMILY_CYTOKINE_PRODUCTION | 144 | 0.592573 | 1.566533 | 0.038153 | 0.153127 | 0.989 | 1602 | tags=36%, list=7%, signal=39% |
| GO_REGULATION_OF_RESPONSE_TO_BIOTIC_STIMULUS | 472 | 0.540505 | 1.701238 | 0.011696 | 0.196978 | 0.897 | 3436 | tags=36%, list=16%, signal=42% |
| GO_ANTIGEN_PROCESSING_AND_PRESENTATION | 200 | 0.526552 | 1.75989 | 0.007921 | 0.352187 | 0.769 | 3046 | tags=29%, list=14%, signal=33% |
| GO_ADAPTIVE_IMMUNE_RESPONSE_BASED_ON_SOMATIC_RECOMBINATION_OF_IMMUNE_RECEPTORS_BUILT_FROM_IMMUNOGLOBULIN_SUPERFAMILY_DOMAINS | 232 | 0.630871 | 1.667789 | 0.011561 | 0.15828 | 0.936 | 2422 | tags=43%, list=11%, signal=48% |
| GO_REGULATION_OF_T_CELL_ACTIVATION | 292 | 0.647371 | 1.69573 | 0.001996 | 0.193919 | 0.902 | 2259 | tags=42%, list=10%, signal=46% |
| GO_POSITIVE_REGULATION_OF_CYTOKINE_PRODUCTION | 425 | 0.584386 | 1.691284 | 0.007813 | 0.188449 | 0.909 | 2226 | tags=32%, list=10%, signal=35% |
| GO_REGULATION_OF_HEMOPOIESIS | 411 | 0.517847 | 1.667312 | 0.006048 | 0.155347 | 0.936 | 2988 | tags=31%, list=14%, signal=35% |
| GO_POSITIVE_REGULATION_OF_CELL_CELL_ADHESION | 236 | 0.637768 | 1.675806 | 0.001961 | 0.176961 | 0.925 | 2280 | tags=44%, list=10%, signal=48% |

| | | | | | | | | |
|---|---|---|---|---|---|---|---|---|
| GO_POSITIVE_REGULATION_OF_IMMUNE_EFFECTOR_PROCESS | 191 | 0.621286 | 1.659109 | 0.009881 | 0.148943 | 0.941 | 3092 | tags=45%, list=14%, signal=52% |
| GO_POSITIVE_REGULATION_OF_INTERFERON_GAMMA_PRODUCTION | 59 | 0.726944 | 1.652257 | 0.013233 | 0.144955 | 0.944 | 2226 | tags=54%, list=10%, signal=60% |
| GO_POSITIVE_REGULATION_OF_DEFENSE_RESPONSE_TO_VIRUS_BY_HOST | 23 | 0.721825 | 1.605185 | 0.017717 | 0.143212 | 0.976 | 1387 | tags=39%, list=6%, signal=42% |
| GO_IMMUNOLOGICAL_SYNAPSE | 34 | 0.758648 | 1.695442 | 0.011278 | 0.186894 | 0.903 | 1701 | tags=41%, list=8%, signal=45% |
| GO_POSITIVE_REGULATION_OF_MULTI_ORGANISM_PROCESS | 486 | 0.480173 | 1.675935 | 0.013972 | 0.180359 | 0.925 | 3344 | tags=28%, list=15%, signal=33% |
| GO_T_CELL_MEDIATED_IMMUNITY | 90 | 0.668253 | 1.723569 | 0 | 0.22703 | 0.857 | 1749 | tags=41%, list=8%, signal=45% |
| GO_REGULATION_OF_NATURAL_KILLER_CELL_MEDIATED_IMMUNITY | 37 | 0.757347 | 1.552845 | 0.021154 | 0.158586 | 0.992 | 2680 | tags=65%, list=12%, signal=74% |
| GO_REGULATION_OF_MACROPHAGE_MIGRATION | 35 | 0.647091 | 1.63291 | 0.005929 | 0.13505 | 0.956 | 1990 | tags=37%, list=9%, signal=41% |
| GO_LEUKOCYTE_CELL_CELL_ADHESION | 311 | 0.621727 | 1.616395 | 0.015968 | 0.139394 | 0.968 | 2259 | tags=41%, list=10%, signal=45% |
| GO_CYTOKINE_SECRETION | 213 | 0.610559 | 1.61639 | 0.015474 | 0.138848 | 0.968 | 2077 | tags=40%, list=10%, signal=44% |
| GO_LYMPHOCYTE_MEDIATED_IMMUNITY | 226 | 0.655432 | 1.705111 | 0.005814 | 0.199548 | 0.89 | 2108 | tags=42%, list=10%, signal=46% |
| GO_RESPONSE_TO_INTERFERON_GAMMA | 176 | 0.614366 | 1.672355 | 0.007937 | 0.170389 | 0.927 | 3073 | tags=46%, list=14%, signal=53% |
| GO_CD8_POSITIVE_ALPHA_BETA_T_CELL_ACTIVATION | 21 | 0.757175 | 1.599962 | 0.009653 | 0.142147 | 0.98 | 2236 | tags=62%, list=10%, signal=69% |
| GO_SYNCYTIUM_FORMATION | 48 | 0.592116 | 1.668541 | 0.016822 | 0.162141 | 0.934 | 2770 | tags=35%, list=13%, signal=41% |
| GO_REGULATION_OF_IMMUNE_EFFECTOR_PROCESS | 357 | 0.596355 | 1.670183 | 0.005941 | 0.16885 | 0.932 | 3163 | tags=42%, list=15%, signal=48% |
| GO_MYOBLAST_FUSION | 36 | 0.584878 | 1.609542 | 0.027726 | 0.141783 | 0.971 | 2770 | tags=36%, list=13%, signal=41% |
| GO_REGULATION_OF_LYMPHOCYTE_ACTIVATION | 391 | 0.623001 | 1.681151 | 0.004 | 0.184226 | 0.917 | 2259 | tags=39%, list=10%, signal=43% |
| GO_LEUKOCYTE_PROLIFERATION | 269 | 0.586043 | 1.625038 | 0.015873 | 0.136793 | 0.963 | 2312 | tags=38%, list=11%, signal=41% |

| | | | | | | | | |
|---|---|---|---|---|---|---|---|---|
| GO_PRODUCTION_OF_MOLECULAR_MEDIATOR_OF_IMMUNE_RESPONSE | 188 | 0.55472 | 1.547922 | 0.047431 | 0.15983 | 0.993 | 3143 | tags=39%, list=14%, signal=45% |
| GO_POSITIVE_REGULATION_OF_T_CELL_PROLIFERATION | 85 | 0.694952 | 1.687499 | 0.001942 | 0.18778 | 0.913 | 2259 | tags=48%, list=10%, signal=54% |
| GO_POSITIVE_REGULATION_OF_MYELOID_LEUKOCYTE_DIFFERENTIATION | 53 | 0.639712 | 1.715147 | 0.003976 | 0.226511 | 0.872 | 2380 | tags=36%, list=11%, signal=40% |
| GO_POSITIVE_REGULATION_OF_TUMOR_NECROSIS_FACTOR_SUPERFAMILY_CYTOKINE_PRODUCTION | 82 | 0.620637 | 1.622987 | 0.023211 | 0.137991 | 0.964 | 1148 | tags=37%, list=5%, signal=38% |
| GO_POSITIVE_REGULATION_OF_CD4_POSITIVE_ALPHA_BETA_T_CELL_ACTIVATION | 35 | 0.739347 | 1.599006 | 0.007828 | 0.141576 | 0.98 | 2951 | tags=63%, list=14%, signal=73% |
| GO_REGULATION_OF_LEUKOCYTE_MEDIATED_CYTOTOXICITY | 65 | 0.711503 | 1.604395 | 0.01378 | 0.142239 | 0.976 | 2108 | tags=57%, list=10%, signal=63% |
| GO_INTERLEUKIN_12_PRODUCTION | 54 | 0.697462 | 1.669918 | 0.003795 | 0.167903 | 0.932 | 2226 | tags=50%, list=10%, signal=56% |
| GO_REGULATION_OF_LEUKOCYTE_MIGRATION | 176 | 0.577243 | 1.539436 | 0.045187 | 0.162467 | 0.993 | 2232 | tags=36%, list=10%, signal=40% |
| GO_INTERLEUKIN_8_SECRETION | 28 | 0.665304 | 1.549334 | 0.021526 | 0.158964 | 0.992 | 1427 | tags=43%, list=7%, signal=46% |
| GO_REGULATION_OF_LEUKOCYTE_MEDIATED_IMMUNITY | 180 | 0.652107 | 1.693103 | 0.001976 | 0.186785 | 0.905 | 3139 | tags=49%, list=14%, signal=57% |
| GO_T_CELL_MIGRATION | 61 | 0.72474 | 1.676006 | 0.00198 | 0.182017 | 0.925 | 2014 | tags=51%, list=9%, signal=56% |
| GO_REGULATION_OF_MYELOID_LEUKOCYTE_MEDIATED_IMMUNITY | 50 | 0.61928 | 1.57531 | 0.033932 | 0.146725 | 0.988 | 2620 | tags=40%, list=12%, signal=45% |
| GO_REGULATION_OF_RESPONSE_TO_CYTOKINE_STIMULUS | 162 | 0.536259 | 1.649265 | 0.023576 | 0.143722 | 0.947 | 4014 | tags=41%, list=18%, signal=50% |
| GO_LYMPHOCYTE_HOMEOSTASIS | 61 | 0.648387 | 1.754054 | 0.001916 | 0.324515 | 0.777 | 2704 | tags=36%, list=12%, signal=41% |
| GO_NEGATIVE_REGULATION_OF_CYTOKINE_PRODUCTION | 249 | 0.544997 | 1.561676 | 0.036072 | 0.153674 | 0.991 | 2424 | tags=34%, list=11%, signal=38% |
| GO_FC_RECEPTOR_MEDIATED_STIMULATORY_SIGNALING_PATHWAY | 80 | 0.502192 | 1.624398 | 0.01996 | 0.136574 | 0.963 | 3821 | tags=35%, list=18%, signal=42% |
| GO_LYMPHOCYTE_MIGRATION | 101 | 0.687346 | 1.595362 | 0.019646 | 0.142233 | 0.98 | 2014 | tags=48%, list=9%, signal=52% |
| GO_REGULATION_OF_CD4_POSITIVE_ALPHA_BETA_T_CELL_DIFFERENTIATION | 45 | 0.757258 | 1.668146 | 0 | 0.161481 | 0.934 | 3139 | tags=64%, list=14%, signal=75% |

| | | | | | | | | |
|---|---|---|---|---|---|---|---|---|
| GO_LEUKOCYTE_MEDIATED_CYTOTOXICITY | 94 | 0.670961 | 1.667716 | 0.009747 | 0.157177 | 0.936 | 1897 | tags=46%, list=9%, signal=50% |
| GO_INTERLEUKIN_8_PRODUCTION | 75 | 0.595735 | 1.543537 | 0.033865 | 0.162144 | 0.993 | 1427 | tags=31%, list=7%, signal=33% |
| GO_ANTIGEN_PROCESSING_AND_PRESENTATION_OF_EXOGENOUS_PEPTIDE_ANTIGEN_VIA_MHC_CLASS_I | 70 | 0.591493 | 1.726731 | 0.001969 | 0.222534 | 0.845 | 6739 | tags=64%, list=31%, signal=93% |
| GO_REGULATION_OF_LYMPHOCYTE_DIFFERENTIATION | 160 | 0.63558 | 1.649375 | 0.007984 | 0.144371 | 0.947 | 2226 | tags=41%, list=10%, signal=45% |
| GO_POSITIVE_REGULATION_OF_LEUKOCYTE_PROLIFERATION | 125 | 0.624584 | 1.619692 | 0.009766 | 0.137171 | 0.967 | 3602 | tags=53%, list=17%, signal=63% |
| GO_REGULATION_OF_T_CELL_DIFFERENTIATION | 129 | 0.661295 | 1.665499 | 0.005894 | 0.152543 | 0.938 | 2226 | tags=44%, list=10%, signal=49% |
| GO_POSITIVE_REGULATION_OF_CD4_POSITIVE_ALPHA_BETA_T_CELL_DIFFERENTIATION | 29 | 0.748337 | 1.576903 | 0.013861 | 0.146158 | 0.988 | 2951 | tags=66%, list=14%, signal=76% |
| GO_REGULATION_OF_LYMPHOCYTE_MIGRATION | 55 | 0.689108 | 1.69555 | 0.003831 | 0.189128 | 0.902 | 2014 | tags=44%, list=9%, signal=48% |
| GO_T_CELL_SELECTION | 46 | 0.750541 | 1.646313 | 0.009416 | 0.137883 | 0.947 | 4197 | tags=72%, list=19%, signal=89% |
| GO_POSITIVE_REGULATION_OF_LEUKOCYTE_MIGRATION | 121 | 0.623584 | 1.613673 | 0.019608 | 0.138965 | 0.969 | 1990 | tags=38%, list=9%, signal=42% |
| GO_LIPOPOLYSACCHARIDE_MEDIATED_SIGNALING_PATHWAY | 55 | 0.64547 | 1.607776 | 0.03876 | 0.141834 | 0.972 | 2226 | tags=40%, list=10%, signal=44% |
| GO_NEGATIVE_REGULATION_OF_REGULATED_SECRETORY_PATHWAY | 20 | 0.752734 | 1.609297 | 0.004082 | 0.141593 | 0.971 | 1700 | tags=40%, list=8%, signal=43% |
| GO_INTERLEUKIN_1_BETA_PRODUCTION | 83 | 0.622144 | 1.533827 | 0.034884 | 0.16784 | 0.994 | 1575 | tags=43%, list=7%, signal=47% |
| GO_REGULATION_OF_CELL_KILLING | 86 | 0.684438 | 1.641583 | 0.015385 | 0.138433 | 0.952 | 2680 | tags=52%, list=12%, signal=59% |
| GO_POSITIVE_REGULATION_OF_CYTOKINE_SECRETION | 130 | 0.579451 | 1.55734 | 0.033074 | 0.156184 | 0.992 | 2035 | tags=36%, list=9%, signal=40% |
| GO_NEGATIVE_REGULATION_OF_IMMUNE_SYSTEM_PROCESS | 408 | 0.555341 | 1.621046 | 0.01006 | 0.137441 | 0.964 | 2077 | tags=32%, list=10%, signal=35% |
| GO_REGULATION_OF_SYNCYTIUM_FORMATION_BY_PLASMA_MEMBRANE_FUSION | 26 | 0.729529 | 1.632993 | 0.01341 | 0.135542 | 0.956 | 465 | tags=31%, list=2%, signal=31% |
| GO_NIK_NF_KAPPAB_SIGNALING | 154 | 0.485864 | 1.637969 | 0.014028 | 0.138134 | 0.954 | 4947 | tags=38%, list=23%, signal=48% |

| | | | | | | | | |
|---|---|---|---|---|---|---|---|---|
| GO_ANTIGEN_PROCESSING_AND_PRESENTATION_OF_PEPTIDE_ANTIGEN_VIA_MHC_CLASS_I | 91 | 0.529362 | 1.742364 | 0.011881 | 0.254226 | 0.804 | 6739 | tags=58%, list=31%, signal=84% |
| GO_POSITIVE_REGULATION_OF_NATURAL_KILLER_CELL_MEDIATED_CYTOTOXICITY | 20 | 0.832431 | 1.529116 | 0.019493 | 0.171089 | 0.994 | 2514 | tags=70%, list=12%, signal=79% |
| GO_INTERFERON_GAMMA_MEDIATED_SIGNALING_PATHWAY | 72 | 0.643796 | 1.631347 | 0.032755 | 0.134262 | 0.958 | 3030 | tags=50%, list=14%, signal=58% |
| GO_POSITIVE_REGULATION_OF_CELL_ADHESION | 372 | 0.524638 | 1.516244 | 0.038229 | 0.180509 | 0.997 | 2586 | tags=34%, list=12%, signal=38% |
| GO_POSITIVE_REGULATION_OF_TUMOR_NECROSIS_FACTOR_BIOSYNTHETIC_PROCESS | 20 | 0.794877 | 1.71824 | 0.001919 | 0.227315 | 0.864 | 1148 | tags=45%, list=5%, signal=47% |
| GO_REGULATION_OF_MYELOID_CELL_DIFFERENTIATION | 199 | 0.49506 | 1.677346 | 0.003992 | 0.182839 | 0.923 | 2580 | tags=29%, list=12%, signal=32% |
| GO_POSITIVE_REGULATION_OF_OSTEOCLAST_DIFFERENTIATION | 25 | 0.729097 | 1.699722 | 0.005792 | 0.194898 | 0.899 | 1840 | tags=40%, list=8%, signal=44% |
| GO_I_KAPPAB_PHOSPHORYLATION | 18 | 0.720468 | 1.654744 | 0.001931 | 0.148209 | 0.943 | 4705 | tags=61%, list=22%, signal=78% |
| GO_POSITIVE_REGULATION_OF_NATURAL_KILLER_CELL_MEDIATED_IMMUNITY | 23 | 0.817612 | 1.512059 | 0.036538 | 0.184622 | 0.997 | 1294 | tags=61%, list=6%, signal=65% |
| GO_PRODUCTION_OF_MOLECULAR_MEDIATOR_INVOLVED_IN_INFLAMMATORY_RESPONSE | 64 | 0.557946 | 1.504455 | 0.042254 | 0.188977 | 0.998 | 1148 | tags=27%, list=5%, signal=28% |
| GO_POSITIVE_REGULATION_OF_LEUKOCYTE_MEDIATED_CYTOTOXICITY | 43 | 0.757691 | 1.63672 | 0.013619 | 0.137141 | 0.955 | 2108 | tags=60%, list=10%, signal=67% |
| GO_REGULATION_OF_MAST_CELL_ACTIVATION | 36 | 0.608929 | 1.551067 | 0.035573 | 0.157617 | 0.992 | 2620 | tags=39%, list=12%, signal=44% |
| GO_REGULATION_OF_MACROPHAGE_CHEMOTAXIS | 23 | 0.584108 | 1.530758 | 0.044574 | 0.170442 | 0.994 | 1990 | tags=30%, list=9%, signal=33% |
| GO_INTERLEUKIN_1_PRODUCTION | 96 | 0.604986 | 1.520069 | 0.044574 | 0.177688 | 0.996 | 2324 | tags=45%, list=11%, signal=50% |
| GO_NEGATIVE_REGULATION_OF_EXOCYTOSIS | 28 | 0.692518 | 1.667625 | 0.007984 | 0.15603 | 0.936 | 2068 | tags=32%, list=10%, signal=35% |
| GO_MHC_PROTEIN_BINDING | 27 | 0.751244 | 1.600268 | 0.029412 | 0.142762 | 0.98 | 1060 | tags=52%, list=5%, signal=54% |
| GO_T_CELL_MEDIATED_CYTOTOXICITY | 35 | 0.710116 | 1.672631 | 0.007828 | 0.171495 | 0.926 | 2108 | tags=51%, list=10%, signal=57% |
| GO_POSITIVE_REGULATION_OF_CYTOKINE_BIOSYNTHETIC_PROCESS | 67 | 0.768965 | 1.847391 | 0 | 0.675251 | 0.493 | 1553 | tags=45%, list=7%, signal=48% |

| | | | | | | | | |
|---|---|---|---|---|---|---|---|---|
| GO_CELLULAR_DEFENSE_RESPONSE | 49 | 0.718718 | 1.636794 | 0.011472 | 0.137665 | 0.955 | 1227 | tags=47%, list=6%, signal=50% |
| GO_REGULATION_OF_T_CELL_MIGRATION | 40 | 0.689454 | 1.676333 | 0 | 0.183084 | 0.924 | 1869 | tags=43%, list=9%, signal=46% |
| GO_TUMOR_NECROSIS_FACTOR_MEDIATED_SIGNALING_PATHWAY | 154 | 0.507805 | 1.649515 | 0.021739 | 0.144946 | 0.946 | 5298 | tags=45%, list=24%, signal=60% |
| GO_RESPIRATORY_BURST | 30 | 0.763634 | 1.645026 | 0.001946 | 0.138351 | 0.949 | 2141 | tags=53%, list=10%, signal=59% |
| GO_MYELOID_LEUKOCYTE_DIFFERENTIATION | 194 | 0.526718 | 1.635197 | 0.011928 | 0.136834 | 0.956 | 3762 | tags=40%, list=17%, signal=48% |
| GO_DENDRITIC_CELL_DIFFERENTIATION | 37 | 0.712 | 1.644301 | 0.009728 | 0.138079 | 0.951 | 2659 | tags=51%, list=12%, signal=58% |
| GO_POSITIVE_REGULATION_OF_LYMPHOCYTE_MEDIATED_IMMUNITY | 90 | 0.728478 | 1.695708 | 0.001969 | 0.191359 | 0.902 | 3602 | tags=63%, list=17%, signal=76% |
| GO_REGULATION_OF_T_HELPER_CELL_DIFFERENTIATION | 34 | 0.708638 | 1.659369 | 0.001961 | 0.149613 | 0.941 | 3436 | tags=62%, list=16%, signal=73% |
| GO_LEUKOCYTE_ACTIVATION_INVOLVED_IN_INFLAMMATORY_RESPONSE | 43 | 0.743805 | 1.562629 | 0.015656 | 0.155018 | 0.991 | 1165 | tags=51%, list=5%, signal=54% |
| GO_TYPE_I_INTERFERON_PRODUCTION | 119 | 0.589848 | 1.818684 | 0.001976 | 0.427165 | 0.606 | 5855 | tags=48%, list=27%, signal=65% |
| GO_INTERLEUKIN_2_PRODUCTION | 63 | 0.659841 | 1.622658 | 0.009766 | 0.137271 | 0.964 | 1667 | tags=41%, list=8%, signal=45% |
| GO_REGULATION_OF_CELL_CELL_ADHESION | 367 | 0.576465 | 1.604828 | 0.01002 | 0.142685 | 0.976 | 2280 | tags=36%, list=10%, signal=40% |
| GO_REGULATION_OF_CD4_POSITIVE_ALPHA_BETA_T_CELL_ACTIVATION | 56 | 0.735077 | 1.668852 | 0 | 0.164262 | 0.934 | 3139 | tags=59%, list=14%, signal=69% |
| GO_RESPONSE_TO_AMYLOID_BETA | 47 | 0.637475 | 1.561687 | 0.026104 | 0.15406 | 0.991 | 1183 | tags=30%, list=5%, signal=31% |
| GO_INTERFERON_BETA_PRODUCTION | 48 | 0.582404 | 1.721311 | 0.005871 | 0.228245 | 0.86 | 5572 | tags=52%, list=26%, signal=70% |
| GO_REGULATION_OF_LYMPHOCYTE_MEDIATED_IMMUNITY | 132 | 0.670852 | 1.668999 | 0.001988 | 0.166822 | 0.934 | 3139 | tags=53%, list=14%, signal=62% |
| GO_RESPONSE_TO_INTERFERON_BETA | 27 | 0.772964 | 1.785216 | 0 | 0.383432 | 0.699 | 2355 | tags=52%, list=11%, signal=58% |
| GO_CELL_KILLING | 148 | 0.566326 | 1.578983 | 0.02277 | 0.145301 | 0.988 | 2680 | tags=39%, list=12%, signal=44% |

| | | | | | | | | |
|---|---|---|---|---|---|---|---|---|
| GO_MYD88_DEPENDENT_TOLL_LIKE_RECEPTOR_SIGNALING_PATHWAY | 32 | 0.729719 | 1.584935 | 0.033138 | 0.145727 | 0.984 | 1390 | tags=44%, list=6%, signal=47% |
| GO_POSITIVE_REGULATION_OF_DEFENSE_RESPONSE | 451 | 0.506731 | 1.569983 | 0.040777 | 0.149952 | 0.989 | 3272 | tags=34%, list=15%, signal=39% |
| GO_POSITIVE_REGULATION_OF_LEUKOCYTE_MEDIATED_IMMUNITY | 116 | 0.696066 | 1.715748 | 0.005917 | 0.229474 | 0.871 | 3092 | tags=52%, list=14%, signal=60% |
| GO_POSITIVE_REGULATION_OF_MACROPHAGE_ACTIVATION | 21 | 0.696343 | 1.559372 | 0.022556 | 0.155115 | 0.992 | 2620 | tags=52%, list=12%, signal=60% |
| GO_POSITIVE_REGULATION_OF_CELL_KILLING | 56 | 0.743797 | 1.71187 | 0.001923 | 0.213628 | 0.876 | 2108 | tags=52%, list=10%, signal=57% |
| GO_B_CELL_ACTIVATION_INVOLVED_IN_IMMUNE_RESPONSE | 68 | 0.642114 | 1.648377 | 0.009921 | 0.140826 | 0.947 | 4771 | tags=56%, list=22%, signal=71% |
| GO_LEUKOCYTE_HOMEOSTASIS | 81 | 0.671093 | 1.728475 | 0.001965 | 0.234102 | 0.837 | 2704 | tags=40%, list=12%, signal=45% |
| GO_INTERLEUKIN_10_PRODUCTION | 50 | 0.777316 | 1.647994 | 0 | 0.139055 | 0.947 | 1492 | tags=52%, list=7%, signal=56% |
| GO_FICOLIN_1_RICH_GRANULE_MEMBRANE | 56 | 0.619805 | 1.689659 | 0.017613 | 0.187799 | 0.911 | 1899 | tags=34%, list=9%, signal=37% |
| GO_REGULATION_OF_MYOBLAST_FUSION | 20 | 0.74135 | 1.638447 | 0.005693 | 0.138191 | 0.954 | 1792 | tags=40%, list=8%, signal=44% |
| GO_LEUKOCYTE_MIGRATION | 402 | 0.564321 | 1.570929 | 0.021912 | 0.149912 | 0.989 | 2173 | tags=35%, list=10%, signal=38% |
| GO_LEUKOCYTE_CHEMOTAXIS | 194 | 0.622202 | 1.595452 | 0.015873 | 0.142571 | 0.98 | 2077 | tags=43%, list=10%, signal=47% |
| GO_PHAGOCYTIC_CUP | 24 | 0.664571 | 1.592329 | 0.025896 | 0.141975 | 0.981 | 3487 | tags=58%, list=16%, signal=69% |
| GO_POSITIVE_REGULATION_OF_INTERLEUKIN_12_PRODUCTION | 33 | 0.687373 | 1.567614 | 0.013566 | 0.152155 | 0.989 | 1148 | tags=42%, list=5%, signal=45% |
| GO_REGULATION_OF_LEUKOCYTE_PROLIFERATION | 203 | 0.609616 | 1.649255 | 0.005917 | 0.142904 | 0.947 | 2312 | tags=41%, list=11%, signal=45% |
| GO_VIRAL_GENOME_REPLICATION | 113 | 0.523149 | 1.645398 | 0.027505 | 0.13859 | 0.949 | 2910 | tags=32%, list=13%, signal=37% |
| GO_POSITIVE_REGULATION_OF_NF_KAPPAB_TRANSCRIPTION_FACTOR_ACTIVITY | 144 | 0.512094 | 1.558875 | 0.040404 | 0.15509 | 0.992 | 3492 | tags=34%, list=16%, signal=40% |
| GO_MACROPHAGE_ACTIVATION | 87 | 0.658847 | 1.62848 | 0.013462 | 0.136671 | 0.962 | 1165 | tags=39%, list=5%, signal=41% |

| | | | | | | | | |
|---|---|---|---|---|---|---|---|---|
| GO_OSTEOCLAST_DIFFERENTIATION | 91 | 0.52933 | 1.587034 | 0.01222 | 0.145938 | 0.983 | 3648 | tags=42%, list=17%, signal=50% |
| GO_NITRIC_OXIDE_SYNTHASE_BIOSYNTHETIC_PROCESS | 18 | 0.752606 | 1.58284 | 0.011811 | 0.145743 | 0.986 | 2014 | tags=56%, list=9%, signal=61% |
| GO_POSITIVE_REGULATION_OF_T_CELL_MIGRATION | 29 | 0.748188 | 1.707908 | 0 | 0.205792 | 0.886 | 1869 | tags=48%, list=9%, signal=53% |
| GO_NEGATIVE_REGULATION_OF_INTERLEUKIN_10_PRODUCTION | 16 | 0.810801 | 1.582621 | 0.007547 | 0.145649 | 0.986 | 2026 | tags=63%, list=9%, signal=69% |
| GO_B_CELL_PROLIFERATION | 84 | 0.618715 | 1.516039 | 0.046512 | 0.180494 | 0.997 | 3602 | tags=49%, list=17%, signal=58% |
| GO_REGULATION_OF_B_CELL_RECEPTOR_SIGNALING_PATHWAY | 24 | 0.780802 | 1.589691 | 0.001916 | 0.144051 | 0.983 | 2077 | tags=58%, list=10%, signal=64% |
| GO_T_CELL_CYTOKINE_PRODUCTION | 40 | 0.660812 | 1.614714 | 0.016162 | 0.140194 | 0.969 | 3602 | tags=52%, list=17%, signal=63% |
| GO_ANTIGEN_BINDING | 35 | 0.699988 | 1.640808 | 0.012 | 0.138937 | 0.952 | 3073 | tags=63%, list=14%, signal=73% |
| GO_NATURAL_KILLER_CELL_MEDIATED_IMMUNITY | 54 | 0.744015 | 1.622508 | 0.013487 | 0.136905 | 0.964 | 1758 | tags=50%, list=8%, signal=54% |
| GO_REGULATION_OF_ADAPTIVE_IMMUNE_RESPONSE | 146 | 0.646475 | 1.65964 | 0.004024 | 0.150189 | 0.941 | 3602 | tags=53%, list=17%, signal=63% |
| GO_POSITIVE_REGULATION_OF_ADAPTIVE_IMMUNE_RESPONSE | 93 | 0.696237 | 1.708679 | 0.001953 | 0.20737 | 0.886 | 3602 | tags=58%, list=17%, signal=69% |
| GO_REGULATION_OF_INTERLEUKIN_4_PRODUCTION | 27 | 0.749614 | 1.666462 | 0.005792 | 0.151913 | 0.937 | 1041 | tags=48%, list=5%, signal=51% |
| GO_POSITIVE_REGULATION_OF_MACROPHAGE_MIGRATION | 20 | 0.767491 | 1.666819 | 0.007767 | 0.153686 | 0.936 | 3516 | tags=60%, list=16%, signal=72% |
| GO_REGULATION_OF_T_CELL_MEDIATED_IMMUNITY | 63 | 0.665917 | 1.641935 | 0.006 | 0.138625 | 0.952 | 1749 | tags=43%, list=8%, signal=46% |
| GO_INTERLEUKIN_6_PRODUCTION | 134 | 0.588348 | 1.562414 | 0.029821 | 0.154542 | 0.991 | 2226 | tags=40%, list=10%, signal=45% |
| GO_NEGATIVE_REGULATION_OF_INNATE_IMMUNE_RESPONSE | 52 | 0.697602 | 1.741231 | 0 | 0.242438 | 0.808 | 1823 | tags=42%, list=8%, signal=46% |
| GO_NEGATIVE_REGULATION_OF_MULTI_ORGANISM_PROCESS | 198 | 0.56716 | 1.745954 | 0.00396 | 0.2909 | 0.797 | 3452 | tags=41%, list=16%, signal=49% |
| GO_POSITIVE_REGULATION_OF_INTERLEUKIN_1_PRODUCTION | 54 | 0.644087 | 1.597146 | 0.009542 | 0.142043 | 0.98 | 1148 | tags=39%, list=5%, signal=41% |

| | | | | | | | | |
|---|---|---|---|---|---|---|---|---|
| GO_PHOSPHATIDYLINOSITOL_3_4_BISPHOSPHATE_BINDING | 24 | 0.620163 | 1.742027 | 0.007874 | 0.247575 | 0.806 | 955 | tags=21%, list=4%, signal=22% |
| GO_MACROPHAGE_MIGRATION | 47 | 0.596808 | 1.577267 | 0.017751 | 0.146074 | 0.988 | 2014 | tags=36%, list=9%, signal=40% |
| GO_INTERLEUKIN_4_PRODUCTION | 34 | 0.70805 | 1.575716 | 0.019724 | 0.146542 | 0.988 | 1041 | tags=44%, list=5%, signal=46% |
| GO_POSITIVE_REGULATION_OF_INTERLEUKIN_8_PRODUCTION | 49 | 0.632772 | 1.542038 | 0.04008 | 0.162185 | 0.993 | 1427 | tags=33%, list=7%, signal=35% |
| GO_POSITIVE_REGULATION_OF_LYMPHOCYTE_MIGRATION | 36 | 0.737191 | 1.668915 | 0 | 0.165548 | 0.934 | 1869 | tags=47%, list=9%, signal=52% |
| GO_CELL_CHEMOTAXIS | 264 | 0.583531 | 1.54934 | 0.037624 | 0.159335 | 0.992 | 2077 | tags=39%, list=10%, signal=43% |
| GO_POSITIVE_REGULATION_OF_INTERLEUKIN_8_SECRETION | 16 | 0.742549 | 1.502181 | 0.037255 | 0.189794 | 0.999 | 1427 | tags=56%, list=7%, signal=60% |
| GO_POSITIVE_REGULATION_OF_LEUKOCYTE_CHEMOTAXIS | 79 | 0.641457 | 1.646462 | 0 | 0.138351 | 0.947 | 1990 | tags=41%, list=9%, signal=44% |
| GO_NEGATIVE_REGULATION_OF_RESPONSE_TO_BIOTIC_STIMULUS | 84 | 0.61215 | 1.757298 | 0.001957 | 0.327894 | 0.772 | 1823 | tags=32%, list=8%, signal=35% |
| GO_T_CELL_HOMEOSTASIS | 38 | 0.631626 | 1.705438 | 0.003854 | 0.205335 | 0.89 | 2026 | tags=29%, list=9%, signal=32% |
| GO_REGULATION_OF_CYTOKINE_PRODUCTION_INVOLVED_IN_IMMUNE_RESPONSE | 80 | 0.663989 | 1.579221 | 0.026052 | 0.145335 | 0.988 | 3139 | tags=51%, list=14%, signal=60% |
| GO_NEGATIVE_REGULATION_OF_B_CELL_PROLIFERATION | 16 | 0.843428 | 1.727295 | 0 | 0.232093 | 0.843 | 2077 | tags=56%, list=10%, signal=62% |
| GO_MYELOID_CELL_DIFFERENTIATION | 355 | 0.464557 | 1.632773 | 0.01002 | 0.133971 | 0.956 | 3762 | tags=33%, list=17%, signal=40% |
| GO_HOMEOSTASIS_OF_NUMBER_OF_CELLS | 225 | 0.508756 | 1.680739 | 0.006085 | 0.183092 | 0.919 | 4196 | tags=38%, list=19%, signal=46% |
| GO_POSITIVE_REGULATION_OF_INTERLEUKIN_4_PRODUCTION | 22 | 0.752042 | 1.622675 | 0.005825 | 0.137843 | 0.964 | 1041 | tags=50%, list=5%, signal=52% |
| GO_B_CELL_HOMEOSTASIS | 28 | 0.665912 | 1.602981 | 0.029183 | 0.142255 | 0.976 | 4459 | tags=50%, list=21%, signal=63% |
| GO_NEGATIVE_REGULATION_OF_VIRAL_GENOME_REPLICATION | 55 | 0.651572 | 1.589706 | 0.029528 | 0.144478 | 0.983 | 2355 | tags=49%, list=11%, signal=55% |
| GO_REGULATION_OF_T_CELL_MEDIATED_CYTOTOXICITY | 27 | 0.730886 | 1.62155 | 0.015748 | 0.137226 | 0.964 | 2108 | tags=59%, list=10%, signal=66% |

| | | | | | | | | |
|---|---|---|---|---|---|---|---|---|
| GO_REGULATION_OF_PRODUCTION_OF_MOLECULAR_MEDIATOR_OF_IMMUNE_RESPONSE | 129 | 0.607851 | 1.59724 | 0.02 | 0.142386 | 0.98 | 3139 | tags=45%, list=14%, signal=52% |
| GO_REGULATION_OF_LEUKOCYTE_CHEMOTAXIS | 100 | 0.627285 | 1.634112 | 0.007722 | 0.136582 | 0.956 | 2077 | tags=40%, list=10%, signal=44% |
| GO_B_CELL_MEDIATED_IMMUNITY | 107 | 0.621184 | 1.618552 | 0.00947 | 0.137273 | 0.968 | 2053 | tags=40%, list=9%, signal=44% |
| GO_CELLULAR_RESPONSE_TO_BIOTIC_STIMULUS | 213 | 0.625709 | 1.698394 | 0.001957 | 0.195597 | 0.899 | 2226 | tags=38%, list=10%, signal=41% |
| GO_SPECIFIC_GRANULE_MEMBRANE | 83 | 0.638533 | 1.632775 | 0.009901 | 0.134592 | 0.956 | 2158 | tags=39%, list=10%, signal=43% |
| GO_RESPONSE_TO_CHEMOKINE | 85 | 0.712135 | 1.604035 | 0.009634 | 0.14174 | 0.976 | 2014 | tags=49%, list=9%, signal=54% |
| GO_B_CELL_ACTIVATION | 224 | 0.587612 | 1.601377 | 0.021569 | 0.142573 | 0.978 | 3400 | tags=41%, list=16%, signal=48% |
| GO_POSITIVE_REGULATION_OF_INTERLEUKIN_2_PRODUCTION | 31 | 0.718526 | 1.583031 | 0.021318 | 0.145886 | 0.986 | 3092 | tags=55%, list=14%, signal=64% |
| GO_RECEPTOR_SIGNALING_PATHWAY_VIA_STAT | 145 | 0.452543 | 1.498655 | 0.02544 | 0.192897 | 0.999 | 2077 | tags=29%, list=10%, signal=32% |
| GO_POSITIVE_REGULATION_OF_RESPONSE_TO_CYTOKINE_STIMULUS | 54 | 0.662697 | 1.673528 | 0.006098 | 0.173046 | 0.926 | 4014 | tags=56%, list=18%, signal=68% |
| GO_POSITIVE_REGULATION_OF_CALCIUM_MEDIATED_SIGNALING | 40 | 0.499346 | 1.466152 | 0.042636 | 0.215539 | 1 | 1081 | tags=23%, list=5%, signal=24% |
| GO_REGULATION_OF_NIK_NF_KAPPAB_SIGNALING | 95 | 0.576637 | 1.673937 | 0.004 | 0.173848 | 0.926 | 1335 | tags=25%, list=6%, signal=27% |
| GO_CYTOKINE_METABOLIC_PROCESS | 114 | 0.654664 | 1.777672 | 0 | 0.392539 | 0.718 | 1553 | tags=37%, list=7%, signal=39% |
| GO_POSITIVE_REGULATION_OF_ALPHA_BETA_T_CELL_PROLIFERATION | 18 | 0.812361 | 1.564506 | 0.009671 | 0.153668 | 0.989 | 1111 | tags=61%, list=5%, signal=64% |
| GO_POSITIVE_REGULATION_OF_NITRIC_OXIDE_SYNTHASE_BIOSYNTHETIC_PROCESS | 15 | 0.795992 | 1.541588 | 0.013619 | 0.162098 | 0.993 | 2014 | tags=67%, list=9%, signal=73% |
| GO_REGULATION_OF_B_CELL_PROLIFERATION | 60 | 0.636772 | 1.526316 | 0.033074 | 0.173241 | 0.995 | 3602 | tags=55%, list=17%, signal=66% |
| GO_NEGATIVE_REGULATION_OF_VIRAL_LIFE_CYCLE | 77 | 0.579488 | 1.579899 | 0.031683 | 0.144792 | 0.988 | 3600 | tags=49%, list=17%, signal=59% |
| GO_POSITIVE_REGULATION_OF_TYPE_I_INTERFERON_PRODUCTION | 73 | 0.598652 | 1.826392 | 0.001942 | 0.447535 | 0.575 | 5827 | tags=49%, list=27%, signal=67% |

| | | | | | | | | |
|---|---|---|---|---|---|---|---|---|
| GO_T_CELL_APOPTOTIC_PROCESS | 47 | 0.684316 | 1.745239 | 0.001873 | 0.272434 | 0.799 | 1418 | tags=32%, list=7%, signal=34% |
| GO_POSITIVE_REGULATION_OF_MYELOID_CELL_DIFFERENTIATION | 85 | 0.567208 | 1.677666 | 0.002008 | 0.185821 | 0.921 | 2580 | tags=35%, list=12%, signal=40% |
| GO_MYELOID_LEUKOCYTE_MIGRATION | 184 | 0.609361 | 1.585081 | 0.019724 | 0.145929 | 0.984 | 2158 | tags=41%, list=10%, signal=45% |
| GO_NEGATIVE_REGULATION_OF_CELL_ACTIVATION | 181 | 0.657419 | 1.711707 | 0 | 0.206543 | 0.876 | 2077 | tags=41%, list=10%, signal=45% |
| GO_PROTEIN_AUTOPHOSPHORYLATION | 223 | 0.374869 | 1.483751 | 0.028283 | 0.204879 | 0.999 | 2711 | tags=24%, list=12%, signal=27% |
| GO_RESPONSE_TO_MOLECULE_OF_BACTERIAL_ORIGIN | 317 | 0.544006 | 1.53313 | 0.039216 | 0.168592 | 0.994 | 2226 | tags=34%, list=10%, signal=38% |
| GO_T_CELL_RECEPTOR_COMPLEX | 17 | 0.807172 | 1.551909 | 0.046296 | 0.158296 | 0.992 | 1701 | tags=65%, list=8%, signal=70% |
| GO_TUMOR_NECROSIS_FACTOR_RECEPTOR_SUPERFAMILY_BINDING | 42 | 0.697434 | 1.710937 | 0.005929 | 0.204747 | 0.877 | 2236 | tags=45%, list=10%, signal=50% |
| GO_REGULATION_OF_MYOTUBE_DIFFERENTIATION | 48 | 0.584307 | 1.667805 | 0.001919 | 0.159551 | 0.936 | 3400 | tags=40%, list=16%, signal=47% |
| GO_POSITIVE_REGULATION_OF_PRODUCTION_OF_MOLECULAR_MEDIATOR_OF_IMMUNE_RESPONSE | 86 | 0.599371 | 1.541512 | 0.036437 | 0.161846 | 0.993 | 3092 | tags=45%, list=14%, signal=53% |
| GO_REGULATORY_T_CELL_DIFFERENTIATION | 28 | 0.855135 | 1.653422 | 0 | 0.146691 | 0.943 | 1667 | tags=75%, list=8%, signal=81% |
| GO_NEGATIVE_REGULATION_OF_T_CELL_MEDIATED_IMMUNITY | 17 | 0.762996 | 1.652374 | 0.002041 | 0.146642 | 0.944 | 1275 | tags=47%, list=6%, signal=50% |
| GO_POSITIVE_REGULATION_OF_NIK_NF_KAPPAB_SIGNALING | 70 | 0.628253 | 1.663686 | 0.001972 | 0.153605 | 0.939 | 1335 | tags=29%, list=6%, signal=30% |
| GO_MYELOID_DENDRITIC_CELL_ACTIVATION | 26 | 0.82026 | 1.683927 | 0.00198 | 0.182743 | 0.914 | 2369 | tags=65%, list=11%, signal=73% |
| GO_REGULATION_OF_T_CELL_CYTOKINE_PRODUCTION | 30 | 0.694568 | 1.559095 | 0.024096 | 0.155176 | 0.992 | 3092 | tags=53%, list=14%, signal=62% |
| GO_POSITIVE_REGULATION_OF_PHAGOCYTOSIS | 65 | 0.601504 | 1.576884 | 0.016 | 0.145766 | 0.988 | 2729 | tags=45%, list=13%, signal=51% |
| GO_INTERLEUKIN_1_SECRETION | 55 | 0.599248 | 1.472741 | 0.042969 | 0.212651 | 0.999 | 1213 | tags=38%, list=6%, signal=40% |
| GO_NEGATIVE_REGULATION_OF_ALPHA_BETA_T_CELL_ACTIVATION | 34 | 0.70559 | 1.712989 | 0.003968 | 0.218617 | 0.874 | 2068 | tags=47%, list=10%, signal=52% |

| | | | | | | | | |
|---|---|---|---|---|---|---|---|---|
| GO_POSITIVE_REGULATION_OF_ANTIGEN_RECEPTOR_MEDIATED_SIGNALING_PATHWAY | 19 | 0.775929 | 1.528425 | 0.031068 | 0.171084 | 0.994 | 1962 | tags=58%, list=9%, signal=64% |
| GO_LYMPHOCYTE_CHEMOTAXIS | 56 | 0.748544 | 1.612056 | 0.007813 | 0.140202 | 0.971 | 2014 | tags=57%, list=9%, signal=63% |
| GO_GLIAL_CELL_ACTIVATION | 51 | 0.704474 | 1.583692 | 0.015534 | 0.146159 | 0.986 | 1165 | tags=45%, list=5%, signal=48% |
| GO_NEGATIVE_REGULATION_OF_INTERLEUKIN_12_PRODUCTION | 16 | 0.812378 | 1.585309 | 0.00578 | 0.14606 | 0.983 | 2035 | tags=63%, list=9%, signal=69% |
| GO_REGULATION_OF_MONONUCLEAR_CELL_MIGRATION | 41 | 0.679039 | 1.611812 | 0.011561 | 0.140014 | 0.971 | 2077 | tags=44%, list=10%, signal=48% |
| GO_POSITIVE_REGULATION_OF_SMALL_GTPASE_MEDIATED_SIGNAL_TRANSDUCTION | 66 | 0.536887 | 1.535395 | 0.029644 | 0.166515 | 0.993 | 2077 | tags=29%, list=10%, signal=32% |
| GO_POSITIVE_REGULATION_OF_INTERLEUKIN_6_PRODUCTION | 83 | 0.596875 | 1.560501 | 0.031746 | 0.154266 | 0.992 | 2226 | tags=36%, list=10%, signal=40% |
| GO_ALPHA_BETA_T_CELL_PROLIFERATION | 30 | 0.763289 | 1.647083 | 0.003868 | 0.139814 | 0.947 | 1111 | tags=47%, list=5%, signal=49% |
| GO_NEGATIVE_REGULATION_OF_LYMPHOCYTE_ACTIVATION | 137 | 0.668741 | 1.765317 | 0 | 0.347583 | 0.754 | 2077 | tags=42%, list=10%, signal=47% |
| GO_THYMIC_T_CELL_SELECTION | 21 | 0.743312 | 1.567529 | 0.02907 | 0.151879 | 0.989 | 1019 | tags=43%, list=5%, signal=45% |
| GO_INTERLEUKIN_2_BIOSYNTHETIC_PROCESS | 23 | 0.755652 | 1.64689 | 0.005671 | 0.139309 | 0.947 | 1431 | tags=52%, list=7%, signal=56% |
| GO_PHOSPHATIDYLINOSITOL_3_4_5_TRISPHOSPHATE_BINDING | 34 | 0.563466 | 1.849898 | 0.001976 | 0.974251 | 0.48 | 2390 | tags=26%, list=11%, signal=30% |
| GO_PATTERN_RECOGNITION_RECEPTOR_ACTIVITY | 21 | 0.7397 | 1.563044 | 0.023256 | 0.154803 | 0.991 | 1553 | tags=48%, list=7%, signal=51% |
| GO_NEGATIVE_REGULATION_OF_INTERFERON_GAMMA_PRODUCTION | 30 | 0.650789 | 1.480014 | 0.042945 | 0.207921 | 0.999 | 1492 | tags=37%, list=7%, signal=39% |
| GO_NEGATIVE_REGULATION_OF_VIRAL_PROCESS | 93 | 0.572498 | 1.637013 | 0.012 | 0.138062 | 0.955 | 3600 | tags=45%, list=17%, signal=54% |
| GO_SH2_DOMAIN_BINDING | 35 | 0.599715 | 1.524776 | 0.02686 | 0.174139 | 0.995 | 2935 | tags=34%, list=14%, signal=40% |
| GO_IMMUNOGLOBULIN_PRODUCTION | 98 | 0.592497 | 1.593983 | 0.021359 | 0.14227 | 0.98 | 3918 | tags=44%, list=18%, signal=53% |
| GO_GRANULOCYTE_MIGRATION | 122 | 0.64776 | 1.587267 | 0.021739 | 0.146576 | 0.983 | 2068 | tags=44%, list=10%, signal=49% |

| | | | | | | | | |
|---|---|---|---|---|---|---|---|---|
| GO_REGULATION_OF_PHAGOCYTOSIS | 87 | 0.56558 | 1.594719 | 0.005952 | 0.142235 | 0.98 | 2203 | tags=40%, list=10%, signal=45% |
| GO_LEUKOCYTE_APOPTOTIC_PROCESS | 101 | 0.640109 | 1.71478 | 0.005859 | 0.218393 | 0.873 | 2077 | tags=35%, list=10%, signal=38% |
| GO_POSITIVE_REGULATION_OF_CHEMOTAXIS | 126 | 0.566594 | 1.532692 | 0.027944 | 0.168917 | 0.994 | 1990 | tags=35%, list=9%, signal=38% |
| GO_POSITIVE_REGULATION_OF_CALCIUM_ION_TRANSPORT_INTO_CYTOSOL | 50 | 0.590516 | 1.573114 | 0.007859 | 0.148055 | 0.989 | 3306 | tags=44%, list=15%, signal=52% |
| GO_NEGATIVE_REGULATION_OF_LEUKOCYTE_CELL_CELL_ADHESION | 111 | 0.616475 | 1.633141 | 0.001988 | 0.136589 | 0.956 | 2068 | tags=38%, list=10%, signal=42% |
| GO_DEFENSE_RESPONSE_TO_BACTERIUM | 209 | 0.434721 | 1.409884 | 0.032946 | 0.258066 | 1 | 2514 | tags=29%, list=12%, signal=32% |
| GO_CYTOKINE_PRODUCTION_INVOLVED_IN_INFLAMMATORY_RESPONSE | 42 | 0.623174 | 1.564865 | 0.01996 | 0.154757 | 0.989 | 1148 | tags=29%, list=5%, signal=30% |
| GO_POSITIVE_REGULATION_OF_T_CELL_MEDIATED_IMMUNITY | 43 | 0.67693 | 1.584709 | 0.013917 | 0.145584 | 0.984 | 3602 | tags=60%, list=17%, signal=72% |
| GO_REGULATION_OF_VIRAL_LIFE_CYCLE | 134 | 0.509807 | 1.626468 | 0.02729 | 0.135898 | 0.962 | 3452 | tags=35%, list=16%, signal=41% |
| GO_LYMPHOCYTE_APOPTOTIC_PROCESS | 70 | 0.65084 | 1.739605 | 0 | 0.240517 | 0.813 | 2077 | tags=34%, list=10%, signal=38% |
| GO_PURINERGIC_RECEPTOR_ACTIVITY | 25 | 0.659924 | 1.479237 | 0.048263 | 0.208839 | 0.999 | 2232 | tags=56%, list=10%, signal=62% |
| GO_POSITIVE_REGULATION_OF_INTERLEUKIN_10_PRODUCTION | 33 | 0.844374 | 1.651867 | 0 | 0.142991 | 0.944 | 1148 | tags=58%, list=5%, signal=61% |
| GO_ACUTE_INFLAMMATORY_RESPONSE_TO_ANTIGENIC_STIMULUS | 18 | 0.782743 | 1.564792 | 0.006085 | 0.153622 | 0.989 | 774 | tags=44%, list=4%, signal=46% |
| GO_POSITIVE_REGULATION_OF_RELEASE_OF_SEQUESTERED_CALCIUM_ION_INTO_CYTOSOL | 36 | 0.639342 | 1.562154 | 0.00996 | 0.154543 | 0.991 | 3306 | tags=47%, list=15%, signal=56% |
| GO_POSITIVE_REGULATION_OF_MONOCYTE_CHEMOTAXIS | 17 | 0.766396 | 1.529833 | 0.025194 | 0.171062 | 0.994 | 1636 | tags=59%, list=8%, signal=64% |
| GO_NEUTROPHIL_MIGRATION | 102 | 0.697523 | 1.596986 | 0.015905 | 0.141743 | 0.98 | 2014 | tags=49%, list=9%, signal=54% |
| GO_POSITIVE_REGULATION_OF_JNK_CASCADE | 124 | 0.445748 | 1.488026 | 0.04142 | 0.20145 | 0.999 | 3092 | tags=27%, list=14%, signal=32% |
| GO_REGULATION_OF_MONOCYTE_CHEMOTAXIS | 23 | 0.749101 | 1.516487 | 0.029014 | 0.180488 | 0.997 | 2077 | tags=57%, list=10%, signal=62% |

| | | | | | | | | |
|---|---|---|---|---|---|---|---|---|
| GO_TYROSINE_PHOSPHORYLATION_OF_STAT_PROTEIN | 79 | 0.502347 | 1.489096 | 0.031008 | 0.201486 | 0.999 | 2648 | tags=38%, list=12%, signal=43% |
| GO_MONONUCLEAR_CELL_MIGRATION | 80 | 0.703346 | 1.611646 | 0.015595 | 0.139726 | 0.971 | 2158 | tags=50%, list=10%, signal=55% |
| GO_NEGATIVE_REGULATION_OF_IMMUNE_RESPONSE | 140 | 0.598235 | 1.618858 | 0.002016 | 0.137282 | 0.968 | 2514 | tags=41%, list=12%, signal=46% |
| GO_NEGATIVE_REGULATION_OF_ADAPTIVE_IMMUNE_RESPONSE | 44 | 0.607749 | 1.543108 | 0.024145 | 0.162067 | 0.993 | 1275 | tags=34%, list=6%, signal=36% |
| GO_NEUROINFLAMMATORY_RESPONSE | 63 | 0.687638 | 1.588274 | 0.011719 | 0.145606 | 0.983 | 1897 | tags=49%, list=9%, signal=54% |
| GO_HOMEOSTASIS_OF_NUMBER_OF_CELLS_WITHIN_A_TISSUE | 27 | 0.645225 | 1.673373 | 0.006061 | 0.171667 | 0.926 | 3516 | tags=48%, list=16%, signal=57% |
| GO_REGULATION_OF_CALCIUM_ION_TRANSPORT | 228 | 0.425793 | 1.413653 | 0.048387 | 0.255828 | 1 | 3306 | tags=32%, list=15%, signal=37% |
| GO_NEGATIVE_REGULATION_OF_LEUKOCYTE_MEDIATED_CYTOTOXICITY | 15 | 0.777744 | 1.587115 | 0.017341 | 0.146296 | 0.983 | 1758 | tags=60%, list=8%, signal=65% |
| GO_DEFENSE_RESPONSE_TO_GRAM_POSITIVE_BACTERIUM | 78 | 0.465921 | 1.398109 | 0.032567 | 0.268418 | 1 | 2236 | tags=33%, list=10%, signal=37% |
| GO_MONOCYTE_CHEMOTAXIS | 59 | 0.741498 | 1.582009 | 0.013619 | 0.145767 | 0.986 | 2077 | tags=56%, list=10%, signal=62% |
| GO_NEGATIVE_REGULATION_OF_CELL_CELL_ADHESION | 159 | 0.548803 | 1.539863 | 0.016 | 0.162553 | 0.993 | 2068 | tags=31%, list=10%, signal=34% |
| GO_NEGATIVE_REGULATION_OF_INTERLEUKIN_2_PRODUCTION | 23 | 0.660505 | 1.493925 | 0.030181 | 0.196846 | 0.999 | 1209 | tags=43%, list=6%, signal=46% |
| GO_POSITIVE_REGULATION_OF_MONONUCLEAR_CELL_MIGRATION | 22 | 0.706162 | 1.517436 | 0.023346 | 0.179368 | 0.997 | 1636 | tags=50%, list=8%, signal=54% |
| GO_POSITIVE_REGULATION_OF_RECEPTOR_SIGNALING_PATHWAY_VIA_STAT | 87 | 0.489641 | 1.432507 | 0.038986 | 0.243151 | 1 | 2980 | tags=41%, list=14%, signal=48% |
| GO_REGULATION_OF_CHEMOTAXIS | 191 | 0.51561 | 1.477487 | 0.049801 | 0.211061 | 0.999 | 2231 | tags=31%, list=10%, signal=35% |
| GO_NEGATIVE_REGULATION_OF_LEUKOCYTE_MEDIATED_IMMUNITY | 46 | 0.674911 | 1.645003 | 0.001976 | 0.137637 | 0.949 | 2026 | tags=48%, list=9%, signal=53% |
| GO_POSITIVE_REGULATION_OF_CALCIUM_ION_TRANSPORT | 112 | 0.473982 | 1.497458 | 0.00994 | 0.194063 | 0.999 | 3306 | tags=36%, list=15%, signal=42% |
| GO_ACTIVATION_OF_PHOSPHOLIPASE_C_ACTIVITY | 31 | 0.605547 | 1.445945 | 0.036 | 0.234851 | 1 | 3535 | tags=55%, list=16%, signal=65% |

| | | | | | | | | |
|---|---|---|---|---|---|---|---|---|
| GO_NEGATIVE_REGULATION_OF_HEMOPOIESIS | 132 | 0.476585 | 1.48807 | 0.034343 | 0.201736 | 0.999 | 2077 | tags=29%, list=10%, signal=32% |
| GO_NEGATIVE_REGULATION_OF_LEUKOCYTE_DIFFERENTIATION | 95 | 0.543435 | 1.580779 | 0.013944 | 0.143908 | 0.988 | 2077 | tags=36%, list=10%, signal=39% |
| GO_VITAMIN_D_METABOLIC_PROCESS | 22 | 0.553086 | 1.461212 | 0.04845 | 0.217894 | 1 | 1234 | tags=27%, list=6%, signal=29% |

**High-expression *SLAMF8* vs. low-expression *SLAMF8* gastric cancer tissues (GSE62254)**

| NAME | SIZE | ES | NES | NOM.p.val | FDR.q.val | FWER.p.val | RANK.AT.MAX | LEADING.EDGE |
|---|---|---|---|---|---|---|---|---|
| GO_DOUBLE_STRANDED_RNA_BINDING | 71 | 0.59326 | 1.955017 | 0 | 0.016286 | 0.299 | 1423 | tags=20%, list=6%, signal=21% |
| GO_T_CELL_RECEPTOR_SIGNALING_PATHWAY | 177 | 0.718104 | 2.07907 | 0 | 0.023697 | 0.096 | 2591 | tags=36%, list=12%, signal=40% |
| GO_ANTIGEN_RECEPTOR_MEDIATED_SIGNALING_PATHWAY | 226 | 0.755765 | 1.99388 | 0 | 0.018044 | 0.217 | 2518 | tags=42%, list=11%, signal=46% |
| GO_NEGATIVE_REGULATION_OF_TYPE_I_INTERFERON_PRODUCTION | 41 | 0.743664 | 1.939196 | 0 | 0.0165 | 0.343 | 1748 | tags=37%, list=8%, signal=40% |
| GO_IMMUNE_RESPONSE_REGULATING_CELL_SURFACE_RECEPTOR_SIGNALING_PATHWAY | 367 | 0.725011 | 1.996541 | 0 | 0.01894 | 0.212 | 1910 | tags=34%, list=9%, signal=37% |
| GO_FC_RECEPTOR_SIGNALING_PATHWAY | 176 | 0.678946 | 2.124753 | 0 | 0.04496 | 0.058 | 3318 | tags=34%, list=15%, signal=39% |
| GO_NEGATIVE_REGULATION_OF_ANTIGEN_RECEPTOR_MEDIATED_SIGNALING_PATHWAY | 28 | 0.851966 | 1.749407 | 0 | 0.028585 | 0.928 | 1610 | tags=50%, list=7%, signal=54% |
| GO_REGULATION_OF_DEFENSE_RESPONSE_TO_VIRUS | 64 | 0.74106 | 1.951909 | 0 | 0.016281 | 0.305 | 2547 | tags=45%, list=12%, signal=51% |
| GO_ALPHA_BETA_T_CELL_DIFFERENTIATION | 95 | 0.767748 | 1.827434 | 0 | 0.0222 | 0.71 | 2640 | tags=58%, list=12%, signal=66% |
| GO_REGULATION_OF_T_CELL_RECEPTOR_SIGNALING_PATHWAY | 38 | 0.813637 | 1.789642 | 0 | 0.024708 | 0.837 | 2348 | tags=53%, list=11%, signal=59% |
| GO_T_HELPER_1_TYPE_IMMUNE_RESPONSE | 41 | 0.83013 | 1.819502 | 0 | 0.022679 | 0.729 | 1794 | tags=61%, list=8%, signal=66% |

| | | | | | | | | |
|---|---|---|---|---|---|---|---|---|
| GO_NEGATIVE_REGULATION_OF_T_CELL_RECEPTOR_SIGNALING_PATHWAY | 20 | 0.827348 | 1.717477 | 0 | 0.033052 | 0.968 | 1610 | tags=45%, list=7%, signal=49% |
| GO_T_CELL_ACTIVATION_INVOLVED_IN_IMMUNE_RESPONSE | 100 | 0.782252 | 1.921796 | 0 | 0.016603 | 0.399 | 1878 | tags=45%, list=9%, signal=49% |
| GO_RESPONSE_TO_TYPE_I_INTERFERON | 88 | 0.778059 | 1.928884 | 0 | 0.016733 | 0.371 | 2449 | tags=49%, list=11%, signal=55% |
| GO_POSITIVE_REGULATION_OF_ALPHA_BETA_T_CELL_ACTIVATION | 58 | 0.788274 | 1.767791 | 0 | 0.026695 | 0.882 | 1699 | tags=53%, list=8%, signal=58% |
| GO_ALPHA_BETA_T_CELL_ACTIVATION | 125 | 0.77952 | 1.824684 | 0 | 0.022315 | 0.717 | 1699 | tags=50%, list=8%, signal=54% |
| GO_POSITIVE_REGULATION_OF_LEUKOCYTE_DIFFERENTIATION | 143 | 0.746741 | 1.886988 | 0 | 0.018935 | 0.508 | 2493 | tags=50%, list=11%, signal=56% |
| GO_NATURAL_KILLER_CELL_ACTIVATION | 77 | 0.746862 | 2.004396 | 0 | 0.018379 | 0.199 | 1984 | tags=34%, list=9%, signal=37% |
| GO_POSITIVE_REGULATION_OF_ALPHA_BETA_T_CELL_DIFFERENTIATION | 45 | 0.77487 | 1.780283 | 0 | 0.025283 | 0.852 | 1699 | tags=51%, list=8%, signal=55% |
| GO_NON_MEMBRANE_SPANNING_PROTEIN_TYROSINE_KINASE_ACTIVITY | 45 | 0.691924 | 1.813227 | 0 | 0.022943 | 0.76 | 2587 | tags=40%, list=12%, signal=45% |
| GO_CD4_POSITIVE_ALPHA_BETA_T_CELL_DIFFERENTIATION | 69 | 0.785206 | 1.842485 | 0 | 0.021272 | 0.666 | 2624 | tags=61%, list=12%, signal=69% |
| GO_LYMPHOCYTE_ACTIVATION_INVOLVED_IN_IMMUNE_RESPONSE | 168 | 0.763967 | 2.023399 | 0 | 0.018187 | 0.167 | 2348 | tags=45%, list=11%, signal=50% |
| GO_POSITIVE_REGULATION_OF_CELL_ACTIVATION | 305 | 0.775334 | 1.901967 | 0 | 0.017312 | 0.454 | 1787 | tags=46%, list=8%, signal=49% |
| GO_T_CELL_ACTIVATION | 432 | 0.747336 | 1.991544 | 0 | 0.017888 | 0.221 | 1794 | tags=39%, list=8%, signal=42% |
| GO_REGULATION_OF_ANTIGEN_RECEPTOR_MEDIATED_SIGNALING_PATHWAY | 61 | 0.819349 | 1.797942 | 0 | 0.023534 | 0.813 | 1843 | tags=52%, list=8%, signal=57% |
| GO_POSITIVE_REGULATION_OF_LYMPHOCYTE_DIFFERENTIATION | 91 | 0.798044 | 1.858897 | 0 | 0.019753 | 0.609 | 2002 | tags=56%, list=9%, signal=61% |
| GO_LYMPHOCYTE_DIFFERENTIATION | 336 | 0.730655 | 1.947066 | 0 | 0.016482 | 0.32 | 2860 | tags=46%, list=13%, signal=53% |
| GO_DEFENSE_RESPONSE_TO_VIRUS | 218 | 0.73019 | 1.983906 | 0 | 0.017648 | 0.235 | 2547 | tags=43%, list=12%, signal=48% |
| GO_REGULATION_OF_LEUKOCYTE_DEGRANULATION | 42 | 0.797907 | 1.802652 | 0 | 0.023376 | 0.796 | 2493 | tags=52%, list=11%, signal=59% |

| | | | | | | | | |
|---|---|---|---|---|---|---|---|---|
| GO_POSITIVE_REGULATION_OF_INTERFERON_ALPHA_PRODUCTION | 22 | 0.835511 | 1.756272 | 0 | 0.02752 | 0.911 | 1461 | tags=45%, list=7%, signal=49% |
| GO_RESPONSE_TO_VIRUS | 295 | 0.705234 | 1.990985 | 0 | 0.017436 | 0.222 | 2567 | tags=39%, list=12%, signal=44% |
| GO_REGULATION_OF_ALPHA_BETA_T_CELL_DIFFERENTIATION | 60 | 0.777516 | 1.867116 | 0 | 0.019006 | 0.578 | 1878 | tags=53%, list=9%, signal=58% |
| GO_REGULATION_OF_DEFENSE_RESPONSE_TO_VIRUS_BY_VIRUS | 30 | 0.708149 | 1.877816 | 0 | 0.018767 | 0.544 | 2860 | tags=37%, list=13%, signal=42% |
| GO_CD4_POSITIVE_ALPHA_BETA_T_CELL_ACTIVATION | 83 | 0.774985 | 1.847208 | 0 | 0.021269 | 0.649 | 1693 | tags=51%, list=8%, signal=55% |
| GO_REGULATION_OF_NATURAL_KILLER_CELL_ACTIVATION | 32 | 0.791045 | 1.868744 | 0 | 0.019178 | 0.574 | 1984 | tags=47%, list=9%, signal=51% |
| GO_POSITIVE_REGULATION_OF_LEUKOCYTE_CELL_CELL_ADHESION | 203 | 0.78428 | 1.924573 | 0 | 0.016716 | 0.39 | 1843 | tags=49%, list=8%, signal=53% |
| GO_INTERFERON_GAMMA_PRODUCTION | 100 | 0.826651 | 1.880726 | 0 | 0.018798 | 0.527 | 1835 | tags=55%, list=8%, signal=60% |
| GO_ACTIVATED_T_CELL_PROLIFERATION | 39 | 0.735605 | 1.911369 | 0 | 0.016556 | 0.421 | 1693 | tags=38%, list=8%, signal=42% |
| GO_CELLULAR_RESPONSE_TO_INTERFERON_BETA | 18 | 0.866009 | 1.764844 | 0 | 0.026811 | 0.89 | 835 | tags=39%, list=4%, signal=40% |
| GO_ACTIVATION_OF_INNATE_IMMUNE_RESPONSE | 290 | 0.689124 | 2.075543 | 0 | 0.020247 | 0.1 | 2773 | tags=36%, list=13%, signal=40% |
| GO_MYD88_INDEPENDENT_TOLL_LIKE_RECEPTOR_SIGNALING_PATHWAY | 32 | 0.757867 | 1.797119 | 0 | 0.023704 | 0.817 | 1683 | tags=34%, list=8%, signal=37% |
| GO_INTERFERON_ALPHA_PRODUCTION | 29 | 0.846866 | 1.809526 | 0 | 0.023308 | 0.771 | 1461 | tags=48%, list=7%, signal=52% |
| GO_T_CELL_DIFFERENTIATION | 226 | 0.754506 | 1.920971 | 0 | 0.01639 | 0.4 | 1878 | tags=42%, list=9%, signal=45% |
| GO_POSITIVE_REGULATION_OF_INTERFERON_BETA_PRODUCTION | 30 | 0.760581 | 1.8028 | 0 | 0.023398 | 0.796 | 1744 | tags=37%, list=8%, signal=40% |
| GO_T_CELL_PROLIFERATION | 172 | 0.75633 | 1.989193 | 0 | 0.017241 | 0.226 | 1794 | tags=42%, list=8%, signal=45% |
| GO_TUMOR_NECROSIS_FACTOR_BIOSYNTHETIC_PROCESS | 28 | 0.858437 | 1.688591 | 0 | 0.037116 | 0.99 | 718 | tags=39%, list=3%, signal=41% |
| GO_RESPONSE_TO_INTERFERON_ALPHA | 20 | 0.857017 | 1.718355 | 0 | 0.032905 | 0.968 | 1922 | tags=60%, list=9%, signal=66% |

| | | | | | | | | |
|---|---|---|---|---|---|---|---|---|
| GO_REGULATION_OF_ALPHA_BETA_T_CELL_ACTIVATION | 83 | 0.793679 | 1.850895 | 0 | 0.02091 | 0.638 | 1699 | tags=54%, list=8%, signal=59% |
| GO_POSITIVE_REGULATION_OF_RESPONSE_TO_BIOTIC_STIMULUS | 361 | 0.705319 | 2.066495 | 0 | 0.020998 | 0.109 | 2591 | tags=37%, list=12%, signal=41% |
| GO_T_CELL_DIFFERENTIATION_INVOLVED_IN_IMMUNE_RESPONSE | 65 | 0.79352 | 1.804832 | 0 | 0.023347 | 0.792 | 1878 | tags=54%, list=9%, signal=59% |
| GO_LEUKOCYTE_DIFFERENTIATION | 492 | 0.698004 | 1.963433 | 0 | 0.01726 | 0.279 | 2508 | tags=39%, list=11%, signal=44% |
| GO_REGULATION_OF_T_HELPER_1_TYPE_IMMUNE_RESPONSE | 26 | 0.872198 | 1.759716 | 0 | 0.027029 | 0.905 | 1794 | tags=77%, list=8%, signal=84% |
| GO_REGULATION_OF_OSTEOCLAST_DIFFERENTIATION | 61 | 0.728227 | 1.78146 | 0 | 0.025214 | 0.849 | 2503 | tags=44%, list=11%, signal=50% |
| GO_POSITIVE_REGULATION_OF_HEMOPOIESIS | 178 | 0.705672 | 1.936022 | 0 | 0.016707 | 0.35 | 2659 | tags=46%, list=12%, signal=51% |
| GO_POSITIVE_REGULATION_OF_T_HELPER_1_TYPE_IMMUNE_RESPONSE | 19 | 0.895768 | 1.728396 | 0 | 0.031311 | 0.957 | 1794 | tags=84%, list=8%, signal=92% |
| GO_REGULATION_OF_MYELOID_LEUKOCYTE_DIFFERENTIATION | 110 | 0.676643 | 1.849218 | 0 | 0.02103 | 0.646 | 2986 | tags=44%, list=14%, signal=50% |
| GO_ADAPTIVE_IMMUNE_RESPONSE | 375 | 0.802703 | 1.923011 | 0 | 0.016641 | 0.395 | 2002 | tags=51%, list=9%, signal=55% |
| GO_REGULATION_OF_DEFENSE_RESPONSE_TO_VIRUS_BY_HOST | 32 | 0.78512 | 1.793445 | 0 | 0.024017 | 0.829 | 2547 | tags=56%, list=12%, signal=64% |
| GO_PHAGOCYTOSIS | 244 | 0.728161 | 1.940916 | 0 | 0.016514 | 0.337 | 2111 | tags=38%, list=10%, signal=42% |
| GO_REGULATION_OF_LEUKOCYTE_DIFFERENTIATION | 259 | 0.719716 | 1.957875 | 0 | 0.016549 | 0.292 | 2503 | tags=44%, list=11%, signal=49% |
| GO_TUMOR_NECROSIS_FACTOR_SUPERFAMILY_CYTOKINE_PRODUCTION | 139 | 0.787494 | 1.817686 | 0 | 0.02268 | 0.739 | 1787 | tags=43%, list=8%, signal=47% |
| GO_REGULATION_OF_RESPONSE_TO_BIOTIC_STIMULUS | 475 | 0.71857 | 2.079365 | 0 | 0.024802 | 0.094 | 2591 | tags=39%, list=12%, signal=43% |
| GO_ANTIGEN_PROCESSING_AND_PRESENTATION | 198 | 0.672086 | 2.093362 | 0 | 0.024053 | 0.083 | 2130 | tags=29%, list=10%, signal=32% |
| GO_ADAPTIVE_IMMUNE_RESPONSE_BASED_ON_SOMATIC_RECOMBINATION_OF_IMMUNE_RECEPTORS_BUILT_FROM_IMMUNOGLOBULIN_SUPERFAMILY_DOMAINS | 240 | 0.784206 | 1.924668 | 0 | 0.016808 | 0.39 | 2394 | tags=51%, list=11%, signal=56% |

| | | | | | | | | |
|---|---|---|---|---|---|---|---|---|
| GO_REGULATION_OF_T_CELL_ACTIVATION | 291 | 0.759037 | 1.990 52 | 0 | 0.017159 | 0.223 | 1878 | tags=43%, list=9%, signal=46% |
| GO_POSITIVE_REGULATION_OF_CYTOKINE_PRODUCTIO N | 421 | 0.734962 | 1.996 229 | 0 | 0.018569 | 0.212 | 1855 | tags=37%, list=8%, signal=39% |
| GO_REGULATION_OF_HEMOPOIESIS | 421 | 0.654756 | 2.075 563 | 0 | 0.021512 | 0.1 | 2379 | tags=33%, list=11%, signal=36% |
| GO_POSITIVE_REGULATION_OF_CELL_CELL_ADHESION | 239 | 0.744603 | 1.927 715 | 0 | 0.016548 | 0.378 | 1878 | tags=45%, list=9%, signal=48% |
| GO_POSITIVE_REGULATION_OF_IMMUNE_EFFECTOR_PR OCESS | 189 | 0.767986 | 1.927 852 | 0 | 0.016648 | 0.378 | 1984 | tags=44%, list=9%, signal=48% |
| GO_POSITIVE_REGULATION_OF_INTERFERON_GAMMA_P RODUCTION | 57 | 0.858161 | 1.800 882 | 0 | 0.023555 | 0.804 | 1835 | tags=63%, list=8%, signal=69% |
| GO_POSITIVE_REGULATION_OF_DEFENSE_RESPONSE_TO _VIRUS_BY_HOST | 22 | 0.780808 | 1.804 955 | 0.0 02 04 5 | 0.02338 | 0.792 | 2547 | tags=59%, list=12%, signal=67% |
| GO_IMMUNOLOGICAL_SYNAPSE | 35 | 0.797977 | 1.771 566 | 0 | 0.026406 | 0.875 | 1602 | tags=40%, list=7%, signal=43% |
| GO_POSITIVE_REGULATION_OF_MULTI_ORGANISM_PRO CESS | 494 | 0.660645 | 2.133 885 | 0 | 0.05398 | 0.05 | 2663 | tags=32%, list=12%, signal=36% |
| GO_T_CELL_MEDIATED_IMMUNITY | 93 | 0.784674 | 1.903 693 | 0 | 0.01713 | 0.449 | 1984 | tags=47%, list=9%, signal=52% |
| GO_REGULATION_OF_NATURAL_KILLER_CELL_MEDIATE D_IMMUNITY | 36 | 0.823817 | 1.706 575 | 0 | 0.034345 | 0.974 | 2348 | tags=56%, list=11%, signal=62% |
| GO_REGULATION_OF_MACROPHAGE_MIGRATION | 35 | 0.781408 | 1.676 999 | 0 | 0.039437 | 0.993 | 1463 | tags=46%, list=7%, signal=49% |
| GO_LEUKOCYTE_CELL_CELL_ADHESION | 313 | 0.754614 | 1.940 054 | 0 | 0.016574 | 0.34 | 1878 | tags=45%, list=9%, signal=48% |
| GO_CYTOKINE_SECRETION | 217 | 0.751916 | 1.858 196 | 0 | 0.019853 | 0.61 | 1844 | tags=44%, list=8%, signal=47% |
| GO_LYMPHOCYTE_MEDIATED_IMMUNITY | 235 | 0.791206 | 1.939 942 | 0 | 0.01643 | 0.34 | 2002 | tags=46%, list=9%, signal=50% |
| GO_RESPONSE_TO_INTERFERON_GAMMA | 166 | 0.781386 | 1.950 556 | 0 | 0.016333 | 0.308 | 2774 | tags=58%, list=13%, signal=66% |

| | | | | | | | | |
|---|---|---|---|---|---|---|---|---|
| GO_CD8_POSITIVE_ALPHA_BETA_T_CELL_ACTIVATION | 19 | 0.848101 | 1.589159 | 0.0042228 | 0.063196 | 1 | 2420 | tags=68%, list=11%, signal=77% |
| GO_SYNCYTIUM_FORMATION | 50 | 0.704486 | 1.942645 | 0 | 0.016592 | 0.334 | 2493 | tags=38%, list=11%, signal=43% |
| GO_REGULATION_OF_IMMUNE_EFFECTOR_PROCESS | 360 | 0.742376 | 1.959976 | 0 | 0.017553 | 0.29 | 2566 | tags=45%, list=12%, signal=50% |
| GO_MYOBLAST_FUSION | 37 | 0.69498 | 1.894916 | 0 | 0.018179 | 0.476 | 2493 | tags=38%, list=11%, signal=43% |
| GO_REGULATION_OF_LYMPHOCYTE_ACTIVATION | 398 | 0.755279 | 1.976847 | 0 | 0.017815 | 0.249 | 1787 | tags=41%, list=8%, signal=44% |
| GO_LEUKOCYTE_PROLIFERATION | 280 | 0.752767 | 1.971418 | 0 | 0.018249 | 0.264 | 1794 | tags=40%, list=8%, signal=43% |
| GO_PRODUCTION_OF_MOLECULAR_MEDIATOR_OF_IMMUNE_RESPONSE | 190 | 0.738759 | 1.924092 | 0 | 0.016693 | 0.394 | 1982 | tags=40%, list=9%, signal=44% |
| GO_POSITIVE_REGULATION_OF_T_CELL_PROLIFERATION | 87 | 0.804347 | 1.886301 | 0 | 0.018748 | 0.509 | 1693 | tags=48%, list=8%, signal=52% |
| GO_POSITIVE_REGULATION_OF_MYELOID_LEUKOCYTE_DIFFERENTIATION | 54 | 0.704477 | 1.833127 | 0 | 0.022172 | 0.694 | 2986 | tags=48%, list=14%, signal=56% |
| GO_POSITIVE_REGULATION_OF_TUMOR_NECROSIS_FACTOR_SUPERFAMILY_CYTOKINE_PRODUCTION | 79 | 0.818294 | 1.765371 | 0 | 0.026949 | 0.89 | 1787 | tags=47%, list=8%, signal=51% |
| GO_POSITIVE_REGULATION_OF_CD4_POSITIVE_ALPHA_BETA_T_CELL_ACTIVATION | 35 | 0.841239 | 1.726328 | 0 | 0.031595 | 0.96 | 1693 | tags=63%, list=8%, signal=68% |
| GO_REGULATION_OF_LEUKOCYTE_MEDIATED_CYTOTOXICITY | 66 | 0.811243 | 1.746319 | 0 | 0.02889 | 0.93 | 1949 | tags=53%, list=9%, signal=58% |
| GO_INTERLEUKIN_12_PRODUCTION | 56 | 0.83612 | 1.787054 | 0 | 0.024841 | 0.84 | 1835 | tags=54%, list=8%, signal=58% |
| GO_REGULATION_OF_LEUKOCYTE_MIGRATION | 183 | 0.752287 | 1.830642 | 0 | 0.022243 | 0.701 | 2129 | tags=47%, list=10%, signal=52% |
| GO_INTERLEUKIN_8_SECRETION | 30 | 0.836872 | 1.691705 | 0 | 0.036717 | 0.985 | 1694 | tags=50%, list=8%, signal=54% |
| GO_REGULATION_OF_LEUKOCYTE_MEDIATED_IMMUNITY | 183 | 0.793738 | 1.965742 | 0 | 0.017769 | 0.277 | 1984 | tags=46%, list=9%, signal=51% |
| GO_T_CELL_MIGRATION | 61 | 0.839423 | 1.839955 | 0 | 0.02138 | 0.672 | 1787 | tags=57%, list=8%, signal=62% |

| | | | | | | | | |
|---|---|---|---|---|---|---|---|---|
| GO_REGULATION_OF_MYELOID_LEUKOCYTE_MEDIATED_IMMUNITY | 51 | 0.794797 | 1.833282 | 0 | 0.022184 | 0.694 | 1861 | tags=47%, list=8%, signal=51% |
| GO_REGULATION_OF_RESPONSE_TO_CYTOKINE_STIMULUS | 159 | 0.695834 | 2.027518 | 0 | 0.018326 | 0.158 | 2646 | tags=40%, list=12%, signal=45% |
| GO_LYMPHOCYTE_HOMEOSTASIS | 61 | 0.739533 | 1.953599 | 0 | 0.016463 | 0.302 | 2691 | tags=46%, list=12%, signal=52% |
| GO_NEGATIVE_REGULATION_OF_CYTOKINE_PRODUCTION | 251 | 0.714429 | 1.945353 | 0 | 0.016525 | 0.329 | 2348 | tags=41%, list=11%, signal=46% |
| GO_FC_RECEPTOR_MEDIATED_STIMULATORY_SIGNALING_PATHWAY | 83 | 0.700023 | 1.928083 | 0 | 0.016873 | 0.377 | 3284 | tags=42%, list=15%, signal=49% |
| GO_LYMPHOCYTE_MIGRATION | 100 | 0.820289 | 1.815523 | 0 | 0.022802 | 0.747 | 1787 | tags=55%, list=8%, signal=60% |
| GO_REGULATION_OF_CD4_POSITIVE_ALPHA_BETA_T_CELL_DIFFERENTIATION | 44 | 0.827268 | 1.810852 | 0 | 0.023126 | 0.767 | 1878 | tags=61%, list=9%, signal=67% |
| GO_LEUKOCYTE_MEDIATED_CYTOTOXICITY | 96 | 0.78362 | 1.812567 | 0 | 0.022982 | 0.763 | 1949 | tags=46%, list=9%, signal=50% |
| GO_INTERLEUKIN_8_PRODUCTION | 74 | 0.763168 | 1.823548 | 0 | 0.022404 | 0.72 | 1694 | tags=39%, list=8%, signal=42% |
| GO_ANTIGEN_PROCESSING_AND_PRESENTATION_OF_EXOGENOUS_PEPTIDE_ANTIGEN_VIA_MHC_CLASS_I | 68 | 0.747054 | 2.061956 | 0 | 0.017683 | 0.111 | 2234 | tags=29%, list=10%, signal=33% |
| GO_REGULATION_OF_LYMPHOCYTE_DIFFERENTIATION | 162 | 0.758995 | 1.957642 | 0 | 0.016464 | 0.292 | 2073 | tags=47%, list=9%, signal=51% |
| GO_POSITIVE_REGULATION_OF_LEUKOCYTE_PROLIFERATION | 127 | 0.778547 | 1.912256 | 0 | 0.016568 | 0.421 | 1735 | tags=45%, list=8%, signal=48% |
| GO_REGULATION_OF_T_CELL_DIFFERENTIATION | 131 | 0.76304 | 1.913832 | 0 | 0.016586 | 0.42 | 2073 | tags=48%, list=9%, signal=53% |
| GO_POSITIVE_REGULATION_OF_CD4_POSITIVE_ALPHA_BETA_T_CELL_DIFFERENTIATION | 29 | 0.852513 | 1.712143 | 0 | 0.033668 | 0.971 | 1693 | tags=66%, list=8%, signal=71% |
| GO_REGULATION_OF_LYMPHOCYTE_MIGRATION | 58 | 0.783609 | 1.805821 | 0 | 0.023349 | 0.786 | 1787 | tags=47%, list=8%, signal=51% |
| GO_T_CELL_SELECTION | 47 | 0.80924 | 1.710598 | 0 | 0.033773 | 0.973 | 2640 | tags=62%, list=12%, signal=70% |
| GO_POSITIVE_REGULATION_OF_LEUKOCYTE_MIGRATION | 123 | 0.79414 | 1.851023 | 0 | 0.020972 | 0.638 | 2129 | tags=50%, list=10%, signal=55% |
| GO_LIPOPOLYSACCHARIDE_MEDIATED_SIGNALING_PATHWAY | 56 | 0.77814 | 1.885409 | 0 | 0.018687 | 0.511 | 2537 | tags=52%, list=12%, signal=58% |

| | | | | | | | | |
|---|---|---|---|---|---|---|---|---|
| GO_NEGATIVE_REGULATION_OF_REGULATED_SECRETORY_PATHWAY | 21 | 0.789089 | 1.643194 | 0.00404 | 0.046879 | 1 | 1704 | tags=43%, list=8%, signal=46% |
| GO_INTERLEUKIN_1_BETA_PRODUCTION | 82 | 0.789092 | 1.738878 | 0 | 0.029428 | 0.941 | 1748 | tags=54%, list=8%, signal=58% |
| GO_REGULATION_OF_CELL_KILLING | 87 | 0.793013 | 1.781918 | 0 | 0.025232 | 0.849 | 2348 | tags=51%, list=11%, signal=56% |
| GO_POSITIVE_REGULATION_OF_CYTOKINE_SECRETION | 128 | 0.76589 | 1.786933 | 0 | 0.024773 | 0.84 | 1941 | tags=45%, list=9%, signal=49% |
| GO_NEGATIVE_REGULATION_OF_IMMUNE_SYSTEM_PROCESS | 409 | 0.704167 | 1.96605 | 0 | 0.017927 | 0.276 | 2503 | tags=41%, list=11%, signal=45% |
| GO_REGULATION_OF_SYNCYTIUM_FORMATION_BY_PLASMA_MEMBRANE_FUSION | 27 | 0.783439 | 1.773433 | 0 | 0.026265 | 0.87 | 2493 | tags=52%, list=11%, signal=58% |
| GO_NIK_NF_KAPPAB_SIGNALING | 156 | 0.657684 | 2.135638 | 0 | 0.07848 | 0.049 | 2891 | tags=32%, list=13%, signal=37% |
| GO_ANTIGEN_PROCESSING_AND_PRESENTATION_OF_PEPTIDE_ANTIGEN_VIA_MHC_CLASS_I | 89 | 0.703419 | 2.045506 | 0 | 0.017724 | 0.132 | 2234 | tags=30%, list=10%, signal=34% |
| GO_POSITIVE_REGULATION_OF_NATURAL_KILLER_CELL_MEDIATED_CYTOTOXICITY | 19 | 0.894618 | 1.628814 | 0 | 0.050755 | 1 | 1262 | tags=53%, list=6%, signal=56% |
| GO_INTERFERON_GAMMA_MEDIATED_SIGNALING_PATHWAY | 70 | 0.773314 | 1.815929 | 0 | 0.022797 | 0.747 | 2234 | tags=56%, list=10%, signal=62% |
| GO_POSITIVE_REGULATION_OF_CELL_ADHESION | 376 | 0.679959 | 1.911566 | 0 | 0.016627 | 0.421 | 2099 | tags=38%, list=10%, signal=41% |
| GO_POSITIVE_REGULATION_OF_TUMOR_NECROSIS_FACTOR_BIOSYNTHETIC_PROCESS | 20 | 0.906751 | 1.696208 | 0 | 0.035772 | 0.983 | 718 | tags=45%, list=3%, signal=46% |
| GO_REGULATION_OF_MYELOID_CELL_DIFFERENTIATION | 208 | 0.614475 | 1.932516 | 0 | 0.016662 | 0.359 | 2986 | tags=36%, list=14%, signal=41% |
| GO_POSITIVE_REGULATION_OF_OSTEOCLAST_DIFFERENTIATION | 26 | 0.736571 | 1.712023 | 0 | 0.033654 | 0.971 | 1693 | tags=38%, list=8%, signal=42% |
| GO_I_KAPPAB_PHOSPHORYLATION | 18 | 0.836097 | 1.868993 | 0 | 0.019271 | 0.572 | 1748 | tags=39%, list=8%, signal=42% |
| GO_POSITIVE_REGULATION_OF_NATURAL_KILLER_CELL_MEDIATED_IMMUNITY | 22 | 0.86151 | 1.61853 | 0 | 0.054254 | 1 | 1809 | tags=55%, list=8%, signal=59% |
| GO_PRODUCTION_OF_MOLECULAR_MEDIATOR_INVOLVED_IN_INFLAMMATORY_RESPONSE | 63 | 0.739126 | 1.814857 | 0 | 0.022816 | 0.748 | 2547 | tags=44%, list=12%, signal=50% |

| | | | | | | | | |
|---|---|---|---|---|---|---|---|---|
| GO_POSITIVE_REGULATION_OF_LEUKOCYTE_MEDIATED_CYTOTOXICITY | 44 | 0.825352 | 1.674626 | 0 | 0.039883 | 0.993 | 1949 | tags=55%, list=9%, signal=60% |
| GO_REGULATION_OF_MAST_CELL_ACTIVATION | 38 | 0.749776 | 1.725015 | 0 | 0.031824 | 0.96 | 2167 | tags=47%, list=10%, signal=52% |
| GO_REGULATION_OF_MACROPHAGE_CHEMOTAXIS | 24 | 0.780567 | 1.691406 | 0.0020045 | 0.036738 | 0.985 | 798 | tags=33%, list=4%, signal=35% |
| GO_INTERLEUKIN_1_PRODUCTION | 95 | 0.779269 | 1.772164 | 0 | 0.026378 | 0.873 | 1748 | tags=52%, list=8%, signal=56% |
| GO_NEGATIVE_REGULATION_OF_EXOCYTOSIS | 28 | 0.745422 | 1.69772 | 0 | 0.035498 | 0.981 | 1704 | tags=36%, list=8%, signal=39% |
| GO_MHC_PROTEIN_BINDING | 23 | 0.835355 | 1.652874 | 0.0020062 | 0.044927 | 1 | 1189 | tags=48%, list=5%, signal=51% |
| GO_T_CELL_MEDIATED_CYTOTOXICITY | 37 | 0.76858 | 1.703915 | 0.0020037 | 0.034582 | 0.975 | 1949 | tags=51%, list=9%, signal=56% |
| GO_POSITIVE_REGULATION_OF_CYTOKINE_BIOSYNTHETIC_PROCESS | 66 | 0.839014 | 1.829462 | 0 | 0.022055 | 0.704 | 1537 | tags=48%, list=7%, signal=52% |
| GO_CELLULAR_DEFENSE_RESPONSE | 48 | 0.831501 | 1.671595 | 0 | 0.040551 | 0.994 | 1879 | tags=56%, list=9%, signal=61% |
| GO_REGULATION_OF_T_CELL_MIGRATION | 41 | 0.81156 | 1.810761 | 0 | 0.023093 | 0.769 | 2101 | tags=51%, list=10%, signal=57% |
| GO_TUMOR_NECROSIS_FACTOR_MEDIATED_SIGNALING_PATHWAY | 150 | 0.592777 | 1.969041 | 0 | 0.018553 | 0.271 | 2547 | tags=28%, list=12%, signal=31% |
| GO_RESPIRATORY_BURST | 30 | 0.825143 | 1.712176 | 0 | 0.033699 | 0.971 | 2079 | tags=50%, list=9%, signal=55% |
| GO_MYELOID_LEUKOCYTE_DIFFERENTIATION | 196 | 0.664277 | 1.909455 | 0 | 0.016501 | 0.426 | 2503 | tags=38%, list=11%, signal=43% |
| GO_DENDRITIC_CELL_DIFFERENTIATION | 39 | 0.804437 | 1.868333 | 0 | 0.019164 | 0.575 | 2847 | tags=59%, list=13%, signal=68% |
| GO_POSITIVE_REGULATION_OF_LYMPHOCYTE_MEDIATED_IMMUNITY | 89 | 0.808073 | 1.865433 | 0 | 0.019237 | 0.585 | 1984 | tags=49%, list=9%, signal=54% |

| | | | | | | | | |
|---|---|---|---|---|---|---|---|---|
| GO_REGULATION_OF_T_HELPER_CELL_DIFFERENTIATION | 33 | 0.804379 | 1.814603 | 0 | 0.022833 | 0.749 | 1878 | tags=58%, list=9%, signal=63% |
| GO_LEUKOCYTE_ACTIVATION_INVOLVED_IN_INFLAMMATORY_RESPONSE | 42 | 0.887616 | 1.641745 | 0 | 0.047138 | 1 | 1463 | tags=67%, list=7%, signal=71% |
| GO_TYPE_I_INTERFERON_PRODUCTION | 119 | 0.689816 | 2.048374 | 0 | 0.017887 | 0.129 | 2547 | tags=33%, list=12%, signal=37% |
| GO_INTERLEUKIN_2_PRODUCTION | 62 | 0.767136 | 1.753969 | 0.00207 | 0.027728 | 0.916 | 1564 | tags=47%, list=7%, signal=50% |
| GO_REGULATION_OF_CELL_CELL_ADHESION | 375 | 0.692488 | 1.958907 | 0 | 0.016744 | 0.291 | 1878 | tags=37%, list=9%, signal=40% |
| GO_REGULATION_OF_CD4_POSITIVE_ALPHA_BETA_T_CELL_ACTIVATION | 54 | 0.814371 | 1.815482 | 0 | 0.022737 | 0.747 | 1878 | tags=59%, list=9%, signal=65% |
| GO_RESPONSE_TO_AMYLOID_BETA | 45 | 0.822319 | 1.748974 | 0 | 0.028611 | 0.928 | 1536 | tags=49%, list=7%, signal=52% |
| GO_INTERFERON_BETA_PRODUCTION | 49 | 0.735619 | 1.898888 | 0 | 0.017545 | 0.46 | 2547 | tags=41%, list=12%, signal=46% |
| GO_REGULATION_OF_LYMPHOCYTE_MEDIATED_IMMUNITY | 133 | 0.799937 | 1.949071 | 0 | 0.016264 | 0.312 | 1984 | tags=47%, list=9%, signal=51% |
| GO_RESPONSE_TO_INTERFERON_BETA | 26 | 0.865676 | 1.839537 | 0 | 0.02142 | 0.675 | 1941 | tags=58%, list=9%, signal=63% |
| GO_CELL_KILLING | 146 | 0.742431 | 1.855253 | 0 | 0.020381 | 0.619 | 2400 | tags=41%, list=11%, signal=46% |
| GO_MYD88_DEPENDENT_TOLL_LIKE_RECEPTOR_SIGNALING_PATHWAY | 35 | 0.850245 | 1.703173 | 0 | 0.034525 | 0.977 | 2284 | tags=54%, list=10%, signal=60% |
| GO_POSITIVE_REGULATION_OF_DEFENSE_RESPONSE | 458 | 0.709903 | 2.015769 | 0 | 0.018143 | 0.178 | 2343 | tags=36%, list=11%, signal=40% |
| GO_POSITIVE_REGULATION_OF_LEUKOCYTE_MEDIATED_IMMUNITY | 117 | 0.784342 | 1.922574 | 0 | 0.016561 | 0.396 | 1984 | tags=44%, list=9%, signal=48% |
| GO_POSITIVE_REGULATION_OF_MACROPHAGE_ACTIVATION | 22 | 0.820509 | 1.654143 | 0.002075 | 0.044616 | 1 | 1439 | tags=55%, list=7%, signal=58% |
| GO_POSITIVE_REGULATION_OF_CELL_KILLING | 57 | 0.808597 | 1.710659 | 0 | 0.033805 | 0.973 | 1949 | tags=47%, list=9%, signal=52% |

| | | | | | | | | |
|---|---|---|---|---|---|---|---|---|
| GO_B_CELL_ACTIVATION_INVOLVED_IN_IMMUNE_RESPONSE | 67 | 0.766306 | 1.964694 | 0 | 0.017422 | 0.278 | 2344 | tags=45%, list=11%, signal=50% |
| GO_LEUKOCYTE_HOMEOSTASIS | 83 | 0.780179 | 1.889945 | 0 | 0.018841 | 0.496 | 2129 | tags=47%, list=10%, signal=52% |
| GO_INTERLEUKIN_10_PRODUCTION | 50 | 0.84242 | 1.769502 | 0 | 0.026575 | 0.879 | 1693 | tags=56%, list=8%, signal=61% |
| GO_FICOLIN_1_RICH_GRANULE_MEMBRANE | 57 | 0.789797 | 1.897648 | 0 | 0.017625 | 0.467 | 1794 | tags=40%, list=8%, signal=44% |
| GO_REGULATION_OF_MYOBLAST_FUSION | 20 | 0.770395 | 1.721433 | 0 | 0.032572 | 0.964 | 2493 | tags=50%, list=11%, signal=56% |
| GO_LEUKOCYTE_MIGRATION | 413 | 0.74811 | 1.870771 | 0 | 0.019234 | 0.563 | 2365 | tags=48%, list=11%, signal=52% |
| GO_LEUKOCYTE_CHEMOTAXIS | 200 | 0.798099 | 1.816334 | 0 | 0.022828 | 0.747 | 2101 | tags=53%, list=10%, signal=58% |
| GO_PHAGOCYTIC_CUP | 24 | 0.843301 | 1.727441 | 0 | 0.031315 | 0.958 | 2095 | tags=63%, list=10%, signal=69% |
| GO_POSITIVE_REGULATION_OF_INTERLEUKIN_12_PRODUCTION | 35 | 0.847839 | 1.776342 | 0 | 0.025866 | 0.864 | 1835 | tags=51%, list=8%, signal=56% |
| GO_REGULATION_OF_LEUKOCYTE_PROLIFERATION | 206 | 0.762026 | 1.931267 | 0 | 0.016503 | 0.363 | 1735 | tags=42%, list=8%, signal=45% |
| GO_VIRAL_GENOME_REPLICATION | 113 | 0.622485 | 1.93679 | 0 | 0.01678 | 0.349 | 2534 | tags=33%, list=12%, signal=37% |
| GO_POSITIVE_REGULATION_OF_NF_KAPPAB_TRANSCRIPTION_FACTOR_ACTIVITY | 143 | 0.712442 | 2.051414 | 0 | 0.018671 | 0.127 | 1861 | tags=33%, list=8%, signal=36% |
| GO_MACROPHAGE_ACTIVATION | 82 | 0.833666 | 1.743377 | 0 | 0.029126 | 0.936 | 2196 | tags=59%, list=10%, signal=65% |
| GO_OSTEOCLAST_DIFFERENTIATION | 94 | 0.672125 | 1.805594 | 0 | 0.02337 | 0.789 | 2503 | tags=40%, list=11%, signal=45% |
| GO_NITRIC_OXIDE_SYNTHASE_BIOSYNTHETIC_PROCESS | 18 | 0.877789 | 1.634011 | 0.004219 | 0.049017 | 1 | 999 | tags=56%, list=5%, signal=58% |
| GO_POSITIVE_REGULATION_OF_T_CELL_MIGRATION | 29 | 0.825386 | 1.721895 | 0.002096 | 0.032613 | 0.963 | 2640 | tags=62%, list=12%, signal=70% |

| | | | | | | | | |
|---|---|---|---|---|---|---|---|---|
| GO_NEGATIVE_REGULATION_OF_INTERLEUKIN_10_PRODUCTION | 17 | 0.863167 | 1.60176 | 0 | 0.05914 | 1 | 1693 | tags=65%, list=8%, signal=70% |
| GO_B_CELL_PROLIFERATION | 92 | 0.783837 | 1.898422 | 0 | 0.017588 | 0.466 | 1784 | tags=37%, list=8%, signal=40% |
| GO_REGULATION_OF_B_CELL_RECEPTOR_SIGNALING_PATHWAY | 26 | 0.843848 | 1.719784 | 0 | 0.032913 | 0.965 | 1555 | tags=58%, list=7%, signal=62% |
| GO_T_CELL_CYTOKINE_PRODUCTION | 40 | 0.820193 | 1.825456 | 0 | 0.022267 | 0.716 | 1878 | tags=50%, list=9%, signal=55% |
| GO_ANTIGEN_BINDING | 44 | 0.842617 | 1.715769 | 0 | 0.033157 | 0.969 | 1992 | tags=66%, list=9%, signal=72% |
| GO_NATURAL_KILLER_CELL_MEDIATED_IMMUNITY | 54 | 0.819454 | 1.781158 | 0 | 0.025177 | 0.849 | 2348 | tags=52%, list=11%, signal=58% |
| GO_REGULATION_OF_ADAPTIVE_IMMUNE_RESPONSE | 147 | 0.797555 | 1.959463 | 0 | 0.017421 | 0.291 | 1984 | tags=50%, list=9%, signal=54% |
| GO_POSITIVE_REGULATION_OF_ADAPTIVE_IMMUNE_RESPONSE | 93 | 0.803633 | 1.875998 | 0 | 0.018622 | 0.551 | 1984 | tags=52%, list=9%, signal=56% |
| GO_REGULATION_OF_INTERLEUKIN_4_PRODUCTION | 26 | 0.795327 | 1.67547 | 0.00211 | 0.039707 | 0.993 | 2503 | tags=62%, list=11%, signal=69% |
| GO_POSITIVE_REGULATION_OF_MACROPHAGE_MIGRATION | 20 | 0.870037 | 1.727502 | 0 | 0.03135 | 0.958 | 1463 | tags=55%, list=7%, signal=59% |
| GO_REGULATION_OF_T_CELL_MEDIATED_IMMUNITY | 65 | 0.792652 | 1.830283 | 0 | 0.022067 | 0.701 | 2394 | tags=54%, list=11%, signal=60% |
| GO_INTERLEUKIN_6_PRODUCTION | 139 | 0.758804 | 1.849522 | 0 | 0.021059 | 0.645 | 2225 | tags=46%, list=10%, signal=51% |
| GO_NEGATIVE_REGULATION_OF_INNATE_IMMUNE_RESPONSE | 51 | 0.792239 | 1.890717 | 0 | 0.018795 | 0.493 | 2348 | tags=55%, list=11%, signal=61% |
| GO_NEGATIVE_REGULATION_OF_MULTI_ORGANISM_PROCESS | 195 | 0.686704 | 2.031096 | 0 | 0.018258 | 0.153 | 2348 | tags=39%, list=11%, signal=43% |
| GO_POSITIVE_REGULATION_OF_INTERLEUKIN_1_PRODUCTION | 52 | 0.817755 | 1.755902 | 0 | 0.027571 | 0.911 | 1530 | tags=50%, list=7%, signal=54% |
| GO_PHOSPHATIDYLINOSITOL_3_4_BISPHOSPHATE_BINDING | 26 | 0.680059 | 1.867987 | 0 | 0.019177 | 0.576 | 1548 | tags=27%, list=7%, signal=29% |
| GO_MACROPHAGE_MIGRATION | 46 | 0.773627 | 1.703446 | 0 | 0.034512 | 0.975 | 1463 | tags=43%, list=7%, signal=46% |

| | | | | | | | | |
|---|---|---|---|---|---|---|---|---|
| GO_INTERLEUKIN_4_PRODUCTION | 33 | 0.768622 | 1.704076 | 0.0020775 | 0.034622 | 0.975 | 2503 | tags=61%, list=11%, signal=68% |
| GO_POSITIVE_REGULATION_OF_INTERLEUKIN_8_PRODUCTION | 47 | 0.781344 | 1.71628 | 0 | 0.033071 | 0.968 | 1694 | tags=45%, list=8%, signal=48% |
| GO_POSITIVE_REGULATION_OF_LYMPHOCYTE_MIGRATION | 35 | 0.810504 | 1.714185 | 0.0020964 | 0.033483 | 0.969 | 1787 | tags=51%, list=8%, signal=56% |
| GO_CELL_CHEMOTAXIS | 270 | 0.765652 | 1.828884 | 0 | 0.022113 | 0.705 | 2344 | tags=50%, list=11%, signal=55% |
| GO_POSITIVE_REGULATION_OF_INTERLEUKIN_8_SECRETION | 16 | 0.862207 | 1.508767 | 0.0041667 | 0.100068 | 1 | 1694 | tags=69%, list=8%, signal=74% |
| GO_POSITIVE_REGULATION_OF_LEUKOCYTE_CHEMOTAXIS | 82 | 0.805328 | 1.832708 | 0 | 0.022083 | 0.694 | 1693 | tags=46%, list=8%, signal=50% |
| GO_NEGATIVE_REGULATION_OF_RESPONSE_TO_BIOTIC_STIMULUS | 84 | 0.738638 | 1.977912 | 0 | 0.017919 | 0.247 | 2464 | tags=44%, list=11%, signal=49% |
| GO_T_CELL_HOMEOSTASIS | 38 | 0.683877 | 1.866767 | 0 | 0.01903 | 0.58 | 2691 | tags=42%, list=12%, signal=48% |
| GO_REGULATION_OF_CYTOKINE_PRODUCTION_INVOLVED_IN_IMMUNE_RESPONSE | 79 | 0.803399 | 1.807855 | 0 | 0.023377 | 0.776 | 1941 | tags=54%, list=9%, signal=59% |
| GO_NEGATIVE_REGULATION_OF_B_CELL_PROLIFERATION | 16 | 0.849144 | 1.603886 | 0.0020833 | 0.058684 | 1 | 1159 | tags=50%, list=5%, signal=53% |
| GO_MYELOID_CELL_DIFFERENTIATION | 368 | 0.603602 | 2.016002 | 0 | 0.018554 | 0.177 | 3010 | tags=34%, list=14%, signal=39% |
| GO_HOMEOSTASIS_OF_NUMBER_OF_CELLS | 232 | 0.649019 | 2.117239 | 0 | 0.032903 | 0.064 | 2483 | tags=30%, list=11%, signal=34% |
| GO_POSITIVE_REGULATION_OF_INTERLEUKIN_4_PRODUCTION | 21 | 0.823096 | 1.642053 | 0.0042202 | 0.047082 | 1 | 1537 | tags=57%, list=7%, signal=61% |

| | | | | | | | | |
|---|---|---|---|---|---|---|---|---|
| GO_B_CELL_HOMEOSTASIS | 28 | 0.770019 | 1.806259 | 0 | 0.023364 | 0.785 | 2691 | tags=50%, list=12%, signal=57% |
| GO_NEGATIVE_REGULATION_OF_VIRAL_GENOME_REPLICATION | 52 | 0.714082 | 1.708888 | 0.0020208 | 0.033889 | 0.974 | 1941 | tags=44%, list=9%, signal=48% |
| GO_REGULATION_OF_T_CELL_MEDIATED_CYTOTOXICITY | 29 | 0.776513 | 1.657801 | 0.0020008 | 0.043673 | 0.998 | 1949 | tags=52%, list=9%, signal=57% |
| GO_REGULATION_OF_PRODUCTION_OF_MOLECULAR_MEDIATOR_OF_IMMUNE_RESPONSE | 128 | 0.762824 | 1.905876 | 0 | 0.016988 | 0.442 | 1982 | tags=44%, list=9%, signal=48% |
| GO_REGULATION_OF_LEUKOCYTE_CHEMOTAXIS | 104 | 0.779091 | 1.783816 | 0 | 0.025127 | 0.846 | 1693 | tags=44%, list=8%, signal=48% |
| GO_B_CELL_MEDIATED_IMMUNITY | 111 | 0.791582 | 1.885557 | 0 | 0.018752 | 0.51 | 2002 | tags=45%, list=9%, signal=49% |
| GO_CELLULAR_RESPONSE_TO_BIOTIC_STIMULUS | 218 | 0.747262 | 1.909629 | 0 | 0.016635 | 0.424 | 1761 | tags=40%, list=8%, signal=43% |
| GO_SPECIFIC_GRANULE_MEMBRANE | 87 | 0.774442 | 1.887699 | 0 | 0.018985 | 0.504 | 3126 | tags=59%, list=14%, signal=68% |
| GO_RESPONSE_TO_CHEMOKINE | 88 | 0.842962 | 1.739819 | 0 | 0.029374 | 0.938 | 1863 | tags=61%, list=8%, signal=67% |
| GO_B_CELL_ACTIVATION | 238 | 0.742005 | 1.98591 | 0 | 0.017381 | 0.232 | 2952 | tags=47%, list=13%, signal=53% |
| GO_POSITIVE_REGULATION_OF_INTERLEUKIN_2_PRODUCTION | 30 | 0.836231 | 1.685204 | 0 | 0.037771 | 0.99 | 1564 | tags=57%, list=7%, signal=61% |
| GO_RECEPTOR_SIGNALING_PATHWAY_VIA_STAT | 152 | 0.673905 | 1.971729 | 0 | 0.018415 | 0.261 | 2510 | tags=37%, list=11%, signal=41% |
| GO_POSITIVE_REGULATION_OF_RESPONSE_TO_CYTOKINE_STIMULUS | 51 | 0.768307 | 1.875774 | 0 | 0.018607 | 0.551 | 1941 | tags=47%, list=9%, signal=51% |
| GO_POSITIVE_REGULATION_OF_CALCIUM_MEDIATED_SIGNALING | 39 | 0.649638 | 1.764525 | 0 | 0.026791 | 0.891 | 2492 | tags=36%, list=11%, signal=40% |
| GO_REGULATION_OF_NIK_NF_KAPPAB_SIGNALING | 99 | 0.695489 | 1.919913 | 0 | 0.016206 | 0.403 | 2891 | tags=42%, list=13%, signal=49% |
| GO_CYTOKINE_METABOLIC_PROCESS | 111 | 0.76819 | 1.883707 | 0 | 0.018628 | 0.518 | 1612 | tags=41%, list=7%, signal=44% |

| | | | | | | | | |
|---|---|---|---|---|---|---|---|---|
| GO_POSITIVE_REGULATION_OF_ALPHA_BETA_T_CELL_PROLIFERATION | 18 | 0.849835 | 1.644999 | 0 | 0.046317 | 1 | 1693 | tags=72%, list=8%, signal=78% |
| GO_POSITIVE_REGULATION_OF_NITRIC_OXIDE_SYNTHASE_BIOSYNTHETIC_PROCESS | 15 | 0.922282 | 1.609215 | 0 | 0.057138 | 1 | 999 | tags=67%, list=5%, signal=70% |
| GO_REGULATION_OF_B_CELL_PROLIFERATION | 60 | 0.810683 | 1.825183 | 0 | 0.022278 | 0.716 | 1159 | tags=40%, list=5%, signal=42% |
| GO_NEGATIVE_REGULATION_OF_VIRAL_LIFE_CYCLE | 74 | 0.700029 | 1.829505 | 0 | 0.022126 | 0.704 | 2038 | tags=39%, list=9%, signal=43% |
| GO_POSITIVE_REGULATION_OF_TYPE_I_INTERFERON_PRODUCTION | 74 | 0.690953 | 2.005027 | 0 | 0.01867 | 0.198 | 1744 | tags=26%, list=8%, signal=28% |
| GO_T_CELL_APOPTOTIC_PROCESS | 48 | 0.740908 | 1.843197 | 0 | 0.021438 | 0.662 | 2712 | tags=48%, list=12%, signal=55% |
| GO_POSITIVE_REGULATION_OF_MYELOID_CELL_DIFFERENTIATION | 86 | 0.641631 | 1.880628 | 0 | 0.018723 | 0.527 | 2986 | tags=42%, list=14%, signal=48% |
| GO_MYELOID_LEUKOCYTE_MIGRATION | 190 | 0.791666 | 1.804069 | 0 | 0.023362 | 0.793 | 1904 | tags=52%, list=9%, signal=56% |
| GO_NEGATIVE_REGULATION_OF_CELL_ACTIVATION | 182 | 0.73634 | 1.879466 | 0 | 0.018796 | 0.534 | 2079 | tags=44%, list=9%, signal=48% |
| GO_PROTEIN_AUTOPHOSPHORYLATION | 223 | 0.507402 | 1.92345 | 0 | 0.016739 | 0.395 | 2900 | tags=24%, list=13%, signal=27% |
| GO_RESPONSE_TO_MOLECULE_OF_BACTERIAL_ORIGIN | 322 | 0.717051 | 1.886812 | 0 | 0.018869 | 0.508 | 2224 | tags=41%, list=10%, signal=45% |
| GO_T_CELL_RECEPTOR_COMPLEX | 21 | 0.839008 | 1.510183 | 0.008565 | 0.099402 | 1 | 1610 | tags=57%, list=7%, signal=62% |
| GO_TUMOR_NECROSIS_FACTOR_RECEPTOR_SUPERFAMILY_BINDING | 41 | 0.714936 | 1.85461 | 0.004264 | 0.02035 | 0.62 | 2331 | tags=41%, list=11%, signal=46% |
| GO_REGULATION_OF_MYOTUBE_DIFFERENTIATION | 49 | 0.593265 | 1.673167 | 0.005882 | 0.040171 | 0.993 | 2121 | tags=27%, list=10%, signal=29% |
| GO_POSITIVE_REGULATION_OF_PRODUCTION_OF_MOLECULAR_MEDIATOR_OF_IMMUNE_RESPONSE | 85 | 0.757766 | 1.878069 | 0 | 0.018811 | 0.542 | 1982 | tags=44%, list=9%, signal=48% |

| | | | | | | | | |
|---|---|---|---|---|---|---|---|---|
| GO_REGULATORY_T_CELL_DIFFERENTIATION | 28 | 0.856444 | 1.693619 | 0 | 0.03619 | 0.985 | 1641 | tags=61%, list=7%, signal=66% |
| GO_NEGATIVE_REGULATION_OF_T_CELL_MEDIATED_IMMUNITY | 17 | 0.816996 | 1.732606 | 0.002088 | 0.030734 | 0.95 | 2348 | tags=47%, list=11%, signal=53% |
| GO_POSITIVE_REGULATION_OF_NIK_NF_KAPPAB_SIGNALING | 75 | 0.717609 | 1.899907 | 0 | 0.017542 | 0.457 | 2891 | tags=45%, list=13%, signal=52% |
| GO_MYELOID_DENDRITIC_CELL_ACTIVATION | 27 | 0.83214 | 1.811952 | 0 | 0.023025 | 0.765 | 2847 | tags=70%, list=13%, signal=81% |
| GO_REGULATION_OF_T_CELL_CYTOKINE_PRODUCTION | 30 | 0.839294 | 1.737343 | 0.002132 | 0.029693 | 0.944 | 1332 | tags=53%, list=6%, signal=57% |
| GO_POSITIVE_REGULATION_OF_PHAGOCYTOSIS | 63 | 0.772464 | 1.754077 | 0 | 0.027728 | 0.915 | 1794 | tags=46%, list=8%, signal=50% |
| GO_INTERLEUKIN_1_SECRETION | 53 | 0.755497 | 1.71733 | 0 | 0.033003 | 0.968 | 1713 | tags=51%, list=8%, signal=55% |
| GO_NEGATIVE_REGULATION_OF_ALPHA_BETA_T_CELL_ACTIVATION | 31 | 0.76904 | 1.838379 | 0 | 0.021638 | 0.679 | 2493 | tags=65%, list=11%, signal=73% |
| GO_POSITIVE_REGULATION_OF_ANTIGEN_RECEPTOR_MEDIATED_SIGNALING_PATHWAY | 21 | 0.832583 | 1.609012 | 0 | 0.05717 | 1 | 2142 | tags=67%, list=10%, signal=74% |
| GO_LYMPHOCYTE_CHEMOTAXIS | 52 | 0.844449 | 1.732362 | 0 | 0.030704 | 0.95 | 2216 | tags=67%, list=10%, signal=75% |
| GO_GLIAL_CELL_ACTIVATION | 49 | 0.875162 | 1.69767 | 0 | 0.035459 | 0.981 | 1463 | tags=59%, list=7%, signal=63% |
| GO_NEGATIVE_REGULATION_OF_INTERLEUKIN_12_PRODUCTION | 16 | 0.88127 | 1.58078 | 0 | 0.066352 | 1 | 1512 | tags=75%, list=7%, signal=80% |
| GO_REGULATION_OF_MONONUCLEAR_CELL_MIGRATION | 43 | 0.824517 | 1.790381 | 0 | 0.024584 | 0.836 | 1159 | tags=44%, list=5%, signal=47% |
| GO_POSITIVE_REGULATION_OF_SMALL_GTPASE_MEDIATED_SIGNAL_TRANSDUCTION | 70 | 0.614499 | 1.750618 | 0 | 0.028409 | 0.926 | 2342 | tags=29%, list=11%, signal=32% |
| GO_POSITIVE_REGULATION_OF_INTERLEUKIN_6_PRODUCTION | 89 | 0.770278 | 1.819478 | 0 | 0.022614 | 0.729 | 2351 | tags=47%, list=11%, signal=53% |
| GO_ALPHA_BETA_T_CELL_PROLIFERATION | 29 | 0.832186 | 1.705138 | 0 | 0.03433 | 0.974 | 1693 | tags=66%, list=8%, signal=71% |

| | | | | | | | | |
|---|---|---|---|---|---|---|---|---|
| GO_NEGATIVE_REGULATION_OF_LYMPHOCYTE_ACTIVATION | 136 | 0.756201 | 1.920528 | 0 | 0.016186 | 0.4 | 1699 | tags=43%, list=8%, signal=46% |
| GO_THYMIC_T_CELL_SELECTION | 21 | 0.817989 | 1.654786 | 0 | 0.044462 | 0.999 | 1699 | tags=48%, list=8%, signal=52% |
| GO_INTERLEUKIN_2_BIOSYNTHETIC_PROCESS | 22 | 0.837284 | 1.631797 | 0.00641 | 0.049683 | 1 | 1541 | tags=55%, list=7%, signal=59% |
| GO_PHOSPHATIDYLINOSITOL_3_4_5_TRISPHOSPHATE_BINDING | 36 | 0.580683 | 1.793584 | 0.002012 | 0.02407 | 0.827 | 1926 | tags=22%, list=9%, signal=24% |
| GO_PATTERN_RECOGNITION_RECEPTOR_ACTIVITY | 21 | 0.88374 | 1.558488 | 0 | 0.075637 | 1 | 878 | tags=62%, list=4%, signal=64% |
| GO_NEGATIVE_REGULATION_OF_INTERFERON_GAMMA_PRODUCTION | 30 | 0.813437 | 1.808866 | 0 | 0.023325 | 0.771 | 1530 | tags=47%, list=7%, signal=50% |
| GO_NEGATIVE_REGULATION_OF_VIRAL_PROCESS | 89 | 0.678502 | 1.877101 | 0 | 0.018856 | 0.547 | 2284 | tags=37%, list=10%, signal=41% |
| GO_SH2_DOMAIN_BINDING | 35 | 0.735767 | 1.859379 | 0 | 0.019706 | 0.606 | 2797 | tags=43%, list=13%, signal=49% |
| GO_IMMUNOGLOBULIN_PRODUCTION | 101 | 0.739307 | 1.944789 | 0 | 0.016399 | 0.329 | 2493 | tags=41%, list=11%, signal=46% |
| GO_GRANULOCYTE_MIGRATION | 128 | 0.818331 | 1.786576 | 0 | 0.024662 | 0.84 | 1904 | tags=57%, list=9%, signal=62% |
| GO_REGULATION_OF_PHAGOCYTOSIS | 86 | 0.760257 | 1.818115 | 0 | 0.022635 | 0.737 | 1794 | tags=42%, list=8%, signal=45% |
| GO_LEUKOCYTE_APOPTOTIC_PROCESS | 103 | 0.777835 | 1.869699 | 0 | 0.019203 | 0.569 | 1787 | tags=44%, list=8%, signal=47% |
| GO_POSITIVE_REGULATION_OF_CHEMOTAXIS | 130 | 0.755838 | 1.817303 | 0 | 0.022746 | 0.741 | 1863 | tags=42%, list=8%, signal=45% |
| GO_POSITIVE_REGULATION_OF_CALCIUM_ION_TRANSPORT_INTO_CYTOSOL | 51 | 0.661767 | 1.699829 | 0 | 0.035225 | 0.98 | 2194 | tags=35%, list=10%, signal=39% |
| GO_NEGATIVE_REGULATION_OF_LEUKOCYTE_CELL_CELL_ADHESION | 112 | 0.728414 | 1.915642 | 0 | 0.016255 | 0.415 | 1699 | tags=40%, list=8%, signal=43% |
| GO_DEFENSE_RESPONSE_TO_BACTERIUM | 209 | 0.672049 | 1.852704 | 0 | 0.020583 | 0.629 | 2776 | tags=39%, list=13%, signal=44% |

| | | | | | | | | |
|---|---|---|---|---|---|---|---|---|
| GO_CYTOKINE_PRODUCTION_INVOLVED_IN_INFLAMMATORY_RESPONSE | 40 | 0.749268 | 1.78218 | 0 | 0.025278 | 0.849 | 2224 | tags=40%, list=10%, signal=44% |
| GO_POSITIVE_REGULATION_OF_T_CELL_MEDIATED_IMMUNITY | 44 | 0.787339 | 1.767538 | 0 | 0.026711 | 0.883 | 2234 | tags=57%, list=10%, signal=63% |
| GO_REGULATION_OF_VIRAL_LIFE_CYCLE | 132 | 0.611931 | 1.883644 | 0 | 0.018533 | 0.518 | 2642 | tags=33%, list=12%, signal=37% |
| GO_LYMPHOCYTE_APOPTOTIC_PROCESS | 71 | 0.728088 | 1.890886 | 0 | 0.018857 | 0.491 | 2712 | tags=44%, list=12%, signal=50% |
| GO_PURINERGIC_RECEPTOR_ACTIVITY | 23 | 0.874581 | 1.66846 | 0 | 0.041179 | 0.995 | 895 | tags=52%, list=4%, signal=54% |
| GO_POSITIVE_REGULATION_OF_INTERLEUKIN_10_PRODUCTION | 32 | 0.851097 | 1.708075 | 0 | 0.034048 | 0.974 | 1693 | tags=59%, list=8%, signal=64% |
| GO_ACUTE_INFLAMMATORY_RESPONSE_TO_ANTIGENIC_STIMULUS | 19 | 0.856623 | 1.648338 | 0 | 0.045649 | 1 | 747 | tags=42%, list=3%, signal=44% |
| GO_POSITIVE_REGULATION_OF_RELEASE_OF_SEQUESTERED_CALCIUM_ION_INTO_CYTOSOL | 37 | 0.692906 | 1.678587 | 0.0020041 | 0.038954 | 0.992 | 2194 | tags=38%, list=10%, signal=42% |
| GO_POSITIVE_REGULATION_OF_MONOCYTE_CHEMOTAXIS | 18 | 0.873965 | 1.64759 | 0.0020012 | 0.04584 | 1 | 997 | tags=56%, list=5%, signal=58% |
| GO_NEUTROPHIL_MIGRATION | 107 | 0.838374 | 1.746261 | 0 | 0.028851 | 0.93 | 1863 | tags=62%, list=8%, signal=67% |
| GO_POSITIVE_REGULATION_OF_JNK_CASCADE | 131 | 0.570891 | 1.758142 | 0 | 0.027238 | 0.908 | 2891 | tags=31%, list=13%, signal=35% |
| GO_REGULATION_OF_MONOCYTE_CHEMOTAXIS | 24 | 0.847239 | 1.614016 | 0.0020045 | 0.055591 | 1 | 1159 | tags=54%, list=5%, signal=57% |
| GO_TYROSINE_PHOSPHORYLATION_OF_STAT_PROTEIN | 81 | 0.717407 | 1.977988 | 0 | 0.018146 | 0.247 | 2510 | tags=44%, list=11%, signal=50% |
| GO_MONONUCLEAR_CELL_MIGRATION | 77 | 0.831299 | 1.761796 | 0 | 0.027062 | 0.9 | 1863 | tags=57%, list=8%, signal=62% |
| GO_NEGATIVE_REGULATION_OF_IMMUNE_RESPONSE | 140 | 0.748506 | 1.95296 | 0 | 0.016251 | 0.303 | 2351 | tags=50%, list=11%, signal=56% |

| | | | | | | | | |
|---|---|---|---|---|---|---|---|---|
| GO_NEGATIVE_REGULATION_OF_ADAPTIVE_IMMUNE_RESPONSE | 43 | 0.787939 | 1.88521 | 0 | 0.01862 | 0.513 | 1878 | tags=47%, list=9%, signal=51% |
| GO_NEUROINFLAMMATORY_RESPONSE | 61 | 0.881156 | 1.760387 | 0 | 0.027136 | 0.901 | 1463 | tags=62%, list=7%, signal=67% |
| GO_HOMEOSTASIS_OF_NUMBER_OF_CELLS_WITHIN_A_TISSUE | 29 | 0.660955 | 1.778674 | 0.002062 | 0.025467 | 0.857 | 2121 | tags=31%, list=10%, signal=34% |
| GO_REGULATION_OF_CALCIUM_ION_TRANSPORT | 232 | 0.559044 | 1.691116 | 0.002008 | 0.036812 | 0.987 | 3000 | tags=32%, list=14%, signal=37% |
| GO_NEGATIVE_REGULATION_OF_LEUKOCYTE_MEDIATED_CYTOTOXICITY | 15 | 0.848612 | 1.628079 | 0.002088 | 0.050871 | 1 | 2348 | tags=73%, list=11%, signal=82% |
| GO_DEFENSE_RESPONSE_TO_GRAM_POSITIVE_BACTERIUM | 69 | 0.695502 | 1.813331 | 0 | 0.022993 | 0.759 | 2721 | tags=41%, list=12%, signal=46% |
| GO_MONOCYTE_CHEMOTAXIS | 55 | 0.852848 | 1.678406 | 0 | 0.038963 | 0.992 | 1863 | tags=65%, list=8%, signal=71% |
| GO_NEGATIVE_REGULATION_OF_CELL_CELL_ADHESION | 163 | 0.662148 | 1.918805 | 0 | 0.01625 | 0.407 | 1878 | tags=34%, list=9%, signal=37% |
| GO_NEGATIVE_REGULATION_OF_INTERLEUKIN_2_PRODUCTION | 23 | 0.730447 | 1.551977 | 0.031621 | 0.079015 | 1 | 984 | tags=39%, list=4%, signal=41% |
| GO_POSITIVE_REGULATION_OF_MONONUCLEAR_CELL_MIGRATION | 23 | 0.855244 | 1.725974 | 0 | 0.031673 | 0.96 | 997 | tags=48%, list=5%, signal=50% |
| GO_POSITIVE_REGULATION_OF_RECEPTOR_SIGNALING_PATHWAY_VIA_STAT | 88 | 0.709538 | 1.854866 | 0 | 0.020385 | 0.619 | 3047 | tags=51%, list=14%, signal=59% |
| GO_REGULATION_OF_CHEMOTAXIS | 199 | 0.695545 | 1.827111 | 0 | 0.02222 | 0.711 | 1761 | tags=33%, list=8%, signal=36% |
| GO_NEGATIVE_REGULATION_OF_LEUKOCYTE_MEDIATED_IMMUNITY | 46 | 0.831702 | 1.8617 | 0 | 0.019669 | 0.599 | 2348 | tags=59%, list=11%, signal=66% |

| | | | | | | | | |
|---|---|---|---|---|---|---|---|---|
| GO_POSITIVE_REGULATION_OF_CALCIUM_ION_TRANSPORT | 115 | 0.571194 | 1.630533 | 0.001988 | 0.050135 | 1 | 2897 | tags=34%, list=13%, signal=39% |
| GO_ACTIVATION_OF_PHOSPHOLIPASE_C_ACTIVITY | 30 | 0.733026 | 1.605951 | 0.00198 | 0.058043 | 1 | 2869 | tags=43%, list=13%, signal=50% |
| GO_NEGATIVE_REGULATION_OF_HEMOPOIESIS | 130 | 0.620958 | 1.861355 | 0 | 0.01972 | 0.6 | 2493 | tags=32%, list=11%, signal=36% |
| GO_NEGATIVE_REGULATION_OF_LEUKOCYTE_DIFFERENTIATION | 94 | 0.668702 | 1.86286 | 0 | 0.01955 | 0.596 | 2493 | tags=39%, list=11%, signal=44% |
| GO_VITAMIN_D_METABOLIC_PROCESS | 22 | 0.667356 | 1.517227 | 0.015487 | 0.096468 | 1 | 2024 | tags=36%, list=9%, signal=40% |
| **High-expression *SLAMF8* vs. low-expression *SLAMF8* colorectal cancer tissues (GSE14333)** | | | | | | | | |
| NAME | SIZE | ES | NES | NOM.p.val | FDR.q.val | FWER.p.val | RANK.AT.MAX | LEADING.EDGE |
| GO_DOUBLE_STRANDED_RNA_BINDING | 71 | 0.383642 | 1.753444 | 0.02686 | 0.068359 | 0.986 | 3337 | tags=15%, list=14%, signal=18% |
| GO_T_CELL_RECEPTOR_SIGNALING_PATHWAY | 177 | 0.49015 | 1.916435 | 0.016064 | 0.069494 | 0.761 | 4220 | tags=29%, list=18%, signal=36% |
| GO_ANTIGEN_RECEPTOR_MEDIATED_SIGNALING_PATHWAY | 226 | 0.528749 | 1.845068 | 0.020492 | 0.058657 | 0.904 | 4232 | tags=34%, list=18%, signal=41% |
| GO_NEGATIVE_REGULATION_OF_TYPE_I_INTERFERON_PRODUCTION | 41 | 0.597512 | 1.883927 | 0.002083 | 0.06072 | 0.842 | 3812 | tags=37%, list=16%, signal=44% |

| | | | | | | | | |
|---|---|---|---|---|---|---|---|---|
| GO_IMMUNE_RESPONSE_REGULATING_CELL_SURFACE_RECEPTOR_SIGNALING_PATHWAY | 367 | 0.51636 | 1.884659 | 0.0010331 | 0.061126 | 0.84 | 4747 | tags=35%, list=20%, signal=43% |
| GO_FC_RECEPTOR_SIGNALING_PATHWAY | 176 | 0.444502 | 1.981939 | 0.0004132 | 0.101125 | 0.573 | 4979 | tags=26%, list=21%, signal=33% |
| GO_NEGATIVE_REGULATION_OF_ANTIGEN_RECEPTOR_MEDIATED_SIGNALING_PATHWAY | 28 | 0.647988 | 1.757378 | 0.0004107 | 0.068666 | 0.985 | 4828 | tags=57%, list=21%, signal=72% |
| GO_REGULATION_OF_DEFENSE_RESPONSE_TO_VIRUS | 64 | 0.53041 | 1.916082 | 0.0004264 | 0.069166 | 0.761 | 3285 | tags=33%, list=14%, signal=38% |
| GO_ALPHA_BETA_T_CELL_DIFFERENTIATION | 95 | 0.489395 | 1.609541 | 0.0046316 | 0.107697 | 1 | 5019 | tags=42%, list=21%, signal=53% |
| GO_REGULATION_OF_T_CELL_RECEPTOR_SIGNALING_PATHWAY | 38 | 0.595375 | 1.775356 | 0.001626 | 0.068181 | 0.978 | 4106 | tags=45%, list=17%, signal=54% |
| GO_T_HELPER_1_TYPE_IMMUNE_RESPONSE | 41 | 0.581493 | 1.653433 | 0.0015021 | 0.0926 | 1 | 2727 | tags=41%, list=12%, signal=47% |
| GO_NEGATIVE_REGULATION_OF_T_CELL_RECEPTOR_SIGNALING_PATHWAY | 20 | 0.556472 | 1.60799 | 0.0040241 | 0.107877 | 1 | 4828 | tags=50%, list=21%, signal=63% |
| GO_T_CELL_ACTIVATION_INVOLVED_IN_IMMUNE_RESPONSE | 100 | 0.527208 | 1.719737 | 0.0019108 | 0.074375 | 0.997 | 1290 | tags=25%, list=5%, signal=26% |

| | | | | | | | | |
|---|---|---|---|---|---|---|---|---|
| GO_RESPONSE_TO_TYPE_I_INTERFERON | 88 | 0.528139 | 1.775453 | 0.0040685 | 0.068324 | 0.978 | 3892 | tags=33%, list=17%, signal=39% |
| GO_POSITIVE_REGULATION_OF_ALPHA_BETA_T_CELL_ACTIVATION | 58 | 0.571468 | 1.746833 | 0.0146144 | 0.068831 | 0.991 | 2983 | tags=36%, list=13%, signal=41% |
| GO_ALPHA_BETA_T_CELL_ACTIVATION | 125 | 0.567392 | 1.830541 | 0.0061988 | 0.059295 | 0.92 | 3220 | tags=37%, list=14%, signal=42% |
| GO_POSITIVE_REGULATION_OF_LEUKOCYTE_DIFFERENTIATION | 143 | 0.567369 | 1.881682 | 0 | 0.060426 | 0.851 | 4537 | tags=43%, list=19%, signal=53% |
| GO_NATURAL_KILLER_CELL_ACTIVATION | 77 | 0.559543 | 1.728608 | 0.0086621 | 0.072389 | 0.993 | 4334 | tags=40%, list=18%, signal=49% |
| GO_POSITIVE_REGULATION_OF_ALPHA_BETA_T_CELL_DIFFERENTIATION | 45 | 0.516947 | 1.628626 | 0.0267499 | 0.100979 | 1 | 2801 | tags=31%, list=12%, signal=35% |
| GO_NON_MEMBRANE_SPANNING_PROTEIN_TYROSINE_KINASE_ACTIVITY | 45 | 0.572785 | 1.88713 | 0.00404 | 0.062042 | 0.835 | 912 | tags=20%, list=4%, signal=21% |
| GO_CD4_POSITIVE_ALPHA_BETA_T_CELL_DIFFERENTIATION | 69 | 0.513923 | 1.639782 | 0.0306612 | 0.096635 | 1 | 5019 | tags=45%, list=21%, signal=57% |
| GO_LYMPHOCYTE_ACTIVATION_INVOLVED_IN_IMMUNE_RESPONSE | 168 | 0.513866 | 1.820689 | 0.01222 | 0.060451 | 0.93 | 4723 | tags=40%, list=20%, signal=50% |
| GO_POSITIVE_REGULATION_OF_CELL_ACTIVATION | 305 | 0.615113 | 2.031769 | 0 | 0.15916 | 0.394 | 4362 | tags=46%, list=19%, signal=56% |

| | | | | | | | | |
|---|---|---|---|---|---|---|---|---|
| GO_T_CELL_ACTIVATION | 432 | 0.555944 | 1.982338 | 0.0020045 | 0.105793 | 0.572 | 4362 | tags=40%, list=19%, signal=48% |
| GO_REGULATION_OF_ANTIGEN_RECEPTOR_MEDIATED_SIGNALING_PATHWAY | 61 | 0.594571 | 1.78903 | 0.0101006 | 0.065534 | 0.967 | 4405 | tags=46%, list=19%, signal=56% |
| GO_POSITIVE_REGULATION_OF_LYMPHOCYTE_DIFFERENTIATION | 91 | 0.60838 | 1.892767 | 0.0082822 | 0.06136 | 0.823 | 4362 | tags=47%, list=19%, signal=58% |
| GO_LYMPHOCYTE_DIFFERENTIATION | 336 | 0.512893 | 1.849743 | 0.0104438 | 0.058444 | 0.898 | 4723 | tags=39%, list=20%, signal=48% |
| GO_DEFENSE_RESPONSE_TO_VIRUS | 218 | 0.50743 | 1.885747 | 0.0085547 | 0.061273 | 0.836 | 3673 | tags=32%, list=16%, signal=37% |
| GO_REGULATION_OF_LEUKOCYTE_DEGRANULATION | 42 | 0.686726 | 2.080969 | 0 | 0.2714 | 0.283 | 1621 | tags=31%, list=7%, signal=33% |
| GO_POSITIVE_REGULATION_OF_INTERFERON_ALPHA_PRODUCTION | 22 | 0.582119 | 1.645835 | 0.0306612 | 0.096256 | 1 | 3673 | tags=41%, list=16%, signal=48% |
| GO_RESPONSE_TO_VIRUS | 295 | 0.508219 | 1.947244 | 0.0064244 | 0.08376 | 0.671 | 3742 | tags=31%, list=16%, signal=36% |
| GO_REGULATION_OF_ALPHA_BETA_T_CELL_DIFFERENTIATION | 60 | 0.472705 | 1.563552 | 0.04888 | 0.125248 | 1 | 4909 | tags=40%, list=21%, signal=50% |
| GO_REGULATION_OF_DEFENSE_RESPONSE_TO_VIRUS_BY_VIRUS | 30 | 0.628481 | 1.906292 | 0.0060036 | 0.066249 | 0.783 | 3839 | tags=33%, list=16%, signal=40% |

| | | | | | | | | |
|---|---|---|---|---|---|---|---|---|
| GO_CD4_POSITIVE_ALPHA_BETA_T_CELL_ACTIVATION | 83 | 0.530324 | 1.735621 | 0.0012397 | 0.071594 | 0.992 | 5019 | tags=46%, list=21%, signal=58% |
| GO_REGULATION_OF_NATURAL_KILLER_CELL_ACTIVATION | 32 | 0.662459 | 1.771591 | 0.0041 49 | 0.068617 | 0.98 | 5862 | tags=63%, list=25%, signal=83% |
| GO_POSITIVE_REGULATION_OF_LEUKOCYTE_CELL_CELL_ADHESION | 203 | 0.619851 | 1.979063 | 0.0020779 | 0.101223 | 0.585 | 4423 | tags=48%, list=19%, signal=58% |
| GO_INTERFERON_GAMMA_PRODUCTION | 100 | 0.611245 | 1.802486 | 0.00823 | 0.063859 | 0.947 | 3092 | tags=44%, list=13%, signal=50% |
| GO_ACTIVATED_T_CELL_PROLIFERATION | 39 | 0.503599 | 1.524933 | 0.0291 67 | 0.140825 | 1 | 5911 | tags=51%, list=25%, signal=68% |
| GO_CELLULAR_RESPONSE_TO_INTERFERON_BETA | 18 | 0.630759 | 1.633326 | 0.0390 14 | 0.099806 | 1 | 3189 | tags=44%, list=14%, signal=51% |
| GO_ACTIVATION_OF_INNATE_IMMUNE_RESPONSE | 290 | 0.493433 | 2.009849 | 0.0020 96 | 0.124322 | 0.458 | 3892 | tags=26%, list=17%, signal=31% |
| GO_MYD88_INDEPENDENT_TOLL_LIKE_RECEPTOR_SIGNALING_PATHWAY | 32 | 0.522131 | 1.752095 | 0.0185 19 | 0.068576 | 0.986 | 3153 | tags=22%, list=13%, signal=25% |
| GO_INTERFERON_ALPHA_PRODUCTION | 29 | 0.626119 | 1.724703 | 0.0125 26 | 0.073341 | 0.994 | 3673 | tags=45%, list=16%, signal=53% |

| | | | | | | | | |
|---|---|---|---|---|---|---|---|---|
| GO_T_CELL_DIFFERENTIATION | 226 | 0.528922 | 1.823678 | 0.0010571 | 0.060361 | 0.926 | 4558 | tags=42%, list=19%, signal=51% |
| GO_POSITIVE_REGULATION_OF_INTERFERON_BETA_PRODUCTION | 30 | 0.593329 | 1.857831 | 0.0040073 | 0.057779 | 0.889 | 4881 | tags=40%, list=21%, signal=50% |
| GO_T_CELL_PROLIFERATION | 172 | 0.578607 | 1.901161 | 0.0020062 | 0.064577 | 0.797 | 4294 | tags=45%, list=18%, signal=54% |
| GO_TUMOR_NECROSIS_FACTOR_BIOSYNTHETIC_PROCESS | 28 | 0.700841 | 1.736839 | 0.0083368 | 0.071074 | 0.992 | 3953 | tags=57%, list=17%, signal=69% |
| GO_RESPONSE_TO_INTERFERON_ALPHA | 20 | 0.642976 | 1.640341 | 0.02935 | 0.096542 | 1 | 2737 | tags=35%, list=12%, signal=40% |
| GO_REGULATION_OF_ALPHA_BETA_T_CELL_ACTIVATION | 83 | 0.571025 | 1.802738 | 0.0012474 | 0.063928 | 0.947 | 4909 | tags=48%, list=21%, signal=61% |
| GO_POSITIVE_REGULATION_OF_RESPONSE_TO_BIOTIC_STIMULUS | 361 | 0.509011 | 1.991695 | 0.0021005 | 0.122188 | 0.541 | 3923 | tags=28%, list=17%, signal=33% |
| GO_T_CELL_DIFFERENTIATION_INVOLVED_IN_IMMUNE_RESPONSE | 65 | 0.526346 | 1.64926 | 0.0032787 | 0.094461 | 1 | 1290 | tags=26%, list=5%, signal=28% |
| GO_LEUKOCYTE_DIFFERENTIATION | 492 | 0.48498 | 1.870467 | 0.0041175 | 0.059387 | 0.875 | 4362 | tags=34%, list=19%, signal=41% |

| | | | | | | | | |
|---|---|---|---|---|---|---|---|---|
| GO_REGULATION_OF_T_HELPER_1_TYPE_IMMUNE_RESPONSE | 26 | 0.619103 | 1.607067 | 0.0167 01 | 0.108387 | 1 | 2727 | tags=50%, list=12%, signal=56% |
| GO_REGULATION_OF_OSTEOCLAST_DIFFERENTIATION | 61 | 0.528031 | 1.699452 | 0.0080 65 | 0.078796 | 0.999 | 4511 | tags=39%, list=19%, signal=49% |
| GO_POSITIVE_REGULATION_OF_HEMOPOIESIS | 178 | 0.527321 | 1.893671 | 0 | 0.061329 | 0.821 | 4537 | tags=38%, list=19%, signal=47% |
| GO_POSITIVE_REGULATION_OF_T_HELPER_1_TYPE_IMMUNE_RESPONSE | 19 | 0.675712 | 1.632503 | 0.0180 36 | 0.099878 | 1 | 2727 | tags=58%, list=12%, signal=65% |
| GO_REGULATION_OF_MYELOID_LEUKOCYTE_DIFFERENTIATION | 110 | 0.480341 | 1.723573 | 0.0061 35 | 0.073413 | 0.994 | 4537 | tags=35%, list=19%, signal=44% |
| GO_ADAPTIVE_IMMUNE_RESPONSE | 375 | 0.603362 | 1.909092 | 0.0081 14 | 0.069302 | 0.776 | 4747 | tags=50%, list=20%, signal=62% |
| GO_REGULATION_OF_DEFENSE_RESPONSE_TO_VIRUS_BY_HOST | 32 | 0.590207 | 1.863418 | 0 | 0.057001 | 0.884 | 3285 | tags=44%, list=14%, signal=51% |
| GO_PHAGOCYTOSIS | 244 | 0.579946 | 2.000153 | 0 | 0.13285 | 0.494 | 3483 | tags=36%, list=15%, signal=41% |
| GO_REGULATION_OF_LEUKOCYTE_DIFFERENTIATION | 259 | 0.522377 | 1.908355 | 0.0021 14 | 0.066743 | 0.779 | 4537 | tags=39%, list=19%, signal=48% |
| GO_TUMOR_NECROSIS_FACTOR_SUPERFAMILY_CYTOKINE_PRODUCTION | 139 | 0.654879 | 1.941725 | 0 | 0.076496 | 0.685 | 3953 | tags=49%, list=17%, signal=58% |
| GO_REGULATION_OF_RESPONSE_TO_BIOTIC_STIMULUS | 475 | 0.519058 | 2.053773 | 0.0020 7 | 0.211872 | 0.341 | 4340 | tags=31%, list=18%, signal=38% |

| | | | | | | | | |
|---|---|---|---|---|---|---|---|---|
| GO_ANTIGEN_PROCESSING_AND_PRESENTATION | 198 | 0.502073 | 2.212674 | 0.002058 | 0.20959 | 0.087 | 4064 | tags=23%, list=17%, signal=27% |
| GO_ADAPTIVE_IMMUNE_RESPONSE_BASED_ON_SOMATIC_RECOMBINATION_OF_IMMUNE_RECEPTORS_BUILT_FROM_IMMUNOGLOBULIN_SUPERFAMILY_DOMAINS | 240 | 0.580635 | 1.87715 | 0.004073 | 0.059839 | 0.865 | 4932 | tags=49%, list=21%, signal=62% |
| GO_REGULATION_OF_T_CELL_ACTIVATION | 291 | 0.578692 | 1.977132 | 0.002053 | 0.097981 | 0.593 | 4362 | tags=43%, list=19%, signal=53% |
| GO_POSITIVE_REGULATION_OF_CYTOKINE_PRODUCTION | 421 | 0.574911 | 2.009983 | 0 | 0.129422 | 0.458 | 3798 | tags=37%, list=16%, signal=43% |
| GO_REGULATION_OF_HEMOPOIESIS | 421 | 0.454115 | 1.950592 | 0.002101 | 0.084778 | 0.663 | 4537 | tags=30%, list=19%, signal=37% |
| GO_POSITIVE_REGULATION_OF_CELL_CELL_ADHESION | 239 | 0.589185 | 1.99143 | 0 | 0.118755 | 0.542 | 4423 | tags=44%, list=19%, signal=54% |
| GO_POSITIVE_REGULATION_OF_IMMUNE_EFFECTOR_PROCESS | 189 | 0.596565 | 2.017376 | 0 | 0.131928 | 0.437 | 2689 | tags=33%, list=11%, signal=37% |
| GO_POSITIVE_REGULATION_OF_INTERFERON_GAMMA_PRODUCTION | 57 | 0.629001 | 1.779578 | 0.00616 | 0.067844 | 0.974 | 3092 | tags=46%, list=13%, signal=52% |
| GO_POSITIVE_REGULATION_OF_DEFENSE_RESPONSE_TO_VIRUS_BY_HOST | 22 | 0.548 | 1.563311 | 0.048319 | 0.125288 | 1 | 3069 | tags=41%, list=13%, signal=47% |
| GO_IMMUNOLOGICAL_SYNAPSE | 35 | 0.651923 | 1.758961 | 0.024194 | 0.068468 | 0.985 | 5089 | tags=54%, list=22%, signal=69% |
| GO_POSITIVE_REGULATION_OF_MULTI_ORGANISM_PROCESS | 494 | 0.468012 | 2.03806 | 0 | 0.17161 | 0.382 | 3923 | tags=25%, list=17%, signal=29% |

| | | | | | | | | |
|---|---|---|---|---|---|---|---|---|
| GO_T_CELL_MEDIATED_IMMUNITY | 93 | 0.613577 | 1.938337 | 0 | 0.077157 | 0.695 | 4275 | tags=48%, list=18%, signal=59% |
| GO_REGULATION_OF_NATURAL_KILLER_CELL_MEDIATED_IMMUNITY | 36 | 0.701648 | 1.753554 | 0.00813 | 0.068477 | 0.986 | 3923 | tags=58%, list=17%, signal=70% |
| GO_REGULATION_OF_MACROPHAGE_MIGRATION | 35 | 0.645928 | 1.668615 | 0.016495 | 0.087813 | 1 | 2643 | tags=43%, list=11%, signal=48% |
| GO_LEUKOCYTE_CELL_CELL_ADHESION | 313 | 0.588336 | 1.984448 | 0 | 0.109311 | 0.564 | 4239 | tags=43%, list=18%, signal=52% |
| GO_CYTOKINE_SECRETION | 217 | 0.623457 | 1.945327 | 0 | 0.08173 | 0.675 | 3548 | tags=44%, list=15%, signal=52% |
| GO_LYMPHOCYTE_MEDIATED_IMMUNITY | 235 | 0.607805 | 1.91241 | 0.006073 | 0.070882 | 0.769 | 4275 | tags=46%, list=18%, signal=56% |
| GO_RESPONSE_TO_INTERFERON_GAMMA | 166 | 0.577902 | 1.974359 | 0.004057 | 0.09448 | 0.599 | 3402 | tags=34%, list=14%, signal=39% |
| GO_CD8_POSITIVE_ALPHA_BETA_T_CELL_ACTIVATION | 19 | 0.711396 | 1.709825 | 0.010482 | 0.07694 | 0.998 | 4362 | tags=58%, list=19%, signal=71% |
| GO_SYNCYTIUM_FORMATION | 50 | 0.605592 | 1.786735 | 0 | 0.065923 | 0.969 | 1565 | tags=30%, list=7%, signal=32% |
| GO_REGULATION_OF_IMMUNE_EFFECTOR_PROCESS | 360 | 0.570686 | 1.978365 | 0.002012 | 0.099391 | 0.589 | 4281 | tags=40%, list=18%, signal=48% |
| GO_MYOBLAST_FUSION | 37 | 0.589702 | 1.678016 | 0.002053 | 0.084937 | 1 | 1565 | tags=30%, list=7%, signal=32% |

| | | | | | | | | |
|---|---|---|---|---|---|---|---|---|
| GO_REGULATION_OF_LYMPHOCYTE_ACTIVATION | 398 | 0.586384 | 1.998357 | 0 | 0.129799 | 0.502 | 4362 | tags=43%, list=19%, signal=52% |
| GO_LEUKOCYTE_PROLIFERATION | 280 | 0.575534 | 1.895628 | 0 | 0.061736 | 0.817 | 4334 | tags=45%, list=18%, signal=54% |
| GO_PRODUCTION_OF_MOLECULAR_MEDIATOR_OF_IMMUNE_RESPONSE | 190 | 0.579545 | 1.963584 | 0 | 0.089149 | 0.624 | 4549 | tags=42%, list=19%, signal=52% |
| GO_POSITIVE_REGULATION_OF_T_CELL_PROLIFERATION | 87 | 0.649425 | 1.866554 | 0.0020333 | 0.057589 | 0.879 | 3035 | tags=45%, list=13%, signal=51% |
| GO_POSITIVE_REGULATION_OF_MYELOID_LEUKOCYTE_DIFFERENTIATION | 54 | 0.520849 | 1.703765 | 0.0082644 | 0.078182 | 0.999 | 1698 | tags=26%, list=7%, signal=28% |
| GO_POSITIVE_REGULATION_OF_TUMOR_NECROSIS_FACTOR_SUPERFAMILY_CYTOKINE_PRODUCTION | 79 | 0.672967 | 1.873587 | 0.0020092 | 0.058673 | 0.872 | 3548 | tags=49%, list=15%, signal=58% |
| GO_POSITIVE_REGULATION_OF_CD4_POSITIVE_ALPHA_BETA_T_CELL_ACTIVATION | 35 | 0.627699 | 1.778788 | 0.0143744 | 0.067678 | 0.974 | 2645 | tags=40%, list=11%, signal=45% |
| GO_REGULATION_OF_LEUKOCYTE_MEDIATED_CYTOTOXICITY | 66 | 0.64233 | 1.772355 | 0.0124448 | 0.068586 | 0.98 | 4275 | tags=53%, list=18%, signal=65% |
| GO_INTERLEUKIN_12_PRODUCTION | 56 | 0.701077 | 1.926458 | 0 | 0.071021 | 0.734 | 3673 | tags=52%, list=16%, signal=61% |
| GO_REGULATION_OF_LEUKOCYTE_MIGRATION | 183 | 0.60286 | 1.942392 | 0 | 0.079285 | 0.684 | 2829 | tags=37%, list=12%, signal=42% |
| GO_INTERLEUKIN_8_SECRETION | 30 | 0.73284 | 1.875587 | 0 | 0.058466 | 0.869 | 3305 | tags=47%, list=14%, signal=54% |
| GO_REGULATION_OF_LEUKOCYTE_MEDIATED_IMMUNITY | 183 | 0.605721 | 1.973216 | 0 | 0.093297 | 0.602 | 4599 | tags=48%, list=20%, signal=59% |
| GO_T_CELL_MIGRATION | 61 | 0.724322 | 1.966808 | 0 | 0.095021 | 0.619 | 2508 | tags=48%, list=11%, signal=53% |

| | | | | | | | | |
|---|---|---|---|---|---|---|---|---|
| GO_REGULATION_OF_MYELOID_LEUKOCYTE_MEDIATED_IMMUNITY | 51 | 0.642674 | 2.013018 | 0 | 0.131764 | 0.448 | 1621 | tags=29%, list=7%, signal=32% |
| GO_REGULATION_OF_RESPONSE_TO_CYTOKINE_STIMULUS | 159 | 0.496183 | 1.995088 | 0.002045 | 0.125725 | 0.523 | 4232 | tags=33%, list=18%, signal=40% |
| GO_LYMPHOCYTE_HOMEOSTASIS | 61 | 0.563441 | 1.851099 | 0.008602 | 0.058424 | 0.896 | 3955 | tags=36%, list=17%, signal=43% |
| GO_NEGATIVE_REGULATION_OF_CYTOKINE_PRODUCTION | 251 | 0.592826 | 1.983741 | 0 | 0.107325 | 0.569 | 3855 | tags=40%, list=16%, signal=48% |
| GO_FC_RECEPTOR_MEDIATED_STIMULATORY_SIGNALING_PATHWAY | 83 | 0.509124 | 1.903836 | 0.012552 | 0.064123 | 0.79 | 3387 | tags=29%, list=14%, signal=34% |
| GO_LYMPHOCYTE_MIGRATION | 100 | 0.661819 | 1.869935 | 0.002012 | 0.059384 | 0.875 | 2993 | tags=46%, list=13%, signal=52% |
| GO_REGULATION_OF_CD4_POSITIVE_ALPHA_BETA_T_CELL_DIFFERENTIATION | 44 | 0.53156 | 1.633093 | 0.028689 | 0.099715 | 1 | 4909 | tags=45%, list=21%, signal=57% |
| GO_LEUKOCYTE_MEDIATED_CYTOTOXICITY | 96 | 0.594458 | 1.784202 | 0.006224 | 0.066371 | 0.969 | 4275 | tags=46%, list=18%, signal=56% |
| GO_INTERLEUKIN_8_PRODUCTION | 74 | 0.628637 | 1.886038 | 0 | 0.061482 | 0.836 | 3305 | tags=39%, list=14%, signal=45% |
| GO_ANTIGEN_PROCESSING_AND_PRESENTATION_OF_EXOGENOUS_PEPTIDE_ANTIGEN_VIA_MHC_CLASS_I | 68 | 0.5407 | 2.034859 | 0.002114 | 0.164181 | 0.388 | 1933 | tags=15%, list=8%, signal=16% |

| | | | | | | | | |
|---|---|---|---|---|---|---|---|---|
| GO_REGULATION_OF_LYMPHOCYTE_DIFFERENTIATION | 162 | 0.547855 | 1.899529 | 0.004124 | 0.063183 | 0.803 | 4909 | tags=45%, list=21%, signal=57% |
| GO_POSITIVE_REGULATION_OF_LEUKOCYTE_PROLIFERATION | 127 | 0.634533 | 1.886504 | 0 | 0.061542 | 0.836 | 3035 | tags=43%, list=13%, signal=49% |
| GO_REGULATION_OF_T_CELL_DIFFERENTIATION | 131 | 0.534219 | 1.82347 | 0.010526 | 0.060234 | 0.926 | 4362 | tags=42%, list=19%, signal=51% |
| GO_POSITIVE_REGULATION_OF_CD4_POSITIVE_ALPHA_BETA_T_CELL_DIFFERENTIATION | 29 | 0.636804 | 1.755818 | 0.022965 | 0.068492 | 0.985 | 2645 | tags=41%, list=11%, signal=47% |
| GO_REGULATION_OF_LYMPHOCYTE_MIGRATION | 58 | 0.643304 | 1.877324 | 0 | 0.060429 | 0.864 | 2993 | tags=40%, list=13%, signal=45% |
| GO_T_CELL_SELECTION | 47 | 0.591107 | 1.584288 | 0.045549 | 0.116747 | 1 | 4533 | tags=49%, list=19%, signal=60% |
| GO_POSITIVE_REGULATION_OF_LEUKOCYTE_MIGRATION | 123 | 0.666063 | 2.026164 | 0 | 0.149213 | 0.407 | 2722 | tags=42%, list=12%, signal=48% |
| GO_LIPOPOLYSACCHARIDE_MEDIATED_SIGNALING_PATHWAY | 56 | 0.604851 | 1.844236 | 0.002096 | 0.05828 | 0.906 | 2394 | tags=36%, list=10%, signal=40% |
| GO_NEGATIVE_REGULATION_OF_REGULATED_SECRETORY_PATHWAY | 21 | 0.676505 | 1.832148 | 0.002012 | 0.05986 | 0.918 | 2052 | tags=33%, list=9%, signal=36% |
| GO_INTERLEUKIN_1_BETA_PRODUCTION | 82 | 0.688958 | 1.936436 | 0.002024 | 0.074855 | 0.701 | 3548 | tags=55%, list=15%, signal=64% |

| | | | | | | | | |
|---|---|---|---|---|---|---|---|---|
| GO_REGULATION_OF_CELL_KILLING | 87 | 0.636137 | 1.830969 | 0.006085 | 0.059738 | 0.92 | 4275 | tags=49%, list=18%, signal=60% |
| GO_POSITIVE_REGULATION_OF_CYTOKINE_SECRETION | 128 | 0.627197 | 1.902245 | 0 | 0.064486 | 0.795 | 3548 | tags=45%, list=15%, signal=52% |
| GO_NEGATIVE_REGULATION_OF_IMMUNE_SYSTEM_PROCESS | 409 | 0.540242 | 1.955147 | 0 | 0.086628 | 0.648 | 4723 | tags=39%, list=20%, signal=48% |
| GO_REGULATION_OF_SYNCYTIUM_FORMATION_BY_PLASMA_MEMBRANE_FUSION | 27 | 0.678339 | 1.702274 | 0.002132 | 0.078219 | 0.999 | 1310 | tags=41%, list=6%, signal=43% |
| GO_NIK_NF_KAPPAB_SIGNALING | 156 | 0.441959 | 2.028258 | 0.002004 | 0.154353 | 0.403 | 3264 | tags=20%, list=14%, signal=23% |
| GO_ANTIGEN_PROCESSING_AND_PRESENTATION_OF_PEPTIDE_ANTIGEN_VIA_MHC_CLASS_I | 89 | 0.503121 | 2.02274 | 0.008368 | 0.132298 | 0.422 | 3892 | tags=20%, list=17%, signal=24% |
| GO_POSITIVE_REGULATION_OF_NATURAL_KILLER_CELL_MEDIATED_CYTOTOXICITY | 19 | 0.702196 | 1.623173 | 0.034205 | 0.1028 | 1 | 3923 | tags=58%, list=17%, signal=69% |
| GO_INTERFERON_GAMMA_MEDIATED_SIGNALING_PATHWAY | 70 | 0.514441 | 1.772402 | 0.025918 | 0.068751 | 0.98 | 3953 | tags=34%, list=17%, signal=41% |
| GO_POSITIVE_REGULATION_OF_CELL_ADHESION | 376 | 0.532868 | 1.944265 | 0 | 0.079178 | 0.678 | 4511 | tags=39%, list=19%, signal=48% |
| GO_POSITIVE_REGULATION_OF_TUMOR_NECROSIS_FACTOR_BIOSYNTHETIC_PROCESS | 20 | 0.706414 | 1.694775 | 0.010331 | 0.080729 | 0.999 | 553 | tags=40%, list=2%, signal=41% |

| | | | | | | | | |
|---|---|---|---|---|---|---|---|---|
| GO_REGULATION_OF_MYELOID_CELL_DIFFERENTIATION | 208 | 0.41527 | 1.754606 | 0.0060061 | 0.068327 | 0.985 | 3855 | tags=25%, list=16%, signal=29% |
| GO_POSITIVE_REGULATION_OF_OSTEOCLAST_DIFFERENTIATION | 26 | 0.545977 | 1.554348 | 0.0440888 | 0.127963 | 1 | 5788 | tags=50%, list=25%, signal=66% |
| GO_I_KAPPAB_PHOSPHORYLATION | 18 | 0.653138 | 1.753719 | 0.0203677 | 0.068533 | 0.986 | 2666 | tags=33%, list=11%, signal=38% |
| GO_POSITIVE_REGULATION_OF_NATURAL_KILLER_CELL_MEDIATED_IMMUNITY | 22 | 0.706093 | 1.638585 | 0.0222267 | 0.09699 | 1 | 3923 | tags=59%, list=17%, signal=71% |
| GO_PRODUCTION_OF_MOLECULAR_MEDIATOR_INVOLVED_IN_INFLAMMATORY_RESPONSE | 63 | 0.549873 | 1.707486 | 0.0082999 | 0.077206 | 0.999 | 2794 | tags=32%, list=12%, signal=36% |
| GO_POSITIVE_REGULATION_OF_LEUKOCYTE_MEDIATED_CYTOTOXICITY | 44 | 0.635764 | 1.676248 | 0.0225587 | 0.085682 | 1 | 4275 | tags=52%, list=18%, signal=64% |
| GO_REGULATION_OF_MAST_CELL_ACTIVATION | 38 | 0.628639 | 1.899546 | 0 | 0.06364 | 0.803 | 2478 | tags=32%, list=11%, signal=35% |
| GO_REGULATION_OF_MACROPHAGE_CHEMOTAXIS | 24 | 0.621815 | 1.567738 | 0.0289266 | 0.12308 | 1 | 4315 | tags=54%, list=18%, signal=66% |
| GO_INTERLEUKIN_1_PRODUCTION | 95 | 0.66467 | 1.895147 | 0.0020028 | 0.061298 | 0.818 | 3548 | tags=53%, list=15%, signal=62% |

| | | | | | | | | |
|---|---|---|---|---|---|---|---|---|
| GO_NEGATIVE_REGULATION_OF_EXOCYTOSIS | 28 | 0.573507 | 1.709834 | 0.006024 | 0.077075 | 0.998 | 2052 | tags=25%, list=9%, signal=27% |
| GO_MHC_PROTEIN_BINDING | 23 | 0.845704 | 1.896395 | 0 | 0.06218 | 0.813 | 382 | tags=39%, list=2%, signal=40% |
| GO_T_CELL_MEDIATED_CYTOTOXICITY | 37 | 0.612745 | 1.754113 | 0.006198 | 0.068418 | 0.985 | 4275 | tags=46%, list=18%, signal=56% |
| GO_POSITIVE_REGULATION_OF_CYTOKINE_BIOSYNTHETIC_PROCESS | 66 | 0.654222 | 1.879447 | 0 | 0.060579 | 0.86 | 4747 | tags=56%, list=20%, signal=70% |
| GO_CELLULAR_DEFENSE_RESPONSE | 48 | 0.763495 | 1.937914 | 0 | 0.076522 | 0.697 | 2564 | tags=50%, list=11%, signal=56% |
| GO_REGULATION_OF_T_CELL_MIGRATION | 41 | 0.662101 | 1.892315 | 0.003861 | 0.061214 | 0.823 | 2492 | tags=37%, list=11%, signal=41% |
| GO_TUMOR_NECROSIS_FACTOR_MEDIATED_SIGNALING_PATHWAY | 150 | 0.396032 | 1.734128 | 0.012632 | 0.072147 | 0.992 | 4549 | tags=29%, list=19%, signal=35% |
| GO_RESPIRATORY_BURST | 30 | 0.693429 | 1.810416 | 0.006211 | 0.062349 | 0.943 | 2727 | tags=40%, list=12%, signal=45% |
| GO_MYELOID_LEUKOCYTE_DIFFERENTIATION | 196 | 0.476119 | 1.813922 | 0.002079 | 0.061619 | 0.937 | 3855 | tags=30%, list=16%, signal=36% |
| GO_DENDRITIC_CELL_DIFFERENTIATION | 39 | 0.695099 | 1.982121 | 0 | 0.103371 | 0.572 | 972 | tags=28%, list=4%, signal=29% |
| GO_POSITIVE_REGULATION_OF_LYMPHOCYTE_MEDIATED_IMMUNITY | 89 | 0.61552 | 1.852553 | 0.008114 | 0.058693 | 0.895 | 4549 | tags=49%, list=19%, signal=61% |

| | | | | | | | | |
|---|---|---|---|---|---|---|---|---|
| GO_REGULATION_OF_T_HELPER_CELL_DIFFERENTIATION | 33 | 0.523356 | 1.598148 | 0.033932 | 0.112313 | 1 | 1290 | tags=27%, list=5%, signal=29% |
| GO_LEUKOCYTE_ACTIVATION_INVOLVED_IN_INFLAMMATORY_RESPONSE | 42 | 0.742282 | 1.778511 | 0 | 0.067254 | 0.974 | 2413 | tags=55%, list=10%, signal=61% |
| GO_TYPE_I_INTERFERON_PRODUCTION | 119 | 0.509189 | 2.049263 | 0.002088 | 0.182377 | 0.352 | 4941 | tags=31%, list=21%, signal=39% |
| GO_INTERLEUKIN_2_PRODUCTION | 62 | 0.652755 | 1.814285 | 0.006224 | 0.061652 | 0.937 | 3285 | tags=45%, list=14%, signal=52% |
| GO_REGULATION_OF_CELL_CELL_ADHESION | 375 | 0.531037 | 1.929867 | 0 | 0.071655 | 0.722 | 4238 | tags=37%, list=18%, signal=44% |
| GO_REGULATION_OF_CD4_POSITIVE_ALPHA_BETA_T_CELL_ACTIVATION | 54 | 0.560987 | 1.730911 | 0.018947 | 0.072701 | 0.992 | 2645 | tags=35%, list=11%, signal=40% |
| GO_RESPONSE_TO_AMYLOID_BETA | 45 | 0.690976 | 1.855044 | 0 | 0.058557 | 0.891 | 4174 | tags=56%, list=18%, signal=67% |
| GO_INTERFERON_BETA_PRODUCTION | 49 | 0.577751 | 1.967324 | 0.002066 | 0.096467 | 0.618 | 4941 | tags=35%, list=21%, signal=44% |
| GO_REGULATION_OF_LYMPHOCYTE_MEDIATED_IMMUNITY | 133 | 0.613178 | 1.895272 | 0.006061 | 0.061581 | 0.818 | 4599 | tags=51%, list=20%, signal=63% |
| GO_RESPONSE_TO_INTERFERON_BETA | 26 | 0.646143 | 1.768265 | 0.010504 | 0.067433 | 0.98 | 3189 | tags=38%, list=14%, signal=44% |

| | | | | | | | | |
|---|---|---|---|---|---|---|---|---|
| GO_CELL_KILLING | 146 | 0.568462 | 1.765632 | 0.0061 48 | 0.068208 | 0.981 | 4278 | tags=43%, list=18%, signal=52% |
| GO_MYD88_DEPENDENT_TOLL_LIKE_RECEPTOR_SIGNALING_PATHWAY | 35 | 0.794742 | 1.921054 | 0 | 0.07185 | 0.751 | 3153 | tags=51%, list=13%, signal=59% |
| GO_POSITIVE_REGULATION_OF_DEFENSE_RESPONSE | 458 | 0.497716 | 1.941706 | 0.002028 | 0.075528 | 0.685 | 4341 | tags=31%, list=18%, signal=38% |
| GO_POSITIVE_REGULATION_OF_LEUKOCYTE_MEDIATED_IMMUNITY | 117 | 0.59547 | 1.92009 | 0.004065 | 0.070434 | 0.754 | 2645 | tags=35%, list=11%, signal=39% |
| GO_POSITIVE_REGULATION_OF_MACROPHAGE_ACTIVATION | 22 | 0.712065 | 1.780912 | 0 | 0.067507 | 0.972 | 3153 | tags=50%, list=13%, signal=58% |
| GO_POSITIVE_REGULATION_OF_CELL_KILLING | 57 | 0.64023 | 1.746535 | 0.014315 | 0.068865 | 0.991 | 4275 | tags=49%, list=18%, signal=60% |
| GO_B_CELL_ACTIVATION_INVOLVED_IN_IMMUNE_RESPONSE | 67 | 0.545324 | 1.746021 | 0.018145 | 0.068985 | 0.991 | 3916 | tags=39%, list=17%, signal=46% |
| GO_LEUKOCYTE_HOMEOSTASIS | 83 | 0.575811 | 1.862567 | 0.006356 | 0.056802 | 0.884 | 4251 | tags=40%, list=18%, signal=48% |
| GO_INTERLEUKIN_10_PRODUCTION | 50 | 0.752161 | 2.025252 | 0 | 0.14326 | 0.412 | 1948 | tags=48%, list=8%, signal=52% |
| GO_FICOLIN_1_RICH_GRANULE_MEMBRANE | 57 | 0.584689 | 1.750275 | 0.008214 | 0.06859 | 0.988 | 4331 | tags=46%, list=18%, signal=56% |

| | | | | | | | | |
|---|---|---|---|---|---|---|---|---|
| GO_REGULATION_OF_MYOBLAST_FUSION | 20 | 0.661876 | 1.619583 | 0.0171131 | 0.103929 | 1 | 1310 | tags=40%, list=6%, signal=42% |
| GO_LEUKOCYTE_MIGRATION | 413 | 0.581843 | 1.965646 | 0 | 0.092319 | 0.622 | 2924 | tags=36%, list=12%, signal=40% |
| GO_LEUKOCYTE_CHEMOTAXIS | 200 | 0.630229 | 1.864378 | 0.0020062 | 0.057049 | 0.882 | 2924 | tags=42%, list=12%, signal=48% |
| GO_PHAGOCYTIC_CUP | 24 | 0.625792 | 1.663783 | 0.0220044 | 0.089897 | 1 | 2852 | tags=42%, list=12%, signal=47% |
| GO_POSITIVE_REGULATION_OF_INTERLEUKIN_12_PRODUCTION | 35 | 0.675803 | 1.768622 | 0.00207 | 0.067726 | 0.98 | 2393 | tags=43%, list=10%, signal=48% |
| GO_REGULATION_OF_LEUKOCYTE_PROLIFERATION | 206 | 0.611642 | 1.900205 | 0 | 0.064231 | 0.8 | 4334 | tags=49%, list=18%, signal=59% |
| GO_VIRAL_GENOME_REPLICATION | 113 | 0.469629 | 1.959311 | 0.0021055 | 0.087708 | 0.636 | 3189 | tags=21%, list=14%, signal=24% |
| GO_POSITIVE_REGULATION_OF_NF_KAPPAB_TRANSCRIPTION_FACTOR_ACTIVITY | 143 | 0.5071 | 1.954644 | 0.0019984 | 0.08553 | 0.651 | 3539 | tags=28%, list=15%, signal=33% |
| GO_MACROPHAGE_ACTIVATION | 82 | 0.692112 | 1.860864 | 0 | 0.057418 | 0.884 | 4341 | tags=60%, list=18%, signal=73% |
| GO_OSTEOCLAST_DIFFERENTIATION | 94 | 0.503967 | 1.806591 | 0.0019984 | 0.063086 | 0.945 | 4511 | tags=37%, list=19%, signal=46% |
| GO_NITRIC_OXIDE_SYNTHASE_BIOSYNTHETIC_PROCESS | 18 | 0.735554 | 1.74225 | 0.00409 | 0.069927 | 0.992 | 1789 | tags=44%, list=8%, signal=48% |

| | | | | | | | | |
|---|---|---|---|---|---|---|---|---|
| GO_POSITIVE_REGULATION_OF_T_CELL_MIGRATION | 29 | 0.761823 | 1.877161 | 0.002041 | 0.060165 | 0.865 | 1554 | tags=41%, list=7%, signal=44% |
| GO_NEGATIVE_REGULATION_OF_INTERLEUKIN_10_PRODUCTION | 17 | 0.824222 | 1.841381 | 0 | 0.058413 | 0.908 | 1290 | tags=65%, list=5%, signal=68% |
| GO_B_CELL_PROLIFERATION | 92 | 0.590112 | 1.769382 | 0.00616 | 0.068186 | 0.98 | 3196 | tags=40%, list=14%, signal=46% |
| GO_REGULATION_OF_B_CELL_RECEPTOR_SIGNALING_PATHWAY | 26 | 0.612265 | 1.557249 | 0.042254 | 0.127373 | 1 | 4405 | tags=46%, list=19%, signal=57% |
| GO_T_CELL_CYTOKINE_PRODUCTION | 40 | 0.616521 | 1.682396 | 0.00431 | 0.082835 | 0.999 | 3189 | tags=48%, list=14%, signal=55% |
| GO_ANTIGEN_BINDING | 44 | 0.690416 | 1.72892 | 0.008299 | 0.072669 | 0.993 | 4040 | tags=59%, list=17%, signal=71% |
| GO_NATURAL_KILLER_CELL_MEDIATED_IMMUNITY | 54 | 0.662679 | 1.751839 | 0.010288 | 0.068395 | 0.986 | 4909 | tags=57%, list=21%, signal=72% |
| GO_REGULATION_OF_ADAPTIVE_IMMUNE_RESPONSE | 147 | 0.579474 | 1.931096 | 0.004073 | 0.073958 | 0.717 | 4909 | tags=50%, list=21%, signal=62% |
| GO_POSITIVE_REGULATION_OF_ADAPTIVE_IMMUNE_RESPONSE | 93 | 0.62381 | 1.945047 | 0.002066 | 0.080888 | 0.677 | 2801 | tags=39%, list=12%, signal=44% |
| GO_REGULATION_OF_INTERLEUKIN_4_PRODUCTION | 26 | 0.648298 | 1.685297 | 0.01217 | 0.081788 | 0.999 | 2983 | tags=42%, list=13%, signal=48% |

| | | | | | | | | |
|---|---|---|---|---|---|---|---|---|
| GO_POSITIVE_REGULATION_OF_MACROPHAGE_MIGRATION | 20 | 0.755584 | 1.691026 | 0 | 0.081041 | 0.999 | 2643 | tags=65%, list=11%, signal=73% |
| GO_REGULATION_OF_T_CELL_MEDIATED_IMMUNITY | 65 | 0.616653 | 1.829343 | 0.004141 | 0.059702 | 0.921 | 4275 | tags=49%, list=18%, signal=60% |
| GO_INTERLEUKIN_6_PRODUCTION | 139 | 0.629149 | 1.98872 | 0 | 0.114504 | 0.549 | 3548 | tags=42%, list=15%, signal=50% |
| GO_NEGATIVE_REGULATION_OF_INNATE_IMMUNE_RESPONSE | 51 | 0.627183 | 1.948698 | 0 | 0.085142 | 0.666 | 3908 | tags=41%, list=17%, signal=49% |
| GO_NEGATIVE_REGULATION_OF_MULTI_ORGANISM_PROCESS | 195 | 0.497169 | 1.948146 | 0.002092 | 0.084303 | 0.669 | 3908 | tags=30%, list=17%, signal=35% |
| GO_POSITIVE_REGULATION_OF_INTERLEUKIN_1_PRODUCTION | 52 | 0.654685 | 1.754717 | 0.004149 | 0.068424 | 0.985 | 3548 | tags=48%, list=15%, signal=56% |
| GO_PHOSPHATIDYLINOSITOL_3_4_BISPHOSPHATE_BINDING | 26 | 0.483683 | 1.540555 | 0.034188 | 0.133996 | 1 | 3386 | tags=27%, list=14%, signal=31% |
| GO_MACROPHAGE_MIGRATION | 46 | 0.619902 | 1.660613 | 0.014286 | 0.09051 | 1 | 2722 | tags=43%, list=12%, signal=49% |
| GO_INTERLEUKIN_4_PRODUCTION | 33 | 0.671711 | 1.786138 | 0.00202 | 0.066035 | 0.969 | 2983 | tags=45%, list=13%, signal=52% |
| GO_POSITIVE_REGULATION_OF_INTERLEUKIN_8_PRODUCTION | 47 | 0.66382 | 1.909882 | 0.004141 | 0.070554 | 0.773 | 4902 | tags=49%, list=21%, signal=62% |
| GO_POSITIVE_REGULATION_OF_LYMPHOCYTE_MIGRATION | 35 | 0.758851 | 1.907721 | 0 | 0.066496 | 0.78 | 1554 | tags=43%, list=7%, signal=46% |

| | | | | | | | | |
|---|---|---|---|---|---|---|---|---|
| GO_CELL_CHEMOTAXIS | 270 | 0.586707 | 1.841273 | 0.0041667 | 0.05822 | 0.908 | 2722 | tags=36%, list=12%, signal=40% |
| GO_POSITIVE_REGULATION_OF_INTERLEUKIN_8_SECRETION | 16 | 0.770907 | 1.769643 | 0.0063422 | 0.068221 | 0.98 | 1789 | tags=44%, list=8%, signal=47% |
| GO_POSITIVE_REGULATION_OF_LEUKOCYTE_CHEMOTAXIS | 82 | 0.692237 | 1.959043 | 0 | 0.086372 | 0.636 | 2722 | tags=45%, list=12%, signal=51% |
| GO_NEGATIVE_REGULATION_OF_RESPONSE_TO_BIOTIC_STIMULUS | 84 | 0.535774 | 1.909543 | 0.0020204 | 0.069529 | 0.773 | 3908 | tags=33%, list=17%, signal=40% |
| GO_T_CELL_HOMEOSTASIS | 38 | 0.548801 | 1.756751 | 0.0107776 | 0.068652 | 0.985 | 2426 | tags=26%, list=10%, signal=29% |
| GO_REGULATION_OF_CYTOKINE_PRODUCTION_INVOLVED_IN_IMMUNE_RESPONSE | 79 | 0.695878 | 1.928469 | 0 | 0.070442 | 0.728 | 4533 | tags=62%, list=19%, signal=77% |
| GO_NEGATIVE_REGULATION_OF_B_CELL_PROLIFERATION | 16 | 0.734367 | 1.715912 | 0.01222 | 0.075742 | 0.997 | 2499 | tags=44%, list=11%, signal=49% |
| GO_MYELOID_CELL_DIFFERENTIATION | 368 | 0.393076 | 1.800709 | 0.0040665 | 0.063973 | 0.948 | 3239 | tags=20%, list=14%, signal=23% |
| GO_HOMEOSTASIS_OF_NUMBER_OF_CELLS | 232 | 0.463884 | 1.985807 | 0 | 0.110763 | 0.558 | 3955 | tags=27%, list=17%, signal=32% |
| GO_POSITIVE_REGULATION_OF_INTERLEUKIN_4_PRODUCTION | 21 | 0.679295 | 1.650391 | 0.0080997 | 0.09402 | 1 | 2983 | tags=48%, list=13%, signal=54% |
| GO_B_CELL_HOMEOSTASIS | 28 | 0.570287 | 1.732911 | 0.02449 | 0.072451 | 0.992 | 3754 | tags=32%, list=16%, signal=38% |

| | | | | | | | | |
|---|---|---|---|---|---|---|---|---|
| GO_NEGATIVE_REGULATION_OF_VIRAL_GENOME_REPLICATION | 52 | 0.566007 | 1.789739 | 0.0082822 | 0.065478 | 0.965 | 3492 | tags=29%, list=15%, signal=34% |
| GO_REGULATION_OF_T_CELL_MEDIATED_CYTOTOXICITY | 29 | 0.653364 | 1.732421 | 0.0081477 | 0.072262 | 0.992 | 2080 | tags=38%, list=9%, signal=42% |
| GO_REGULATION_OF_PRODUCTION_OF_MOLECULAR_MEDIATOR_OF_IMMUNE_RESPONSE | 128 | 0.628819 | 1.975897 | 0 | 0.097337 | 0.596 | 4549 | tags=48%, list=19%, signal=60% |
| GO_REGULATION_OF_LEUKOCYTE_CHEMOTAXIS | 104 | 0.673402 | 1.931448 | 0.0020445 | 0.074578 | 0.716 | 2722 | tags=43%, list=12%, signal=49% |
| GO_B_CELL_MEDIATED_IMMUNITY | 111 | 0.602822 | 1.845072 | 0.0097728 | 0.058901 | 0.904 | 2938 | tags=39%, list=12%, signal=44% |
| GO_CELLULAR_RESPONSE_TO_BIOTIC_STIMULUS | 218 | 0.558834 | 1.894193 | 0 | 0.061516 | 0.819 | 3291 | tags=32%, list=14%, signal=37% |
| GO_SPECIFIC_GRANULE_MEMBRANE | 87 | 0.590158 | 1.917644 | 0.0041244 | 0.069324 | 0.759 | 1327 | tags=24%, list=6%, signal=25% |
| GO_RESPONSE_TO_CHEMOKINE | 88 | 0.689443 | 1.806724 | 0.0020833 | 0.063239 | 0.945 | 2421 | tags=47%, list=10%, signal=52% |
| GO_B_CELL_ACTIVATION | 238 | 0.536521 | 1.867156 | 0.00611 | 0.058219 | 0.878 | 4549 | tags=39%, list=19%, signal=48% |
| GO_POSITIVE_REGULATION_OF_INTERLEUKIN_2_PRODUCTION | 30 | 0.705601 | 1.803561 | 0.0060998 | 0.063679 | 0.947 | 2983 | tags=50%, list=13%, signal=57% |

| | | | | | | | | |
|---|---|---|---|---|---|---|---|---|
| GO_RECEPTOR_SIGNALING_PATHWAY_VIA_STAT | 152 | 0.441949 | 1.674814 | 0.006303 | 0.086159 | 1 | 3798 | tags=32%, list=16%, signal=38% |
| GO_POSITIVE_REGULATION_OF_RESPONSE_TO_CYTOKINE_STIMULUS | 51 | 0.591216 | 1.900367 | 0.004132 | 0.0646 | 0.8 | 4174 | tags=39%, list=18%, signal=48% |
| GO_POSITIVE_REGULATION_OF_CALCIUM_MEDIATED_SIGNALING | 39 | 0.533957 | 1.60993 | 0.011952 | 0.107834 | 1 | 3883 | tags=41%, list=17%, signal=49% |
| GO_REGULATION_OF_NIK_NF_KAPPAB_SIGNALING | 99 | 0.524569 | 1.962118 | 0 | 0.088578 | 0.627 | 1922 | tags=23%, list=8%, signal=25% |
| GO_CYTOKINE_METABOLIC_PROCESS | 111 | 0.589876 | 1.87584 | 0 | 0.058695 | 0.867 | 3153 | tags=39%, list=13%, signal=45% |
| GO_POSITIVE_REGULATION_OF_ALPHA_BETA_T_CELL_PROLIFERATION | 18 | 0.736754 | 1.729096 | 0.007905 | 0.072853 | 0.993 | 1290 | tags=44%, list=5%, signal=47% |
| GO_POSITIVE_REGULATION_OF_NITRIC_OXIDE_SYNTHASE_BIOSYNTHETIC_PROCESS | 15 | 0.755689 | 1.642909 | 0.006198 | 0.09615 | 1 | 1789 | tags=53%, list=8%, signal=58% |
| GO_REGULATION_OF_B_CELL_PROLIFERATION | 60 | 0.669057 | 1.851757 | 0.004158 | 0.05861 | 0.896 | 2801 | tags=45%, list=12%, signal=51% |
| GO_NEGATIVE_REGULATION_OF_VIRAL_LIFE_CYCLE | 74 | 0.510536 | 1.762204 | 0.014523 | 0.068734 | 0.982 | 3675 | tags=27%, list=16%, signal=32% |
| GO_POSITIVE_REGULATION_OF_TYPE_I_INTERFERON_PRODUCTION | 74 | 0.493459 | 1.94439 | 0.006289 | 0.080085 | 0.678 | 3673 | tags=26%, list=16%, signal=30% |

| | | | | | | | | |
|---|---|---|---|---|---|---|---|---|
| GO_T_CELL_APOPTOTIC_PROCESS | 48 | 0.513928 | 1.749218 | 0.0126632 | 0.068387 | 0.989 | 3475 | tags=29%, list=15%, signal=34% |
| GO_POSITIVE_REGULATION_OF_MYELOID_CELL_DIFFERENTIATION | 86 | 0.483132 | 1.77778 | 0.0062899 | 0.067479 | 0.976 | 2356 | tags=24%, list=10%, signal=27% |
| GO_MYELOID_LEUKOCYTE_MIGRATION | 190 | 0.633889 | 1.857782 | 0 | 0.057523 | 0.889 | 2924 | tags=42%, list=12%, signal=48% |
| GO_NEGATIVE_REGULATION_OF_CELL_ACTIVATION | 182 | 0.600714 | 1.910591 | 0.0021055 | 0.070628 | 0.772 | 4362 | tags=44%, list=19%, signal=54% |
| GO_PROTEIN_AUTOPHOSPHORYLATION | 223 | 0.28812 | 1.387363 | 0.0418841 | 0.215689 | 1 | 1999 | tags=11%, list=8%, signal=12% |
| GO_RESPONSE_TO_MOLECULE_OF_BACTERIAL_ORIGIN | 322 | 0.541392 | 1.891498 | 0 | 0.061307 | 0.825 | 3802 | tags=34%, list=16%, signal=41% |
| GO_T_CELL_RECEPTOR_COMPLEX | 21 | 0.735579 | 1.620389 | 0.0365585 | 0.103599 | 1 | 912 | tags=48%, list=4%, signal=49% |
| GO_TUMOR_NECROSIS_FACTOR_RECEPTOR_SUPERFAMILY_BINDING | 41 | 0.460919 | 1.628771 | 0.02045 | 0.101137 | 1 | 4549 | tags=32%, list=19%, signal=39% |
| GO_REGULATION_OF_MYOTUBE_DIFFERENTIATION | 49 | 0.540352 | 1.642877 | 0.0101163 | 0.096035 | 1 | 2052 | tags=27%, list=9%, signal=29% |
| GO_POSITIVE_REGULATION_OF_PRODUCTION_OF_MOLECULAR_MEDIATOR_OF_IMMUNE_RESPONSE | 85 | 0.619927 | 1.993041 | 0 | 0.124034 | 0.533 | 4717 | tags=48%, list=20%, signal=60% |

| | | | | | | | | |
|---|---|---|---|---|---|---|---|---|
| GO_REGULATORY_T_CELL_DIFFERENTIATION | 28 | 0.744641 | 1.781818 | 0.008639 | 0.067438 | 0.972 | 4315 | tags=68%, list=18%, signal=83% |
| GO_NEGATIVE_REGULATION_OF_T_CELL_MEDIATED_IMMUNITY | 17 | 0.653706 | 1.521194 | 0.03719 | 0.142743 | 1 | 3714 | tags=53%, list=16%, signal=63% |
| GO_POSITIVE_REGULATION_OF_NIK_NF_KAPPAB_SIGNALING | 75 | 0.564514 | 2.024275 | 0 | 0.137378 | 0.414 | 1922 | tags=25%, list=8%, signal=27% |
| GO_MYELOID_DENDRITIC_CELL_ACTIVATION | 27 | 0.668017 | 1.724284 | 0.010638 | 0.073284 | 0.994 | 2770 | tags=41%, list=12%, signal=46% |
| GO_REGULATION_OF_T_CELL_CYTOKINE_PRODUCTION | 30 | 0.6596 | 1.731799 | 0.004193 | 0.072475 | 0.992 | 3189 | tags=53%, list=14%, signal=62% |
| GO_POSITIVE_REGULATION_OF_PHAGOCYTOSIS | 63 | 0.628592 | 1.850587 | 0.002049 | 0.058412 | 0.897 | 2976 | tags=41%, list=13%, signal=47% |
| GO_INTERLEUKIN_1_SECRETION | 53 | 0.660769 | 1.844477 | 0 | 0.058473 | 0.906 | 3548 | tags=51%, list=15%, signal=60% |
| GO_NEGATIVE_REGULATION_OF_ALPHA_BETA_T_CELL_ACTIVATION | 31 | 0.586977 | 1.715101 | 0.012685 | 0.075899 | 0.998 | 4723 | tags=45%, list=20%, signal=56% |
| GO_POSITIVE_REGULATION_OF_ANTIGEN_RECEPTOR_MEDIATED_SIGNALING_PATHWAY | 21 | 0.665158 | 1.658998 | 0.024145 | 0.090644 | 1 | 4232 | tags=52%, list=18%, signal=64% |
| GO_LYMPHOCYTE_CHEMOTAXIS | 52 | 0.698642 | 1.744175 | 0.006198 | 0.069588 | 0.991 | 2699 | tags=54%, list=11%, signal=61% |

| | | | | | | | | |
|---|---|---|---|---|---|---|---|---|
| GO_GLIAL_CELL_ACTIVATION | 49 | 0.731852 | 1.8707 | 0 | 0.059542 | 0.875 | 2413 | tags=49%, list=10%, signal=54% |
| GO_NEGATIVE_REGULATION_OF_INTERLEUKIN_12_PRODUCTION | 16 | 0.792028 | 1.741985 | 0 | 0.069776 | 0.992 | 3397 | tags=69%, list=14%, signal=80% |
| GO_REGULATION_OF_MONONUCLEAR_CELL_MIGRATION | 43 | 0.686775 | 1.73279 | 0 | 0.07223 | 0.992 | 2643 | tags=51%, list=11%, signal=58% |
| GO_POSITIVE_REGULATION_OF_SMALL_GTPASE_MEDIATED_SIGNAL_TRANSDUCTION | 70 | 0.465051 | 1.506332 | 0.03212 | 0.150912 | 1 | 4366 | tags=37%, list=19%, signal=45% |
| GO_POSITIVE_REGULATION_OF_INTERLEUKIN_6_PRODUCTION | 89 | 0.629386 | 1.929888 | 0 | 0.072413 | 0.722 | 3548 | tags=40%, list=15%, signal=47% |
| GO_ALPHA_BETA_T_CELL_PROLIFERATION | 29 | 0.663897 | 1.739327 | 0.016194 | 0.070428 | 0.992 | 3053 | tags=48%, list=13%, signal=55% |
| GO_NEGATIVE_REGULATION_OF_LYMPHOCYTE_ACTIVATION | 136 | 0.614635 | 1.904004 | 0 | 0.065048 | 0.789 | 1547 | tags=29%, list=7%, signal=31% |
| GO_THYMIC_T_CELL_SELECTION | 21 | 0.691895 | 1.641293 | 0.023529 | 0.096421 | 1 | 2983 | tags=52%, list=13%, signal=60% |
| GO_INTERLEUKIN_2_BIOSYNTHETIC_PROCESS | 22 | 0.679685 | 1.62855 | 0.034623 | 0.100889 | 1 | 2983 | tags=55%, list=13%, signal=62% |
| GO_PHOSPHATIDYLINOSITOL_3_4_5_TRISPHOSPHATE_BINDING | 36 | 0.49156 | 1.658701 | 0.018789 | 0.090719 | 1 | 3474 | tags=28%, list=15%, signal=33% |
| GO_PATTERN_RECOGNITION_RECEPTOR_ACTIVITY | 21 | 0.821017 | 1.776144 | 0 | 0.0681 | 0.978 | 1789 | tags=67%, list=8%, signal=72% |
| GO_NEGATIVE_REGULATION_OF_INTERFERON_GAMMA_PRODUCTION | 30 | 0.667242 | 1.670147 | 0.012448 | 0.087356 | 1 | 1789 | tags=40%, list=8%, signal=43% |

| | | | | | | | | |
|---|---|---|---|---|---|---|---|---|
| GO_NEGATIVE_REGULATION_OF_VIRAL_PROCESS | 89 | 0.499085 | 1.799775 | 0.006342 | 0.064274 | 0.949 | 3675 | tags=25%, list=16%, signal=29% |
| GO_SH2_DOMAIN_BINDING | 35 | 0.553236 | 1.790795 | 0.004193 | 0.065584 | 0.961 | 4902 | tags=40%, list=21%, signal=50% |
| GO_IMMUNOGLOBULIN_PRODUCTION | 101 | 0.564441 | 1.898503 | 0.002066 | 0.06283 | 0.806 | 2938 | tags=32%, list=12%, signal=36% |
| GO_GRANULOCYTE_MIGRATION | 128 | 0.663273 | 1.84254 | 0 | 0.058446 | 0.908 | 2397 | tags=45%, list=10%, signal=50% |
| GO_REGULATION_OF_PHAGOCYTOSIS | 86 | 0.601328 | 1.85689 | 0 | 0.057753 | 0.889 | 2727 | tags=36%, list=12%, signal=41% |
| GO_LEUKOCYTE_APOPTOTIC_PROCESS | 103 | 0.617217 | 2.044555 | 0 | 0.174599 | 0.364 | 3475 | tags=35%, list=15%, signal=41% |
| GO_POSITIVE_REGULATION_OF_CHEMOTAXIS | 130 | 0.637042 | 1.912386 | 0 | 0.070249 | 0.769 | 2319 | tags=37%, list=10%, signal=41% |
| GO_POSITIVE_REGULATION_OF_CALCIUM_ION_TRANSPORT_INTO_CYTOSOL | 51 | 0.58719 | 1.713756 | 0 | 0.075999 | 0.998 | 4341 | tags=53%, list=18%, signal=65% |
| GO_NEGATIVE_REGULATION_OF_LEUKOCYTE_CELL_CELL_ADHESION | 112 | 0.569803 | 1.848491 | 0.002083 | 0.05851 | 0.9 | 3220 | tags=35%, list=14%, signal=40% |
| GO_DEFENSE_RESPONSE_TO_BACTERIUM | 209 | 0.501471 | 1.640216 | 0.00611 | 0.09649 | 1 | 4352 | tags=38%, list=19%, signal=46% |
| GO_CYTOKINE_PRODUCTION_INVOLVED_IN_INFLAMMATORY_RESPONSE | 40 | 0.549462 | 1.58705 | 0.02459 | 0.116034 | 1 | 3153 | tags=35%, list=13%, signal=40% |
| GO_POSITIVE_REGULATION_OF_T_CELL_MEDIATED_IMMUNITY | 44 | 0.645123 | 1.844745 | 0.002053 | 0.058569 | 0.904 | 2645 | tags=41%, list=11%, signal=46% |

| | | | | | | | | |
|---|---|---|---|---|---|---|---|---|
| GO_REGULATION_OF_VIRAL_LIFE_CYCLE | 132 | 0.434954 | 1.848315 | 0.002114 | 0.058365 | 0.901 | 3675 | tags=22%, list=16%, signal=26% |
| GO_LYMPHOCYTE_APOPTOTIC_PROCESS | 71 | 0.567368 | 1.95873 | 0 | 0.085155 | 0.636 | 3475 | tags=30%, list=15%, signal=35% |
| GO_PURINERGIC_RECEPTOR_ACTIVITY | 23 | 0.645153 | 1.559038 | 0.044898 | 0.126636 | 1 | 3113 | tags=57%, list=13%, signal=65% |
| GO_POSITIVE_REGULATION_OF_INTERLEUKIN_10_PRODUCTION | 32 | 0.721205 | 1.899607 | 0.002004 | 0.064064 | 0.803 | 1948 | tags=44%, list=8%, signal=48% |
| GO_ACUTE_INFLAMMATORY_RESPONSE_TO_ANTIGENIC_STIMULUS | 19 | 0.739943 | 1.674321 | 0.00998 | 0.086229 | 1 | 2022 | tags=47%, list=9%, signal=52% |
| GO_POSITIVE_REGULATION_OF_RELEASE_OF_SEQUESTERED_CALCIUM_ION_INTO_CYTOSOL | 37 | 0.623136 | 1.705743 | 0 | 0.077818 | 0.999 | 4341 | tags=54%, list=18%, signal=66% |
| GO_POSITIVE_REGULATION_OF_MONOCYTE_CHEMOTAXIS | 18 | 0.767392 | 1.702185 | 0 | 0.078134 | 0.999 | 1859 | tags=56%, list=8%, signal=60% |
| GO_NEUTROPHIL_MIGRATION | 107 | 0.687986 | 1.846637 | 0 | 0.058831 | 0.902 | 2397 | tags=48%, list=10%, signal=53% |
| GO_POSITIVE_REGULATION_OF_JNK_CASCADE | 131 | 0.434873 | 1.688553 | 0.007905 | 0.081083 | 0.999 | 2678 | tags=21%, list=11%, signal=24% |
| GO_REGULATION_OF_MONOCYTE_CHEMOTAXIS | 24 | 0.764869 | 1.738063 | 0.002146 | 0.070701 | 0.992 | 1859 | tags=54%, list=8%, signal=59% |
| GO_TYROSINE_PHOSPHORYLATION_OF_STAT_PROTEIN | 81 | 0.488352 | 1.628887 | 0.010616 | 0.101189 | 1 | 5911 | tags=54%, list=25%, signal=72% |

| | | | | | | | | |
|---|---|---|---|---|---|---|---|---|
| GO_MONONUCLEAR_CELL_MIGRATION | 77 | 0.696672 | 1.786079 | 0.0020096 | 0.065864 | 0.969 | 2876 | tags=52%, list=12%, signal=59% |
| GO_NEGATIVE_REGULATION_OF_IMMUNE_RESPONSE | 140 | 0.577044 | 1.930486 | 0 | 0.072732 | 0.719 | 3908 | tags=39%, list=17%, signal=47% |
| GO_NEGATIVE_REGULATION_OF_ADAPTIVE_IMMUNE_RESPONSE | 43 | 0.570313 | 1.650956 | 0.0164461 | 0.093765 | 1 | 3714 | tags=42%, list=16%, signal=50% |
| GO_NEUROINFLAMMATORY_RESPONSE | 61 | 0.673164 | 1.79649 | 0 | 0.064531 | 0.951 | 4511 | tags=61%, list=19%, signal=75% |
| GO_HOMEOSTASIS_OF_NUMBER_OF_CELLS_WITHIN_A_TISSUE | 29 | 0.584606 | 1.760322 | 0.0020333 | 0.06874 | 0.982 | 3305 | tags=38%, list=14%, signal=44% |
| GO_REGULATION_OF_CALCIUM_ION_TRANSPORT | 232 | 0.477143 | 1.714267 | 0.0021466 | 0.076114 | 0.998 | 5137 | tags=46%, list=22%, signal=58% |
| GO_NEGATIVE_REGULATION_OF_LEUKOCYTE_MEDIATED_CYTOTOXICITY | 15 | 0.72288 | 1.632238 | 0.0225587 | 0.099804 | 1 | 3256 | tags=53%, list=14%, signal=62% |
| GO_DEFENSE_RESPONSE_TO_GRAM_POSITIVE_BACTERIUM | 69 | 0.540607 | 1.608544 | 0.0124744 | 0.107725 | 1 | 2917 | tags=33%, list=12%, signal=38% |
| GO_MONOCYTE_CHEMOTAXIS | 55 | 0.729532 | 1.759362 | 0.0021011 | 0.068552 | 0.984 | 1876 | tags=49%, list=8%, signal=53% |
| GO_NEGATIVE_REGULATION_OF_CELL_CELL_ADHESION | 163 | 0.521923 | 1.888374 | 0.00207 | 0.062212 | 0.832 | 4835 | tags=39%, list=21%, signal=48% |

| | | | | | | | | |
|---|---|---|---|---|---|---|---|---|
| GO_NEGATIVE_REGULATION_OF_INTERLEUKIN_2_PRODUCTION | 23 | 0.712968 | 1.72547 | 0.0060048 | 0.073208 | 0.994 | 2051 | tags=43%, list=9%, signal=48% |
| GO_POSITIVE_REGULATION_OF_MONONUCLEAR_CELL_MIGRATION | 23 | 0.743045 | 1.731539 | 0 | 0.072481 | 0.992 | 1859 | tags=52%, list=8%, signal=57% |
| GO_POSITIVE_REGULATION_OF_RECEPTOR_SIGNALING_PATHWAY_VIA_STAT | 88 | 0.498726 | 1.660154 | 0.0082999 | 0.090536 | 1 | 3953 | tags=42%, list=17%, signal=50% |
| GO_REGULATION_OF_CHEMOTAXIS | 199 | 0.566801 | 1.858888 | 0.0041558 | 0.058108 | 0.885 | 2722 | tags=33%, list=12%, signal=37% |
| GO_NEGATIVE_REGULATION_OF_LEUKOCYTE_MEDIATED_IMMUNITY | 46 | 0.650234 | 1.764497 | 0.0081663 | 0.068728 | 0.981 | 3714 | tags=48%, list=16%, signal=57% |
| GO_POSITIVE_REGULATION_OF_CALCIUM_ION_TRANSPORT | 115 | 0.508148 | 1.728645 | 0.0020008 | 0.07252 | 0.993 | 4341 | tags=43%, list=18%, signal=53% |
| GO_ACTIVATION_OF_PHOSPHOLIPASE_C_ACTIVITY | 30 | 0.587423 | 1.604732 | 0.0143344 | 0.109044 | 1 | 1491 | tags=37%, list=6%, signal=39% |
| GO_NEGATIVE_REGULATION_OF_HEMOPOIESIS | 130 | 0.453729 | 1.749352 | 0.0041558 | 0.068626 | 0.989 | 4799 | tags=34%, list=20%, signal=42% |
| GO_NEGATIVE_REGULATION_OF_LEUKOCYTE_DIFFERENTIATION | 94 | 0.504537 | 1.779453 | 0.0020066 | 0.067714 | 0.974 | 4799 | tags=39%, list=20%, signal=49% |

| | | | | | | | | |
|---|---|---|---|---|---|---|---|---|
| GO_VITAMIN_D_METABOLIC_PROCESS | 22 | 0.575765 | 1.549092 | 0.030181 | 0.130594 | 1 | 4511 | tags=41%, list=19%, signal=51% |

**EBV(+) vs. EBV(-) gastric cancer tissues (GSE51575)**

| NAME | SIZE | ES | NES | NOM.p.val | FDR.q.val | FWER.p.val | RANK.AT.MAX | LEADING.EDGE |
|---|---|---|---|---|---|---|---|---|
| GO_DOUBLE_STRANDED_RNA_BINDING | 67 | 0.532143 | 2.07555 | 0 | 0.118608 | 0.075 | 1335 | tags=21%, list=6%, signal=22% |
| GO_T_CELL_RECEPTOR_SIGNALING_PATHWAY | 171 | 0.6057 | 2.068084 | 0 | 0.066787 | 0.081 | 2254 | tags=27%, list=10%, signal=30% |
| GO_ANTIGEN_RECEPTOR_MEDIATED_SIGNALING_PATHWAY | 212 | 0.655218 | 2.016444 | 0 | 0.09044 | 0.141 | 2339 | tags=35%, list=11%, signal=39% |
| GO_NEGATIVE_REGULATION_OF_TYPE_I_INTERFERON_PRODUCTION | 41 | 0.662911 | 1.975701 | 0 | 0.12449 | 0.229 | 2596 | tags=41%, list=12%, signal=47% |
| GO_IMMUNE_RESPONSE_REGULATING_CELL_SURFACE_RECEPTOR_SIGNALING_PATHWAY | 349 | 0.609736 | 1.974831 | 0 | 0.101337 | 0.233 | 2937 | tags=36%, list=14%, signal=41% |
| GO_FC_RECEPTOR_SIGNALING_PATHWAY | 172 | 0.478577 | 1.944169 | 0.006073 | 0.092807 | 0.308 | 2075 | tags=20%, list=10%, signal=22% |
| GO_NEGATIVE_REGULATION_OF_ANTIGEN_RECEPTOR_MEDIATED_SIGNALING_PATHWAY | 27 | 0.812242 | 1.929389 | 0 | 0.091005 | 0.357 | 2239 | tags=56%, list=10%, signal=62% |
| GO_REGULATION_OF_DEFENSE_RESPONSE_TO_VIRUS | 65 | 0.623399 | 1.916685 | 0.00625 | 0.098472 | 0.398 | 3521 | tags=42%, list=16%, signal=49% |
| GO_ALPHA_BETA_T_CELL_DIFFERENTIATION | 97 | 0.699449 | 1.912125 | 0 | 0.095753 | 0.406 | 2847 | tags=49%, list=13%, signal=57% |
| GO_REGULATION_OF_T_CELL_RECEPTOR_SIGNALING_PATHWAY | 36 | 0.769538 | 1.907191 | 0 | 0.095157 | 0.425 | 2159 | tags=42%, list=10%, signal=46% |
| GO_T_HELPER_1_TYPE_IMMUNE_RESPONSE | 41 | 0.771303 | 1.9044 | 0 | 0.091853 | 0.435 | 3008 | tags=61%, list=14%, signal=71% |

| | | | | | | | | |
|---|---|---|---|---|---|---|---|---|
| GO_NEGATIVE_REGULATION_OF_T_CELL_RECEPTOR_SIGNALING_PATHWAY | 20 | 0.809517 | 1.89716 | 0 | 0.095051 | 0.457 | 1886 | tags=45%, list=9%, signal=49% |
| GO_T_CELL_ACTIVATION_INVOLVED_IN_IMMUNE_RESPONSE | 93 | 0.689298 | 1.89236 | 0 | 0.096687 | 0.481 | 3170 | tags=49%, list=15%, signal=58% |
| GO_RESPONSE_TO_TYPE_I_INTERFERON | 83 | 0.656337 | 1.89105 | 0.007921 | 0.092699 | 0.484 | 2349 | tags=41%, list=11%, signal=46% |
| GO_POSITIVE_REGULATION_OF_ALPHA_BETA_T_CELL_ACTIVATION | 58 | 0.735926 | 1.889221 | 0 | 0.088889 | 0.487 | 2740 | tags=55%, list=13%, signal=63% |
| GO_ALPHA_BETA_T_CELL_ACTIVATION | 129 | 0.696362 | 1.888638 | 0 | 0.084711 | 0.49 | 2847 | tags=52%, list=13%, signal=59% |
| GO_POSITIVE_REGULATION_OF_LEUKOCYTE_DIFFERENTIATION | 139 | 0.646592 | 1.882527 | 0 | 0.083264 | 0.512 | 2984 | tags=44%, list=14%, signal=51% |
| GO_NATURAL_KILLER_CELL_ACTIVATION | 70 | 0.64797 | 1.880601 | 0.002028 | 0.081306 | 0.517 | 1540 | tags=40%, list=7%, signal=43% |
| GO_POSITIVE_REGULATION_OF_ALPHA_BETA_T_CELL_DIFFERENTIATION | 45 | 0.700164 | 1.879509 | 0 | 0.078576 | 0.52 | 2740 | tags=51%, list=13%, signal=58% |
| GO_NON_MEMBRANE_SPANNING_PROTEIN_TYROSINE_KINASE_ACTIVITY | 44 | 0.664984 | 1.875492 | 0 | 0.079667 | 0.528 | 3595 | tags=50%, list=17%, signal=60% |
| GO_CD4_POSITIVE_ALPHA_BETA_T_CELL_DIFFERENTIATION | 71 | 0.703661 | 1.875399 | 0 | 0.076512 | 0.528 | 3170 | tags=54%, list=15%, signal=62% |
| GO_LYMPHOCYTE_ACTIVATION_INVOLVED_IN_IMMUNE_RESPONSE | 161 | 0.638088 | 1.875173 | 0.002045 | 0.073817 | 0.531 | 3008 | tags=43%, list=14%, signal=50% |
| GO_POSITIVE_REGULATION_OF_CELL_ACTIVATION | 295 | 0.641128 | 1.871428 | 0.001976 | 0.074893 | 0.542 | 2944 | tags=46%, list=14%, signal=53% |
| GO_T_CELL_ACTIVATION | 428 | 0.610333 | 1.861958 | 0 | 0.081683 | 0.574 | 3064 | tags=44%, list=14%, signal=50% |
| GO_REGULATION_OF_ANTIGEN_RECEPTOR_MEDIATED_SIGNALING_PATHWAY | 57 | 0.76958 | 1.859198 | 0 | 0.082267 | 0.587 | 2339 | tags=53%, list=11%, signal=59% |

| | | | | | | | | |
|---|---|---|---|---|---|---|---|---|
| GO_POSITIVE_REGULATION_OF_LYMPHOCYTE_DIFFERENTIATION | 90 | 0.697315 | 1.852445 | 0 | 0.087073 | 0.607 | 2740 | tags=50%, list=13%, signal=57% |
| GO_LYMPHOCYTE_DIFFERENTIATION | 330 | 0.596995 | 1.844472 | 0.004 | 0.091501 | 0.634 | 2948 | tags=41%, list=14%, signal=47% |
| GO_DEFENSE_RESPONSE_TO_VIRUS | 216 | 0.587663 | 1.842569 | 0.004158 | 0.091415 | 0.643 | 3204 | tags=41%, list=15%, signal=47% |
| GO_REGULATION_OF_LEUKOCYTE_DEGRANULATION | 41 | 0.658022 | 1.842412 | 0.002049 | 0.088914 | 0.644 | 2339 | tags=44%, list=11%, signal=49% |
| GO_POSITIVE_REGULATION_OF_INTERFERON_ALPHA_PRODUCTION | 21 | 0.712938 | 1.839819 | 0.004115 | 0.089487 | 0.652 | 1198 | tags=33%, list=6%, signal=35% |
| GO_RESPONSE_TO_VIRUS | 293 | 0.552695 | 1.839111 | 0.002045 | 0.087719 | 0.654 | 3303 | tags=38%, list=15%, signal=44% |
| GO_REGULATION_OF_ALPHA_BETA_T_CELL_DIFFERENTIATION | 60 | 0.696276 | 1.83504 | 0 | 0.089871 | 0.666 | 3008 | tags=50%, list=14%, signal=58% |
| GO_REGULATION_OF_DEFENSE_RESPONSE_TO_VIRUS_BY_VIRUS | 30 | 0.644294 | 1.834945 | 0.008147 | 0.087611 | 0.667 | 2698 | tags=33%, list=12%, signal=38% |
| GO_CD4_POSITIVE_ALPHA_BETA_T_CELL_ACTIVATION | 86 | 0.667926 | 1.834328 | 0 | 0.084143 | 0.668 | 3170 | tags=52%, list=15%, signal=61% |
| GO_REGULATION_OF_NATURAL_KILLER_CELL_ACTIVATION | 32 | 0.713663 | 1.830459 | 0 | 0.083872 | 0.681 | 2741 | tags=50%, list=13%, signal=57% |
| GO_POSITIVE_REGULATION_OF_LEUKOCYTE_CELL_CELL_ADHESION | 202 | 0.667313 | 1.829509 | 0.002004 | 0.083033 | 0.684 | 2984 | tags=50%, list=14%, signal=57% |

| | | | | | | | | |
|---|---|---|---|---|---|---|---|---|
| GO_INTERFERON_GAMMA_PRODUCTION | 102 | 0.701112 | 1.829297 | 0.0020004 | 0.081587 | 0.686 | 3172 | tags=58%, list=15%, signal=67% |
| GO_ACTIVATED_T_CELL_PROLIFERATION | 39 | 0.638953 | 1.82811 | 0 | 0.079241 | 0.687 | 3172 | tags=46%, list=15%, signal=54% |
| GO_CELLULAR_RESPONSE_TO_INTERFERON_BETA | 19 | 0.714158 | 1.828074 | 0.0062111 | 0.077604 | 0.687 | 990 | tags=37%, list=5%, signal=39% |
| GO_ACTIVATION_OF_INNATE_IMMUNE_RESPONSE | 288 | 0.492785 | 1.827737 | 0.0041841 | 0.076396 | 0.687 | 3283 | tags=31%, list=15%, signal=36% |
| GO_MYD88_INDEPENDENT_TOLL_LIKE_RECEPTOR_SIGNALING_PATHWAY | 32 | 0.601914 | 1.826671 | 0.0123711 | 0.075901 | 0.691 | 2251 | tags=25%, list=10%, signal=28% |
| GO_INTERFERON_ALPHA_PRODUCTION | 29 | 0.714405 | 1.822396 | 0.0041149 | 0.073613 | 0.699 | 1198 | tags=34%, list=6%, signal=36% |
| GO_T_CELL_DIFFERENTIATION | 226 | 0.619099 | 1.816524 | 0.0083333 | 0.07805 | 0.715 | 3008 | tags=45%, list=14%, signal=51% |
| GO_POSITIVE_REGULATION_OF_INTERFERON_BETA_PRODUCTION | 30 | 0.587582 | 1.814785 | 0.0020058 | 0.078551 | 0.722 | 1198 | tags=23%, list=6%, signal=25% |
| GO_T_CELL_PROLIFERATION | 169 | 0.605301 | 1.813714 | 0 | 0.076811 | 0.725 | 3172 | tags=47%, list=15%, signal=54% |
| GO_TUMOR_NECROSIS_FACTOR_BIOSYNTHETIC_PROCESS | 30 | 0.743548 | 1.813475 | 0.0060012 | 0.075804 | 0.727 | 2639 | tags=57%, list=12%, signal=64% |

| | | | | | | | | |
|---|---|---|---|---|---|---|---|---|
| GO_RESPONSE_TO_INTERFERON_ALPHA | 20 | 0.802559 | 1.813431 | 0 | 0.074591 | 0.727 | 2958 | tags=50%, list=14%, signal=58% |
| GO_REGULATION_OF_ALPHA_BETA_T_CELL_ACTIVATION | 85 | 0.70215 | 1.812802 | 0 | 0.074049 | 0.731 | 2740 | tags=53%, list=13%, signal=60% |
| GO_POSITIVE_REGULATION_OF_RESPONSE_TO_BIOTIC_STIMULUS | 357 | 0.519389 | 1.811001 | 0.00207 | 0.074529 | 0.736 | 2855 | tags=31%, list=13%, signal=35% |
| GO_T_CELL_DIFFERENTIATION_INVOLVED_IN_IMMUNE_RESPONSE | 65 | 0.696255 | 1.810075 | 0 | 0.074065 | 0.738 | 3170 | tags=52%, list=15%, signal=61% |
| GO_LEUKOCYTE_DIFFERENTIATION | 483 | 0.549542 | 1.809998 | 0.004032 | 0.072967 | 0.738 | 3008 | tags=37%, list=14%, signal=42% |
| GO_REGULATION_OF_T_HELPER_1_TYPE_IMMUNE_RESPONSE | 26 | 0.784888 | 1.806428 | 0 | 0.075277 | 0.746 | 3008 | tags=69%, list=14%, signal=80% |
| GO_REGULATION_OF_OSTEOCLAST_DIFFERENTIATION | 60 | 0.622047 | 1.806098 | 0.003984 | 0.074388 | 0.746 | 3112 | tags=43%, list=14%, signal=50% |
| GO_POSITIVE_REGULATION_OF_HEMOPOIESIS | 174 | 0.56977 | 1.806073 | 0.004115 | 0.073287 | 0.746 | 2984 | tags=39%, list=14%, signal=44% |
| GO_POSITIVE_REGULATION_OF_T_HELPER_1_TYPE_IMMUNE_RESPONSE | 19 | 0.802101 | 1.805928 | 0.002083 | 0.072315 | 0.746 | 2740 | tags=68%, list=13%, signal=78% |
| GO_REGULATION_OF_MYELOID_LEUKOCYTE_DIFFERENTIATION | 110 | 0.551528 | 1.805397 | 0.002049 | 0.071775 | 0.747 | 4014 | tags=43%, list=18%, signal=52% |
| GO_ADAPTIVE_IMMUNE_RESPONSE | 360 | 0.669405 | 1.804561 | 0 | 0.071565 | 0.749 | 3008 | tags=52%, list=14%, signal=60% |

| | | | | | | | |
|---|---|---|---|---|---|---|---|
| GO_REGULATION_OF_DEFENSE_RESPONSE_TO_VIRUS_BY_HOST | 33 | 0.681596 | 1.802333 | 0.008264 | 0.072543 | 0.756 | 3172 | tags=48%, list=15%, signal=57% |
| GO_PHAGOCYTOSIS | 244 | 0.530821 | 1.8009 | 0.006061 | 0.073 | 0.758 | 3172 | tags=37%, list=15%, signal=43% |
| GO_REGULATION_OF_LEUKOCYTE_DIFFERENTIATION | 256 | 0.572929 | 1.800725 | 0.00611 | 0.072157 | 0.758 | 3008 | tags=39%, list=14%, signal=45% |
| GO_TUMOR_NECROSIS_FACTOR_SUPERFAMILY_CYTOKINE_PRODUCTION | 144 | 0.647568 | 1.800362 | 0.002092 | 0.071484 | 0.76 | 2740 | tags=44%, list=13%, signal=51% |
| GO_REGULATION_OF_RESPONSE_TO_BIOTIC_STIMULUS | 472 | 0.524319 | 1.799777 | 0.002079 | 0.071097 | 0.762 | 3008 | tags=33%, list=14%, signal=37% |
| GO_ANTIGEN_PROCESSING_AND_PRESENTATION | 200 | 0.497105 | 1.796585 | 0.008247 | 0.073207 | 0.769 | 2790 | tags=24%, list=13%, signal=27% |
| GO_ADAPTIVE_IMMUNE_RESPONSE_BASED_ON_SOMATIC_RECOMBINATION_OF_IMMUNE_RECEPTORS_BUILT_FROM_IMMUNOGLOBULIN_SUPERFAMILY_DOMAINS | 232 | 0.629235 | 1.792233 | 0.002041 | 0.075187 | 0.78 | 3008 | tags=47%, list=14%, signal=54% |
| GO_REGULATION_OF_T_CELL_ACTIVATION | 292 | 0.611557 | 1.787092 | 0.004049 | 0.077396 | 0.794 | 3064 | tags=46%, list=14%, signal=53% |
| GO_POSITIVE_REGULATION_OF_CYTOKINE_PRODUCTION | 425 | 0.562649 | 1.784505 | 0.004107 | 0.078934 | 0.798 | 3213 | tags=40%, list=15%, signal=45% |

| | | | | | | | | |
|---|---|---|---|---|---|---|---|---|
| GO_REGULATION_OF_HEMOPOIESIS | 411 | 0.493657 | 1.78205 | 0.0061733 | 0.079482 | 0.806 | 3008 | tags=30%, list=14%, signal=35% |
| GO_POSITIVE_REGULATION_OF_CELL_CELL_ADHESION | 236 | 0.619326 | 1.781671 | 0.0061355 | 0.078922 | 0.807 | 3172 | tags=47%, list=15%, signal=54% |
| GO_POSITIVE_REGULATION_OF_IMMUNE_EFFECTOR_PROCESS | 191 | 0.607795 | 1.778337 | 0.0041558 | 0.081026 | 0.812 | 2400 | tags=39%, list=11%, signal=43% |
| GO_POSITIVE_REGULATION_OF_INTERFERON_GAMMA_PRODUCTION | 59 | 0.735751 | 1.777663 | 0.0020204 | 0.080728 | 0.814 | 3172 | tags=63%, list=15%, signal=73% |
| GO_POSITIVE_REGULATION_OF_DEFENSE_RESPONSE_TO_VIRUS_BY_HOST | 23 | 0.72683 | 1.775511 | 0.006 | 0.080614 | 0.815 | 3172 | tags=61%, list=15%, signal=71% |
| GO_IMMUNOLOGICAL_SYNAPSE | 34 | 0.753183 | 1.768777 | 0.0059760 | 0.084469 | 0.826 | 1041 | tags=41%, list=5%, signal=43% |
| GO_POSITIVE_REGULATION_OF_MULTI_ORGANISM_PROCESS | 486 | 0.461857 | 1.765812 | 0.0041755 | 0.08551 | 0.827 | 3896 | tags=32%, list=18%, signal=38% |
| GO_T_CELL_MEDIATED_IMMUNITY | 90 | 0.634361 | 1.763922 | 0.0103733 | 0.085024 | 0.833 | 2980 | tags=44%, list=14%, signal=51% |
| GO_REGULATION_OF_NATURAL_KILLER_CELL_MEDIATED_IMMUNITY | 37 | 0.786649 | 1.763025 | 0.00198 | 0.084973 | 0.833 | 1936 | tags=59%, list=9%, signal=65% |

| | | | | | | | | |
|---|---|---|---|---|---|---|---|---|
| GO_REGULATION_OF_MACROPHAGE_MIGRATION | 35 | 0.678628 | 1.762488 | 0.002088 | 0.083866 | 0.833 | 2944 | tags=54%, list=14%, signal=63% |
| GO_LEUKOCYTE_CELL_CELL_ADHESION | 311 | 0.607091 | 1.760927 | 0.004032 | 0.084754 | 0.837 | 3172 | tags=47%, list=15%, signal=54% |
| GO_CYTOKINE_SECRETION | 213 | 0.618408 | 1.760452 | 0.004115 | 0.08435 | 0.837 | 3176 | tags=47%, list=15%, signal=55% |
| GO_LYMPHOCYTE_MEDIATED_IMMUNITY | 226 | 0.623315 | 1.75393 | 0.002053 | 0.089076 | 0.849 | 2464 | tags=44%, list=11%, signal=49% |
| GO_RESPONSE_TO_INTERFERON_GAMMA | 176 | 0.603542 | 1.753501 | 0.008475 | 0.088598 | 0.849 | 2439 | tags=38%, list=11%, signal=43% |
| GO_CD8_POSITIVE_ALPHA_BETA_T_CELL_ACTIVATION | 21 | 0.774716 | 1.752461 | 0.00207 | 0.08813 | 0.851 | 2188 | tags=62%, list=10%, signal=69% |
| GO_SYNCYTIUM_FORMATION | 48 | 0.604808 | 1.752112 | 0.002033 | 0.087622 | 0.852 | 3519 | tags=35%, list=16%, signal=42% |
| GO_REGULATION_OF_IMMUNE_EFFECTOR_PROCESS | 357 | 0.5728 | 1.75154 | 0.006186 | 0.086617 | 0.853 | 3008 | tags=41%, list=14%, signal=47% |
| GO_MYOBLAST_FUSION | 36 | 0.624999 | 1.75114 | 0 | 0.086252 | 0.853 | 3519 | tags=39%, list=16%, signal=46% |

| | | | | | | | | |
|---|---|---|---|---|---|---|---|---|
| GO_REGULATION_OF_LYMPHOCYTE_ACTIVATION | 391 | 0.58108 | 1.749263 | 0.0101633 | 0.087223 | 0.855 | 2944 | tags=42%, list=14%, signal=48% |
| GO_LEUKOCYTE_PROLIFERATION | 269 | 0.582168 | 1.749237 | 0.0060733 | 0.086494 | 0.855 | 3172 | tags=45%, list=15%, signal=52% |
| GO_PRODUCTION_OF_MOLECULAR_MEDIATOR_OF_IMMUNE_RESPONSE | 188 | 0.578198 | 1.748427 | 0.01006 | 0.086537 | 0.856 | 2711 | tags=37%, list=12%, signal=42% |
| GO_POSITIVE_REGULATION_OF_T_CELL_PROLIFERATION | 85 | 0.677528 | 1.747109 | 0.0020249 | 0.08716 | 0.858 | 3172 | tags=55%, list=15%, signal=64% |
| GO_POSITIVE_REGULATION_OF_MYELOID_LEUKOCYTE_DIFFERENTIATION | 53 | 0.60329 | 1.746511 | 0.0106844 | 0.086975 | 0.858 | 4013 | tags=45%, list=18%, signal=55% |
| GO_POSITIVE_REGULATION_OF_TUMOR_NECROSIS_FACTOR_SUPERFAMILY_CYTOKINE_PRODUCTION | 82 | 0.64123 | 1.745379 | 0.0062889 | 0.087573 | 0.862 | 3406 | tags=49%, list=16%, signal=58% |
| GO_POSITIVE_REGULATION_OF_CD4_POSITIVE_ALPHA_BETA_T_CELL_ACTIVATION | 35 | 0.755068 | 1.744388 | 0.0020996 | 0.087781 | 0.862 | 2740 | tags=63%, list=13%, signal=72% |
| GO_REGULATION_OF_LEUKOCYTE_MEDIATED_CYTOTOXICITY | 65 | 0.712696 | 1.740307 | 0.0060036 | 0.090354 | 0.872 | 2790 | tags=57%, list=13%, signal=65% |
| GO_INTERLEUKIN_12_PRODUCTION | 54 | 0.696942 | 1.739938 | 0.0061733 | 0.090123 | 0.873 | 2944 | tags=56%, list=14%, signal=64% |

| | | | | | | | | |
|---|---|---|---|---|---|---|---|---|
| GO_REGULATION_OF_LEUKOCYTE_MIGRATION | 176 | 0.611181 | 1.739868 | 0.002053 | 0.089435 | 0.873 | 3234 | tags=50%, list=15%, signal=58% |
| GO_INTERLEUKIN_8_SECRETION | 28 | 0.701842 | 1.739168 | 0 | 0.088617 | 0.873 | 3489 | tags=61%, list=16%, signal=72% |
| GO_REGULATION_OF_LEUKOCYTE_MEDIATED_IMMUNITY | 180 | 0.611164 | 1.73909 | 0.004158 | 0.087972 | 0.873 | 2342 | tags=41%, list=11%, signal=46% |
| GO_T_CELL_MIGRATION | 61 | 0.706476 | 1.73721 | 0.010225 | 0.088358 | 0.873 | 3172 | tags=61%, list=15%, signal=71% |
| GO_REGULATION_OF_MYELOID_LEUKOCYTE_MEDIATED_IMMUNITY | 50 | 0.605102 | 1.735227 | 0.008299 | 0.088387 | 0.875 | 2339 | tags=42%, list=11%, signal=47% |
| GO_REGULATION_OF_RESPONSE_TO_CYTOKINE_STIMULUS | 162 | 0.513426 | 1.734185 | 0.014228 | 0.088667 | 0.875 | 3453 | tags=36%, list=16%, signal=42% |
| GO_LYMPHOCYTE_HOMEOSTASIS | 61 | 0.611723 | 1.732582 | 0.008403 | 0.089132 | 0.876 | 2339 | tags=38%, list=11%, signal=42% |
| GO_NEGATIVE_REGULATION_OF_CYTOKINE_PRODUCTION | 249 | 0.551638 | 1.73235 | 0.00611 | 0.088691 | 0.876 | 3172 | tags=41%, list=15%, signal=48% |
| GO_FC_RECEPTOR_MEDIATED_STIMULATORY_SIGNALING_PATHWAY | 80 | 0.490009 | 1.732124 | 0.022634 | 0.088293 | 0.876 | 1775 | tags=21%, list=8%, signal=23% |

| | | | | | | | | |
|---|---|---|---|---|---|---|---|---|
| GO_LYMPHOCYTE_MIGRATION | 101 | 0.699145 | 1.731363 | 0.008114 | 0.08845 | 0.878 | 2421 | tags=52%, list=11%, signal=59% |
| GO_REGULATION_OF_CD4_POSITIVE_ALPHA_BETA_T_CELL_DIFFERENTIATION | 45 | 0.720555 | 1.72789 | 0.004167 | 0.089381 | 0.882 | 3008 | tags=56%, list=14%, signal=64% |
| GO_LEUKOCYTE_MEDIATED_CYTOTOXICITY | 94 | 0.645264 | 1.726987 | 0.006098 | 0.089755 | 0.883 | 2188 | tags=49%, list=10%, signal=54% |
| GO_INTERLEUKIN_8_PRODUCTION | 75 | 0.620417 | 1.726975 | 0.002066 | 0.089129 | 0.883 | 3826 | tags=51%, list=18%, signal=61% |
| GO_ANTIGEN_PROCESSING_AND_PRESENTATION_OF_EXOGENOUS_PEPTIDE_ANTIGEN_VIA_MHC_CLASS_I | 70 | 0.537368 | 1.726889 | 0.016327 | 0.088533 | 0.883 | 2555 | tags=26%, list=12%, signal=29% |
| GO_REGULATION_OF_LYMPHOCYTE_DIFFERENTIATION | 160 | 0.599258 | 1.725773 | 0.010267 | 0.089109 | 0.886 | 3008 | tags=44%, list=14%, signal=51% |
| GO_POSITIVE_REGULATION_OF_LEUKOCYTE_PROLIFERATION | 125 | 0.620684 | 1.724812 | 0.006211 | 0.089503 | 0.886 | 2740 | tags=46%, list=13%, signal=52% |
| GO_REGULATION_OF_T_CELL_DIFFERENTIATION | 129 | 0.622571 | 1.724585 | 0.010267 | 0.089104 | 0.886 | 2740 | tags=46%, list=13%, signal=52% |
| GO_POSITIVE_REGULATION_OF_CD4_POSITIVE_ALPHA_BETA_T_CELL_DIFFERENTIATION | 29 | 0.754175 | 1.724539 | 0.004149 | 0.088519 | 0.886 | 2740 | tags=62%, list=13%, signal=71% |

| | | | | | | | | |
|---|---|---|---|---|---|---|---|---|
| GO_REGULATION_OF_LYMPHOCYTE_MIGRATION | 55 | 0.655742 | 1.722753 | 0.0119 76 | 0.089694 | 0.887 | 3234 | tags=56%, list=15%, signal=66% |
| GO_T_CELL_SELECTION | 46 | 0.73819 | 1.722177 | 0.0058 94 | 0.089629 | 0.887 | 2254 | tags=57%, list=10%, signal=63% |
| GO_POSITIVE_REGULATION_OF_LEUKOCYTE_MIGRATION | 121 | 0.627913 | 1.720353 | 0.0020 75 | 0.089788 | 0.892 | 3222 | tags=53%, list=15%, signal=62% |
| GO_LIPOPOLYSACCHARIDE_MEDIATED_SIGNALING_PATHWAY | 55 | 0.627051 | 1.719611 | 0.0123 71 | 0.089964 | 0.893 | 3461 | tags=40%, list=16%, signal=47% |
| GO_NEGATIVE_REGULATION_OF_REGULATED_SECRETORY_PATHWAY | 20 | 0.71304 | 1.719377 | 0 | 0.089607 | 0.893 | 2065 | tags=45%, list=10%, signal=50% |
| GO_INTERLEUKIN_1_BETA_PRODUCTION | 83 | 0.664539 | 1.718675 | 0.0061 98 | 0.089809 | 0.893 | 2323 | tags=51%, list=11%, signal=56% |
| GO_REGULATION_OF_CELL_KILLING | 86 | 0.671233 | 1.716264 | 0.0061 73 | 0.09082 | 0.895 | 2790 | tags=51%, list=13%, signal=58% |
| GO_POSITIVE_REGULATION_OF_CYTOKINE_SECRETION | 130 | 0.595605 | 1.715394 | 0.0041 67 | 0.090609 | 0.898 | 3406 | tags=45%, list=16%, signal=53% |
| GO_NEGATIVE_REGULATION_OF_IMMUNE_SYSTEM_PROCESS | 408 | 0.528804 | 1.713604 | 0.0081 3 | 0.092048 | 0.9 | 3064 | tags=38%, list=14%, signal=43% |
| GO_REGULATION_OF_SYNCYTIUM_FORMATION_BY_PLASMA_MEMBRANE_FUSION | 26 | 0.738185 | 1.71264 | 0 | 0.092451 | 0.9 | 2895 | tags=50%, list=13%, signal=58% |

| | | | | | | | | |
|---|---|---|---|---|---|---|---|---|
| GO_NIK_NF_KAPPAB_SIGNALING | 154 | 0.455834 | 1.712259 | 0.0184443 | 0.092245 | 0.9 | 3130 | tags=22%, list=14%, signal=26% |
| GO_ANTIGEN_PROCESSING_AND_PRESENTATION_OF_PEPTIDE_ANTIGEN_VIA_MHC_CLASS_I | 91 | 0.493832 | 1.711387 | 0.0357789 | 0.092559 | 0.901 | 2555 | tags=22%, list=12%, signal=25% |
| GO_POSITIVE_REGULATION_OF_NATURAL_KILLER_CELL_MEDIATED_CYTOTOXICITY | 20 | 0.860364 | 1.710702 | 0.0019844 | 0.092073 | 0.902 | 1090 | tags=65%, list=5%, signal=68% |
| GO_INTERFERON_GAMMA_MEDIATED_SIGNALING_PATHWAY | 72 | 0.638614 | 1.704809 | 0.0103733 | 0.09329 | 0.911 | 2318 | tags=42%, list=11%, signal=46% |
| GO_POSITIVE_REGULATION_OF_CELL_ADHESION | 372 | 0.542566 | 1.702229 | 0.0103733 | 0.094989 | 0.916 | 3229 | tags=40%, list=15%, signal=47% |
| GO_POSITIVE_REGULATION_OF_TUMOR_NECROSIS_FACTOR_BIOSYNTHETIC_PROCESS | 20 | 0.731927 | 1.702123 | 0.0098433 | 0.09457 | 0.916 | 2639 | tags=55%, list=12%, signal=63% |
| GO_REGULATION_OF_MYELOID_CELL_DIFFERENTIATION | 199 | 0.462122 | 1.702103 | 0.0083333 | 0.094046 | 0.916 | 2984 | tags=29%, list=14%, signal=33% |
| GO_POSITIVE_REGULATION_OF_OSTEOCLAST_DIFFERENTIATION | 25 | 0.695878 | 1.70117 | 0.0142228 | 0.093891 | 0.916 | 1305 | tags=40%, list=6%, signal=43% |
| GO_I_KAPPAB_PHOSPHORYLATION | 18 | 0.714063 | 1.700445 | 0.0149899 | 0.094126 | 0.917 | 2175 | tags=33%, list=10%, signal=37% |

| | | | | | | | | |
|---|---|---|---|---|---|---|---|---|
| GO_POSITIVE_REGULATION_OF_NATURAL_KILLER_CELL_MEDIATED_IMMUNITY | 23 | 0.851879 | 1.700028 | 0.004 | 0.094052 | 0.917 | 1090 | tags=65%, list=5%, signal=69% |
| GO_PRODUCTION_OF_MOLECULAR_MEDIATOR_INVOLVED_IN_INFLAMMATORY_RESPONSE | 64 | 0.573841 | 1.697523 | 0.012048 | 0.095049 | 0.922 | 3826 | tags=45%, list=18%, signal=55% |
| GO_POSITIVE_REGULATION_OF_LEUKOCYTE_MEDIATED_CYTOTOXICITY | 43 | 0.753409 | 1.69715 | 0.008197 | 0.094929 | 0.922 | 1305 | tags=51%, list=6%, signal=54% |
| GO_REGULATION_OF_MAST_CELL_ACTIVATION | 36 | 0.603179 | 1.696913 | 0.01006 | 0.09465 | 0.922 | 3890 | tags=53%, list=18%, signal=64% |
| GO_REGULATION_OF_MACROPHAGE_CHEMOTAXIS | 23 | 0.635588 | 1.695188 | 0.016293 | 0.095355 | 0.922 | 2944 | tags=52%, list=14%, signal=60% |
| GO_INTERLEUKIN_1_PRODUCTION | 96 | 0.64295 | 1.694339 | 0.004202 | 0.095772 | 0.922 | 2464 | tags=47%, list=11%, signal=53% |
| GO_NEGATIVE_REGULATION_OF_EXOCYTOSIS | 28 | 0.639229 | 1.693805 | 0.003984 | 0.095902 | 0.922 | 3606 | tags=46%, list=17%, signal=56% |
| GO_MHC_PROTEIN_BINDING | 27 | 0.746001 | 1.691088 | 0.010395 | 0.097806 | 0.924 | 1387 | tags=52%, list=6%, signal=55% |
| GO_T_CELL_MEDIATED_CYTOTOXICITY | 35 | 0.696986 | 1.690623 | 0.019397 | 0.097708 | 0.924 | 2790 | tags=51%, list=13%, signal=59% |

| | | | | | | | | |
|---|---|---|---|---|---|---|---|---|
| GO_POSITIVE_REGULATION_OF_CYTOKINE_BIOSYNTHETIC_PROCESS | 67 | 0.65781 | 1.689663 | 0.0142228 | 0.096641 | 0.924 | 2254 | tags=46%, list=10%, signal=51% |
| GO_CELLULAR_DEFENSE_RESPONSE | 49 | 0.694775 | 1.688768 | 0.0060625 | 0.097088 | 0.924 | 2937 | tags=65%, list=14%, signal=75% |
| GO_REGULATION_OF_T_CELL_MIGRATION | 40 | 0.651461 | 1.688088 | 0.0083333 | 0.096827 | 0.925 | 3172 | tags=55%, list=15%, signal=64% |
| GO_TUMOR_NECROSIS_FACTOR_MEDIATED_SIGNALING_PATHWAY | 154 | 0.468989 | 1.687174 | 0.02714 | 0.096744 | 0.928 | 3489 | tags=29%, list=16%, signal=35% |
| GO_RESPIRATORY_BURST | 30 | 0.722644 | 1.686566 | 0.01222 | 0.09703 | 0.929 | 3461 | tags=50%, list=16%, signal=59% |
| GO_MYELOID_LEUKOCYTE_DIFFERENTIATION | 194 | 0.495534 | 1.686534 | 0.0102267 | 0.096597 | 0.929 | 2991 | tags=34%, list=14%, signal=39% |
| GO_DENDRITIC_CELL_DIFFERENTIATION | 37 | 0.680844 | 1.686317 | 0.0083316 | 0.096316 | 0.93 | 2613 | tags=54%, list=12%, signal=61% |
| GO_POSITIVE_REGULATION_OF_LYMPHOCYTE_MEDIATED_IMMUNITY | 90 | 0.662857 | 1.685799 | 0.0123977 | 0.096454 | 0.932 | 2790 | tags=47%, list=13%, signal=53% |
| GO_REGULATION_OF_T_HELPER_CELL_DIFFERENTIATION | 34 | 0.659059 | 1.682898 | 0.0104438 | 0.098181 | 0.935 | 3008 | tags=47%, list=14%, signal=55% |

| | | | | | | | | |
|---|---|---|---|---|---|---|---|---|
| GO_LEUKOCYTE_ACTIVATION_INVOLVED_IN_INFLAMMATORY_RESPONSE | 43 | 0.738744 | 1.681784 | 0.0060048 | 0.09886 | 0.936 | 1587 | tags=49%, list=7%, signal=53% |
| GO_TYPE_I_INTERFERON_PRODUCTION | 119 | 0.483092 | 1.680991 | 0.0346694 | 0.099197 | 0.936 | 4343 | tags=34%, list=20%, signal=43% |
| GO_INTERLEUKIN_2_PRODUCTION | 63 | 0.630422 | 1.680667 | 0.0040065 | 0.099052 | 0.936 | 2254 | tags=43%, list=10%, signal=48% |
| GO_REGULATION_OF_CELL_CELL_ADHESION | 367 | 0.545398 | 1.680177 | 0.0143374 | 0.099119 | 0.937 | 3196 | tags=41%, list=15%, signal=47% |
| GO_REGULATION_OF_CD4_POSITIVE_ALPHA_BETA_T_CELL_ACTIVATION | 56 | 0.684021 | 1.679877 | 0.0020083 | 0.099016 | 0.938 | 2740 | tags=54%, list=13%, signal=61% |
| GO_RESPONSE_TO_AMYLOID_BETA | 47 | 0.640562 | 1.678387 | 0.0080048 | 0.098721 | 0.94 | 3274 | tags=45%, list=15%, signal=52% |
| GO_INTERFERON_BETA_PRODUCTION | 48 | 0.509982 | 1.678049 | 0.0188688 | 0.098614 | 0.942 | 1198 | tags=19%, list=6%, signal=20% |
| GO_REGULATION_OF_LYMPHOCYTE_MEDIATED_IMMUNITY | 132 | 0.621522 | 1.678018 | 0.0104417 | 0.09819 | 0.942 | 2790 | tags=45%, list=13%, signal=51% |
| GO_RESPONSE_TO_INTERFERON_BETA | 27 | 0.665993 | 1.676567 | 0.0167736 | 0.098811 | 0.943 | 2175 | tags=41%, list=10%, signal=45% |

| | | | | | | | | |
|---|---|---|---|---|---|---|---|---|
| GO_CELL_KILLING | 148 | 0.574832 | 1.675126 | 0.006263 | 0.099385 | 0.944 | 2790 | tags=42%, list=13%, signal=48% |
| GO_MYD88_DEPENDENT_TOLL_LIKE_RECEPTOR_SIGNALING_PATHWAY | 32 | 0.719706 | 1.674273 | 0.016529 | 0.099877 | 0.945 | 2251 | tags=44%, list=10%, signal=49% |
| GO_POSITIVE_REGULATION_OF_DEFENSE_RESPONSE | 451 | 0.503873 | 1.673487 | 0.014831 | 0.099392 | 0.947 | 2984 | tags=32%, list=14%, signal=36% |
| GO_POSITIVE_REGULATION_OF_LEUKOCYTE_MEDIATED_IMMUNITY | 116 | 0.625753 | 1.673474 | 0.012632 | 0.098969 | 0.947 | 2342 | tags=39%, list=11%, signal=43% |
| GO_POSITIVE_REGULATION_OF_MACROPHAGE_ACTIVATION | 21 | 0.732414 | 1.67267 | 0.014257 | 0.09937 | 0.948 | 2639 | tags=57%, list=12%, signal=65% |
| GO_POSITIVE_REGULATION_OF_CELL_KILLING | 56 | 0.70643 | 1.672203 | 0.014737 | 0.099536 | 0.948 | 2790 | tags=55%, list=13%, signal=63% |
| GO_B_CELL_ACTIVATION_INVOLVED_IN_IMMUNE_RESPONSE | 68 | 0.602735 | 1.670883 | 0.024048 | 0.100002 | 0.949 | 2464 | tags=38%, list=11%, signal=43% |
| GO_LEUKOCYTE_HOMEOSTASIS | 81 | 0.584732 | 1.670614 | 0.026157 | 0.099842 | 0.95 | 2723 | tags=41%, list=13%, signal=46% |
| GO_INTERLEUKIN_10_PRODUCTION | 50 | 0.712045 | 1.669795 | 0.008163 | 0.09989 | 0.952 | 3406 | tags=62%, list=16%, signal=73% |

| | | | | | | | | |
|---|---|---|---|---|---|---|---|---|
| GO_FICOLIN_1_RICH_GRANULE_MEMBRANE | 56 | 0.560816 | 1.668633 | 0.0026263 | 0.10033 | 0.953 | 2460 | tags=32%, list=11%, signal=36% |
| GO_REGULATION_OF_MYOBLAST_FUSION | 20 | 0.741889 | 1.666886 | 0.0012146 | 0.101195 | 0.955 | 2895 | tags=50%, list=13%, signal=58% |
| GO_LEUKOCYTE_MIGRATION | 402 | 0.560099 | 1.666192 | 0.0006224 | 0.100991 | 0.955 | 2780 | tags=40%, list=13%, signal=45% |
| GO_LEUKOCYTE_CHEMOTAXIS | 194 | 0.607626 | 1.664689 | 0.0008163 | 0.100864 | 0.957 | 2944 | tags=47%, list=14%, signal=54% |
| GO_PHAGOCYTIC_CUP | 24 | 0.642135 | 1.66283 | 0.022 | 0.101999 | 0.958 | 2984 | tags=50%, list=14%, signal=58% |
| GO_POSITIVE_REGULATION_OF_INTERLEUKIN_12_PRODUCTION | 33 | 0.697868 | 1.660289 | 0.0006122 | 0.104317 | 0.961 | 2944 | tags=58%, list=14%, signal=66% |
| GO_REGULATION_OF_LEUKOCYTE_PROLIFERATION | 203 | 0.56483 | 1.659729 | 0.0020367 | 0.104418 | 0.961 | 3172 | tags=46%, list=15%, signal=54% |
| GO_VIRAL_GENOME_REPLICATION | 113 | 0.476013 | 1.659588 | 0.0038934 | 0.104193 | 0.961 | 3204 | tags=29%, list=15%, signal=34% |
| GO_POSITIVE_REGULATION_OF_NF_KAPPAB_TRANSCRIPTION_FACTOR_ACTIVITY | 144 | 0.495156 | 1.65856 | 0.0016667 | 0.103632 | 0.961 | 4369 | tags=35%, list=20%, signal=43% |

| | | | | | | | | |
|---|---|---|---|---|---|---|---|---|
| GO_MACROPHAGE_ACTIVATION | 87 | 0.627719 | 1.658264 | 0.01227 | 0.103515 | 0.961 | 3418 | tags=51%, list=16%, signal=60% |
| GO_OSTEOCLAST_DIFFERENTIATION | 91 | 0.513336 | 1.655173 | 0.012448 | 0.105057 | 0.962 | 4164 | tags=42%, list=19%, signal=51% |
| GO_NITRIC_OXIDE_SYNTHASE_BIOSYNTHETIC_PROCESS | 18 | 0.738579 | 1.654954 | 0.008197 | 0.104464 | 0.962 | 1147 | tags=39%, list=5%, signal=41% |
| GO_POSITIVE_REGULATION_OF_T_CELL_MIGRATION | 29 | 0.685262 | 1.653475 | 0.014257 | 0.105532 | 0.965 | 3172 | tags=62%, list=15%, signal=73% |
| GO_NEGATIVE_REGULATION_OF_INTERLEUKIN_10_PRODUCTION | 16 | 0.807768 | 1.653083 | 0.002101 | 0.104821 | 0.965 | 2921 | tags=75%, list=13%, signal=87% |
| GO_B_CELL_PROLIFERATION | 84 | 0.635145 | 1.653062 | 0.013834 | 0.104446 | 0.965 | 2464 | tags=45%, list=11%, signal=51% |
| GO_REGULATION_OF_B_CELL_RECEPTOR_SIGNALING_PATHWAY | 24 | 0.770522 | 1.652408 | 0.004057 | 0.104768 | 0.967 | 2339 | tags=67%, list=11%, signal=75% |
| GO_T_CELL_CYTOKINE_PRODUCTION | 40 | 0.636765 | 1.652314 | 0.01232 | 0.104483 | 0.967 | 2323 | tags=38%, list=11%, signal=42% |
| GO_ANTIGEN_BINDING | 35 | 0.669544 | 1.651225 | 0.016842 | 0.104776 | 0.967 | 2790 | tags=57%, list=13%, signal=65% |

| | | | | | | | | |
|---|---|---|---|---|---|---|---|---|
| GO_NATURAL_KILLER_CELL_MEDIATED_IMMUNITY | 54 | 0.704037 | 1.650 93 | 0.0 14 08 5 | 0.104337 | 0.968 | 1936 | tags=56%, list=9%, signal=61% |
| GO_REGULATION_OF_ADAPTIVE_IMMUNE_RESPONSE | 146 | 0.587119 | 1.650 86 | 0.0 16 73 6 | 0.10405 | 0.968 | 3008 | tags=42%, list=14%, signal=49% |
| GO_POSITIVE_REGULATION_OF_ADAPTIVE_IMMUNE_RE SPONSE | 93 | 0.622303 | 1.650 367 | 0.0 19 02 7 | 0.104151 | 0.968 | 2790 | tags=41%, list=13%, signal=47% |
| GO_REGULATION_OF_INTERLEUKIN_4_PRODUCTION | 27 | 0.707242 | 1.646 731 | 0.0 14 19 9 | 0.107134 | 0.969 | 2875 | tags=63%, list=13%, signal=72% |
| GO_POSITIVE_REGULATION_OF_MACROPHAGE_MIGRATI ON | 20 | 0.747323 | 1.645 851 | 0.0 18 75 | 0.107631 | 0.97 | 2944 | tags=60%, list=14%, signal=69% |
| GO_REGULATION_OF_T_CELL_MEDIATED_IMMUNITY | 63 | 0.626303 | 1.645 507 | 0.0 20 79 | 0.107633 | 0.97 | 2790 | tags=43%, list=13%, signal=49% |
| GO_INTERLEUKIN_6_PRODUCTION | 134 | 0.571424 | 1.645 207 | 0.0 25 21 | 0.10762 | 0.97 | 3418 | tags=46%, list=16%, signal=55% |
| GO_NEGATIVE_REGULATION_OF_INNATE_IMMUNE_RESP ONSE | 52 | 0.614702 | 1.640 846 | 0.0 12 39 7 | 0.110252 | 0.97 | 3319 | tags=44%, list=15%, signal=52% |
| GO_NEGATIVE_REGULATION_OF_MULTI_ORGANISM_PR OCESS | 198 | 0.49202 | 1.640 808 | 0.0 16 29 3 | 0.10989 | 0.97 | 3481 | tags=38%, list=16%, signal=45% |

| | | | | | | | | |
|---|---|---|---|---|---|---|---|---|
| GO_POSITIVE_REGULATION_OF_INTERLEUKIN_1_PRODUCTION | 54 | 0.623382 | 1.63948 | 0.0144493 | 0.110657 | 0.97 | 2323 | tags=41%, list=11%, signal=46% |
| GO_PHOSPHATIDYLINOSITOL_3_4_BISPHOSPHATE_BINDING | 24 | 0.548267 | 1.639246 | 0.0333333 | 0.110518 | 0.97 | 2922 | tags=29%, list=13%, signal=34% |
| GO_MACROPHAGE_MIGRATION | 47 | 0.592201 | 1.637127 | 0.0184443 | 0.111995 | 0.97 | 2944 | tags=47%, list=14%, signal=54% |
| GO_INTERLEUKIN_4_PRODUCTION | 34 | 0.678796 | 1.637075 | 0.0160322 | 0.111696 | 0.971 | 2875 | tags=56%, list=13%, signal=64% |
| GO_POSITIVE_REGULATION_OF_INTERLEUKIN_8_PRODUCTION | 49 | 0.6157 | 1.635631 | 0.02449 | 0.112971 | 0.972 | 3826 | tags=51%, list=18%, signal=62% |
| GO_POSITIVE_REGULATION_OF_LYMPHOCYTE_MIGRATION | 36 | 0.672357 | 1.635511 | 0.0213359 | 0.112714 | 0.972 | 3172 | tags=64%, list=15%, signal=75% |
| GO_CELL_CHEMOTAXIS | 264 | 0.576952 | 1.635305 | 0.0061988 | 0.112512 | 0.972 | 2944 | tags=42%, list=14%, signal=48% |
| GO_POSITIVE_REGULATION_OF_INTERLEUKIN_8_SECRETION | 16 | 0.753508 | 1.634012 | 0.0143444 | 0.113659 | 0.973 | 3172 | tags=69%, list=15%, signal=80% |
| GO_POSITIVE_REGULATION_OF_LEUKOCYTE_CHEMOTAXIS | 79 | 0.592868 | 1.63318 | 0.0118344 | 0.113765 | 0.973 | 3083 | tags=51%, list=14%, signal=59% |

| | | | | | | | | |
|---|---|---|---|---|---|---|---|---|
| GO_NEGATIVE_REGULATION_OF_RESPONSE_TO_BIOTIC_STIMULUS | 84 | 0.528513 | 1.633117 | 0.030738 | 0.113486 | 0.973 | 3319 | tags=38%, list=15%, signal=45% |
| GO_T_CELL_HOMEOSTASIS | 38 | 0.575413 | 1.632854 | 0.025641 | 0.113412 | 0.973 | 2093 | tags=32%, list=10%, signal=35% |
| GO_REGULATION_OF_CYTOKINE_PRODUCTION_INVOLVED_IN_IMMUNE_RESPONSE | 80 | 0.632357 | 1.631929 | 0.026 | 0.114021 | 0.973 | 2711 | tags=46%, list=12%, signal=53% |
| GO_NEGATIVE_REGULATION_OF_B_CELL_PROLIFERATION | 16 | 0.745988 | 1.628264 | 0.02686 | 0.116602 | 0.976 | 2464 | tags=56%, list=11%, signal=63% |
| GO_MYELOID_CELL_DIFFERENTIATION | 355 | 0.418781 | 1.626251 | 0.016427 | 0.117242 | 0.976 | 2991 | tags=26%, list=14%, signal=30% |
| GO_HOMEOSTASIS_OF_NUMBER_OF_CELLS | 225 | 0.438113 | 1.625164 | 0.018595 | 0.117344 | 0.976 | 2723 | tags=27%, list=13%, signal=30% |
| GO_POSITIVE_REGULATION_OF_INTERLEUKIN_4_PRODUCTION | 22 | 0.722226 | 1.624901 | 0.012146 | 0.117306 | 0.976 | 2323 | tags=64%, list=11%, signal=71% |
| GO_B_CELL_HOMEOSTASIS | 28 | 0.620491 | 1.622157 | 0.04499 | 0.119606 | 0.976 | 2339 | tags=39%, list=11%, signal=44% |
| GO_NEGATIVE_REGULATION_OF_VIRAL_GENOME_REPLICATION | 55 | 0.603034 | 1.621507 | 0.036217 | 0.119957 | 0.976 | 3204 | tags=45%, list=15%, signal=53% |
| GO_REGULATION_OF_T_CELL_MEDIATED_CYTOTOXICITY | 27 | 0.710552 | 1.621153 | 0.025478 | 0.119932 | 0.977 | 2790 | tags=52%, list=13%, signal=59% |

| | | | | | | | | |
|---|---|---|---|---|---|---|---|---|
| GO_REGULATION_OF_PRODUCTION_OF_MOLECULAR_MEDIATOR_OF_IMMUNE_RESPONSE | 129 | 0.569046 | 1.619851 | 0.0020492 | 0.121155 | 0.977 | 2711 | tags=39%, list=12%, signal=44% |
| GO_REGULATION_OF_LEUKOCYTE_CHEMOTAXIS | 100 | 0.593726 | 1.619711 | 0.0016293 | 0.120941 | 0.977 | 3083 | tags=50%, list=14%, signal=58% |
| GO_B_CELL_MEDIATED_IMMUNITY | 107 | 0.586216 | 1.619169 | 0.0022449 | 0.120693 | 0.977 | 2464 | tags=44%, list=11%, signal=49% |
| GO_CELLULAR_RESPONSE_TO_BIOTIC_STIMULUS | 213 | 0.546571 | 1.61842 | 0.0020619 | 0.120742 | 0.977 | 3826 | tags=42%, list=18%, signal=51% |
| GO_SPECIFIC_GRANULE_MEMBRANE | 83 | 0.58851 | 1.617893 | 0.0030364 | 0.120913 | 0.979 | 2555 | tags=31%, list=12%, signal=35% |
| GO_RESPONSE_TO_CHEMOKINE | 85 | 0.680309 | 1.617713 | 0.0018634 | 0.120758 | 0.979 | 2780 | tags=54%, list=13%, signal=62% |
| GO_B_CELL_ACTIVATION | 224 | 0.543623 | 1.617311 | 0.0042596 | 0.120831 | 0.98 | 2593 | tags=36%, list=12%, signal=40% |
| GO_POSITIVE_REGULATION_OF_INTERLEUKIN_2_PRODUCTION | 31 | 0.675363 | 1.616585 | 0.0035928 | 0.121336 | 0.981 | 2723 | tags=48%, list=13%, signal=55% |
| GO_RECEPTOR_SIGNALING_PATHWAY_VIA_STAT | 145 | 0.456899 | 1.615501 | 0.006 | 0.121924 | 0.981 | 2984 | tags=34%, list=14%, signal=40% |

| | | | | | | | | |
|---|---|---|---|---|---|---|---|---|
| GO_POSITIVE_REGULATION_OF_RESPONSE_TO_CYTOKINE_STIMULUS | 54 | 0.580063 | 1.615101 | 0.0282833 | 0.122027 | 0.981 | 3697 | tags=43%, list=17%, signal=51% |
| GO_POSITIVE_REGULATION_OF_CALCIUM_MEDIATED_SIGNALING | 40 | 0.535194 | 1.614361 | 0.0020208 | 0.122148 | 0.981 | 4274 | tags=45%, list=20%, signal=56% |
| GO_REGULATION_OF_NIK_NF_KAPPAB_SIGNALING | 95 | 0.514965 | 1.613052 | 0.0290406 | 0.122413 | 0.981 | 3489 | tags=29%, list=16%, signal=35% |
| GO_CYTOKINE_METABOLIC_PROCESS | 114 | 0.546713 | 1.612755 | 0.0286869 | 0.12203 | 0.981 | 3063 | tags=44%, list=14%, signal=51% |
| GO_POSITIVE_REGULATION_OF_ALPHA_BETA_T_CELL_PROLIFERATION | 18 | 0.804215 | 1.612694 | 0.0124222 | 0.121751 | 0.981 | 3461 | tags=83%, list=16%, signal=99% |
| GO_POSITIVE_REGULATION_OF_NITRIC_OXIDE_SYNTHASE_BIOSYNTHETIC_PROCESS | 15 | 0.783149 | 1.609693 | 0.0142228 | 0.124516 | 0.983 | 1147 | tags=47%, list=5%, signal=49% |
| GO_REGULATION_OF_B_CELL_PROLIFERATION | 60 | 0.621963 | 1.60787 | 0.0321229 | 0.12621 | 0.985 | 2464 | tags=43%, list=11%, signal=49% |
| GO_NEGATIVE_REGULATION_OF_VIRAL_LIFE_CYCLE | 77 | 0.54812 | 1.606765 | 0.0289266 | 0.126758 | 0.985 | 3204 | tags=43%, list=15%, signal=50% |
| GO_POSITIVE_REGULATION_OF_TYPE_I_INTERFERON_PRODUCTION | 73 | 0.472138 | 1.606652 | 0.0404044 | 0.126516 | 0.985 | 1335 | tags=18%, list=6%, signal=19% |

| | | | | | | | | |
|---|---|---|---|---|---|---|---|---|
| GO_T_CELL_APOPTOTIC_PROCESS | 47 | 0.592546 | 1.606158 | 0.016701 | 0.126799 | 0.986 | 2421 | tags=40%, list=11%, signal=45% |
| GO_POSITIVE_REGULATION_OF_MYELOID_CELL_DIFFERENTIATION | 85 | 0.489004 | 1.60547 | 0.0187171 | 0.12712 | 0.986 | 2984 | tags=33%, list=14%, signal=38% |
| GO_MYELOID_LEUKOCYTE_MIGRATION | 184 | 0.582922 | 1.603419 | 0.0225541 | 0.128837 | 0.987 | 3170 | tags=45%, list=15%, signal=52% |
| GO_NEGATIVE_REGULATION_OF_CELL_ACTIVATION | 181 | 0.548694 | 1.602389 | 0.0243 9 | 0.129577 | 0.988 | 2980 | tags=45%, list=14%, signal=52% |
| GO_PROTEIN_AUTOPHOSPHORYLATION | 223 | 0.380361 | 1.598179 | 0.0263369 | 0.132719 | 0.989 | 2990 | tags=21%, list=14%, signal=24% |
| GO_RESPONSE_TO_MOLECULE_OF_BACTERIAL_ORIGIN | 317 | 0.521708 | 1.592622 | 0.0224495 | 0.138495 | 0.989 | 3466 | tags=39%, list=16%, signal=46% |
| GO_T_CELL_RECEPTOR_COMPLEX | 17 | 0.816614 | 1.591983 | 0.0138889 | 0.138538 | 0.99 | 2254 | tags=71%, list=10%, signal=79% |
| GO_TUMOR_NECROSIS_FACTOR_RECEPTOR_SUPERFAMILY_BINDING | 42 | 0.60185 | 1.591682 | 0.0368885 | 0.138507 | 0.99 | 4354 | tags=50%, list=20%, signal=62% |
| GO_REGULATION_OF_MYOTUBE_DIFFERENTIATION | 48 | 0.531148 | 1.591326 | 0.0135922 | 0.138541 | 0.99 | 2895 | tags=29%, list=13%, signal=34% |

| | | | | | | | | |
|---|---|---|---|---|---|---|---|---|
| GO_POSITIVE_REGULATION_OF_PRODUCTION_OF_MOLECULAR_MEDIATOR_OF_IMMUNE_RESPONSE | 86 | 0.561692 | 1.587644 | 0.022727 | 0.142164 | 0.991 | 2400 | tags=35%, list=11%, signal=39% |
| GO_REGULATORY_T_CELL_DIFFERENTIATION | 28 | 0.74057 | 1.587081 | 0.022312 | 0.142073 | 0.991 | 2186 | tags=61%, list=10%, signal=67% |
| GO_NEGATIVE_REGULATION_OF_T_CELL_MEDIATED_IMMUNITY | 17 | 0.667494 | 1.587078 | 0.021696 | 0.141692 | 0.991 | 2188 | tags=47%, list=10%, signal=52% |
| GO_POSITIVE_REGULATION_OF_NIK_NF_KAPPAB_SIGNALING | 70 | 0.553181 | 1.586961 | 0.033195 | 0.141448 | 0.991 | 3057 | tags=29%, list=14%, signal=33% |
| GO_MYELOID_DENDRITIC_CELL_ACTIVATION | 26 | 0.700851 | 1.58645 | 0.03681 | 0.141749 | 0.991 | 2613 | tags=62%, list=12%, signal=70% |
| GO_REGULATION_OF_T_CELL_CYTOKINE_PRODUCTION | 30 | 0.667841 | 1.586172 | 0.02449 | 0.141297 | 0.991 | 2323 | tags=43%, list=11%, signal=48% |
| GO_POSITIVE_REGULATION_OF_PHAGOCYTOSIS | 65 | 0.567212 | 1.585233 | 0.020161 | 0.142067 | 0.992 | 3172 | tags=46%, list=15%, signal=54% |
| GO_INTERLEUKIN_1_SECRETION | 55 | 0.614199 | 1.585216 | 0.029228 | 0.14171 | 0.992 | 2323 | tags=45%, list=11%, signal=51% |
| GO_NEGATIVE_REGULATION_OF_ALPHA_BETA_T_CELL_ACTIVATION | 34 | 0.588758 | 1.583031 | 0.030675 | 0.143417 | 0.992 | 2188 | tags=47%, list=10%, signal=52% |

| | | | | | | | | |
|---|---|---|---|---|---|---|---|---|
| GO_POSITIVE_REGULATION_OF_ANTIGEN_RECEPTOR_MEDIATED_SIGNALING_PATHWAY | 19 | 0.755182 | 1.582902 | 0.03272 | 0.14318 | 0.992 | 979 | tags=47%, list=5%, signal=50% |
| GO_LYMPHOCYTE_CHEMOTAXIS | 56 | 0.694839 | 1.582657 | 0.028398 | 0.143083 | 0.992 | 2421 | tags=52%, list=11%, signal=58% |
| GO_GLIAL_CELL_ACTIVATION | 51 | 0.654791 | 1.581665 | 0.030426 | 0.143926 | 0.992 | 1731 | tags=43%, list=8%, signal=47% |
| GO_NEGATIVE_REGULATION_OF_INTERLEUKIN_12_PRODUCTION | 16 | 0.767695 | 1.579399 | 0.02449 | 0.145672 | 0.992 | 3535 | tags=75%, list=16%, signal=90% |
| GO_REGULATION_OF_MONONUCLEAR_CELL_MIGRATION | 41 | 0.629825 | 1.579095 | 0.017822 | 0.145665 | 0.992 | 2984 | tags=54%, list=14%, signal=62% |
| GO_POSITIVE_REGULATION_OF_SMALL_GTPASE_MEDIATED_SIGNAL_TRANSDUCTION | 66 | 0.509074 | 1.577991 | 0.028283 | 0.145622 | 0.992 | 2466 | tags=33%, list=11%, signal=37% |
| GO_POSITIVE_REGULATION_OF_INTERLEUKIN_6_PRODUCTION | 83 | 0.556282 | 1.577892 | 0.039501 | 0.145385 | 0.992 | 3406 | tags=43%, list=16%, signal=51% |
| GO_ALPHA_BETA_T_CELL_PROLIFERATION | 30 | 0.681822 | 1.57627 | 0.028866 | 0.145284 | 0.992 | 3461 | tags=70%, list=16%, signal=83% |
| GO_NEGATIVE_REGULATION_OF_LYMPHOCYTE_ACTIVATION | 137 | 0.544893 | 1.575841 | 0.035343 | 0.144348 | 0.992 | 2944 | tags=46%, list=14%, signal=53% |

| | | | | | | | | |
|---|---|---|---|---|---|---|---|---|
| GO_THYMIC_T_CELL_SELECTION | 21 | 0.722722 | 1.575607 | 0.0030242 | 0.14426 | 0.992 | 2254 | tags=52%, list=10%, signal=58% |
| GO_INTERLEUKIN_2_BIOSYNTHETIC_PROCESS | 23 | 0.680165 | 1.575528 | 0.0022312 | 0.143973 | 0.992 | 2254 | tags=52%, list=10%, signal=58% |
| GO_PHOSPHATIDYLINOSITOL_3_4_5_TRISPHOSPHATE_BINDING | 34 | 0.449661 | 1.572448 | 0.0043478 | 0.146676 | 0.994 | 1202 | tags=15%, list=6%, signal=16% |
| GO_PATTERN_RECOGNITION_RECEPTOR_ACTIVITY | 21 | 0.723451 | 1.571554 | 0.0029474 | 0.147165 | 0.994 | 1387 | tags=48%, list=6%, signal=51% |
| GO_NEGATIVE_REGULATION_OF_INTERFERON_GAMMA_PRODUCTION | 30 | 0.645357 | 1.570975 | 0.0014768 | 0.147574 | 0.995 | 2921 | tags=57%, list=13%, signal=65% |
| GO_NEGATIVE_REGULATION_OF_VIRAL_PROCESS | 93 | 0.507962 | 1.569751 | 0.0043121 | 0.148053 | 0.995 | 3204 | tags=39%, list=15%, signal=45% |
| GO_SH2_DOMAIN_BINDING | 35 | 0.558832 | 1.567668 | 0.0044898 | 0.150348 | 0.995 | 2626 | tags=31%, list=12%, signal=36% |
| GO_IMMUNOGLOBULIN_PRODUCTION | 98 | 0.541153 | 1.56714 | 0.0046559 | 0.1507 | 0.995 | 2464 | tags=32%, list=11%, signal=36% |
| GO_GRANULOCYTE_MIGRATION | 122 | 0.598001 | 1.565807 | 0.0024793 | 0.15203 | 0.995 | 3406 | tags=48%, list=16%, signal=57% |

| | | | | | | | | |
|---|---|---|---|---|---|---|---|---|
| GO_REGULATION_OF_PHAGOCYTOSIS | 87 | 0.530417 | 1.564345 | 0.0274426 | 0.153613 | 0.995 | 2984 | tags=43%, list=14%, signal=49% |
| GO_LEUKOCYTE_APOPTOTIC_PROCESS | 101 | 0.534878 | 1.563196 | 0.0410668 | 0.153987 | 0.995 | 2780 | tags=39%, list=13%, signal=44% |
| GO_POSITIVE_REGULATION_OF_CHEMOTAXIS | 126 | 0.547487 | 1.561407 | 0.02454 | 0.1559 | 0.995 | 3216 | tags=43%, list=15%, signal=50% |
| GO_POSITIVE_REGULATION_OF_CALCIUM_ION_TRANSPORT_INTO_CYTOSOL | 50 | 0.564414 | 1.558949 | 0.0201121 | 0.158088 | 0.996 | 2513 | tags=40%, list=12%, signal=45% |
| GO_NEGATIVE_REGULATION_OF_LEUKOCYTE_CELL_CELL_ADHESION | 111 | 0.540662 | 1.558947 | 0.0253316 | 0.157716 | 0.996 | 3064 | tags=46%, list=14%, signal=53% |
| GO_DEFENSE_RESPONSE_TO_BACTERIUM | 209 | 0.468984 | 1.558392 | 0.0061222 | 0.158131 | 0.996 | 3835 | tags=43%, list=18%, signal=51% |
| GO_CYTOKINE_PRODUCTION_INVOLVED_IN_INFLAMMATORY_RESPONSE | 42 | 0.575466 | 1.557872 | 0.04829 | 0.158464 | 0.996 | 3826 | tags=43%, list=18%, signal=52% |
| GO_POSITIVE_REGULATION_OF_T_CELL_MEDIATED_IMMUNITY | 43 | 0.615147 | 1.55733 | 0.0467748 | 0.158107 | 0.996 | 2790 | tags=42%, list=13%, signal=48% |
| GO_REGULATION_OF_VIRAL_LIFE_CYCLE | 134 | 0.452043 | 1.550675 | 0.0456443 | 0.164278 | 0.997 | 3204 | tags=31%, list=15%, signal=36% |

| | | | | | | | | |
|---|---|---|---|---|---|---|---|---|
| GO_LYMPHOCYTE_APOPTOTIC_PROCESS | 70 | 0.545867 | 1.549659 | 0.0321189 | 0.165285 | 0.997 | 2464 | tags=37%, list=11%, signal=42% |
| GO_PURINERGIC_RECEPTOR_ACTIVITY | 25 | 0.673012 | 1.548467 | 0.0220888 | 0.166165 | 0.997 | 1731 | tags=56%, list=8%, signal=61% |
| GO_POSITIVE_REGULATION_OF_INTERLEUKIN_10_PRODUCTION | 33 | 0.713652 | 1.545691 | 0.0447175 | 0.16782 | 0.997 | 3406 | tags=64%, list=16%, signal=75% |
| GO_ACUTE_INFLAMMATORY_RESPONSE_TO_ANTIGENIC_STIMULUS | 18 | 0.700452 | 1.542931 | 0.028112 | 0.170734 | 0.997 | 1763 | tags=50%, list=8%, signal=54% |
| GO_POSITIVE_REGULATION_OF_RELEASE_OF_SEQUESTERED_CALCIUM_ION_INTO_CYTOSOL | 36 | 0.589364 | 1.536248 | 0.0308829 | 0.174799 | 0.997 | 2466 | tags=39%, list=11%, signal=44% |
| GO_POSITIVE_REGULATION_OF_MONOCYTE_CHEMOTAXIS | 17 | 0.739504 | 1.534826 | 0.032454 | 0.174953 | 0.997 | 1587 | tags=59%, list=7%, signal=63% |
| GO_NEUTROPHIL_MIGRATION | 102 | 0.622646 | 1.534672 | 0.035052 | 0.174778 | 0.997 | 3721 | tags=54%, list=17%, signal=65% |
| GO_POSITIVE_REGULATION_OF_JNK_CASCADE | 124 | 0.430747 | 1.533806 | 0.039337 | 0.175188 | 0.998 | 3470 | tags=29%, list=16%, signal=34% |
| GO_REGULATION_OF_MONOCYTE_CHEMOTAXIS | 23 | 0.714487 | 1.529388 | 0.034068 | 0.179402 | 0.998 | 2636 | tags=65%, list=12%, signal=74% |

| | | | | | | | | |
|---|---|---|---|---|---|---|---|---|
| GO_TYROSINE_PHOSPHORYLATION_OF_STAT_PROTEIN | 79 | 0.486878 | 1.528263 | 0.031683 | 0.180626 | 0.998 | 2984 | tags=39%, list=14%, signal=45% |
| GO_MONONUCLEAR_CELL_MIGRATION | 80 | 0.63763 | 1.527834 | 0.040323 | 0.180513 | 0.998 | 2984 | tags=50%, list=14%, signal=58% |
| GO_NEGATIVE_REGULATION_OF_IMMUNE_RESPONSE | 140 | 0.515637 | 1.525324 | 0.035327 | 0.183325 | 0.998 | 2984 | tags=41%, list=14%, signal=47% |
| GO_NEGATIVE_REGULATION_OF_ADAPTIVE_IMMUNE_RESPONSE | 44 | 0.551975 | 1.525016 | 0.03252 | 0.183346 | 0.998 | 2188 | tags=41%, list=10%, signal=45% |
| GO_NEUROINFLAMMATORY_RESPONSE | 63 | 0.60858 | 1.521846 | 0.044807 | 0.186036 | 0.998 | 3303 | tags=52%, list=15%, signal=62% |
| GO_HOMEOSTASIS_OF_NUMBER_OF_CELLS_WITHIN_A_TISSUE | 27 | 0.539376 | 1.521634 | 0.047131 | 0.185627 | 0.998 | 2200 | tags=37%, list=10%, signal=41% |
| GO_REGULATION_OF_CALCIUM_ION_TRANSPORT | 228 | 0.435123 | 1.520519 | 0.011719 | 0.185308 | 0.998 | 2698 | tags=31%, list=12%, signal=35% |
| GO_NEGATIVE_REGULATION_OF_LEUKOCYTE_MEDIATED_CYTOTOXICITY | 15 | 0.711046 | 1.51966 | 0.046748 | 0.185677 | 0.998 | 2711 | tags=53%, list=12%, signal=61% |
| GO_DEFENSE_RESPONSE_TO_GRAM_POSITIVE_BACTERIUM | 78 | 0.500116 | 1.515437 | 0.012346 | 0.18825 | 0.998 | 2984 | tags=33%, list=14%, signal=38% |

| | | | | | | | | |
|---|---|---|---|---|---|---|---|---|
| GO_MONOCYTE_CHEMOTAXIS | 59 | 0.683522 | 1.513854 | 0.0386697 | 0.188747 | 0.998 | 2780 | tags=54%, list=13%, signal=62% |
| GO_NEGATIVE_REGULATION_OF_CELL_CELL_ADHESION | 159 | 0.48915 | 1.508935 | 0.0373344 | 0.194434 | 0.998 | 3064 | tags=38%, list=14%, signal=44% |
| GO_NEGATIVE_REGULATION_OF_INTERLEUKIN_2_PRODUCTION | 23 | 0.628044 | 1.504912 | 0.0383384 | 0.197137 | 0.998 | 2188 | tags=43%, list=10%, signal=48% |
| GO_POSITIVE_REGULATION_OF_MONONUCLEAR_CELL_MIGRATION | 22 | 0.669835 | 1.503565 | 0.0409 | 0.197233 | 0.998 | 1587 | tags=45%, list=7%, signal=49% |
| GO_POSITIVE_REGULATION_OF_RECEPTOR_SIGNALING_PATHWAY_VIA_STAT | 87 | 0.486268 | 1.502135 | 0.0303364 | 0.199045 | 0.998 | 2984 | tags=43%, list=14%, signal=49% |
| GO_REGULATION_OF_CHEMOTAXIS | 191 | 0.489947 | 1.498151 | 0.0386697 | 0.203468 | 0.998 | 3234 | tags=37%, list=15%, signal=43% |
| GO_NEGATIVE_REGULATION_OF_LEUKOCYTE_MEDIATED_IMMUNITY | 46 | 0.571005 | 1.49647 | 0.0267749 | 0.204034 | 0.998 | 2188 | tags=43%, list=10%, signal=48% |
| GO_POSITIVE_REGULATION_OF_CALCIUM_ION_TRANSPORT | 112 | 0.458319 | 1.492927 | 0.0236622 | 0.206657 | 0.998 | 2696 | tags=32%, list=12%, signal=37% |
| GO_ACTIVATION_OF_PHOSPHOLIPASE_C_ACTIVITY | 31 | 0.597164 | 1.488505 | 0.0263316 | 0.209971 | 0.998 | 3785 | tags=58%, list=17%, signal=70% |

| | | | | | | | | |
|---|---|---|---|---|---|---|---|---|
| GO_NEGATIVE_REGULATION_OF_HEMOPOIESIS | 132 | 0.434857 | 1.483592 | 0.0440025 | 0.214753 | 0.999 | 2339 | tags=28%, list=11%, signal=31% |
| GO_NEGATIVE_REGULATION_OF_LEUKOCYTE_DIFFERENTIATION | 95 | 0.469487 | 1.478162 | 0.0485523 | 0.221122 | 0.999 | 3112 | tags=38%, list=14%, signal=44% |
| GO_VITAMIN_D_METABOLIC_PROCESS | 22 | 0.542598 | 1.422083 | 0.0436651 | 0.275781 | 1 | 4070 | tags=55%, list=19%, signal=67% |
